# A Single Bioorthogonal Reaction for Multiplex Cell Surface Protein Labeling

**DOI:** 10.1021/jacs.4c11701

**Published:** 2025-01-03

**Authors:** Yang Huang, Chengyang Wu, Anjing Lu, Jingzhe Wang, Jian Liang, Han Sun, Liqing Yang, Shixiang Duan, Andrey A. Berezin, Chuanliu Wu, Bo Zhang, Yi-Lin Wu, Yu-Hsuan Tsai

**Affiliations:** † School of Basic Medical Sciences, Capital Medical University, Beijing 100069, China; ‡ Institute of Molecular Physiology, Shenzhen Bay Laboratory, Shenzhen 518132, China; § School of Chemistry, Cardiff University, Cardiff CF10 3AT, United Kingdom; ∥ College of Chemistry and Pharmacy, Northwest A&F University, Yangling 712100, China; ⊥ Institute of Neurological and Psychiatric Disorders, Shenzhen Bay Laboratory, Shenzhen 518132, China; # School of Chemical Biology and Biotechnology, Peking University Shenzhen Graduate School, Shenzhen 518055, China; ∇ Department of Chemistry, College of Chemistry and Chemical Engineering, The MOE Key Laboratory of Spectrochemical Analysis and Instrumentation, State Key Laboratory of Physical Chemistry of Solid Surfaces, Xiamen University, Xiamen 361005, China

## Abstract

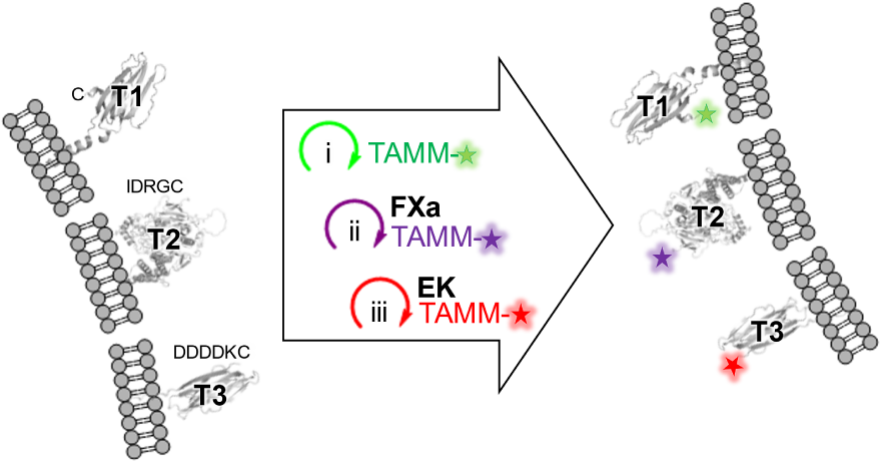

Small-molecule fluorophores are invaluable tools for fluorescence imaging. However, means for their covalent conjugation to the target proteins limit applications in multicolor imaging. Here, we identify 2-[(alkyl**t**hio)(**a**ryl)**m**ethylene]**m**alononitrile (TAMM) molecules reacting with 1,2-aminothiol at a labeling rate over 10^4^ M^–1^ s^–1^ through detailed mechanistic investigation. The fast TAMM molecules and mild reaction conditions enable site-specific labeling of surface proteins in not only cell lines but also primary neurons and living mice. The combination of genetic code expansion and sequence-specific proteolytic cleavage enables selective modification of three different cell surface proteins through iterative TAMM condensation. TAMM condensation is also compatible with Cu-catalyzed azide–alkyne cycloaddition and tetrazine ligation for four-color fluorescent labeling, reaching the maximum available colors of conventional confocal microscopes. Thus, bioconjugation chemistry is no longer the limiting factor for multiplex cell surface protein imaging.

## Introduction

Proteins are critical biomolecules playing essential roles in living organisms. Their functions are often associated with their location, transportation and interaction with other biomolecules. While fluorescence imaging can reveal these properties, most proteins cannot be directly visualized, so attachment of a fluorescent protein or small molecule is needed.^1,2^ Genetic fusion of the target protein with a fluorescent protein is the most widely used strategy by biologists, and multicolor fluorescence imaging can be easily achieved by fusing different targets with varying fluorescent proteins.^1^ However, fluorescent proteins are much larger (ca. 27 kDa or 230 amino acid residues) than small-molecule fluorophores (ca. 0.5 kDa). The large size may affect the protein functions and interactions with other molecules.^3−6^ In addition, fluorescent proteins are less suitable for super-resolution imaging. The large size reduces the localization precision of the target.^7^ It is also challenging to engineer extremely bright and photostable fluorescent proteins for super-resolution imaging based on stimulated emission depletion (STED) and stochastic optical reconstruction microscopy (STORM), which mainly rely on small-molecule fluorophores.

Small-molecule fluorophores can be specifically attached to the target proteins through antibody conjugates (ca. 1300 amino acid residues), affinity tags (e.g., SNAP-tag and HaloTag, ca. 180–300 amino acid residues), enzymes (e.g., SrtA or *Oa*AEP1 recognizing specific sequences of 3–5 amino acid residues) or genetically incorporated bioorthogonal amino acids (i.e., one amino acid residue).^8−13^ The last approach best preserves the size advantage of small-molecule fluorophores as conjugation takes place through a single unnatural amino acid residue via a bioorthogonal reaction, such as Cu-catalyzed azide–alkyne cycloaddition (CuAAC) or tetrazine ligation.^14,15^ However, it is challenging to label more than two targets by this approach. For each additional target, a new reaction is required, as well as a new blank codon and the corresponding orthogonal aminoacyl-tRNA synthetase/tRNA pair. Importantly, each of these must not interfere with the existing ones.

**Figure 1:**
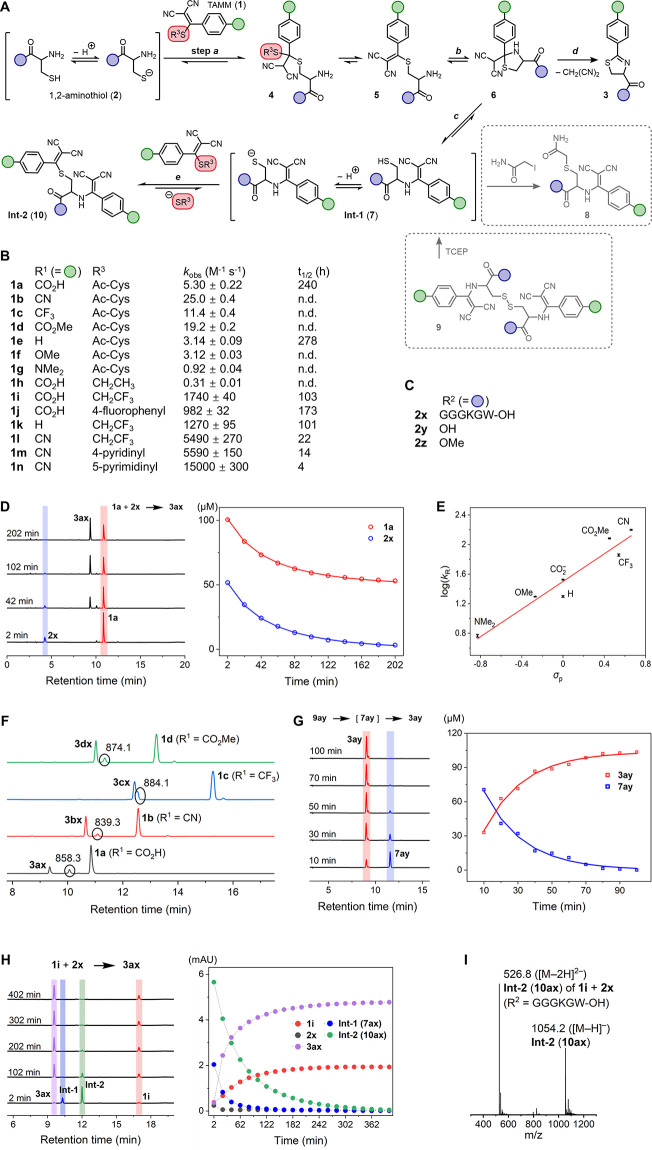
Mechanistic study of TAMM condensation. (A) TAMM condensation mechanism elucidated in this work with two identifiable intermediates **Int-1** and **Int-2**. (B) TAMM molecules employed in mechanistic and kinetic studies. The observed consumption rate constants () at 25 °C and pH 7.4 correspond to reaction with **2x**. The stability of TAMM is indicated by the half-life (*t*_1/2_) in PBS (10 mM, pH 7.4) at 37 °C. n.d. = not determined. (C) 1,2-Aminothiol molecules employed in mechanistic and kinetic studies. (D) A representative TAMM condensation between **1a** and model peptide **2x** to form dihydrothiazole **3ax**. Left: HPLC chromatograms of the reaction mixture analyzed at the indicated time point (λ_monitor_ = 280 nm). Right: time evolution of [**1a**] and [**2x**] (empty circles: data points, solid lines: fitting curves to the model of second-order reaction). (E) Effect of TAMM *para* substitution (R^1^) on reaction kinetics. Hammett plot of rate constants for the consumption of **1** and **2x** versus the Hammett substituent constants σ_p_ at 25 °C; slope ρ = 0.93. (F) Formation of an intermediate in TAMM condensation. HPLC chromatograms for reactions between **1a**–**1d** and **2x** at 42 min. Peak of the intermediates is indicated with a black circle with the *m*/*z* value of the quasi molecular ion (either as [M + H]^+^ or [M–H]^−^) shown. (G) Formation of dihydrothiazole **3ay** from disulfide **9ay** by TCEP reduction. Left: HPLC chromatograms for the reaction mixture analyzed at the time specified. Right: time course of the concentrations of **7ay** and **3ay** (empty circles: data points, solid lines: fitting curves to the model of first-order kinetics). (H) Reaction of TAMM **1i** with peptide **2x** to form dihydrothiazole **3ax**. Left: HPLC chromatograms at the time specified. Right: time course of the HPLC peak intensities. The areas under the peaks, as opposed to the concentrations, are shown here as the calibration of **Int-1**, an unstable intermediate, is not available. The dashed lines connecting data points serve as a guide to the eye. (I) Formation of **Int-2** (**10**) for fast-reacting TAMM supported by mass spectrum of **10ax** for reactions involving **1i** with peptide **2x**.

Here, we demonstrate that fluorescent labeling by 2-((alkyl**t**hio)(**a**ryl)**m**ethylene)**m**alononitrile (TAMM) condensation can break the current maximum color number limit of bioorthogonal reaction-based labeling. TAMM molecules react with 1,2-aminothiol functionality, which can be introduced to the target protein as the N-terminal cysteine or through a genetically incorporated unnatural amino acid.^16^ In this work, we performed a detailed mechanistic study (Figure 1) and achieved a three-order-of-magnitude enhancement in the labeling rate constant, reaching over 10^4^ M^–1^ s^–1^. The mild reaction conditions warrant the use of TAMM condensation for labeling proteins on not only cell lines but also primary neurons. Using genetic code expansion and sequence-specific proteolytic cleavages, we demonstrated the selective modification of three different surface proteins on live mammalian cells through iterative TAMM condensation. We also demonstrated the compatibility of TAMM condensation to CuAAC and tetrazine ligation, as well as their application in four-color fluorescent labeling. Theoretically, the combination of TAMM condensation, CuAAC and tetrazine ligation allows sequential one-pot labeling of five different target proteins through two sequence-specific proteolytic cleavages and three genetically incorporated unnatural amino acids. Lastly, TAMM condensation is also applicable for in vivo labeling, and its combination with tetrazine ligation allows two-color fluorescent labeling in living mice.

## Results

### Mechanistic Insights and Kinetic Enhancement of TAMM Condensation

The kinetics of the condensation reactions between TAMM compounds (**1a**–**g**) and a N-terminal cysteine peptide (**2x**) was studied using HPLC. Taking **1a** and **2x** as the model reactants, their consumption follows a simple second-order kinetics: rate = , where the p*K*_a_ is the acid-dissociation constant of the thiol group (Figures 1D and S1 and Table S1). The pH-dependent fraction factor relates the observed rate constant *k*_obs_ to the intrinsic, pH-independent constant *k* by accounting for the thiolate concentration. This simple kinetic scheme suggests that subsequent reactions of **1a** and **2x** after the initial bimolecular encounter occur readily, without complications from backward reactions.

A range of *k* = 6.3–170 M^–1^ s^–1^ was found by varying the *para* substituents of TAMM reagents (Figure S3), with electron-withdrawing substituents accelerating the reactant consumption. A Hammett plot yielded ρ = 0.93 (Figure 1E), suggesting negative charge buildup in the transition state, likely around the benzylic position. Eyring analysis gave Δ*G*^‡^ = 15.4 ± 0.1 kcal mol^–1^ for the reaction between **1a** and **2x** (Figure S2 and Table S2), comparable to the DFT-computed barrier for a stepwise thiolate-exchange mechanism, where an early transition state (**TS-1’**) was found for the formation of tetrahedral intermediate **4′**, which collapses to **5′** (Figure S3). The computed activation free energy was lowest for acceptor-substituted **1b′** (R^1^ = CN, Δ*G*^‡^_calc_ = 12.30 kcal mol^–1^) and highest for donor-substituted **1g′** (R^1^ = NMe_2_, Δ*G*^‡^_calc_ = 17.48 kcal mol^–1^), aligning with the Hammett analysis.

During kinetic studies, an intermediate species was observed (Figure 1F). Mass spectrometry revealed *m*/*z* 858.3 for the intermediate between **1a** and **2x**, 66 mass units higher than product **3ax** (*m*/*z* 792.3), suggesting addition of a malononitrile unit to the dihydrothiazole product (Figure S4). This *m*/*z* value can correspond to either *S*-linked structure **5**, cyclic thiazolidine **6** or *N*-linked **7** (Figure 1A). DFT calculations of truncated structures **5′**–**7′** indicated that *N*-linked **7′** had the lowest energy (Figure S5). In addition, the intermediate can be trapped by iodoacetamide (Figures S6–S10), and reduction of synthesized disulfide compound **9ay** resulted in an HPLC peak identical to the intermediate observed in the reaction of cysteine **2y** with **1a** (Figure 1G). These results support the *N*-linked species (**7**, termed **Int-1**) as the observed intermediate and imply rapid conversion of *S*-linked **5** into lower energy isomers, a crucial condition for the observed simple second-order kinetics in the labeling of peptide **2x** with TAMM **1a–1h**. Moreover, transformation of **Int-1** to product **3** was unimolecular and unaffected by other 1,2-aminothiol molecules (Figure S11), with Δ*G*^‡^ = 21.80 ± 0.39 kcal mol^–1^ for **3ay** formation from *in situ* generated **7ay** (Figure S12 and Table S3). This value is comparable to the calculated Δ*G*^‡^_calc_ = 19.91 kcal mol^–1^ for malononitrile elimination from cyclic thiazolidine to form dihydrothiazole, involving an ion-pairing, late transition state (Figure S13). It should be noted that, as the reaction involves two distinct stages with apparent second- and first-order kinetics, the formation rate and rate constant of dihydrothiazole (the product) cannot be easily characterized and compared. Specifically, the concentrations of the starting materials, as well as their consumption rate constant, affect the rate of product formation (Figure S12) and may be further complicated by the involvement of additional intermediates (*vide infra*).

We further noticed that the TAMM consumption rate can be greatly accelerated by using low-p*K*a thiol leaving groups. As these fast-reacting TAMM compounds (**1i**–**1n**) were consumed immediately upon mixing with cysteine, their consumption rate constants () were estimated by competition with 3-acetoxy-2-(formylphenyl)boronic acid (Figures S14 and S15).^17−19^ These TAMM molecules all reacted faster than 3-acetoxy-2-(formylphenyl)boronic acid. Their values of  ranged from ∼980 to 15000 M^–1^ s^–1^ (Figure 1B), exceeding the rate constant of CuAAC (∼10–200 M^–1^ s^–1^) and comparable to the rate constant of tetrazine ligation (∼10^2^–10^4^ M^–1^ s^–1^).^14^

Detailed analysis of reactions involving these more reactive TAMM reagents revealed a second intermediate (**10**, termed **Int-2**) composed of two [(phenyl)methylene]malononitrile units attached to an 1,2-aminothiol unit as suggested by mass spectrometry and NMR spectroscopy (Figures 1H, I and S16–S18). This species forms when excess TAMM reacts with the *N*-linked intermediate (**Int-1**). However, the thiolates removed from the initial process can react with **Int-2** to regenerate **Int-1** and TAMM. This explains why in reaction of **2x** and **1i** (Figure 1H), almost no **1i** was found at 2 min, but [**1i**] gradually increased from 2 to 102 min, while [**Int-2**] decreased during the same period.

Based on these findings, we propose a detailed reaction mechanism for TAMM condensation (Figure 1A). The process begins with thiolate capture by TAMM (step *a*), a step significantly influenced by pH, p*K*_a_ of the leaving group, and the electronic nature of the aromatic unit. This thiolate exchange likely proceeds through a tetrahedral intermediate **4** rather than a single-step, sigma-bond-metathesis transition state. The initial *S*-linked intermediate **5** rapidly rearranges to form cyclic thiazolidine **6** and subsequently *N*-linked **Int-1** (**7**) through steps *b* and *c*. These rearrangements are kinetically competitive with the elimination of malononitrile from **6**, which leads to the formation of product **3** (step *d*). The involvement of *N*-linked **Int-1** is supported by disulfide reduction of **9** and our trapping experiments using iodoacetamide. In the presence of excess TAMM reagents with low-p*K*_a_ leaving groups, **Int-1** can become **Int-2** (**10**, step *e*), similar to the iodoacetamide trapping. Although the participation of **Int-1** and **Int-2** delays the final product formation, from a practical standpoint of bioconjugation reactions, these intermediates inevitably become the dihydrothiazole product. This claim is supported by the experimental observation that *in situ* reduction of **9y** to **7y** in the presence of 10–100 equiv of **2x** or **2z** did not yield any **3x** or **3z** from label shuffling (Figure S11), indicating **6** would not revert to **5** and step *b* is practically unidirectional. Thus, the experimentally measured consumption rate, roughly corresponding to the overall rate of steps *a* and *b*, should align with the labeling efficiency and be most critical for the applications of TAMM condensation.

Additionally, TAMM reagents showed good aqueous stability (Figures 1B and S19), with half-lives ranging from 4 h (**1n**) to 278 h (**1e**). Generally, more reactive TAMM molecules were less stable, but not strictly linearly. On the other hand, neither TAMM nor malononitrile (i.e., byproduct from the formation of dihydrothiazole **3**) affected the viability of HEK293T cells at concentrations up to 0.5 mM (Figure S20). Overall, we recommend TAMM with trifluoroethanethiolate leaving group for its fast kinetics, good aqueous stability and lack of toxicity, important features for biological applications.

### Fast TAMM Condensation for Live-Cell Protein Labeling

**Figure 2:**
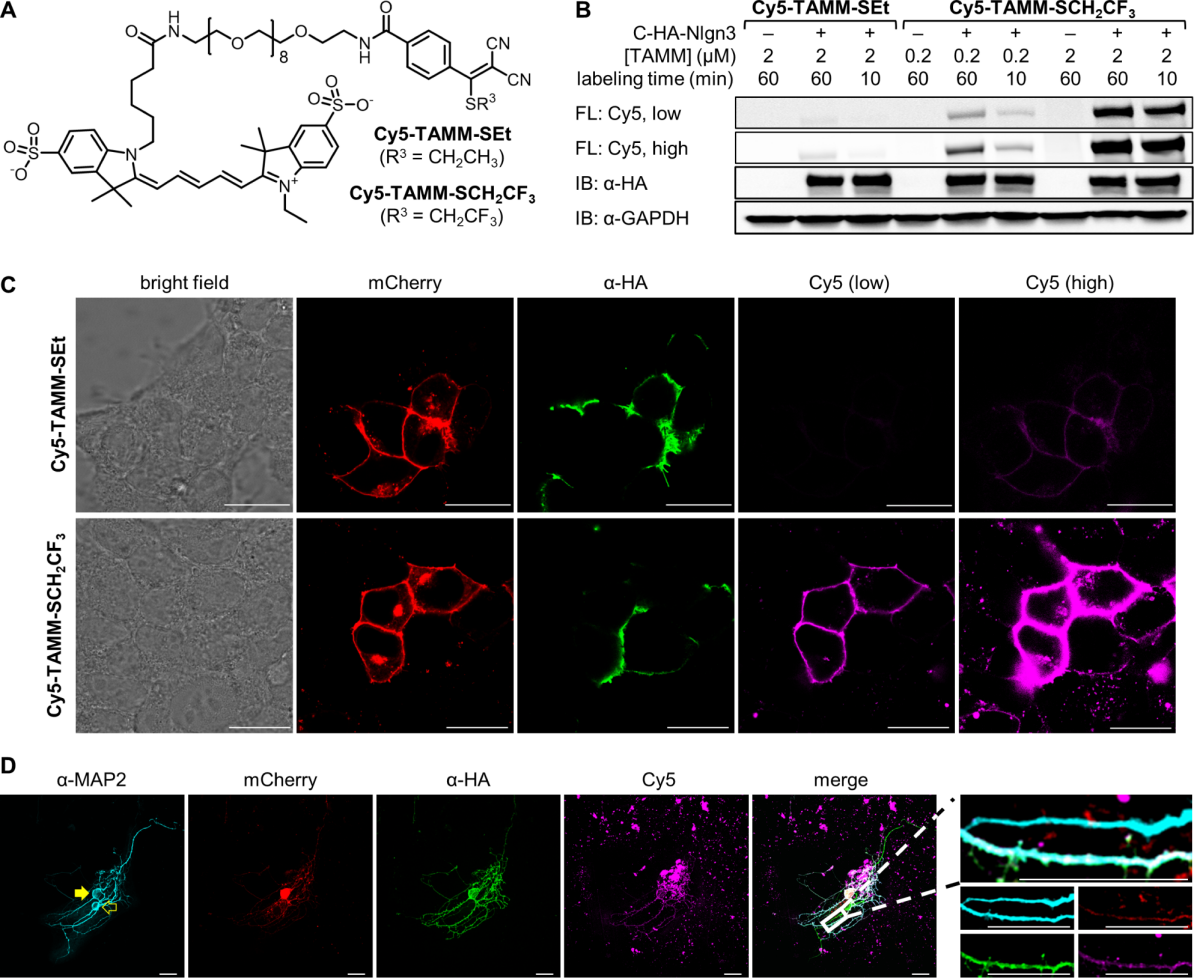
Comparison of ethanethiol or 2,2,2-trifluoroethanethiol as the leaving group in TAMM conjugates for fluorescent labeling of a cell-surface protein on live mammalian cells. (A) Structure of **Cy5-TAMM-SEt** and **Cy5-TAMM-SCH**_**2**_**CF**_**3**_. (B) In-gel fluorescence analysis of HEK293T cells overexpressing C-HA-Nlgn3 labeled with **Cy5-TAMM-SEt** or **Cy5-TAMM-SCH**_**2**_**CF**_**3**_. FL: fluorescence; low: low exposure (0.5 s); high: high exposure (10 s); IB: immunoblotting. Quantification of the fluorescence intensity is shown in Figure S21B. (C) Representative confocal microscopy images of HEK293T cells overexpressing C-HA-Nlgn3-mCherry labeled with 2 μM **Cy5-TAMM-SEt** or **Cy5-TAMM-SCH**_**2**_**CF**_**3**_ for 60 min. Merged images and two other sets of data are shown in Figure S22. (D) Representative confocal microscopy images of primary mouse cortical neurons overexpressing C-HA-Nlgn3-mCherry labeled with **Cy5-TAMM-SCH**_**2**_**CF**_**3**_. Transfected and nontransfected neurons are shown in solid and hollow yellow arrows. The zoom-in section shows labeling of the transfected neuron (i.e., mCherry positive) by TAMM labeling and α-HA immunostaining. Scale bars of all images = 20 μm.

Cell-impermeable fluorophore conjugates are ideal for labeling cell-surface proteins as excess dyes can be readily removed by changing the culture media. To compare the effect of the TAMM leaving group on labeling efficiency, we connected negatively charged sulfo-Cy5 via a PEG9 linker to different TAMM molecules. The presence of negative charge and the hydrophilic linker shall warrant the conjugates as cell impermeable. We first compared the labeling efficiency of **Cy5-TAMM-SCH**_**2**_**CF**_**3**_ to our previously employed fluorophore conjugate **Cy5-TAMM-SEt**.^16^ HEK293T cells were transfected to express a model cell surface protein C-HA-Nlgn3, which contains an N-terminal signal peptide, followed by a cysteine residue (C), HA tag for immunoblotting, and cell surface protein neuroligin 3 (Nlgn3). Upon secretion of the N-terminal to extracellular space, the signal peptide of the fusion protein is removed to expose cysteine residue and its 1,2-aminothiol functionality.^20^ Under all tested conditions, labeling of HEK293T cells overexpressing C-HA-Nlgn3 with **Cy5-TAMM-SCH**_**2**_**CF**_**3**_ led to higher fluorescence signals than **Cy5-TAMM-SPhF** (Figure S21) and **Cy5-TAMM-SEt** (Figure 2A). Importantly, no Cy5 fluorescence was detected for nontransfected cells treated with either TAMM conjugates (e.g., lanes 1/4/7 of Figure 2B), whereas overexpressed C-HA-Nlgn3 could be labeled by the TAMM conjugate, indicating the specificity of TAMM condensation. It is also noteworthy that the relative labeling signals by **Cy5-TAMM-SCH**_**2**_**CF**_**3**_, **Cy5-TAMM-SPhF**, or **Cy5-TAMM-SEt** aligned with the trend of the consumption rate constants of **1i**, **1j**, and **1h**.

We then performed confocal microscopy of HEK293T cells overexpressing C-HA-Nlgn3-mCherry labeled with 2 μM **Cy5-TAMM-SEt** or **Cy5-TAMM-SCH**_**2**_**CF**_**3**_ for 60 min (Figures 2C and S22). Most C-HA-Nlgn3-mCherry localized on the cell membrane, and the cell-surface Nlgn3 was labeled by the TAMM conjugates, with much stronger fluorescence signal observed with **Cy5-TAMM-SCH**_**2**_**CF**_**3**_ than **Cy5-TAMM-SEt**. While intracellular Nlgn3 should not be labeled due to cell impermeability of the TAMM conjugates, we observed some intracellular Cy5 fluorescence signals, likely resulting from endocytosis of the labeled Nlgn3 from the cell surface. On the other hand, no labeling was observed in nontransfected cells (i.e., those lacking mCherry fluorescence), further validating the specificity of the reaction. Notably, Cy5 fluorescence closely resembled that of membrane-localized red fluorescent protein mCherry, while α-HA immunostaining after cell fixation by paraformaldehyde did not. This is likely due to the large size of antibodies preventing antigen recognition in tight cell–cell junctions, showcasing the advantage of using bioorthogonal reactions in fluorescence labeling.

In addition to HEK293T cells, we also demonstrated the use of TAMM condensation for fluorescent labeling in other cell lines, including HeLa (Figure S23), MCF7 (Figure S24), ND7/23 (Figure S25), and SK-OV-3 (Figure S26). With success in the ND7/23 neuronal cell line, we then tested TAMM labeling of Nlgn3 in primary neurons, in which endogenous Nlgn3 plays essential roles for synapse function^21,22^ and associates with autism.^23^ Cultured neurons were transfected to express mCherry and C-HA-Nlgn3 connected by a self-cleaving P2A sequence so that mCherry signal denotes cells expressing C-HA-Nlgn3. We also performed immunostaining against MAP2 to confirm the neuronal nature of the cells. The transfected neuron (i.e., mCherry^+^/MAP2^+^, solid yellow arrow of Figure 2D) also showed Cy5 and α-HA fluorescence, while the nontransfected neuron (i.e., mCherry^–^/MAP2^+^, hollow yellow arrow of Figure 2D) did not, validating the specificity and efficiency of TAMM labeling. In addition, TAMM labeling did not affect cell morphology, indicating very low toxicity (if any) of the reagent to the cultured neurons.

### Iterative TAMM Condensation for Selective Modification of Three Different Cell Surface Proteins

Encouraged by the labeling efficiency of **Cy5-TAMM-SCH**_**2**_**CF**_**3**_, we wondered if TAMM condensation could be employed iteratively for labeling different targets. Genetic code expansion^24−26^ and sequence-specific proteolytic cleavage can also be used to generate proteins with 1,2-aminothiol functionality.^16^ For example, d-Cys-ε-Lys (**CysK**) can be genetically incorporated into proteins produced by mammalian cells through *Methanosarcina mazei* pyrrolysyl-tRNA synthetase (*Mm*PylRS)/tRNA pair via genetic code expansion.^27^ On the other hand, Factor Xa (FXa) protease and enterokinase (EK)cleave the amide bond after the IDGR or DDDDK sequence, respectively, so placing a cysteine residue immediately following these sequences can afford proteins with an N-terminal cysteine after proteolytic cleavage.

For proof of concept, we first investigated labeling two target proteins with either **CysK** or Factor Xa recognition sequence. To this end, HEK293T cells were transfected to express either **CysK**-HA-Nlgn3 or IDGR-C-HA-Nlgn3 (Figure S27). Cells expressing **CysK**-HA-Nlgn3 were cultured in the presence of its methyl ester (i.e., **CysK-OMe**)^28^ to enhance cell permeability of the unnatural amino acid.^29,30^ After 24 h, the two sets of cells were mixed and subjected to further incubation before treatment with 2 μM **Cy3-TAMM-SCH**_**2**_**CF**_**3**_ to label **CysK**-HA-Nlgn3. We then used **PEG-TAMM-SCH**_**2**_**CF**_**3**_ to quench any unreacted 1,2-aminothiol groups before proteolytic cleavage by Factor Xa protease to expose the 1,2-aminothiol functionality of IDGR-C-HA-Nlgn3. The second target was then labeled with 2 μM **Cy5-TAMM-SCH**_**2**_**CF**_**3**_ before confocal imaging. To our delight, cells were labeled with either Cy3 or Cy5 fluorescence but not both, indicating the feasibility of this approach. For further validation, we applied this approach for labeling two target proteins on the same cells. HEK293T cells were transfected to coexpress two cell surface proteins, **CysK**-HA-CHRM3 (ca. 108 kDa) and IDGR-C-HA-Nlgn3 (ca. 90 kDa). Here, we first labeled CHRM3 with **Cy5-TAMM-SCH**_**2**_**CF**_**3**_ and then Nlgn3 with **Cy3-TAMM-SCH**_**2**_**CF**_**3**_. While confocal microscopy images showed negligible differences between the two labels, in-gel fluorescent analysis confirmed specific labeling of CHRM3 with Cy5 and Nlgn3 with Cy3 (Figure S28).

**Figure 3:**
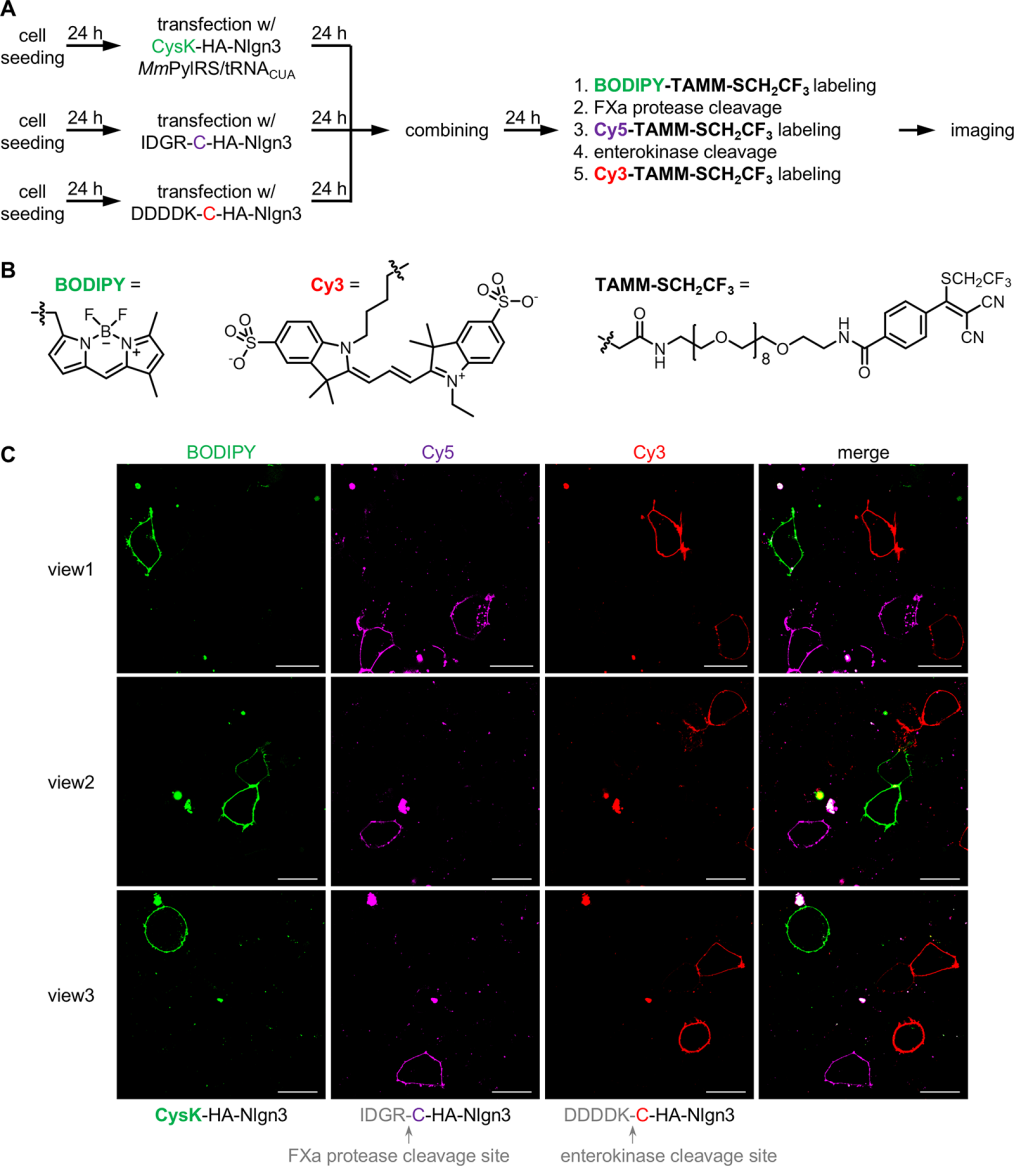
Iterative TAMM condensation for labeling three different target proteins. (A) Schematic presentation of the experimental flow. HEK293T cells overexpressing CysK-HA-Nlgn3 were first labeled with **BODIPY-TAMM-SCH**_**2**_**CF**_**3**_. FXa protease treatment then generated proteins with N-terminal cysteine on cells overexpressing IDGR-C-HA-Nlgn3 for labeling with **Cy5-TAMM-SCH**_**2**_**CF**_**3**_. Lastly, enterokinase treatment removed DDDDK of DDDDK-C-HA-Nlgn3 for labeling with **Cy3-TAMM-SCH**_**2**_**CF**_**3**_. (B) Structure of **BODIPY-TAMM-SCH**_**2**_**CF**_**3**_ and **Cy3-TAMM-SCH**_**2**_**CF**_**3**_. (C) Representative confocal microscopy images. Scale bar = 20 μm.

We then evaluated the feasibility of iterative TAMM condensation for three-color fluorescent labeling. HEK293T cells expressing either **CysK**-HA-Nlgn3, IDGR-C-HA-Nlgn3 or DDDDK-C-HA-Nlgn3 were pooled together and subjected to sequential one-pot labeling (Figure 3). **CysK**-HA-Nlgn3 was first labeled with **BODIPY-TAMM-SCH**_**2**_**CF**_**3**_, followed by Factor Xa treatment and **Cy5-TAMM-SCH**_**2**_**CF**_**3**_ labeling. Finally, treatment with enterokinase converted DDDDK-C-HA-Nlgn3 into C-HA-Nlgn3 for labeling with **Cy3-TAMM-SCH**_**2**_**CF**_**3**_. Confocal microscopy imaging showed specific labeling of different cell populations, demonstrating the feasibility of TAMM condensation for iterative labeling of three different target proteins.

### Combination of TAMM Condensation to CuAAC and Tetrazine Ligation for Multitarget Labeling

**Figure 4:**
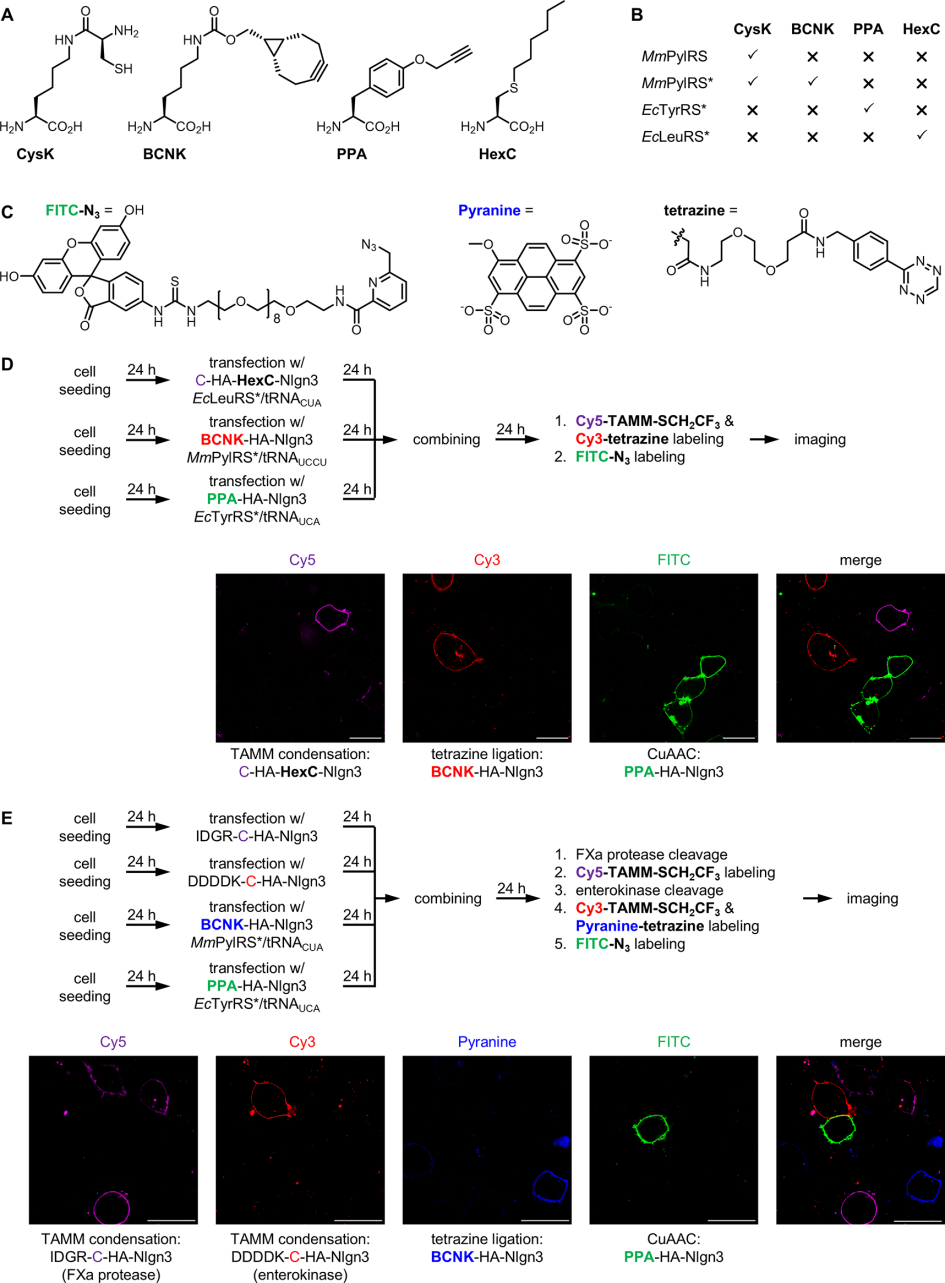
Using TAMM condensation, CuAAC, and tetrazine ligation for multicolor fluorescent labeling. (A) Structure of unnatural amino acids **CysK**, **PPA**, **BCNK**, and **HexC**. (B) Substrate scope of the orthogonal aminoacyl-tRNA synthetases. (C) Structure of **FITC-N**_**3**_ and **Pyranine-tetrazine**. (D) Using three unnatural amino acids to control the bioorthogonal reactions for labeling three target proteins on different HEK293T cells. (E) Using two sequence specific proteases for iterative TAMM condensation in combination with tetrazine ligation and CuAAC for labeling four different target proteins on HEK293T cells. (D,E) Schematic presentation of the experimental flow is shown at the top. Representative confocal microscopy images are shown at the bottom. Two other sets of data are shown in Figures S32 and S33. Scale bar = 20 μm.

Based on the reaction mechanism, we expected TAMM condensation to be compatible with common bioorthogonal reactions, such as CuAAC and tetrazine ligation. To use these bioorthogonal reactions for protein labeling, we employed genetic code expansion to insert unnatural amino acid **PPA** or **BCNK** (Figure 4A) bearing the functional group required for CuAAc or tetrazine ligation, respectively.^25,26^ Incorporation of **PPA** can be achieved by an engineered *Escherichia coli* tyrosyl-tRNA synthetase (*Ec*TyrRS*)/tRNA pair,^31^ whereas **BCNK** by an engineered *M. mazei* pyrrolysyl-tRNA synthetase (*Mm*PylRS*)/tRNA pair.^32^

We first checked the compatibility of TAMM condensation with CuAAC using a pool of HEK293T cells expressing either C-HA-Nlgn3 or **PPA**-HA-Nlgn3 (Figure S29A), subjected to TAMM condensation with **Cy5-TAMM-SCH**_**2**_**CF**_**3**_ and CuAAC with **FITC-N**_**3**_. Confocal microscopy analysis showed cells with either Cy5 or FITC fluorescence but not both, indicating the compatibility and specificity of the two reactions (Figure S29B). Similarly, TAMM condensation could also be used together with tetrazine ligation for dual-color fluorescent labeling (Figure S29C). Here, each labeling step was performed individually for 10 min. While **BCNK** can theoretically react with **FITC-N**_**3**_ via strain-promoted azide–alkyne cycloaddition (SPAAC), this reaction is about 1000 times slower than tetrazine ligation,^14^ and no SPAAC adduct was observed in 10 min (Figure S30). Thus, the three reaction pairs were reckoned as orthogonal under the tested conditions.

We then moved forward to combine these reactions for three-color fluorescent labeling. Here, UAG and UGA codons were used to encode **BCNK** and **PPA**, respectively. HEK293T cells expressed either C-HA-Nlgn3, **BCNK**-HA-Nlgn3 or **PPA**-HA-Nlgn3 were pooled for sequential labeling by TAMM condensation, tetrazine ligation and CuAAC, respectively (Figure S31). Results of confocal microscopy imaging showed all labeled cells with only one fluorescence, confirming the compatibility and specificity of these reactions. For further validation, we transfected HEK293T cells to coexpress three cell surface proteins, C-HA-Trop2 (ca. 36 kDa), **PPA**-HA-Nlgn3 (ca. 90 kDa) and **BCNK**-HA-CHRM3 (ca. 108 kDa). Here, we performed TAMM condensation and tetrazine ligation at the same time, followed by CuAAC. Confocal microscopy showed little difference between the three fluorescence patterns. Nevertheless, in-gel fluorescent analysis confirmed the specific labeling of each target with the designated fluorophore (Figure S32), supporting the orthogonality of these reactions.

Since TAMM condensation alone can label three different proteins through one unnatural amino acid (**CysK**) and two proteolytic cleavages (Figure 4), in theory, combination of iterative TAMM condensation with tetrazine ligation and CuAAC would enable five-color labeling. However, *Mm*PylRS* can use both **BCNK** and **CysK** as its substrate, preventing concurrent use of these two unnatural amino acids. To address this issue, we needed to (i) either identify another unnatural amino acid with 1,2-aminothiol functionality (ii) or use an unnatural amino acid to control production of the N-terminal cysteine bearing target protein. Importantly, the employed unnatural amino acid and the corresponding orthogonal synthetase/tRNA pair must be mutually orthogonal to **BCNK** and **PPA** incorporation. Engineered *Escherichia coli* leucyl-tRNA synthetase (*Ec*LeuRS)/tRNA pair can incorporate various aliphatic unnatural amino acids and is orthogonal to both *Mm*PylRS*/tRNA and *Ec*TyrRS*/tRNA pairs. For example, *S*-hexyl-l-cysteine (**HexC**) can be efficiently incorporated into proteins in mammalian cells by *Ec*LeuRS*/tRNA_CUA_ pair in response to amber codon,^33^ and incorporation of **HexC**, **BCNK** and **PPA** by *Ec*LeuRS*/tRNA_CUA_, *Mm*PylRS*/tRNA_UCCU_ and *Ec*TyrRS*/tRNA_UCA_ in response to UAG, AGGA^34,35^ and UGA codons is mutually orthogonal. We then constructed a plasmid encoding C-HA-TAG-Nlgn3, so that Nlgn3 protein is only produced in the presence of **HexC** and *Ec*LeuRS*/tRNA_CUA_ pair. We then verified the feasibility of using three unnatural amino acids for labeling three different protein targets. HEK293T cells expressing either C-HA-**HexC**-Nlgn3, **BCNK**-HA-Nlgn3 or **PPA**-HA-Nlgn3 were pooled and subjected to simultaneous labeling with **Cy5-TAMM-SCH**_**2**_**CF**_**3**_ and **Cy3-tetrazine**, followed by **FITC-N**_**3**_. Confocal microscopy analysis confirmed the specificity of unnatural amino acid incorporation and the labeling reactions (Figures 4D and S33). On the other hand, combining TAMM condensation via two sequential proteolytic cleavages, tetrazine ligation and CuAAC also successfully afforded specific labeling of four different target proteins (Figures 4E and S34), demonstrating the feasibility of using these three reactions for five-color fluorescent labeling.

### TAMM Condensation and Tetrazine Ligation for In Vivo Dual-Color Labeling

**Figure 5:**
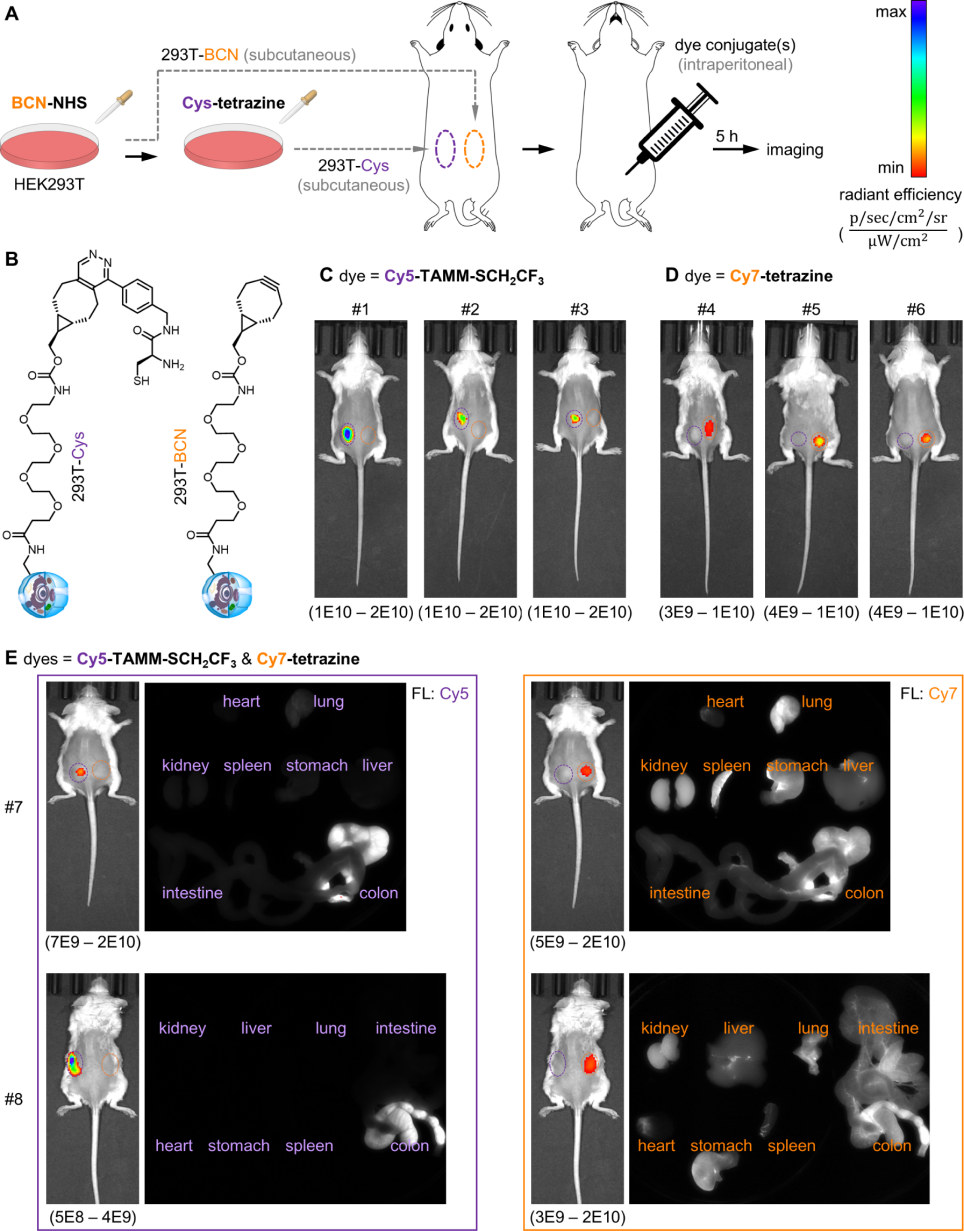
Using TAMM condensation and tetrazine ligation for dual-color labeling in living mice. (A) Schematic presentation of the experimental flow. (B) Structure of the functional groups on 293T-Cys and 293T-BCN. (C) Mice treated with **Cy5-TAMM-SCH**_**2**_**CF**_**3**_. (D) Mice treated with **Cy7-tetrazine**. (E) Cy5 and Cy7 fluorescence of the living mice treated with two dye conjugates and their major organs. Maximum and minimum epi fluorescence values are shown at the top and bottom of the scale bars.

Due to the high efficiency and mild reaction conditions of the fast TAMM condensation, we wondered if it can be used for labeling cells in living mice. Tetrazine ligation was previously shown to enable fluorescent labeling in living mice.^36^ We adapted the protocol (Figure 5A) to subcutaneously graft 1,2-aminothiol functionalized HEK293T cells (i.e., 293T-Cys, Figure 5B) on the left rear limb and bicyclo[6.1.0]non-4-yne (BCN) functionalized HEK293T cells (293T-BCN, Figure 5B) on the right rear limb of immunodeficient mice. Mice were then subjected to intraperitoneal injection with either **Cy5-TAMM-SCH**_**2**_**CF**_**3**_ (Figure 5C) or **Cy7-tetrazine** (Figure 5D). About 5 h postinjection, we observed stronger fluorescence signals on the target cells. Specifically, mice injected with **Cy5-TAMM-SCH**_**2**_**CF**_**3**_ showed stronger Cy5 fluorescence only at the left (i.e., purple circles, sites of 293T-Cys) but not at the right (i.e., orange circles, sites of 293T-BCN) (Figure 5C). In contrast, mice injected with **Cy7-tetrazine** showed stronger Cy7 fluorescence only at the right but not at the left (Figure 5D), confirming the specificity of the labeling. When both **Cy5-TAMM-SCH**_**2**_**CF**_**3**_ and **Cy7-tetrazine** were injected at the same time (Figure 5E), we observed clear labeling of cells by the corresponding fluorophore conjugate, demonstrating not only the feasibility of TAMM condensation in living mice but also its use for in vivo dual-color labeling. It is also noteworthy that fluorescence of the tetrazine conjugate was observed in all major organs, whereas fluorescence of the TAMM conjugate was only observed in the colon, indicating faster metabolism of the TAMM conjugate than that of tetrazine ligation.

## Discussion

Attaching a dye specifically to the target protein is the prerequisite for fluorescence imaging using small molecules. Biologists also use antibody-dye conjugates for immunofluorescence imaging. However, this strategy is more suitable for fixed cell samples due to the harsh washing conditions. In addition, the large size of antibodies (ca. 150 kDa) can affect the outcome of staining. For example, we found immunofluorescence failed to stain some target proteins in cell–cell junctions that could be labeled by TAMM conjugates (Figure 2C). Others also observed that some cell-surface proteins on live neurons failed to be visualized by immunofluorescence but could be labeled through bioorthogonal chemistry,^37^ highlighting the advantage of small-molecule dyes in fluorescence imaging.

For multicolor imaging, each target necessitates a designated bioorthogonal reaction and means to introduce the required bioorthogonal functionality. Here, we demonstrated iterative TAMM condensation for labeling three different targets, overcoming the limitation of one reaction per target. A similar concept in tetrazine ligation has recently been reported by the Tao Liu group, who put a tetrazine amino acid into two target proteins and used the host–guest recognition of tetrazine and naphthotubes to mask and unmask the second tetrazine functionality.^36^ However, this concept is limited to an extracellular and a cytosolic target, whereas TAMM condensation can label three different extracellular targets. In addition, iterative TAMM condensation can theoretically be expanded further through the inclusion of other proteases (e.g., TEV protease),^16^ enabling four-color labeling. This would suit most confocal microscopy experiments as most equipment only has four lasers.

On the other hand, TAMM condensation is also compatible with CuAAC and tetrazine ligation (Figure 4). By combing these three bioorthogonal reactions, here we demonstrated labeling of three or four targets, but five targets can also be readily achievable with suitable equipment. Thus, bioconjugation chemistry is no longer the limiting factor for fluorescent imaging of cell-surface proteins by small-molecule fluorophores. It is noteworthy that while bioorthogonal chemistry-based triple labeling has been demonstrated in purified proteins and cell lysates,^38−40^ our work presents the first example of labeling three or four different target proteins on living cells.

While TAMM condensation has shown great in vitro applications, there are some limitations. Paraformaldehyde, widely used for cell fixation, is also highly reactive to 1,2-aminothiol^41,42^ so TAMM condensation has to be performed prior to cell fixation. On the other hand, when the abundance of the target protein is low (e.g., Figures 2D and 3C) or using high laser power or high gain, such as Cy5 (high) channel in Figure 2C, we noticed the presence of additional puncta. In many instances, the puncta resulted from different TAMM conjugates colocalize (Figure 3C), suggesting nonspecific labeling of cell debris. This seems to be a general phenomenon but much less pronounced when the labeling signal is strong, such as the Cy5 (low) channel in Figure 2C.

In addition to cell lines and primary neurons, TAMM condensation is also applicable for in vivo labeling and can be combined with tetrazine ligation for dual-color labeling in living mice (Figure 5). In contrast to in vitro labeling, where thiol-free culture media can be used to avoid unwanted thio exchange and unreacted fluorophores can be easily washed out, these operations are not feasible for in vivo labeling. The presence of low micromolar of glutathione in plasma^43^ may slow the reaction kinetics of fast TAMM molecules. Additionally, the unreacted fluorophores present in our experiments have equally strong fluorescence as their counterparts labeled to the target proteins. Nevertheless, the unreacted fluorophores can be excreted by the body, whereas the labeled fluorophores remain associated with the target. In comparison to tetrazine conjugates, TAMM conjugates showed faster metabolism in mice, providing higher signal-to-noise ratio than that of tetrazine ligation. Faster metabolism can be an advantageous character for certain in vivo applications, such as small-molecule triggered drug activation, of which tetrazine ligation has been used for proof-of-concept research.^44^

The aforementioned breakthrough in TAMM condensation benefited from our mechanistic investigation, combining techniques such as HPLC, LC-MS, NMR, chemical trapping, and computational analyses. The stability of TAMM reagents in aqueous environments was demonstrated by their long lifetimes, exceeding a hundred hours in most cases. Our study unveiled the identities of two intermediates. Although the formation of the intermediates delays the product formation as evidenced by similar product accumulation profiles for reactions of **2x** with **1a**, **1i**, or **1j** (Figure S12), the transformation of the intermediates into dihydrothiazole product was not interfered with by other aminothiols. In addition, cell surface protein labeling efficiency by different TAMM conjugates aligns with their consumption rate difference. Thus, the rate of starting material consumption was regarded as most relevant for biological applications of TAMM labeling. Our quantitative analysis of reaction kinetics not only sheds light on the reaction pathway but also highlights crucial structural features in TAMM for accelerated reactions. While electron-withdrawing substituents on the aryl group enhance reaction rates, the incorporation of a low-p*K*_a_ thiol leaving group (R^3^) in TAMM yields even more substantial rate enhancements. Our investigation suggests that 2,2,2-trifluoroethanethiol optimally balances the chemical stability (likely due to local lipophilicity) and p*K*_a_ (sufficiently low to facilitate initial thiolate exchange yet nucleophilic enough to convert **Int-2** into the dihydrothiazole product). We were able to enhance the labeling rate from around 10 M^–1^ s^–1^ to 10^4^ M^–1^ s^–1^, about 3 orders of magnitude improvement. The fast TAMM condensation belongs to one of the fastest bioorthogonal reactions reported to date.

## Conclusion

Mechanistic study into TAMM condensation provided a comprehensive understanding of the intricacy underlying this rapid and reliable multistep bioconjugation reaction. Through structural modifications on TAMM reagents and the use of genetic code expansion and sequence-specific proteolytic cleavage for protein substrates, the reactivity and applicability of this reaction were significantly enhanced. Furthermore, this reaction was found to be compatible with common bioorthogonal reactions, such as CuAAC and tetrazine ligation, enabling more multicolor fluorescent cell labeling. Thus, TAMM condensation represents a valuable reaction with wide-ranging applicability for site-specific protein modification from in vitro to in vivo.

## Supporting Information Available

Experimental procedure for chemical syntheses of **1a**–**1n** and dye conjugates, kinetic evaluation, reaction order determination, estimation of the thiol group p*K*_a_ in peptide **2x**, activation parameters of the reaction between **1a** and **2x**, rate constants and activation parameters for the conversion of **Int-1** to **3**, cell culture conditions and transient transfection, cell viability assay, protein fluorescent labeling, confocal imaging, in-gel fluorescent analysis, immunoblotting, and fluorescent labeling in living mice; energies and Cartesian coordinates for optimized geometries; Python script for numerical simulation; DNA and amino acid sequences of the constructs used in this study; Supplementary Figures S1–S34; Table S1–S3; ^1^H and ^13^C NMR spectra of the synthesized compounds (PDF)
