# Supplementary material for: A Single Bioorthogonal Reaction for Multiplex Cell Surface Protein Labeling
Source: J Am Chem Soc. 2025 Jan 3;147(2):1612–23. doi: 10.1021/jacs.4c11701 (PMC11744750; doi:10.1021/jacs.4c11701)
Supplement: Supplementary file 1 — ja4c11701_si_001.pdf [file ja4c11701_si_001.pdf]

Supporting Information

A single bioorthogonal reaction for multiplex cell surface protein labeling

Yang Huang,[1,2,†] Chengyang Wu,[3,†] Anjing Lu,[2,†] Jingzhe Wang,[2,4] Jian Liang,[5,6] Han Sun,[2] Liqing Yang,[2] Shixiang Duan,[2] Andrey A. Berezin,[3] Chuanliu Wu,[7] Bo Zhang,[5,6] Yi-Lin Wu,[3,*] Yu-Hsuan Tsai[2,*]

[1]School of Basic Medical Sciences, Capital Medical University, Beijing, 100069, China

[2]Institute of Molecular Physiology, Shenzhen Bay Laboratory, Shenzhen, 518132, China

[3]School of Chemistry, Cardiff University, Cardiff, CF10 3AT, United Kingdom

[4]College of Chemistry and Pharmacy, Northwest A&F University, Yangling 712100, China.

[5]Institute of Neurological and Psychiatric Disorders, Shenzhen Bay Laboratory, Shenzhen, 518107, China

[6]School of Chemical Biology and Biotechnology, Peking University Shenzhen Graduate School, Shenzhen, 518055, China.

[7]Department of Chemistry, College of Chemistry and Chemical Engineering, The MOE Key Laboratory of Spectrochemical Analysis and Instrumentation, State Key Laboratory of Physical Chemistry of Solid Surfaces, Xiamen University, Xiamen, 361005, China

†These authors contributed equally to this work.

*Correspondence authors: WuYL@cardiff.ac.uk; tsai.y-h@outlook.com

Table of Contents

Materials and Methods 5
Synthesis of TAMM **1a** 5
Synthesis of TAMM **1b** 6
Synthesis of TAMM **1c** 7
Synthesis of TAMM **1d** 8
Synthesis of TAMM **1e** 8
Synthesis of TAMM **1f** 9
Synthesis of TAMM **1g** 10
Synthesis of TAMM **1h** 11
Synthesis of TAMM **1i** 11
Synthesis of TAMM **1j** 12
Synthesis of TAMM **1k** 12
Synthesis of TAMM **1l** 13
Synthesis of TAMM **1m** 13
Synthesis of TAMM **1n** 14
Synthesis of **Cy5-TAMM-SCH$_2$CF$_3$** 14
Synthesis of **Cy3-TAMM-SCH$_2$CF$_3$** 15
Synthesis of **Cy5-TAMM-SEt** 16
Synthesis of **Cy5-TAMM-SPhF** 17
Synthesis of **Cy3-tetrazine** 19
Synthesis of **Cy7-tetrazine** 20
Synthesis of **FITC-N$_3$** 20
Synthesis of **Pyranine-tetrazine** 21
Synthesis of **BCN-NHS** 22
Synthesis of **Tetrazine-cysteine** 23
Kinetic studies 24
The order of the reaction and the observed rate constant 25
Estimation of the pKa of the thiol group in peptide **2x** 26
Activation parameters of the reaction between **1a** and **2x** 27
Rate constants and activation parameters for the conversion of **Int-1** (**7**) to **3** 27
Culture and transfection of cell lines 28
Culture and transfection of primary neurons 28
Cell viability assay 29
Protein fluorescent labeling 29
Confocal imaging 30
In-gel fluorescent analysis 30
Immunoblotting 30
Fluorescent labeling in living mice 31
Supplementary Text 32
Energies and Cartesian coordinates for optimized geometries 32
Python script for numerical simulation 56
Sequences of the constructs used in this manuscript 59
C-HA-Nlgn3 59
C-HA-Nlgn3-mCherry 60
TAG-HA-Nlgn3 62

TGA-HA-Nlgn3 63
AGGA-HA-Nlgn3 65
IDGR-C-HA-Nlgn3 66
DDDDK-C-HA-Nlgn3 68
TAG-HA-CHRM3 69
C-HA-Trop2 71
BFP-P2A-C-HA-EGFR 72
*Mm*PylRS 74
*Mm*PylRS* 75
Pyl-$tRNA_{CUA}$ 76
Pyl-$tRNA_{UCCU}$ 76
*Ec*TyrRS* 76
Tyr-$tRNA_{UCA}$ 77
*Ec*LeuRS* 77
Leu-$tRNA_{CUA}$ 79
Supplementary Figures 80
Fig. S1. Determination of the reaction order. 80
Fig. S2. Eyring (a) and Arrhenius (b) plots for the reaction of **1a** and **2x** at pH 7.4. 81
Fig. S3. Effect of TAMM *para* substitution (R^1) on reaction kinetics. 82
Fig. S4. LC-MS results of reactions involving intermediates. 83
Fig. S5. Calculated free energy of **5'–7'**. 84
Fig. S6. Characterization of the intermediate in TAMM condensation. 85
Fig. S7. Structural characterization of **8bz**. 86
Fig. S8. Structural characterization of **8ez**. 87
Fig. S9. ^{1}H NMR spectra of **8bz** (a) and **8ez** (b) in DMSO-d_6 at 25, 50 or 75 °C. 88
Fig. S10. Characterization of N-linked dicyanovinyl molecules. 89
Fig. S11. Transformation of **Int-1** to **3** unaffected by other 1,2-aminothiol molecules. 90
Fig. S12. Kinetic parameters. 91
Fig. S13. Conversion of **Int-1** to dihydrothiazole **3**. 92
Fig. S14. Standard curve of **p-KL42** (a), **3ax** (b), **3bx** (c) and 3**bex** (d). 93
Fig. S15. Rate constant determination. 94
Fig. S16. Reaction of TAMM **1j** and peptide **2x** to form dihydrothiazole **3ax**. 95
Fig. S17. Characterization of **10bz** from reaction of **1l** and **2z**. 96
Fig. S18. Characterization of **10ez** from reaction of **1k** and **2z**. 97
Fig. S19. Aqueous stability of TAMM molecules. 98
Fig. S20. Effects of malononitrile and **PEG-TAMM-SCH_2CF_3** on cell viability. 99
Fig. S21. Effect of leaving groups in TAMM conjugates for fluorescent labeling. 100
Fig. S22. Representative images of labeling C-HA-Nlgn3-mCherry. 101
Fig. S23. Representative images of labeling in HeLa cells. 102
Fig. S24. Representative images of labeling in MCF7 cells. 103
Fig. S25. Representative images of labeling in ND7/23 cells. 104
Fig. S26. Representative images of labeling in SK-OV-3 cells. 105
Fig. S27. Dual TAMM condensation for labeling two populations of cells. 106
Fig. S28. Dual TAMM condensation for labeling two targets on the same cells. 107
Fig. S29. Compatibility of TAMM condensation to CuAAC and tetrazine ligation 108
Fig. S30. Verification of the orthogonality of **BCNK** and **FITC-N_3**. 109

Fig. S31. Labeling three populations of cells. 110
Fig. S32. Labeling three targets on the same cells. 111
Fig. S33. Using three unnatural amino acids to control the bioorthogonal reactions. 112
Fig. S34. Four-color fluorescence labeling. 113
Supplementary Tables 114
Table S1. Initial rates of **1a** and **2x** 114
Table S2. Rate constants at different temperatures. 114
Table S3. Rate constants for **Int-1** to **3** 114
NMR Spectra 115
Synthesis of **1a** 115
Synthesis of **1b** 118
Synthesis of **1c** 120
Synthesis of **1d** 123
Synthesis of **1e** 124
Synthesis of **1f** 126
Synthesis of **1g** 128
Synthesis of **1h** 130
Synthesis of **1i** 131
Synthesis of **1j** 133
Synthesis of **1k** 134
Synthesis of **1l** 136
Synthesis of **1m** 138
Synthesis of **1n** 139
Synthesis of **Cy5-TAMM-SCH$_2$CF$_3$** 140
Synthesis of **Cy3-TAMM-SCH$_2$CF$_3$** 143
Synthesis of **Cy5-TAMM-SEt** 144
Synthesis of **Cy5-TAMM-SPhF** 147
Synthesis of **Cy3-tetrazine** 150
Synthesis of **Cy7-tetrazine** 152
Synthesis of **FITC-N$_3$** 153
Synthesis of **Pyranine-tetrazine** 155
Synthesis of **BCN-NHS** 158
Synthesis of **Tetrazine-cysteine** 161

Materials and Methods

Synthesis of TAMM **1a**

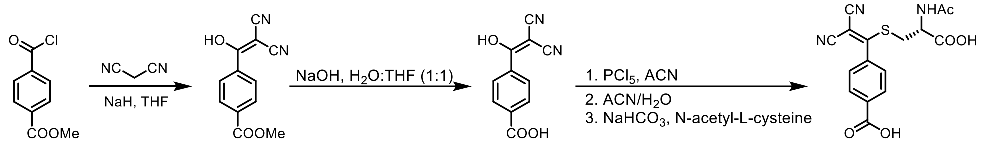

TAMM 1a

To a suspension of NaH (144 mg, 6.0 mmol, 2.0 equiv.) in anhydrous THF (3 mL) under argon atmosphere at 0 °C was added a solution of malononitrile (200 mg, 3.0 mmol, 1.0 equiv.) in anhydrous THF (2 mL) dropwise. After the addition, the reaction was stirred for an hour at 0 °C. A solution of methyl 4-chloroformyl benzoate (600 mg, 3.0 mmol, 1.0 equiv.) in anhydrous THF (3 mL) was added into the mixture dropwise at 0 °C. The reaction mixture was then allowed to warm to room temperature and stirred at room temperature for another one hour. The solvent was then removed under reduced pressure. Ice water (10 mL) was added slowly, and the mixture was acidified to pH 1–2 using 1N $HCl_{(aq)}$ and extracted with EtOAc (3 × 10 mL). The combined organic phases were washed with brine (3 × 10 mL), dried over Na_2SO_4, filtered, and concentrated. The residue was purified by flash chromatography (SiO_2, 10% MeOH in DCM) to yield a light-yellow solid (525 mg, 2.3 mmol, 75%). ^{1}H NMR (400 MHz, CD_3OD) δ 8.04 (d, *J* = 8.0 Hz, 2H), 7.74 (d, *J* = 8.0 Hz, 2H), 3.91 (s, 3H). ^{13}C NMR (101 MHz, CD_3OD) δ 190.7, 167.8, 144.2, 133.0, 130.2, 128.7, 121.5, 120.4, 52.8, 49.9. ESI-HRMS (m/z): calculated for $C_{12}H_7N_2O_3$ $[M-H]^-$: 227.0462; found, 227.0456.

To a solution of methyl 4-(2,2-dicyano-1-hydroxyvinyl)benzoate (456 mg, 2 mmol, 1.0 equiv.) in THF (10 mL) was added a solution of NaOH (320 mg, 8.0 mmol, 4.0 equiv.) in water (10 mL). After three hours at the room temperature, complete conversion was confirmed by TLC. THF was then removed under reduced pressure. Water (20 mL) was added, and the aqueous layer was washed with EtOAc (3 ×10 mL). Then the mixture was acidified to pH 1–2 using 1 N $HCl_{(aq)}$. The mixture was extracted with EtOAc (3 ×20 mL). The combined organic phases were washed with brine (20 mL), dried over Na_2SO_4, filtered, and concentrated to yield 4-(2,2-dicyano-1-hydroxyvinyl)benzoic acid as a light yellow solid (347 mg, 1.8 mmol, 90%). ^{1}H NMR (400 MHz, CD_3OD) δ 8.05 (d, *J* = 8.0 Hz, 1H), 7.72 (d, *J* = 8.4 Hz, 1H). ^{13}C NMR (101 MHz, CD_3OD) δ 191.0, 169.1, 144.2, 133.6, 130.5, 128.6, 121.8, 120.0, 52.6. ESI-HRMS (m/z): calculated for $C_{11}H_5N_2O_3$ $[M-H]^-$: 213. 0306; found, 213.0299.

To a solution of 4-(2,2-dicyano-1-hydroxyvinyl)benzoic acid (214 mg, 1.0 mmol, 1.0 equiv.) in acetonitrile (30 mL) was added PCl_5 (624 mg, 3.0 mmol, 3.0 equiv.). After five hours at 50 °C under argon atmosphere, the mixture was concentrated. The residue was dissolved in DCM (60 mL), washed with water (3 ×20 mL) and brine (20 mL), dried over Na_2SO_4, filtered, and concentrated. The resulting yellow solid was then dissolved in acetonitrile (5 mL) and water (5 mL). After 30 minutes, N-acetyl-L-cysteine (107 mg, 1.5 mmol, 1.5 equiv.) and $NaHCO_3$ (252 mg, 3.0 mmol, 3.0 equiv.) were added. The reaction was stirred at room temperature for two hours before acidification to pH 4–5 using 1 N $HCl_{(aq)}$. The acetonitrile was removed under reduced pressure. Brine (15 mL) was added. The mixture was extracted with EtOAc (3 × 20 mL). The combined organic phases were washed with brine (20 mL), dried over Na_2SO_4, filtered, and concentrated. The residue was purified by flash chromatography (SiO_2, 10% MeOH in EtOAc) to yield TAMM **1a** as a yellow powder (125 mg, 0.5 mmol, 50%).^{1}H NMR (400 MHz, CD_3OD) δ 8.22 (d, *J* = 8.4 Hz, 2H), 7.65 (d, *J* = 8.4 Hz, 2H), 4.43 (dd, *J* = 8.4, 4.8 Hz, 1H), 3.24 – 3.03 (m, 2H), 2.00 (s, 3H). ^{13}C NMR (101 MHz, CD_3OD) δ 181.2, 173.3, 171.8,

168.3, 137.7, 135.4, 131.7, 130.2, 113.5, 113.0, 82.2, 52.8, 36.9, 22.4. ESI-HRMS (m/z): calculated for $C_{16}H_{12}N_3O_5S$ $[M-H]^-$: 358.0503; found, 358.0495.

Synthesis of TAMM **1b**

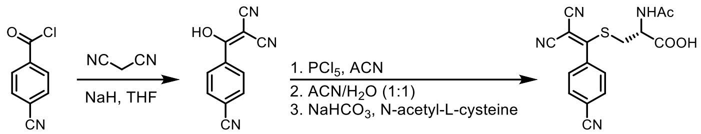

TAMM **1b**

To a suspension of NaH (144 mg, 6.0 mmol, 2.0 equiv.) in anhydrous THF (3 mL) under argon atmosphere at 0 °C was added a solution of malononitrile (200 mg, 3.0 mmol, 1.0 equiv.) in anhydrous THF (2 mL) dropwise. After the addition, the reaction was stirred for one hours at 0 °C. A solution of 4-cyanobenzoyl chloride (496 mg, 3.0 mmol, 1.0 equiv.) in anhydrous THF (3 mL) was added into the mixture dropwise at 0 °C. The reaction mixture was then allowed to warm to room temperature and stirred at room temperature for another hour. The solvent was then removed under reduced pressure. Ice water (10 mL) was added slowly, and the mixture was acidified to pH 1-2 using HCl(aq) and extracted with EtOAc (3 × 10 mL). The combined organic phases were washed with brine (3 × 10 mL), dried over Na_2SO_4, filtered, and concentrated. The residue was purified by flash chromatography (SiO_2, 10% MeOH in DCM) to yield a light-yellow solid (410 mg, 2.1 mmol, 70%). ^{1}H NMR (600 MHz, CD_3OD) δ 7.91 (d, *J* = 7.8 Hz, 2H), 7.85 (d, *J* = 7.8 Hz, 2H). ^{13}C NMR (151 MHz, CD_3OD) δ 190.3, 145.1, 133.1, 129.4, 122.1, 120.3, 119.3, 114.7, 51.8. ESI-HRMS (m/z): calculated for $C_{11}H_4N_3O$ $[M-H]^-$: 194.0360; found, 194.0356.

To a solution of 2-((4-cyanophenyl)(hydroxy)methylene)malononitrile (195 mg, 1.0 mmol, 1.0 equiv.) in acetonitrile (30 mL) was added PCl_5 (624 mg, 3.0 mmol, 3.0 equiv.). After five hours at 50 °C under argon atmosphere, the mixture was concentrated. The residue was dissolved in DCM (60 mL), washed with water (3 × 20 mL) and brine (20 mL), dried over Na_2SO_4, filtered, and concentrated. The resulting yellow solid was then dissolved in acetonitrile (5 mL) and water (5 mL). After 30 min, N-acetyl-L-cysteine (107 mg, 1.5 mmol, 1.5 equiv.) and $NaHCO_3$ (252 mg, 3.0 mmol, 3.0 equiv.) were added. The reaction was stirred at room temperature for 2 hours before acidification to pH 4–5 using 1 N $HCl_{(aq)}$. The acetonitrile was removed under reduced pressure. Brine (15 mL) was added. The mixture was extracted with EtOAc (3 × 20 mL). The combined organic phases were washed with brine (20 mL), dried over Na_2SO_4, filtered, and concentrated. The residue was purified by flash chromatography (SiO_2, 10% MeOH in DCM) to yield TAMM **1b** as a yellow powder (91 mg, 0.27 mmol, 27%). ^{1}H NMR (300 MHz, CD_3OD) δ 7.99 (d, *J* = 8.7 Hz, 2H), 7.82 – 7.67 (m, 2H), 4.40 (dd, *J* = 8.3, 4.7 Hz, 1H), 3.24 – 3.00 (m, 2H), 2.00 (s, 3H). ^{13}C NMR (101 MHz, CD_3OD) δ 179.9, 173.3, 171.7, 138.0, 134.4, 131.0, 118.6, 116.9, 113.3, 112.8, 82.9, 52.8, 36.9, 22.4. ESI-HRMS (m/z): calculated for $C_{16}H_{13}N_4O_3S$ $[M+H]^+$: 341.0703; found, 341.0708.

Synthesis of TAMM **1c**

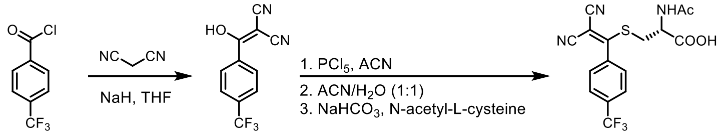

TAMM 1c

To a suspension of NaH (144 mg, 6.0 mmol, 2.0 equiv.) in anhydrous THF (3 mL) under argon atmosphere at 0 °C was added a solution of malononitrile (200 mg, 3.0 mmol, 1.0 equiv.) in anhydrous THF (2 mL) dropwise. After the addition, the reaction was stirred for 1 hour at 0 °C. A solution of 4-(trifluoromethyl)benzoyl chloride (621 mg, 3.0 mmol, 1.0 equiv.) in anhydrous THF (3 mL) was added into the mixture dropwise at 0 °C. The reaction mixture was then allowed to warm to room temperature and stirred at room temperature for another one hour. The solvent was then removed under reduced pressure. Ice water (10 mL) was added slowly, and the mixture was acidified to pH 1–2 using 1 N $HCl_{(aq)}$ and extracted with EtOAc (3 × 10 mL). The combined organic phases were washed with brine (3 × 10 mL), dried over Na_2SO_4, filtered, and concentrated. The residue was purified by flash chromatography (SiO_2, 10% MeOH in DCM) to yield a light-yellow solid (476 mg, 2.0 mmol, 65%). ^{1}H NMR (400 MHz, CD_3OD) δ 7.88 (s, 4H). ^{13}C NMR (101 MHz, CD_3OD) δ 184.9, 137.4, 134.9 (q, *J* = 32.9 Hz), 130.1, 126.9 (q, *J* = 3.8 Hz), 125.0 (q, *J* = 288.7 Hz), 115.8, 113.9, 63.4. ^{19}F NMR (376 MHz, CD_3OD) δ -64.67 (s, 3F). ESI-HRMS (m/z): calculated for $C_{11}H_4F_3N_2O$ $[M-H]^-$: 237.0281; found, 237.0276.

To a solution of 2-(hydroxy(4-(trifluoromethyl)phenyl)methylene)malononitrile (238 mg, 1.0 mmol, 1.0 equiv.) in acetonitrile (30 mL) was added PCl_5 (624 mg, 3.0 mmol, 3.0 equiv.). After five hours at 50 °C under argon atmosphere, the mixture was concentrated. The residue was dissolved in DCM (60 mL), washed with water (3 × 20 mL) and brine (20 mL), dried over Na_2SO_4, filtered, and concentrated. The resulting yellow solid was then dissolved in acetonitrile (5 mL) and water (5 mL). After 30 minutes, N-acetyl-L-cysteine (107 mg, 1.5 mmol, 1.5 equiv.) and $NaHCO_3$ (252 mg, 3.0 mmol, 3.0 equiv.) were added. The reaction was stirred at room temperature for two hours before acidification to pH 4–5 using 1 N $HCl_{(aq)}$. The acetonitrile was removed under reduced pressure. Brine (15 mL) was added. The mixture was extracted with EtOAc (3 × 20 mL). The combined organic phases were washed with brine (20 mL), dried over Na_2SO_4, filtered, and concentrated. The residue was purified by flash chromatography (SiO_2, 10% MeOH in DCM) to yield TAMM **1c** as a yellow powder (115 mg, 0.3 mmol, 30%). ^{1}H NMR (300 MHz, CD_3OD) δ 7.95 – 7.92 (m, 2H), 7.79 – 7.74 (m, 2H), 4.42 (dd, *J* = 8.4, 4.6 Hz, 1H), 3.26 – 2.95 (m, 2H), 2.00 (s, 3H). ^{13}C NMR (101 MHz, CD_3OD) δ 180.4, 173.3, 171.8, 137.5, 134.7 (q, *J* = 33.1 Hz), 130.9, 127.6 (q, *J* = 3.8 Hz), 126.3, 123.6, 82.8, 52.8, 36.9, 22.4. ^{19}F NMR (376 MHz, CD_3OD) δ -64.62 (s, 3F). ESI-HRMS (m/z): calculated for $C_{16}H_{13}F_3N_3O_3S$ $[M+H]^+$: 384.0624; found, 384.0630.

Synthesis of TAMM **1d**

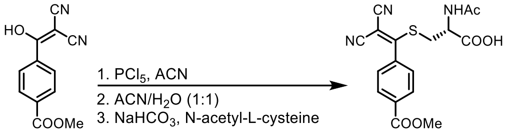

TAMM 1d

To a solution of methyl 4-(2,2-dicyano-1-hydroxyvinyl)benzoate (228 mg, 1.0 mmol, 1.0 equiv., see synthesis of TAMM **1a** for its preparation) in acetonitrile (30 mL) was added PCl_5 (624 mg, 3.0 mmol, 3.0 equiv.). After five hours at 50 °C under argon atmosphere, the mixture was concentrated. The residue was dissolved in DCM (60 mL), washed with water (3 × 20 mL) and brine (20 mL), dried over Na_2SO_4, filtered, and concentrated. The resulting yellow solid was then dissolved in acetonitrile (5 mL) and water (5 mL). After 30 min, N-acetyl-L-cysteine (107 mg, 1.5 mmol, 1.5 equiv.) and $NaHCO_3$ (252 mg, 3.0 mmol, 3.0 equiv.) were added. The reaction was stirred at room temperature for 2 hours before acidification to pH 4–5 using 1 N $HCl_{(aq)}$. The acetonitrile was removed under reduced pressure. Brine (15 mL) was added. The mixture was extracted with EtOAc (3 × 20 mL). The combined organic phases were washed with brine (20 mL), dried over Na_2SO_4, filtered, and concentrated. The residue was purified by flash chromatography (SiO_2, 10% MeOH in DCM) to yield TAMM **1d** as a yellow powder (149 mg, 0.4 mmol, 40%). ^{1}H NMR (300 MHz, CD_3OD) δ 8.23 (d, J = 8.7 Hz, 2H), 7.67 (d, J = 8.7 Hz, 2H), 4.45 – 4.37 (m, 1H), 3.96 (s, 3H), 3.25 – 2.99 (m, 2H), 2.00 (s, 3H). ^{13}C NMR (101 MHz, CD_3OD) δ 181.1, 173.3, 171.8, 167.2, 137.9, 134.7, 131.5, 130.3, 113.4, 113.0, 82.3, 53.1, 52.9, 36.9, 22.4. ESI-HRMS (m/z): calculated for $C_{17}H_{16}N_3O_5S$ $[M+H]^+$: 374.0805; found, 374.0810.

Synthesis of TAMM **1e**

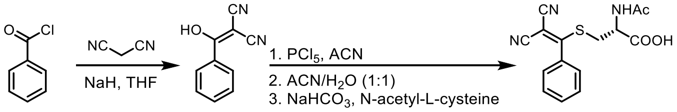

TAMM 1e

To a suspension of NaH (144 mg, 6.0 mmol, 2.0 equiv.) in anhydrous THF (3 mL) under argon atmosphere at 0 °C was added a solution of malononitrile (200 mg, 3.0 mmol, 1.0 equiv.) in anhydrous THF (2 mL) dropwise. After the addition, the reaction was stirred for an hour at 0 °C. A solution of benzoyl chloride (420 mg, 3.0 mmol, 1.0 equiv.) in anhydrous THF (3 mL) was added into the mixture dropwise at 0 °C. The reaction mixture was then allowed to warm to room temperature and stirred at room temperature for another one hour. The solvent was then removed under reduced pressure. Ice water (10 mL) was added slowly, and the mixture was acidified to pH 1–2 using 1 N $HCl_{(aq)}$ and extracted with EtOAc (3 × 10 mL). The combined organic phases were washed with brine (3 × 10 mL), dried over Na_2SO_4, filtered, and concentrated. The residue was purified by flash chromatography (SiO_2, 10% MeOH in DCM) to yield a light-yellow solid (408 mg, 2.4 mmol, 80%).

^{1}H NMR (400 MHz, CD_3OD) δ 6.08 (d, J = 6.8 Hz, 2H), 5.90 – 5.79 (m, 3H). ^{13}C NMR (101 MHz, CD_3OD) δ 191.1, 138.6, 130.4, 127.7, 127.2, 120.6, 119.4, 50.8. ESI-HRMS (m/z): calculated for $C_{10}H_5N_2O$ $[M-H]^-$: 169.0407; found, 169.0392.

To a solution of 2-(hydroxy(phenyl)methylene)malononitrile (170 mg, 1.0 mmol, 1.0 equiv.) in acetonitrile (30 mL) was added PCl_5 (624 mg, 3.0 mmol, 3.0 equiv.). After five hours at 50 °C under argon atmosphere, the mixture was concentrated. The residue was dissolved in DCM (60 mL), washed with water (3 × 20 mL) and brine (20 mL), dried over Na_2SO_4, filtered, and concentrated. The resulting yellow solid was then dissolved in acetonitrile (5 mL) and water (5 mL). After 30 min, N-acetyl-L-cysteine (107 mg, 1.5 mmol, 1.5 equiv.) and $NaHCO_3$ (252

mg, 3.0 mmol, 3.0 equiv.) were added. The reaction was stirred at room temperature for two hours before acidification to pH 4–5 using 1 N $HCl_{(aq)}$. The acetonitrile was removed under reduced pressure. Brine (15 mL) was added. The mixture was extracted with EtOAc (3 × 20 mL). The combined organic phases were washed with brine (20 mL), dried over Na_2SO_4, filtered, and concentrated. The residue was purified by flash chromatography (SiO_2, 10% MeOH in DCM) to yield TAMM **1e** as a yellow powder (110 mg, 0.35 mmol, 35%). ^{1}H NMR (300 MHz, CD_3OD) δ 7.66 – 7.53 (m, 5H), 4.42 (dd, *J* = 8.5, 4.6 Hz, 1H), 3.28 – 2.91 (m, 2H), 2.00 (s, 3H). ^{13}C NMR (101 MHz, CD_3OD) δ 182.4, 173.3, 171.9, 133.8, 133.5, 130.6, 130.0, 113.8, 113.3, 81.4, 52.8, 37.0, 22.4. ESI-HRMS (m/z): calculated for $C_{15}H_{12}N_3O_3S$ [M-H]$^-$: 314.0605; found, 314.0602.

Synthesis of TAMM **1f**

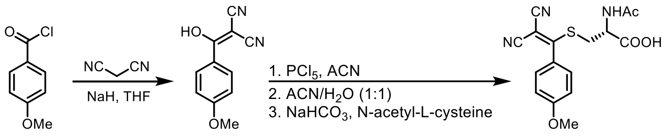

To a suspension of NaH (144 mg, 6.0 mmol, 2.0 equiv.) in anhydrous THF (3 mL) under argon atmosphere at 0 °C was added a solution of malononitrile (200 mg, 3.0 mmol, 1.0 equiv.) in anhydrous THF (2 mL) dropwise. After the addition, the reaction was stirred for one hour at 0 °C. A solution of 4-methoxybenzoyl chloride (540 mg, 3.0 mmol, 1.0 equiv.) in anhydrous THF (3 mL) was added into the mixture dropwise at 0 °C. The reaction mixture was then allowed to warm to room temperature and stirred at room temperature for another one hour. The solvent was then removed under reduced pressure. Ice water (10 mL) was added slowly, and the mixture was acidified to pH 1–2 using 1 N $HCl_{(aq)}$ and extracted with EtOAc (3 × 10 mL). The combined organic phases were washed with brine (3 × 10 mL), dried over Na_2SO_4, filtered, and concentrated. The residue was purified by flash chromatography (SiO_2, 10% MeOH in DCM) to yield a light-yellow solid (410 mg, 2.1 mmol, 70%). ^{1}H NMR (400 MHz, CD_3OD) δ 7.70 (d, *J* = 8.4 Hz, 2H), 7.08 (d, *J* = 8.4 Hz, 2H), 3.88 (s, 3H). ^{13}C NMR (101 MHz, CD_3OD) δ 185.4, 165.1, 131.6, 124.7, 116.3, 115.3, 114.4, 61.3, 56.2. ESI-HRMS (m/z): calculated for $C_{11}H_7N_2O_2$ [M-H]$^-$: 199.0513; found, 199.0507.

To a solution of 2-(hydroxy(phenyl)methylene)malononitrile (200 mg, 1.0 mmol, 1.0 equiv.) in acetonitrile (30 mL) was added PCl_5 (624 mg, 3.0 mmol, 3.0 equiv.). After five hours at 50 °C under the argon atmosphere, the mixture was concentrated. The residue was dissolved in DCM (60 mL), washed with water (3×20 mL) and brine (20 mL), dried over Na_2SO_4, filtered, and concentrated. The resulting yellow solid was then dissolved in acetonitrile (5 mL) and water (5 mL). After 30 min, N-acetyl-L-cysteine (107 mg, 1.5 mmol, 1.5 equiv.) and $NaHCO_3$ (252 mg, 3.0 mmol, 3.0 equiv.) were added. The reaction was stirred at room temperature for two hours before acidification to pH 4–5 using 1 N $HCl_{(aq)}$. The acetonitrile was removed under reduced pressure. Brine (15 mL) was added. The mixture was extracted with EtOAc (3 × 20 mL). The combined organic phases were washed with brine (20 mL), dried over Na_2SO_4, filtered, and concentrated. The residue was purified by flash chromatography (SiO_2, 10% MeOH in DCM) to yield TAMM **1f** as a yellow powder (138 mg, 0.4 mmol, 40%). ^{1}H NMR (300 MHz, CD_3OD) δ 7.57 (d, *J* = 8.9 Hz, 2H), 7.14 (d, *J* = 8.9 Hz, 2H), 4.41 (dd, *J* = 8.5, 4.6 Hz, 1H), 3.90 (s, 2H), 3.34 – 2.99 (m, 2H), 2.00 (s, 3H). ^{13}C NMR (101 MHz, CD_3OD) δ 181.5, 173.3, 172.0, 165.0, 132.5, 125.5, 116.0, 114.4, 113.9, 80.1, 56.2, 52.9, 37.2, 22.4. ESI-HRMS (m/z): calculated for $C_{16}H_{16}N_3O_4S$ [M+H]$^+$: 346.0856; found, 346.0864.

Synthesis of TAMM **1g**

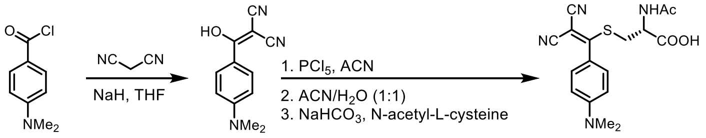

TAMM 1g

To a suspension of NaH (144 mg, 6.0 mmol, 2.0 equiv.) in anhydrous THF (3 mL) under argon atmosphere at 0 °C was added a solution of malononitrile (200 mg, 3.0 mmol, 1.0 equiv.) in anhydrous THF (2 mL) dropwise. After the addition, the reaction was stirred for one hour at 0 °C. A solution of 4-(dimethylamino)benzoyl chloride (549 mg, 3.0 mmol, 1.0 equiv.) in anhydrous THF (3 mL) was added into the mixture dropwise at 0 °C. The reaction mixture was then allowed to warm to room temperature and stirred at room temperature for another one hour. The solvent was then removed under reduced pressure. Ice water (10 mL) was added slowly, and the mixture was acidified to pH 1–2 using 1 N $HCl_{(aq)}$ and extracted with EtOAc (3 × 10 mL). The combined organic phases were washed with brine (3 × 10 mL), dried over Na_2SO_4, filtered, and concentrated. The residue was purified by flash chromatography (SiO_2, 10% MeOH in DCM) to yield a light-yellow solid (498 mg, 2.3 mmol, 78%). ^1H NMR (400 MHz, CD_3OD) δ 7.67 (d, *J* = 9.2 Hz, 2H), 6.89 (d, *J* = 8.8 Hz, 2H), 3.11 (s, 6H). ^{13}C NMR (101 MHz, CD_3OD) δ 185.5, 154.2, 131.4, 121.0, 118.0, 116.0, 113.0, 57.3, 40.9. ESI-HRMS (m/z): calculated for $C_{12}H_{10}N_3O_2$ $[M-H]^-$: 212.0829; found, 212.0827.

To a solution of 2-((4-(dimethylamino)phenyl)(hydroxy)methylene)malononitrile (213 mg, 1.0 mmol, 1.0 equiv.) in acetonitrile (30 mL) was added PCl_5 (624 mg, 3.0 mmol, 3.0 equiv.). After five hours at 50 °C under argon atmosphere, the mixture was concentrated. The residue was dissolved in DCM (60 mL), washed with water (3 × 20 mL) and brine (20 mL), dried over Na_2SO_4, filtered, and concentrated. The resulting yellow solid was then dissolved in acetonitrile (5 mL) and water (5 mL), N-acetyl-L-cysteine (107 mg, 1.5 mmol, 1.5 equiv.) and $NaHCO_3$ (252 mg, 3.0 mmol, 3.0 equiv.) were added. The reaction was stirred at room temperature for two hours before acidification to pH 4–5 using 1 N $HCl_{(aq)}$. The acetonitrile was removed under reduced pressure. Brine (15 mL) was added. The mixture was extracted with EtOAc (3 × 20 mL). The combined organic phases were washed with brine (20 mL), dried over Na_2SO_4, filtered, and concentrated. The residue was purified by flash chromatography (SiO_2, 10% MeOH in DCM) to yield TAMM **1g** as a yellow powder (161 mg, 0.45 mmol, 45%). ^1H NMR (400 MHz, CD_3OD) δ 7.45 (d, *J* = 9.1 Hz, 2H), 6.75 (d, *J* = 9.1 Hz, 2H), 4.33 (dd, *J* = 8.5, 4.6 Hz, 1H), 3.40 – 2.91 (m, 2H), 3.01 (s, 6H), 1.90 (s, 3H). ^{13}C NMR (101 MHz, CD_3OD) δ 180.0, 173.3, 172.3, 155.5, 133.2, 119.7, 115.7, 115.1, 112.7, 75.7, 53.0, 40.2, 37.6, 22.4. ESI-HRMS (m/z): calculated for $C_{17}H_{19}N_4O_3S$ $[M+H]^+$: 359.1172; found, 359.1178.

Synthesis of TAMM **1h**

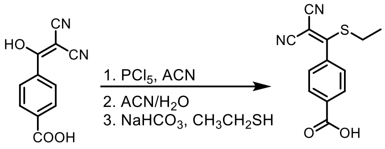

To a solution of 4-(2,2-dicyano-1-hydroxyvinyl)benzoic acid (214 mg, 1.0 mmol, 1.0 equiv., see synthesis of TAMM **1a** for its preparation) in acetonitrile (30 mL) was added PCl_5 (624 mg, 3.0 mmol, 3.0 equiv.). After five hours at 50 °C under argon atmosphere, the mixture was concentrated. The residue was dissolved in DCM (60 mL), washed with water (3 × 20 mL) and brine (20 mL), dried over Na_2SO_4, filtered, and concentrated. The resulting yellow solid was then dissolved in acetonitrile (5 mL) and water (5 mL). After 30 min, ethanethiol (111 µL, 1.5 mmol, 1.5 equiv.) and $NaHCO_3$ (252 mg, 3.0 mmol, 3.0 equiv.) were added. The reaction was stirred at room temperature for two hours before acidification to pH 4–5 using 1 N $HCl_{(aq)}$. The acetonitrile was removed under reduced pressure. Brine (15 mL) was added. The mixture was extracted with EtOAc (3 × 20 mL). The combined organic phases were washed with brine (20 mL), dried over Na_2SO_4, filtered, and concentrated. The residue was purified by flash chromatography (SiO_2, 3% MeOH in DCM) to yield TAMM **1h** as a yellow powder (80 mg, 0.31 mmol, 31%). ^{1}H NMR (400 MHz, CD_3OD) δ 8.21 (d, J = 8.4 Hz, 2H), 7.62 (d, J = 8.4 Hz, 2H), 2.75 (q, J = 7.4 Hz, 2H), 1.14 (t, J = 7.4 Hz, 3H). ^{13}C NMR (101 MHz, CD_3OD) δ 182.8, 168.3, 138.3, 135.1, 131.6, 129.8, 113.6, 113.1, 80.7, 30.0, 14.4. ESI-MS (m/z): calculated for $C_{13}H_9N_2O_2S$ $[M-H]^-$: 257.0390; found, 257.0381.

Synthesis of TAMM **1i**

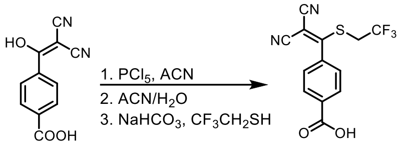

To a solution of 4-(2,2-dicyano-1-hydroxyvinyl)benzoic acid (214 mg, 1.0 mmol, 1.0 equiv., see synthesis of TAMM **1a** for its preparation) in acetonitrile (30 mL) was added PCl_5 (624 mg, 3.0 mmol, 3.0 equiv.). After five hours at 50 °C under argon atmosphere, the mixture was concentrated. The residue was dissolved in DCM (60 mL), washed with water (3 × 20 mL) and brine (20 mL), dried over Na_2SO_4, filtered, and concentrated. The resulting yellow solid was then dissolved in acetonitrile (5 mL) and water (5 mL). After 30 min, 2,2,2-trifluoroethanthiol (107 µL, 1.5 mmol, 1.5 equiv.) and $NaHCO_3$ (252 mg, 3.0 mmol, 3.0 equiv.) were added. The reaction was stirred at room temperature for two hours before acidification to pH 4–5 using 1 N $HCl_{(aq)}$. The acetonitrile was removed under reduced pressure. Brine (15 mL) was added. The mixture was extracted with EtOAc (3 × 20 mL). The combined organic phases were washed with brine (20 mL), dried over Na_2SO_4, filtered, and concentrated. The residue was purified by flash chromatography (SiO_2, 3% MeOH in DCM) to yield TAMM **1i** as a yellow powder (91 mg, 0.29 mmol, 29%). ^{1}H NMR (300 MHz, CD_3OD) δ 8.24 (d, J = 7.8 Hz, 2H), 7.67 (d, J = 8.1 Hz, 2H), 3.52 (q, J = 14.7 Hz, 2H). ^{13}C NMR (101 MHz, CD_3OD) δ 176.2, 166.9, 135.3, 134.7, 130.4, 129.1, 124.3 (q, J = 34.3 Hz), 111.6, 111.1, 83.7, 34.6 (q, J = 34.3 Hz). ^{19}F NMR (376 MHz, CD_3OD) δ -67.80 (t, J = 9.4 Hz, 3F). ESI-HRMS (m/z): calculated for $C_{13}H_6F_3N_2O_2S$ $[M-H]^-$: 311.0108; found, 311.0104.

Synthesis of TAMM **1j**

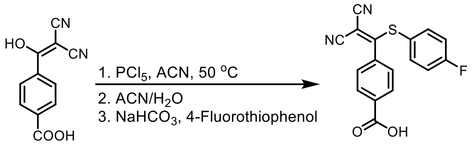

TAMM 1j

To a solution of 4-(2,2-dicyano-1-hydroxyvinyl)benzoic acid (214 mg, 1.0 mmol, 1.0 equiv., see synthesis of TAMM **1a** for its preparation) in acetonitrile (30 mL) was added PCl_5 (624 mg, 3.0 mmol, 3.0 equiv.). After five hours at 50 °C under argon atmosphere, the mixture was concentrated. The residue was dissolved in DCM (60 mL), washed with water (3 × 20 mL) and brine (20 mL), dried over Na_2SO_4, filtered, and concentrated. The resulting yellow solid was then dissolved in acetonitrile (5 mL) and water (5 mL). After 30 min, 4-Fluorothiophenol (192 mg, 1.5 mmol, 1.5 equiv.) and $NaHCO_3$ (252 mg, 3.0 mmol, 3.0 equiv.) were added. The reaction was stirred at room temperature for two hours before acidification to pH 4–5 using 1 N $HCl_{(aq)}$.The acetonitrile was removed under reduced pressure. Brine (15 mL) was added. The mixture was extracted with EtOAc (3 × 20 mL). The combined organic phases were washed with brine (20 mL), dried over Na_2SO_4, filtered, and concentrated. The residue was purified by flash chromatography (SiO_2, 3% MeOH in DCM) to yield TAMM **1j** as a yellow powder yield a yellow powder (178 mg, 0.55 mmol, 55%). ^{1}H NMR (300 MHz, Acetone-d_6) δ 8.05 – 7.87 (m, 2H), 7.71 – 7.42 (m, 4H), 7.08 (t, *J* = 8.8 Hz, 2H). ^{13}C NMR (101 MHz, Acetone-d_6) δ 180.1, 166.4, 164.7 (d, *J* = 250.6 Hz), 138.5 (d, *J* = 9.2 Hz), 137.7, 133.8, 130.5, 130.4, 124.0 (d, *J* = 3.4 Hz), 117.6 (d, *J* = 22.7 Hz), 113.3, 112.7, 81.3. ^{19}F NMR (376 MHz, Acetone-d_6) δ -110.47 (s, 1F). ESI-HRMS (m/z): calculated for $C_{17}H_8FN_2O_2S$ $[M-H]^-$: 323.0296; found, 323.0294.

Synthesis of TAMM **1k**

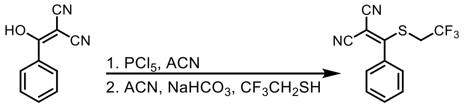

TAMM 1k

To a solution of 2-(hydroxy(phenyl)methylene)malononitrile (170 mg, 1.0 mmol, 1.0 equiv., see synthesis of TAMM **1e** for its preparation) in acetonitrile (30 mL) was added PCl_5 (624 mg, 3.0 mmol, 3.0 equiv.). After five hours at 50 °C under argon atmosphere, the mixture was concentrated. The residue was dissolved in DCM (60 mL), washed with water (3 × 20 mL) and brine (20 mL), dried over Na_2SO_4, filtered, and concentrated. The resulting yellow solid was then dissolved in acetonitrile (5 mL), 2,2,2-trifluoroethanthiol (107 µL, 1.5 mmol, 1.5 equiv.) and $NaHCO_3$ (252 mg, 3.0 mmol, 3.0 equiv.) were added. The reaction was stirred at room temperature for 2 h. The acetonitrile was removed under reduced pressure. The residue was purified by flash chromatography (SiO_2, 50% EtOAc in PE) to yield TAMM **1k** as a yellow powder (123 mg, 0.46 mmol, 46%). ^{1}H NMR (600 MHz, $CDCl_3$) δ 7.67 – 7.62 (m, 1H), 7.60 (t, *J* = 7.6 Hz, 2H), 7.46 (d, *J* = 7.3 Hz, 2H), 3.22 (q, *J* = 9.0 Hz, 1H).^{113}C NMR (151 MHz, $CDCl_3$) δ 176.7, 133.3, 131.2, 130.1, 129.0, 123.8 (q, *J* = 277.0 Hz), 112.0, 111.5, 83.8, 35.7 (q, *J* = 34.6 Hz). ^{19}F NMR (376 MHz, $CDCl_3$) δ -66.16 (t, *J* = 9.0 Hz, 3F). ESI-HRMS (m/z): calculated for $C_{12}H_6F_3N_2S$ $[M-H]^-$: 267.0209; found, 267.0207.

Synthesis of TAMM **1l**

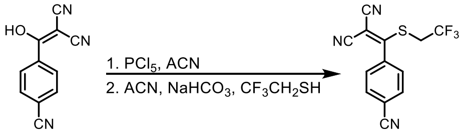

TAMM 1l

To a solution of 2-((4-cyanophenyl)(hydroxy)methylene)malononitrile (195 mg, 1.0 mmol, 1.0 equiv., see synthesis of TAMM **1b** for its preparation) in acetonitrile (30 mL) was added PCl_5 (624 mg, 3.0 mmol, 3.0 equiv.). After five hours at 50 °C under the argon atmosphere, the mixture was concentrated. The residue was dissolved in DCM (60 mL), washed with water (3 × 20 mL) and brine (20 mL), dried over Na_2SO_4, filtered, and concentrated. The resulting yellow solid was then dissolved in acetonitrile (5 mL), 2,2,2-trifluoroethanthiol (107 µL, 1.5 mmol, 1.5 equiv.) and $NaHCO_3$ (252 mg, 3.0 mmol, 3.0 equiv.) were added. The reaction was stirred at room temperature for 2 h. The acetonitrile was removed under reduced pressure and the residue was purified by flash chromatography (SiO_2, 50% EtOAc in PE) to yield TAMM **1l** as a yellow powder (82 mg, 0.28 mmol, 28%). ^{1}H NMR (600 MHz, $CDCl_3$) δ 7.90 (d, *J* = 8.4 Hz, 2H), 7.60 (d, *J* = 8.3 Hz, 2H), 3.22 (q, *J* = 8.9 Hz, 2H). ^{13}C NMR (151 MHz, $CDCl_3$) δ 173.7, 135.4, 133.7, 129.7, 123.6 (q, *J* = 277.2 Hz), 117.1, 117.0, 111.3, 110.8, 35.6 (q, *J* = 34.8 Hz). ^{19}F NMR (376 MHz, $CDCl_3$) δ -66.00 (t, *J* = 9.0 Hz, 3F). ESI-HRMS (m/z): calculated for $C_{13}H_5F_3N_3S$ $[M-H]^-$: 292.0162; found, 292.0161.

Synthesis of TAMM **1m**

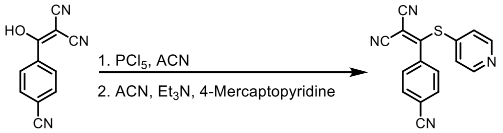

TAMM 1m

To a solution of 2-((4-cyanophenyl)(hydroxy)methylene)malononitrile (195 mg, 1.0 mmol, 1.0 equiv., see synthesis of TAMM **1b** for its preparation) in acetonitrile (30 mL) was added PCl_5 (624 mg, 3.0 mmol, 3.0 equiv.). After five hours at 50 °C under the argon atmosphere, the mixture was concentrated. The residue was dissolved in DCM (60 mL), washed with water (3 × 20 mL) and brine (20 mL), dried over Na_2SO_4, filtered, and concentrated. The resulting yellow solid was then dissolved in acetonitrile (5 mL), 4-mercaptopyridine (167 mg, 1.5 mmol, 1.5 equiv.) and Et_3N (412 µL, 3.0 mmol, 3.0 equiv.) were added. The reaction was stirred at room temperature for two hours. The acetonitrile was removed under reduced pressure and the residue was purified by flash chromatography (SiO_2, 50% EtOAc in PE) to yield TAMM **1m** as a yellow powder (75 mg, 0.26 mmol, 26%). ^{1}H NMR (600 MHz, $CDCl_3$) δ 8.51 (s, 2H), 7.65 (d, *J* = 8.0 Hz, 2H), 7.52 (d, *J* = 8.0 Hz, 2H), 7.12 (s, 2H). ^{13}C NMR (151 MHz, $CDCl_3$) δ 173.5, 150.6, 138.1, 135.8, 132.8, 129.9, 127.1, 116.9, 116.3, 111.5, 110.9, 84.2. ESI-HRMS (m/z): calculated for $C_{16}H_9N_4S$ $[M+H]^+$: 289.0542; found, 289.0537.

Synthesis of TAMM **1n**

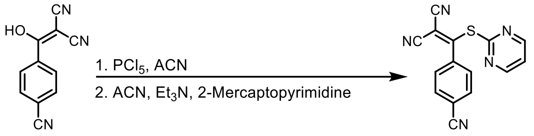

TAMM 1n

To a solution of 2-((4-cyanophenyl)(hydroxy)methylene)malononitrile (195 mg, 1.0 mmol, 1.0 equiv., see synthesis of TAMM **1b** for its preparation) in acetonitrile (30 mL) was added PCl_5 (624 mg, 3.0 mmol, 3.0 equiv.). After five hours at 50 °C under argon atmosphere, the mixture was concentrated. The residue was dissolved in DCM (60 mL), washed with water (3 × 20 mL) and brine (20 mL), dried over Na_2SO_4, filtered, and concentrated. The resulting yellow solid was then dissolved in acetonitrile (5 mL), 2-Mercaptopyrimidine (168 mg, 1.5 mmol, 1.5 equiv.) and Et_3N (412 µL, 3.0 mmol, 3.0 equiv.) were added. The reaction was stirred at room temperature for 2 h. The acetonitrile was removed under reduced pressure and the residue was purified by flash chromatography (SiO_2, 50% EtOAc in PE) to yield TAMM **1n** as a yellow powder (64 mg, 0.22 mmol, 22%). ^{1}H NMR (600 MHz, $CDCl_3$) δ 8.44 (d, *J* = 4.9 Hz, 2H), 7.81 (d, *J* = 8.5 Hz, 2H), 7.68 (d, *J* = 8.5 Hz, 2H), 7.10 (t, *J* = 4.9 Hz, 1H). ^{13}C NMR (151 MHz, $CDCl_3$) δ 170.7, 166.4, 158.3, 138.5, 132.5, 130.4, 119.7, 117.5, 116.0, 112.0, 111.6, 87.9. ESI-HRMS (m/z): calculated for $C_{15}H_8N_5S$ $[M+H]^+$: 290.0495; found, 290.0490.

Synthesis of **Cy5-TAMM-SCH$_2$CF$_3$**

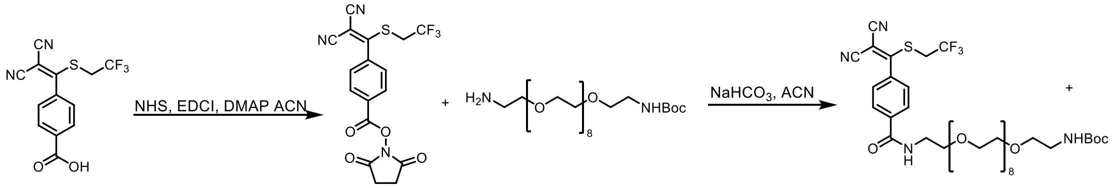

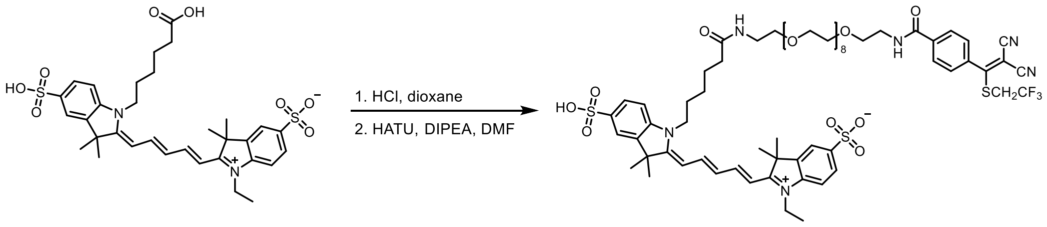

Cy5-TAMM-SCH$_2$CF$_3$

To a solution of 4-(2,2-dicyano-1-((2,2,2-trifluoroethyl)thio)vinyl)benzoic acid (62 mg, 0.2 mmol, 1.0 equiv., see synthesis of TAMM **1i** for its preparation) in acetonitrile (2 mL) was added *N*-hydroxyl succinimide (46 mg, 0.4 mmol, 2.0 equiv.), DMAP (2.4 mg, 0.02 mmol, 0.1 equiv.) and 1-ethyl-(3-dimethylaminopropyl) carbodiimine hydrochloride (77 mg, 0.4 mmol, 2.0 equiv.). After two hours at room temperature, the solvent was removed under reduced pressure. The residue was purified by flash column chromatography (SiO_2, 10% MeOH in DCM) to yield a yellow powder (41 mg, 0.1 mmol, 51%). ^{1}H NMR (400 MHz, CD_3OD) δ 8.38 (d, *J* = 8.4 Hz, 2H), 7.93(d, *J* = 8.4 Hz, 2H), 3.80 (q, *J* = 9.6 Hz, 2H), 2.99 (s, 4H). ^{13}C NMR (101 MHz, Acetone-d_6) δ 176.0, 170.4, 162.0, 138.6, 132.1, 131.0, 129.6, 126.8, 124.0, 112.7, 112.4, 85.7, 35.6 (q, *J* = 33.9 Hz), 26.4. ESI-HRMS (m/z): calculated for $C_{17}H_9F_3N_3O_4S$ $[M-H]^-$: 408.0271; found, 408.0281.

To a solution of 2,5-dioxopyrrolidin-1-yl 4-(2,2-dicyano-1-((2,2,2-trifluoroethyl)thio)vinyl)benzoate (58 mg, 0.14 mmol, 1.0 equiv.) in acetonitrile (2 mL) was

added tert-butyl (29-amino-3,6,9,12,15,18,21,24,27-nonaoxanonacosyl)carbamate (78 mg, 0.14 mmol, 1.0 equiv.) and $NaHCO_3$ (24 mg, 0.28 mmol, 2.0 equiv.). After one hour at room temperature, the solvent was removed under reduced pressure. The residue was purified by flash column chromatography (SiO_2, 10% MeOH in DCM) to yield a yellow oil (60 mg, 0.07 mmol, 50%). ^{1}H NMR (400 MHz, CD_3OD) δ 7.77 (d, *J* = 8.4 Hz, 2H), 7.39 (d, *J* = 8.0 Hz, 2H), 3.41 – 3.25 (m, 38H), 3.21 (t, *J* = 5.6 Hz, 2H), 2.92 (t, *J* = 5.6 Hz, 2H), 1.14 (s, 9H). ^{13}C NMR (101 MHz, CD_3OD) δ 177.7, 168.8, 139.8, 135.4, 130.6, 129.7, 124.3, 113.1, 112.6, 84.9, 80.1, 71.6, 71.5, 71.3, 71.3, 71.1, 70.4, 41.3, 41.2, 36.0 (q, *J* = 34.0 Hz), 28.8. ESI-HRMS (m/z): calculated for $C_{38}H_{58}F_3N_3O_{12}S$ $[M+H]^+$: 851.3719; found, 851.3719.

For Boc removal, tert-butyl (1-(4-(2,2-dicyano-1-((2,2,2-trifluoroethyl)thio)vinyl)phenyl)-1-oxo-5,8,11,14,17,20,23,26,29-nonaoxa-2-azahentriacontan-31-yl)carbamate (60 mg, 0.07 mmol, 1.0 equiv.) was stirred in 4 M HCl (1 mL) in dioxane at room temperature. After 30 min, the solvent was evaporated under reduced pressure to give the hydrochloride. For conjugation, Cy5-COOH (10 mg, 15 μmol. 1.2 equiv.) was dissolved in DMF (1 mL), then HATU (9 mg, 24 μmol, 2.0 equiv.) and DIPEA (6 μL, 36 μmol. 3.0 equiv.) were added. The solution was stirred in room temperature for an hour. To above solution *N*-(29-amino-3,6,9,12,15,18,21,24,27-nonaoxanonacosyl)-4-(2,2-dicyano-1-((2,2,2-trifluoroethyl)thio)vinyl)-benzamide hydrochloride was added, and continue stirred for 12 hours. The DMF was removed under reduced pressure, and the residue was purified by RP-HPLC to give **Cy5-TAMM-SCH₂CF₃** (5 mg, 3.6 μmol, 30 %). ^{1}H NMR (600 MHz, CD_3OD) δ 8.30 (t, *J* = 11.1 Hz, 2H), 8.06 (d, *J* = 8.4 Hz, 2H), 7.91 – 7.88 (m, 4H), 7.67 (d, *J* = 8.4 Hz, 2H), 7.35 (d, *J* = 5.4 Hz, 2H), 6.69 (t, *J* = 12.0 Hz, 1H), 6.35 (t, *J* = 11.7 Hz, 1H), 4.18 – 4.11 (m, 2H), 3.67 (t, *J* = 5.4 Hz, 2H), 3.64 – 3.58 (m, 36H), 3.51 (t, *J* = 5.7 Hz, 2H), 3.33 (t, *J* = 5.4 Hz, 2H), 2.22 (t, *J* = 7.5 Hz, 2H), 1.84 – 1.80 (m, 2H), 1.74 (s, 12H), 1.71 – 1.66 (m, 2H), 1.48 – 1.43 (m, 2H), 1.38 (t, *J* = 7.2 Hz, 2H). ^{13}C NMR (151 MHz, CD_3OD) δ 177.8, 175.9, 175.2, 175.0, 168.7, 156.4, 156.2, 145.0, 144.5, 143.3, 142.8, 142.6, 139.7, 135.4, 130.6, 129.8, 128.2, 127.8, 126.6, 124.8, 121.4, 121.4, 113.2, 112.6, 111.7, 111.5, 105.3, 105.0, 84.8, 71.54, 71.51, 71.46, 71.3, 71.2, 70.6, 70.4, 50.6, 50.6, 49.8, 45.0, 41.2, 40.4, 40.3, 36.6, 36.0 (q, *J* = 34.9 Hz), 28.1, 27.9, 27.8, 27.3, 26.5, 12.6. ESI-HRMS (m/z): calculated for $C_{66}H_{86}F_3N_6O_{17}S_3$ $[M-H]^-$: 1387.5169; found, 1387.5160.

Synthesis of **Cy3-TAMM-SCH₂CF₃**

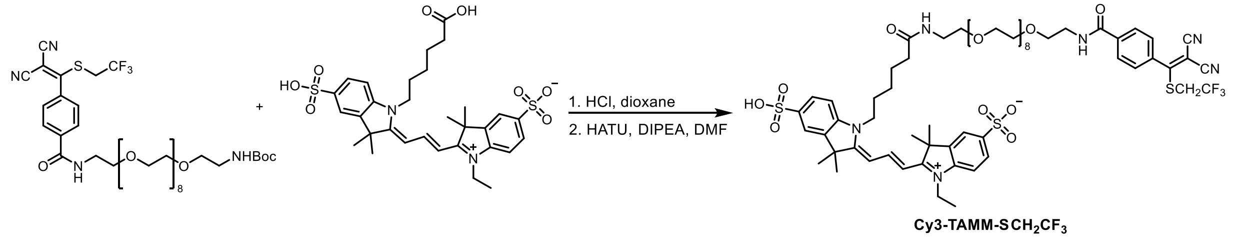

For Boc removal, tert-butyl (1-(4-(2,2-dicyano-1-((2,2,2-trifluoroethyl)thio)vinyl)phenyl)-1-oxo-5,8,11,14,17,20,23,26,29-nonaoxa-2-azahentriacontan-31-yl)carbamate (60 mg, 0.07 mmol, 1.0 equiv., see synthesis of **Cy5-TAMM-SCH₂CF₃** for its preparation) was stirred in 4 M HCl in dioxane (1 mL) at room temperature. After 30 min, the solvent was evaporated under reduced pressure to give the hydrochloride. For conjugation, to a solution of Cy3-COOH (10 mg, 16 μmol. 1.2 equiv.) in DMF (1 mL) was added HATU (10 mg, 26 mmol, 2.0 equiv.) and DIPEA (7 μL, 39 μmol. 3.0 equiv.). The mixture was stirred at room temperature for an hour, then *N*-(29-amino-3,6,9,12,15,18,21,24,27-nonaoxanonacosyl)-4-(2,2-dicyano-1-((2,2,2-trifluoroethyl)thio)vinyl)-benzamide hydrochloride was added, and continued stirred for 12

hours. The DMF was removed under reduced pressure, and the residue was purified by RP-HPLC to give **Cy3-TAMM-SCH$_2$CF$_3$** (5 mg, 4 µmol, 29 %). ^{1}H NMR (600 MHz, CD$_3$OD) δ 8.58 (t, *J* = 13.5 Hz, 1H), 8.05 (d, *J* = 8.4 Hz, 2H), 7.96 (d, *J* = 4.8 Hz, 2H), 7.94 – 7.91 (m, 2H), 7.67 (d, *J* = 8.4 Hz, 2H), 7.42 (dd, *J* = 8.4, 3.0 Hz, 2H), 6.53 (dd, *J* = 22.2, 13.8 Hz, 2H), 4.24 (q, *J* = 7.2 Hz, 2H), 4.18 (t, *J* = 7.8 Hz, 2H), 3.68 – 3.66 (m, 2H), 3.62 – 3.55 (m, 34H), 3.51 (t, *J* = 7.3 Hz, 2H), 3.33 (t, *J* = 5.4 Hz, 2H), 2.23 (t, *J* = 7.2 Hz, 2H), 1.88 – 1.85 (m, 2H), 1.79 (s, 12H), 1.74 – 1.69 (m, 2H), 1.52– 1.47 (m, 2H), 1.44 (t, *J* = 7.2 Hz, 3H). ^{13}C NMR (151 MHz, CD$_3$OD) δ 177.7, 176.8, 176.6, 175.9, 168.7, 152.9, 144.6, 144.2, 142.4, 142.2, 139.8, 135.4, 130.6, 129.7, 128.3, 121.6, 121.5, 113.1, 113.0, 112.6, 112.2, 112.0, 104.6, 104.5, 84.9, 71.5, 71.4, 71.3, 71.2, 70.6, 70.4, 50.8, 50.8, 45.4, 41.2, 40.7, 40.3, 36.6, 36.0 (q, *J* = 34.7 Hz), 28.3, 28.1, 27.3, 26.5, 12.7. ESI-HRMS (m/z): calculated for $C_{64}H_{84}F_3N_6O_{17}S_3$ [M-H]$^-$: 1361.5013; found, 1361.4997.

Synthesis of **Cy5-TAMM-SEt**

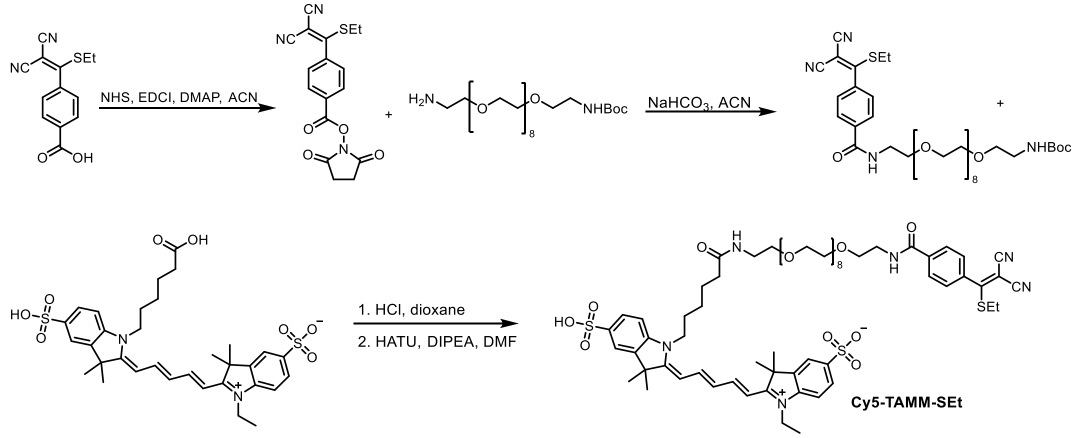

To a solution of 4-(2,2-dicyano-1-((2,2,2-trifluoroethyl)thio)vinyl)benzoic acid (52 mg, 0.2 mmol, 1.0 equiv., see synthesis of TAMM **1h** for its preparation) in acetonitrile (2 mL) was added *N*-hydroxyl succinimide (46 mg, 0.4 mmol, 2.0 equiv.), DMAP (2.4 mg, 0.02 mmol, 0.1 equiv.) and 1-ethyl-(3-dimethylaminopropyl) carbodiimine hydrochloride (77 mg, 0.4 mmol, 2.0 equiv.). After two hours at room temperature, the solvent was removed under reduced pressure. The residue was purified by flash column chromatography (SiO$_2$, 50% EtOAc in PE to yield a yellow powder (53 mg, 0.15 mmol, 75%). ^{1}H NMR (400 MHz, Acetone-*d*$_6$) δ 8.36 (d, *J* = 8.8 Hz, 2H), 7.87 (d, *J* = 8.8 Hz, 2H), 2.99 (s, 4H), 2.83 (q, *J* = 7.4 Hz, 2H) 1.17 (t, *J* = 7.4 Hz, 3H). ^{13}C NMR (101 MHz, Acetone-*d*$_6$) δ 181.2, 170.4, 162.0, 140.1, 131.9, 130.3, 128.9, 113.2, 112.9, 81.1, 29.7, 26.4, 14.8. ESI-HRMS (m/z): calculated for $C_{17}H_{13}N_3NaO_4S$ [M+Na]$^+$: 378.0519; found, 378.0509.

To a solution of 2,5-dioxopyrrolidin-1-yl 4-(2,2-dicyano-1-(ethylthio)vinyl)benzoate (53 mg, 0.15 mmol, 1.0 equiv.) in acetonitrile (2 mL), o-(2-aminoethyl)-o-(2-(BOC-amino)ethyl)octaethylene glycol (83 mg, 0.15 mmol, 1.0 equiv.) and NaHCO$_3$ (25 mg. 0.3 mmol, 2.0 equiv.) were added. The mixture was stirred for an hour. The acetonitrile was removed under reduced pressure, and the residue was purified by flash chromatography (SiO$_2$, 10% methanol in DCM) to yield a yellow oil (72 mg, 0.09 mmol, 60%).^{1}H NMR (400 MHz, CD$_3$OD) δ 8.04 (d, *J* = 8.4 Hz, 1H), 7.62 (d, *J* = 8.4 Hz, 1H), 3.70 – 3.68 (m, 2H), 3.66 – 3.59 (m, 34H), 3.50 (t, *J* = 5.6 Hz, 1H), 3.24 – 3.20 (m, 2H), 2.76 (q, *J* = 7.5 Hz, 2H), 1.44 (s, 9H), 1.14 (t, *J* = 7.4 Hz, 3H). ^{13}C NMR (101 MHz, CD$_3$OD) δ 182.8, 168.9, 138.9, 137.0, 129.8, 129.4, 113.7, 113.2, 80.6, 71.6, 71.5, 71.3, 71.3, 71.1, 70.4, 41.2, 30.1, 28.8, 14.9. ESI-HRMS (m/z): calculated for $C_{38}H_{61}N_4O_{12}S$ [M+H]$^+$: 795.3856; found, 795.3883.

For Boc removal, tert-butyl (1-(4-(2,2-dicyano-1-(ethylthio)vinyl)phenyl)-1-oxo-5,8,11,14,17,20,23,26,29-nonaoxa-2-azahentriacontan-31-yl)carbamate (20 mg, 25 µmol, 1.0 equiv.) was stirred in 4 M HCl in dioxane (1 mL) at room temperature. After 30 min, the solvent was evaporated under reduced pressure to give the hydrochloride. For conjugation, Cy5-COOH (10 mg, 15 µmol. 1.2 equiv.) was dissolved in DMF (1 mL), then HATU (9 mg, 24 µmol, 2.0 equiv.) and DIPEA (6 µL, 36 µmol. 3.0 equiv.) were added. The solution was stirred at room temperature for one hour. To above solution *N*-(29-amino-3,6,9,12,15,18,21,24,27-nonaoxanonacosyl)-4-(2,2-dicyano-1-(ethylthio)vinyl)benzamide hydrochloride was added, and continued stirred for 12 hours. The DMF was removed under reduced pressure, and the residue was purified by RP-HPLC to give Cy5-TAMM-SEt (6 mg, 5 µmol, 40 %). ^{1}H NMR (600 MHz, CD_3OD) δ 8.03 (d, *J* = 8.4 Hz, 2H), 7.91 – 7.88 (m, 4H), 7.61 (d, *J* = 8.4 Hz, 2H), 7.35 (dd, *J* = 7.8, 3.6 Hz, 2H), 6.69 (t, *J* = 12.3 Hz, 1H), 6.35 (dd, *J* = 13.8, 10.2 Hz, 2H), 4.20 – 4.11 (m, 4H), 3.67 (t, *J* = 5.4 Hz, 2H), 3.64 – 3.57 (m, 35H), 3.51 (t, *J* = 5.4 Hz, 2H), 3.34 (t, *J* = 5.4 Hz, 1H), 2.76 (q, *J* = 7.4 Hz, 2H), 2.23 (t, *J* = 7.5 Hz, 2H), 1.85 – 1.79 (m, 2H), 1.72 – 1.65 (m, 2H), 1.49 – 1.44 (m, 2H), 1.39 (t, *J* = 7.2 Hz, 3H), 1.13 (t, *J* = 7.2 Hz, 3H). ^{13}C NMR (151 MHz, CD_3OD) δ 182.9, 176.0, 175.2, 175.0, 168.8, 156.4, 156.2, 144.9, 144.4, 143.4, 142.8, 142.6, 138.9, 137.0, 129.8, 129.4, 128.1, 127.7, 121.4, 121.4, 113.8, 113.2, 111.7, 111.5, 105.2, 105.0, 80.6, 71.54, 71.52, 71.49, 71.46, 71.44, 71.3, 71.2, 70.5, 70.4, 50.6, 50.6, 50.6, 45.0, 41.1, 40.4, 40.3, 36.5, 30.1, 28.1, 27.9, 27.8, 27.3, 26.5, 14.9, 12.6. ESI-HRMS (m/z): calculated for $C_{66}H_{89}N_6O_{17}S_3$ $[M-H]^-$: 1333.5452; found, 1333.5421.

Synthesis of **Cy5-TAMM-SPhF**

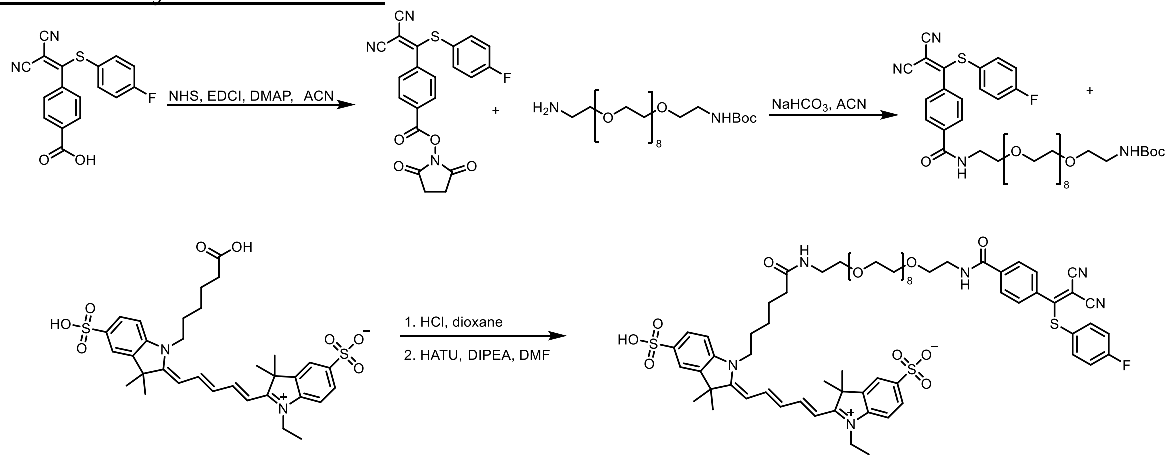

Cy5-TAMM-SPhF

4-(2,2-dicyano-1-((4-fluorophenyl)thio)vinyl)benzoic acid (65 mg, 0.2 mmol, 1.0 equiv., see synthesis of TAMM **1j** for its preparation) was dissolved in acetonitrile (2 mL), to this solution *N*-hydroxyl succinimide (46 mg, 0.4 mmol, 2.0 equiv.), DMAP (2.4 mg, 0.02 mmol, 0.1 equiv.) and 1-ethyl-(3-dimethylaminopropyl) carbodiimine hydrochloride (77 mg, 0.4 mmol, 2.0 equiv.) were added. The reaction was stirred at room temperature. After 2 h, the solvent was removed under reduced pressure. The residue was purified by flash chromatography (SiO_2, 1:2 petroleum ether/ethyl acetate) to yield a yellow powder (59 mg, 0.14 mmol, 70%). ^{1}H NMR (400 MHz, Acetone-d_6) δ 8.11 (d, *J* = 8.4 Hz, 2H), 7.74 (d, *J* = 8.8 Hz, 2H), 7.60 (dd, *J* = 9.2, 5.2 Hz, 2H), 7.09 (t, *J* = 8.8 Hz, 2H), 2.95 (s, 3H). ^{13}C NMR (101 MHz, Acetone-d_6) δ 179.3, 179.2, 170.4, 166.1, 163.6, 161.9, 139.9, 138.6 (d, *J* = 9.2 Hz), 131.0 (d, *J* = 14.6 Hz), 128.5, 123.6 (d, *J*

= 3.3 Hz), 117.7 (d, J = 22.7 Hz), 113.1, 112.5, 81.9, 26.4. ESI-HRMS (m/z): calculated for $C_{21}H_{12}FN_3NaO_4S$ $[M+Na]^+$: 444.0425; found, 444.0411.

To a solution of 2,5-dioxopyrrolidin-1-yl 4-(2,2-dicyano-1-((4-fluorophenyl)thio)vinyl)benzoate (59 mg, 0.14 mmol, 1.0 equiv.) in acetonitirle (2 mL), o-(2-aminoethyl)-o-(2-(BOC-amino)ethyl)octaethylene glycol (79 mg, 0.14 mmol, 1.0 equiv.) and $NaHCO_3$ (25 mg. 0.28 mmol, 2.0 equiv.) were added. The mixture was stirred for one hour. The acetonitrile was removed under reduced pressure, and the residue was purified by flash chromatography (SiO_2, 10% methanol in DCM) to yield a yellow oil (66 mg, 0.08 mmol, 55%). ^{1}H NMR (400 MHz, CD_3OD) δ 7.81 (d, J = 8.4 Hz, 2H), 7.52 – 7.47 (m, 4H), 7.02 (t, J = 8.8 Hz, 2H), 3.67 – 3.59 (m, 34H), 3.56 – 3.51 (m, 4H), 3.25 – 3.22 (m, 2H), 1.45 (s, 9H). ^{13}C NMR (101 MHz, CD_3OD) δ 181.0, 168.6, 166.5, 164.0, 158.4, 138.7 (d, J = 9.0 Hz), 138.4, 136.8, 130.6, 128.7, 124.4 (d, J = 3.5 Hz), 117.8 (d, J = 22.7 Hz), 113.6, 112.8, 81.2, 80.0, 71.53, 71.5, 71.47, 71.2, 71.0, 70.4, 41.3, 41.1, 28.8. ESI-HRMS (m/z): calculated for $C_{42}H_{60}FN_4O_{12}S$ $[M+H]^+$: 863.3907; found, 863.3890.

For Boc removal, tert-butyl (1-(4-(2,2-dicyano-1-((4-fluorophenyl)thio)vinyl)phenyl)-1-oxo-5,8,11,14,17,20,23,26,29-nonaoxa-2-azahentriacontan-31-yl)carbamate (20 mg, 23 μmol, 1.0 equiv.) was stirred in 4 M HCl (1 mL) in dioxane at room temperature. After 30 min, the solvent was evaporated under reduced pressure to give the hydrochloride. For conjugation, Cy5-COOH (10 mg, 15 μmol. 1.2 equiv.) was dissolved in DMF (1 mL), then HATU (9 mg, 24 μmol, 2.0 equiv.) and DIPEA (6 μL, 36 μmol. 3.0 equiv.) were added. The solution was stirred in room temperature for an hour. To above solution *N*-(29-amino-3,6,9,12,15,18,21,24,27-nonaoxanonacosyl)-4-(2,2-dicyano-1-((4-fluorophenyl)thio)vinyl)benzamide hydrochloride was added, and continue stirred for 12 hours. The DMF was removed under reduced pressure, and the residue was purified by RP-HPLC to give **Cy5-TAMM-SPhF** (8 mg, 50%). ^{1}H NMR (400 MHz, CD_3OD) δ 8.34 – 8.27 (m, 2H), 7.91 – 7.87 (m, 3H), 7.36 – 7.33 (m, 2H), 7.49 – 7.43 (m, 4H), 7.34 (dd, J = 8.2, 2.4 Hz, 2H), 6.99 (t, J = 8.6 Hz, 2H), 6.68 (t, J = 12.4 Hz, 1H), 6.34 (dd, J = 14.0, 7.2 Hz, 2H), 4.20 – 4.10 (m, 4H), 3.61– 3.58 (m, 34H), 3.53 – 3.49 (m, 4H), 3.34 – 3.31 (m, 2H), 2.21 (t, J = 7.2 Hz, 2H), 1.84 – 1.79 (m, 2H), 1.74 (s, 12H), 1.71 – 1.67 (m, 2H), 1.49 – 1.44 (m, 2H), 1.38 (t, J = 7.4 Hz, 3H). ^{13}C NMR (101 MHz, CD_3OD) δ 181.1, 175.9, 175.3, 175.1, 168.7, 166.6, 164.1, 156.4, 156.2, 144.9, 144.4, 142.8, 142.6, 138.7 (d, J = 9.0 Hz), 138.4, 136.8, 130.7, 128.7, 128.1, 127.7, 124.4 (d, J = 3.8 Hz), 121.4, 117.8 (d, J = 22.8 Hz), 113.6, 112.9, 111.7, 111.5, 105.2, 105.0, 81.2, 73.5, 71.52, 71.48, 71.46, 71.4, 71.2, 70.6, 70.4, 62.2, 55.8, 50.6, 50.6, 45.0, 44.4, 41.0, 40.3, 36.6, 28.1, 27.9, 27.7, 27.3, 26.5, 18.7, 17.3, 12.6. ESI-HRMS (m/z): calculated for $C_{70}H_{88}FN_6O_{17}S_3$ $[M-H]^-$: 1399.5358; found, 1399.5330.

Synthesis of **Cy3-tetrazine**

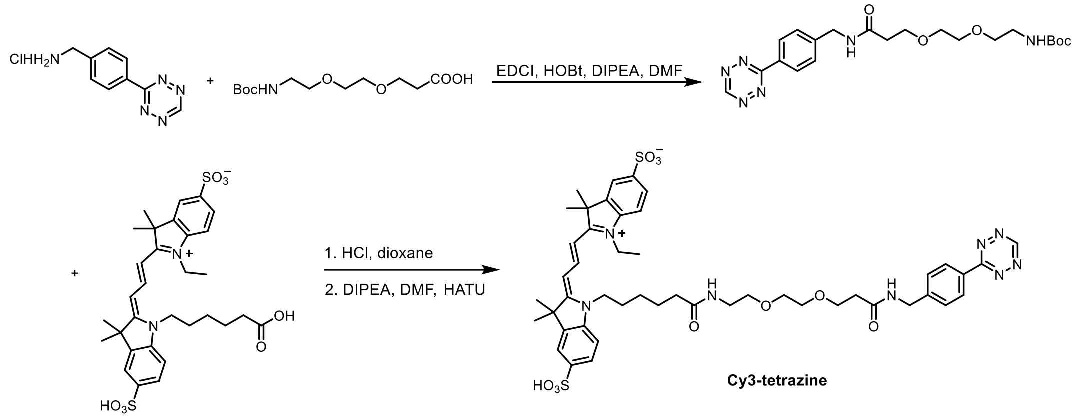

Dissolved (4-(1,2,4,5-tetrazin-3-yl)phenyl)methanamine hydrochloride (20 mg, 89 μmol, 1.0 equiv.) and 2,2-dimethyl-4-oxo-3,8,11-trioxa-5-azatetradecan-14-oic acid (37 mg, 134 μmol, 1.5 equiv.) in the DMF (2 mL), follow by EDCI (51 mg, 267 μmol, 3.0 equiv.), HOBt (6 mg, 45 μmol, 0.5 equiv.) and DIPEA (47 mg, 367 μmol, 3.0 equiv.) were added. The reaction mixture was stirred at room temperature and monitored by LC-MS. After 12 hours, the DMF was removed under reduced pressure, and the residue was purified by flash chromatography (SiO_2, 3% methanol in DCM) to yield a red powder (19 mg, 43 μmol, 48%). ^{1}H NMR (600 MHz, $CDCl_3$) δ 10.19 (s, 1H), 8.55 (d, *J* = 7.8 Hz, 2H), 7.50 (d, *J* = 7.8 Hz, 2H), 7.00 (s, 1H), 4.90 (s, 1H), 4.56 (d, *J* = 6.0 Hz, 2H), 3.78 (t, *J* = 5.7 Hz, 2H), 3.63 – 3.57 (m, 4H), 3.45 (s, 2H), 3.19 (s, 2H), 2.57 (t, *J* = 5.7 Hz, 2H), 1.38 (s, 9H). ^{13}C NMR (151 MHz, $CDCl_3$) δ 171.8, 166.4, 157.9, 156.0, 144.4, 130.6, 128.6, 128.3, 79.5, 70.4, 70.3, 70.0, 67.3, 43.0, 40.3, 37.1, 28.5. ESI-HRMS (m/z): calculated for $C_{21}H_{31}N_6O_5$ $[M+H]^+$: 447.2350; found, 447.2342.

For Boc removal, tert-butyl (2-(2-(3-((4-(1,2,4,5-tetrazin-3-yl)benzyl)amino)-3-oxopropoxy)ethoxy)ethyl)carbamate (19 mg, 42 μmol, 1.0 equiv.) was stirred in 4 M HCl in dioxane (1 mL) at room temperature. After 30 min, the solvent was evaporated under reduced pressure to give the hydrochloride. For conjugation, Cy3-COOH (10 mg, 16 μmol, 1.2 equiv.) was dissolved in DMF (1 mL), then HATU (10 mg, 26 μmol, 2.0 equiv.) and DIPEA (7 μL, 39 μmol, 3.0 equiv.) were added. The solution was stirred in room temperature for an hour. To the above solution *N*-(4-(1,2,4,5-tetrazin-3-yl)benzyl)-3-(2-(2-aminoethoxy)ethoxy)propanamide hydrochloride was added, and continue stirred for 12 hours. The DMF was removed under reduced pressure, and the residue was purified by RP-HPLC to give **Cy3-tetrazine** (3 mg, 3 μmol, 23%). ^{1}H NMR (400 MHz, CD_3OD) δ 10.30 (s, 1H), 8.54 (t, *J* = 13.5 Hz, 1H), 8.48 (d, *J* = 8.4 Hz, 2H), 7.95 (dd, *J* = 3.6, 1.7 Hz, 4H), 7.94 – 7.84 (m, 2H), 7.54 (d, *J* = 8.4 Hz, 2H), 7.44 – 7.34 (m, 2H), 6.60 – 6.47 (m, 2H), 4.50 (s, 2H), 4.25 – 4.19 (m, 2H), 4.18 – 4.12 (m, 2H), 3.78 (t, *J* = 6.0 Hz, 2H), 3.63 – 3.56 (m, 4H), 3.51 (t, *J* = 5.4 Hz, 2H), 3.33 – 3.31 (m, 2H), 2.55 (t, *J* = 6.0 Hz, 2H), 2.24 (t, *J* = 7.4 Hz, 2H), 1.86 – 1.80 (m, 2H), 1.78 (s, 3H), 1.77 (s, 9H) 1.71 – 1.60 (m, 2H), 1.52 – 1.44 (m, 2H), 1.40 (t, *J* = 7.6 Hz, 3H). ^{13}C NMR (101 MHz, CD_3OD) δ 176.7, 176.5, 176.3, 174.2, 167.5, 159.3, 152.8, 145.6, 144.7, 144.2, 144.0, 142.4, 142.2, 132.1, 129.2, 128.3, 121.5, 121.5, 112.2, 112.0, 104.7, 104.6, 71.3, 71.2, 70.4, 68.2, 50.7, 50.7, 49.8, 45.4, 43.8, 40.7, 40.6, 37.6, 36.3, 34.4, 28.3, 28.1, 27.2, 26.5, 25.6, 12.7. ESI-HRMS (m/z): calculated for $C_{47}H_{57}N_8O_{10}S_2$ $[M-H]^-$: 957.3645; found, 957.3600.

Synthesis of **Cy7-tetrazine**

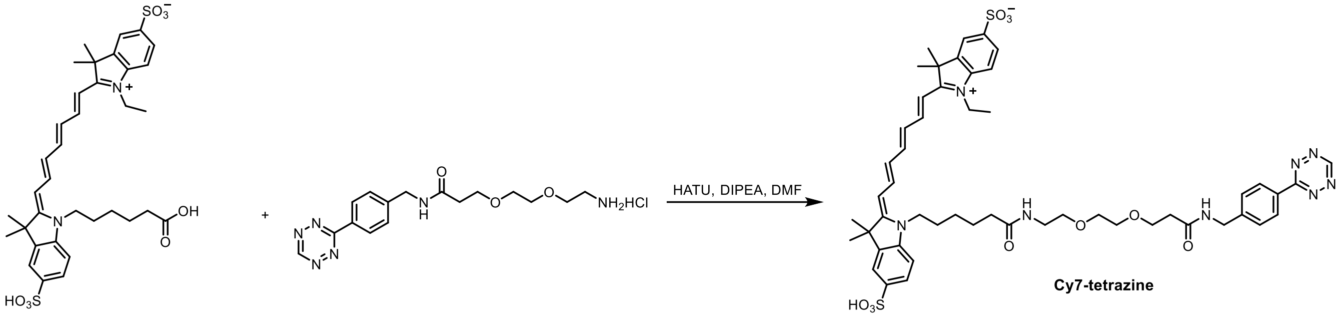

Cy7-COOH (10 mg, 15 μmol, 1.0 equiv.) was dissolved in DMF (1 mL), then HATU (11 mg, 30 μmol, 2.0 equiv.) and DIPEA (8 μL, 45 μmol, 3.0 equiv.) were added. The solution was stirred at room temperature for three hours. To above solution *N*-(4-(1,2,4,5-tetrazin-3-yl)benzyl)-3-(2-(2-aminoethoxy)ethoxy)propanamide hydrochloride (5.6 mg, 15 μmol, 1.0 equiv., see synthesis of **Cy3-tetrazine** for its preparation) was added, continue stirred for 16 h. The DMF was removed under reduced pressure, and the residue was purified by RP-HPLC to give **Cy7-tetrazine** (3 mg, 3 μmol, 20%). ^{1}H NMR (600 MHz, CD_3OD) δ 10.32 (s, 1H), 8.55 (d, *J* = 7.8 Hz, 2H), 8.02 – 7.98 (m, 2H), 7.91 – 7.87 (m, 5H), 7.58 (d, *J* = 9.6 Hz, 2H), 7.34 – 7.28 (m, 2H), 6.60 (t, *J* = 14.1 Hz, 2H), 6.38 – 6.30 (m, 2H), 4.53 (s, 2H), 4.19 – 4.15 (m, 2H), 4.08 (t, *J* = 7.5 Hz, 2H), 3.82 (t, *J* = 5.7 Hz, 2H), 3.66 – 3.61 (m, 5H), 3.52 (t, *J* = 5.4 Hz, 2H), 2.56 (t, *J* = 6.0 Hz, 2H), 2.20 (t, *J* = 7.2 Hz, 2H), 1.84 – 1.78 (m, 3H), 1.73 (s, 6H), 1.72 (s, 6H), 1.70 – 1.65 (m, 3H), 1.41 – 1.38 (m, 4H). ESI-MS (m/z): calculated for $C_{48}H_{59}N_7NaO_{15}S$ $[M-H]^-$: 1009.3958; found, 1009.3999.

Synthesis of **FITC-N$_3$**

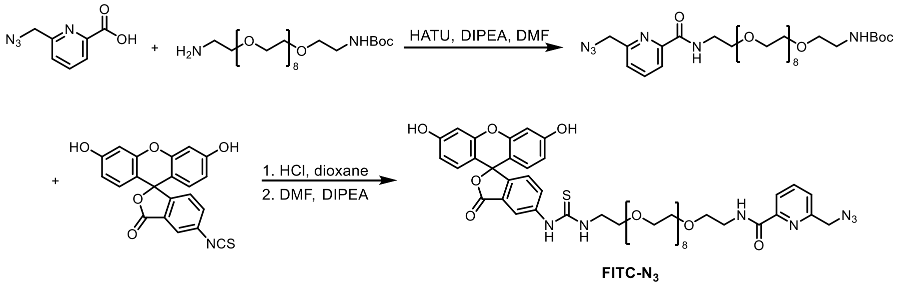

6-(azidomethyl)picolinic acid (50 mg, 0.28 mmol. 1.2 equiv.) was dissolved in DMF (1 mL), then HATU (175 mg, 0.46 mmol, 2.0 equiv.) and DIPEA (120 μL, 0.69 mmol. 3.0 equiv.) were added. The solution was stirred in room temperature for 12 hours. To above solution tert-butyl (29-amino-3,6,9,12,15,18,21,24,27-nonaoxanonacosyl)carbamate (130 mg, 0.23 mmol, 1.0 equiv.) was added. After 12 h, the DMF was removed under reduced pressure, and the residue was purified by flash chromatography (SiO_2, 10% MeOH in DCM) to yield a colorless oil (66 mg, 92 μmol, 40%). ^{1}H NMR (600 MHz, CD_3OD) δ 8.06 (d, *J* = 7.8, 1H), 8.00 (t, *J* = 7.5 Hz, 1H), 7.61 (d, *J* = 7.8, 1H), 4.56 (s, 2H), 3.69 (t, *J* = 5.4 Hz, 2H), 3.66 (s, 4H), 3.63– 3.59 (m, 30H), 3.50 (t, *J* = 5.4 Hz, 2H), 3.22 (q, *J* = 5.6 Hz, 2H), 1.43 (s, 9H). ^{13}C NMR (151 MHz, CD_3OD) δ 166.2, 158.4, 156.9, 150.7, 140.0, 126.2, 122.3, 80.0, 71.6, 71.59, 71.55, 71.5, 71.4, 71.3, 71.0, 70.6, 55.7, 41.3, 40.4, 28.8. ESI-MS (m/z): calculated for $C_{32}H_{57}N_6O_{12}$ $[M+H]^+$: 717.4029; found,717.4010 .

For Boc removal, tert-butyl tert-butyl (1-(6-(azidomethyl)pyridin-2-yl)-1-oxo-5,8,11,14,17,20,23,26,29-nonaoxa-2-azahentriacontan-31-yl)carbamate (20 mg, 28 μmol) was stirred in 4 M HCl in dioxane (1 mL) at room temperature. After 30 min, the solvent was evaporated under reduced pressure to give the hydrochloride. For conjugation, *N*-(29-amino-3,6,9,12,15,18,21,24,27-nonaoxanonacosyl)-6-(azidomethyl)picolinamide hydrochloride and 3',6'-dihydroxy-5-isothiocyanato-3H-spiro[isobenzofuran-1,9'-xanthen]-3-one (13 mg, 34 μmmol, 1.2 equiv.) were dissolved in DMF (2 mL), then DIPEA (10 μL, 56 μmmol, 2.0 equiv.) was added. The mixture was stirred at room temperature for 24 hours. The DMF was removed under reduced pressure, and the residue was purified by RP-HPLC to give **FITC-N$_3$** (12 mg, 12 μmmol, 44%). 3682. ^{1}H NMR (600 MHz, CD_3OD) δ 8.17 (s, 1H), 8.04 (d, *J* = 7.8 Hz, 1H), 7.98 (t, *J* = 7.8 Hz, 1H), 7.80 (d, *J* = 8.4 Hz, 1H), 7.58 (d, *J* = 7.2 Hz, 1H), 7.16 (d, *J* = 8.4 Hz, 1H), 6.73 (d, *J* = 8.4 Hz, 2H), 6.67 (d, *J* = 2.4 Hz, 2H), 6.57 –6.55 (m, 2H), 4.54 (s, 2H), 3.72 (t, *J* = 5.1 Hz, 2H), 3.67 –3.65 (m, 8H), 3.63 – 3.57 (m, 30H). ^{13}C NMR (151 MHz, CD_3OD) δ 182.7, 171.4, 166.2, 156.9, 155.0, 150.7, 142.4, 140.0, 130.8, 126.3, 126.2, 122.3, 120.4, 115.0, 112.3, 103.6, 71.52, 71.47, 71.45, 71.4, 70.6, 55.7, 45.5, 40.4. ESI-MS (m/z): calculated for $C_{48}H_{59}N_7NaO_{15}S$ $[M+H]^+$: 1006.3862; found, 1006.3832.

Synthesis of **Pyranine-tetrazine**

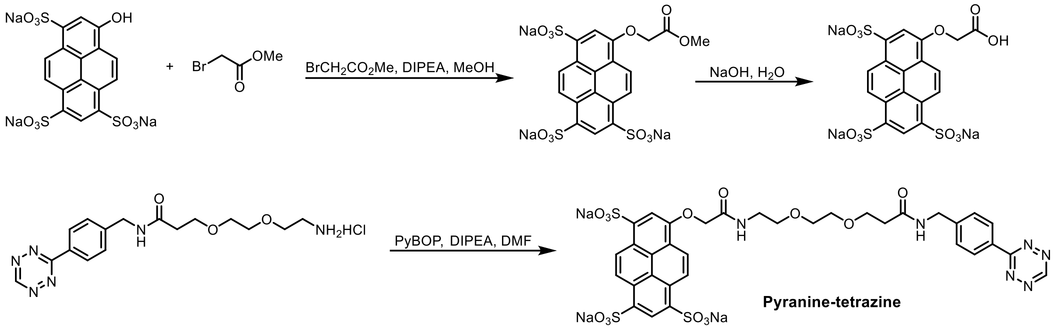

To a solution of sodium 8-hydroxypyrene-1,3,6-trisulfonate (105 mg, 0.20 mmol, 1.0 equiv.) in MeOH (5 mL) was added $BrCH_2CO_2Me$ (61 mg, 0.40 mmol, 2.0 eqiuv.) and DIPEA (70 μL, 0.40 mmol, 2.0 equiv.). The reaction mixture was stirred at 60 °C for 24 hours. The solvent was removed under reduced pressure, then DCM (5 mL) was added. The suspended mixture was centrifuged, liquid supernatant was removed, and the solid was collected to give sodium 8-(2-methoxy-2-oxoethoxy)pyrene-1,3,6-trisulfonate (85 mg, 0.14 mmol. 71%). ^{1}H NMR (600 MHz, DMSO-d_6) δ 9.14 (d, *J* = 9.6 Hz, 1H), 9.05 – 9.02 (m, 2H), 8.97 (d, *J* = 9.6 Hz, 1H), 8.42 (d, *J* = 9.6 Hz, 1H), 8.08 (s, 1H), 5.20 (s, 2H), 3.78 (s, 3H). ^{13}C NMR (151 MHz, DMSO-d_6) δ 169.3, 150.4, 143.1, 140.1, 139.8, 127.9, 127.5, 126.2, 125.5, 125.0, 124.5, 124.0, 121.1, 120.1, 119.8, 108.8, 65.6, 52.0.

Dissolved the sodium 8-(2-methoxy-2-oxoethoxy)pyrene-1,3,6-trisulfonate in the water (5 mL), NaOH (40 mg, 1.0 mmol, 5.0 equiv.) was added. The solution was stirred at 60 °C. After two hours, the solution was cooled to room temperature, 1mL of 1N $HCl_{(aq)}$ was added, the solvent was removed under reduced pressure. The residue was dissolved in the MeOH and filtered to obtain sodium 8-(carboxymethoxy)pyrene-1,3,6-trisulfonate (60 mg, 0.10 mmol, 50%). ^{1}H NMR (600 MHz, DMSO-d_6) δ 9.17 – 9.11 (m, 1H), 9.06 – 9.02 (m 2H), 9.00 – 8.95 (m, 1H), 8.45– 8.41 (m, 1H), 8.09 (s, 1H), 5.08 (s, 2H). ^{13}C NMR (151 MHz, DMSO-d_6) δ 170.6,

151.1, 143.5, 139.8, 128.4, 128.1, 126.8, 126.5, 125.9, 125.5, 125.0, 124.3, 121.3, 120.8, 120.1, 109.2, 65.9.

To a solution of sodium 8-(carboxymethoxy)pyrene-1,3,6-trisulfonate (20.4 mg, 0.035 mmol, 1.0 equiv.) in DMF (3 mL) was added *N*-(4-(1,2,4,5-tetrazin-3-yl)benzyl)-3-(2-(2-aminoethoxy)ethoxy)propanamide hydrochloride (13.5 mg, 0.035 mmol, 1.0 equiv., see synthesis of **Cy3-tetrazine** for its preparation), PyBOP (36.4 mg, 0.070 mmol, 2.0 equiv.) and DIPEA (12 μL, 0.070 mmol, 2.0 equiv.). The solution was stirred at room temperature. After 12 hours, the DMF was removed under reduced pressure, and the residue was purified by RP-HPLC to give **Pyranine-tetrazine** as a red solid (15 mg, 0.016 mmol, 45%). ^{1}H NMR (600 MHz, D_2O) δ 9.94 (s, 1H), 9.11 (s, 1H), 8.90 (d, *J* = 10.2 Hz, 1H), 8.80 (d, *J* = 9.6 Hz, 1H), 8.78 (d, *J* = 9.0 Hz, 1H), 8.33 (d, *J* = 9.6, 1H), 7.84 (s, 1H), 7.27 (d, *J* = 8.4, 2H), 6.65 (d, *J* = 7.8 Hz, 2H), 4.67 (s, 2H), 4.00 (s, 2H) 3.77 (t, *J* = 5.7 Hz, 2H), 3.68 – 3.62 (m, 6H), 3.48 (t, *J* = 5.1 Hz, 2H), 2.55 (t, *J* = 5.7 Hz, 2H). ^{13}C NMR (151 MHz, D_2O) δ 174.5, 170.1, 164.5, 156.7, 151.0, 142.4, 138.8, 135.0, 135.0, 129.5, 129.1, 128.0, 127.0, 126.6, 126.0, 125.1, 125.0, 124.6, 124.5, 123.5, 123.2, 120.9, 120.8, 109.3, 69.8, 69.6, 69.0, 67.1, 66.8, 42.2, 39.0, 36.0.

Synthesis of **BCN-NHS**

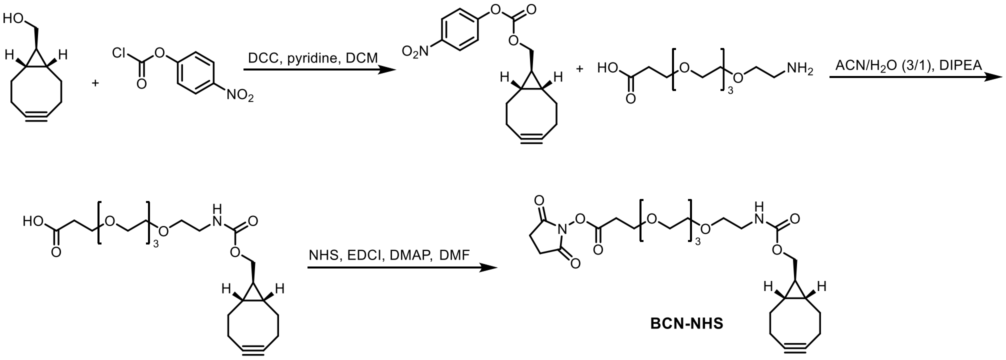

((1*R*,8*S*,9*r*)-bicyclo[6.1.0]non-4-yn-9-yl)methanol (150 mg, 1.0 mmol. 1.0 equiv.) and 4-nitrophenyl carbonochloridate (402 mg, 2.0 mmol, 2.0 equiv.) was dissolved in DCM (10 mL), then pyridine (160 μL, 2.0 mmol. 2.0 equiv.) were added. The solution was stirred in room temperature. After 24 hours, the DCM was removed under reduced pressure, and the residue was purified by flash chromatography (SiO_2, 10% EtOAc in Petroleum ether) to yield ((1*R*,8*S*,9*r*)-bicyclo[6.1.0]non-4-yn-9-yl)methyl (4-nitrophenyl) carbonate as a white solid (224 mg, 0.71 mmol, 71%). ^{1}H NMR (400 MHz, $CDCl_3$) δ 8.27 (d, *J* = 9.2 Hz, 2H), 7.38 (d, *J* = 8.8 Hz, 2H), 4.22 (d, *J* = 6.4 Hz, 2H), 2.47 – 2.43 (m, 2H), 2.34 – 2.26 (m, 2H), 2.21 – 2.15 (m, 2H), 1.46 – 1.36 (m, 2H), 0.87 – 0.80 (m, 3H). ^{13}C NMR (101 MHz, $CDCl_3$) δ 155.7, 152.7, 145.5, 125.4, 121.9, 98.8, 74.1, 33.2, 23.4, 23.1, 21.4.

To a solution of 1-amino-3,6,9,12-tetraoxapentadecan-15-oic acid (236 mg, 0.75 mmol, 1.5 equiv.) in acetonitrile (6 mL) and water (2 mL) was added ((1*R*,8*S*,9*r*)-bicyclo[6.1.0]non-4-yn-9-yl)methyl (4-nitrophenyl) carbonate (158 mg, 0.5 mmol, 1.0 equiv.) and DIPEA (2.0 equiv.). After 24 hours, the solvent were removed under reduced pressure, and the residue was purified by flash chromatography (SiO_2, 3% MeOH in DCM) to yield 1-((1*R*,8*S*,9*r*)-bicyclo[6.1.0]non-4-yn-9-yl)-3-oxo-2,7,10,13,16-pentaoxa-4-azanonadecan-19-oic acid as a colorless oil (88 mg, 0.20 mmol, 39%). ^{1}H NMR (400 MHz, CD_3OD) δ 3.95 (d, *J* = 8.8 Hz, 2H), 3.73 (t, *J* = 6.2 Hz, 2H), 3.65 – 3.60 (m, 12H), 3.53 (t, *J* = 5.6 Hz, 2H), 3.28 (t, *J* = 5.6 Hz, 2H), 2.55 (t, *J* = 6.2 Hz, 2H), 2.41 – 2.35 (m, 2H), 2.29 – 2.20 (m, 2H), 2.13 – 2.07 (m, 2H), 1.43 – 1.33 (m, 2H), 0.79 –

0.64 (m, 3H). ^{13}C NMR (101 MHz, CD_3OD) δ 159.3, 99.4, 71.57, 71.56, 71.55, 71.5, 71.4, 71.3, 71.0, 70.0, 67.9, 41.7, 36.0, 34.4, 25.0, 24.2, 21.9. ESI-HRMS (m/z): calculated for $C_{22}H_{34}NO_8$ $[M-H]^-$: 440.2290; found, 440.2293.

To a solution of 1-((1*R*,8*S*,9*r*)-bicyclo[6.1.0]non-4-yn-9-yl)-3-oxo-2,7,10,13,16-pentaoxa-4-azanonadecan-19-oic acid (88 mg, 0.20 mmol, 1.0 equiv.) in DMF (5 mL) was added EDCI (77 mg, 0.40 mmol, 2.0 equiv.), DMAP (5.0 mg, 0.04 mmol, 0.2 equiv.) and NHS (28 mg, 0.24 mmol, 1.2 equiv.). The solution was stirred at room temperature. After 12 hours, water (30 mL) was added, and the mixture was extracted with EtOAc (3 × 20 mL). The combined organic phases were washed with brine (3 × 20 mL), dried over Na_2SO_4, filtered, and concentrated to yield **BCN-NHS** as a colorless oil (97 mg, 0.18 mmol, 90%) without further purification. ^1H NMR (600 MHz, $CDCl_3$) δ 5.27 (s, 1H), 3.95 (d, *J* = 6.6 Hz, 2H), 3.83 (t, *J* = 6.6 Hz, 2H), 3.65 – 3.59 (m, 12H), 3.54 (t, *J* = 5.1 Hz, 2H), 3.36 – 3.33 (m, 2H), 2.88 (t, *J* = 6.3 Hz, 2H), 2.82 (s, 4H), 2.40 – 2.36 (m, 2H), 2.29 – 2.23 (m, 2H), 2.15 – 2.11 (m, 2H), 1.38 – 1.31 (m, 2H), 0.73 – 0.65 (m, 3H). ^{13}C NMR (151 MHz, $CDCl_3$) δ 169.1, 166.8, 157.0, 98.9, 70.8, 70.7, 70.7, 70.6, 70.58, 70.4, 70.2, 69.1, 65.8, 40.9, 33.4, 32.2, 25.7, 23.9, 22.9, 21.5. ESI-HRMS (m/z): calculated for $C_{26}H_{39}N_2O_{10}$ $[M+H]^+$: 539.2599; found, 539.2596.

Synthesis of **Tetrazine-cysteine**

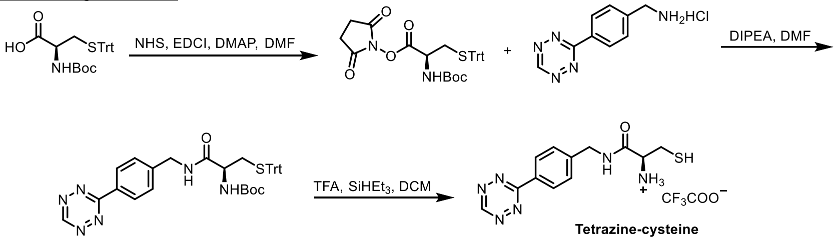

To a solution of *N*-(tert-butoxycarbonyl)-S-trityl-D-cysteine (464 mg, 1.0 mmol, 1.0 equiv.) in DMF (10 mL) was added EDCI (383 mg, 2.0 mmol, 2.0 equiv.), DMAP (24 mg, 0.2 mmol, 0.2 equiv.) and NHS (173 mg, 1.5 mmol, 1.5 equiv.). The solution was stirred at room temperature. After 24 hours, water (30 mL) was added, and the mixture was extracted with EtOAc (3 × 40 mL). The combined organic phases were washed with brine (3 × 20 mL), dried over Na_2SO_4, filtered, and concentrated. The residue was purified by flash chromatography (SiO_2, 1% MeOH in DCM) to yield 2,5-dioxopyrrolidin-1-yl *N*-(tert-butoxycarbonyl)-S-trityl-D-cysteinate as a white solid (432 mg, 0.77 mmol, 77%). ^1H NMR (400 MHz, $CDCl_3$) δ 7.45 – 7.44 (m, 6H), 7.32 – 7.28 (m, 6H), 7.24 – 7.20 (m, 3H), 4.87 (d, *J* = 8.4 Hz, 1H), 4.36 – 4.31 (m, 1H), 2.77 – 2.75 (m, 1H), 2.71 – 7.66 (m, 1H), 1.43 (s, 9H). ^{13}C NMR (101 MHz, $CDCl_3$) δ 168.4, 167.0, 154.6, 144.2, 129.7, 128.3, 127.1, 80.8, 67.6, 51.3, 33.8, 28.4, 25.7.

To a solution of 2,5-dioxopyrrolidin-1-yl *N*-(tert-butoxycarbonyl)-S-trityl-D-cysteinate (100 mg, 0.18 mmol, 1.0 equiv.) and (4-(1,2,4,5-tetrazin-3-yl)phenyl)methanamine hydrochloride (40 mg, 0.18 mmol, 1.0 equiv.) in DMF (2 mL) was added DIPEA (63 μL, 0.36 mmol, 2.0 equiv.). The solution was stirred at room temperature. After 16 hours, water (30 mL) was added, and the mixture was extracted with EtOAc (3 × 20 mL). The combined organic phases were washed with brine (3 × 20 mL), dried over Na_2SO_4, filtered, and concentrated. The residue was purified by flash chromatography (SiO_2, 10% EtOAc in Petroleum ether) to yield tert-butyl (*S*)-(1-((4-(1,2,4,5-tetrazin-3-yl)benzyl)amino)-1-oxo-3-(tritylthio)propan-2-yl)carbamate as a red solid (76 mg, 0.12 mmol, 67%). ^1H NMR (400 MHz, $CDCl_3$) δ 10.20 (s, 1H), 8.53 (d, *J* = 8.0 Hz, 2H), 7.47 – 7.41 (m, 8H), 7.31 – 7.26 (m, 6H), 7.23 – 7.20 (m, 3H), 6.59 (s, 1H), 4.88 (s, 1H), 4.51 (d, *J* = 6.4 Hz, 2H), 3.94 – 3.89 (m, 1H), 2.82 – 2.77 (m, 1H), 2.61 – 2.56 (m, 1H), 1.40 (s, 9H). ^{13}C

NMR (101 MHz, $CDCl_3$) δ 170.8, 166.4, 157.9, 155.6, 144.5, 143.7, 130.8, 129.7, 128.7, 128.4, 128.2, 127.1, 80.6, 67.4, 53.8, 43.2, 33.7, 28.4. ESI-HRMS (m/z): calculated for $C_{36}H_{37}N_6O_5S$ $[M+Na]^+$: 655.2462; found, 655.2442.

To a solution of tert-butyl (*S*)-(1-((4-(1,2,4,5-tetrazin-3-yl)benzyl)amino)-1-oxo-3-(tritylthio)propan-2-yl)carbamate (32 mg, 0.05 mmol, 1.0 equiv.) in DCM (3 mL) cooled to 0 ℃ was added TFA (3 mL) and triethyl silane (40 μL, 0.25 mmol, 5.0 equiv.). The reaction mixture was stirred at room temperature. After two hours, TLC confirmed full conversion. The solvent was removed under reduced pressure, and the residue was dried to ogive **Tetrazine-cysteine** as a red solid (19 mg, 0.047 mmol, 95%) without further purification. ESI-HRMS (m/z): calculated for $C_{36}H_{37}N_6O_5S$ $[M+H]^+$: 291.1023; found, 291.1026.

Kinetic studies

Solutions of TAMM and 1,2-aminothiol derivatives (typically 100 and 50 μM, respectively, unless otherwise specified) containing tris(2-carboxyethyl)phosphine (TCEP, 400 μM) in phosphate buffer (0.1 M, typically pH 7.4) were mixed from the freshly prepared stock solutions at the specified temperatures, maintained using Peltier-controlled thermostat (Agilent G1330B). The reaction progress was monitored by HPLC (Agilent 1260 system, column = 5 μm C18 110 Å 250 x 4.6 mm, mobile phase = water and MeCN containing 0.1% v/v formic acid, flow rate = 0.3 mL/min). Concentrations of the individual components were calculated from the integrated peak areas of the chromatogram using the calibration curves. The rate constants and activation parameters were determined by non-linear fitting of the experimental data to the appropriate equations using software OriginPro (OriginLab). A minimum of three experimental replicates was performed for each condition, and the average value is presented with the standard deviation indicating the experimental error.

For TAMM molecules, of which reaction with 1,2-aminothiol leads to formation of two intermediates, rate constants were determined through competition experiments with (3-acetoxy-2-formylphenyl)boronic acid (**KL42**) as the reference compound at pH 6.0. Given that both TAMM derivatives and **KL42** exhibit specific reactivity towards 1,2-aminothiol, the rate constant (k_{obs}) for TAMM can be approximated by comparing the product ratio at extended reaction time when all 1,2-aminothiol molecules are consumed. We avoided determining the relative rate of different TAMM molecules through direct competition. The leaving group from the slow TAMM molecule can undergo thiol-exchange with the fast TAMM molecule. This is why we used KL42 that reacted with 1,2-aminothiol under a different mechanism for competition experiments.

We first used TAMM **1b** to determine the relative rate constant of **KL42** (**Fig. S15a**). The experiment was set up in potassium phosphate buffer (0.1 M, pH 6) with initial concentrations of the starting materials as [**2x**]$_0$ = 50 μM, [**1b**]$_0$ = 200, 300 or 400 μM, and [**KL42**]$_0$ = 100 μM. Using $k_{obs}^{7.4}$ of **1b** as 25 M^{-1} s^{-1}, the corresponding $k_{obs}^{7.4}$ of **KL42** was obtained by aligning the numerically simulated product ratio to [**3bx**]/[**p-KL42**]. Numerical simulation of the product ratio was done using the Python script (see Supplementary Text). The product ratios from the competition of 200, 300 or 400 μM **1b** with 100 μM **KL42** were found to be 0.0720, 0.105 and 0.136, respectively. We assumed the relative rate between **1b** and **KL42** as pH independent. The pH value affects the thiolate (S^-) concentration in the reaction mixture. Based on the mechanisms of the two reactions, the key steps of the two reactions would be equally accelerated with the rising pH value, so their relative rate remains unchanged. Consequently, the $k_{obs}^{7.4}$ of **KL42** was

estimated to be 919, 935 and 957 M^{-1} s^{-1}. The average value (937 M^{-1} s^{-1}) was used for subsequent competition experiments between **KL42** and TAMM **1i-1n** (**Fig. S15b-g**).

Competition experiments of **KL42** and TAMM **1i-1m** were performed in PBS (0.1 M, pH 6) with initial concentrations of the starting materials as $[\mathbf{2x}]_0$ = 50 µM, $[\mathbf{1}]_0$ = 100 µM, and $[\mathbf{KL42}]_0$ = 100, 150 or 200 µM. Competition experiments of **KL42** and TAMM **1n** were performed in potassium phosphate buffer (0.1 M, pH 6) with initial concentrations of the starting materials as $[\mathbf{2x}]_0$ = 50 µM, $[\mathbf{1n}]_0$ = 100 µM, and $[\mathbf{KL42}]_0$ = 600, 900 or 1200 µM. The corresponding $k_{obs}^{7.4}$ was obtained by aligning the numerically simulated product ratio to [**3**]/[**p-KL42**] as described above.

The order of the reaction and the observed rate constant

The reaction order in TAMM **1a** was determined at 25 °C by comparing the consumption rate of **1a** at $[\mathbf{1a}]_0$ = 70, 100, or 150 µM in the presence of peptide $[\mathbf{2x}]_{\mathrm{tot},0}$ = 50 µM and TCEP (400 µM) in 0.1 M potassium phosphate buffer at pH 7.4 (subscription 0 indicates the concentration at t = 0 min). Similarly, the reaction order in peptide **2x** was determined by comparing the consumption rate of **2x** at $[\mathbf{2x}]_{\mathrm{tot},0}$ = 30, 50, or 80 µM in the presence of TAMM $[\mathbf{1a}]_0$ = 100 µM under the same condition. As only one HPLC peak was found for **2x** in chromatograms, the concentration of **2x** determined by HPLC should represent the total concentration of its various ionization (protonation or deprotonation) forms that may be present in the aqueous solution. Hence, a subscription "tot" is added to the symbol of its concentration as $[\mathbf{2x}]_{\mathrm{tot}}$.

The 'initial' rate of consumption for each experiment was calculated as the Δ[species]/Δt from the first two earliest data points and shown in **Table S1** and **Figure S1**. It was found that the rate increases linearly with respect to concentration changes for both $[\mathbf{1a}]_0$ and $[\mathbf{2x}]_{\mathrm{tot},0}$, suggesting first order in both reactants. Therefore, the reactant consumption rate law for TAMM derivatives **1** and peptide **2x** can be described by **eq S1**, which is further elaborated in the next section.

$$rate = \frac{d[\mathbf{1}]}{dt} = \frac{d[\mathbf{2x}]_{\mathrm{tot}}}{dt} = -k_{\mathrm{obs}}[\mathbf{1}][\mathbf{2x}]_{\mathrm{tot}} \qquad \text{(S1)}$$

Concentrations of all species involved in the reaction at various time points were determined by HPLC from the area under the respective peaks in chromatograms using appropriate calibration curves. The time-evolution of the concentration data was then fit to the model of **eq S1** to estimate the observed rate constant k_{obs}; a representative fit example is shown in **Figure 1D**.

Estimation of the pKa of the thiol group in peptide **2x**

By defining alpha (α) as the ratio of the ionized form ($\mathbf{2x}^{\mathrm{S-}}$) to the neutral form ($\mathbf{2x}^{\mathrm{SH}}$) for the cysteine residue in peptide CGGGKGW (**2x**):

$$\alpha = \frac{[\mathbf{2x}^{\mathrm{S-}}]}{[\mathbf{2x}^{\mathrm{SH}}]} \tag{S2}$$

, the concentration of the ionized form, i.e., the active nucleophile of the reaction, can be expressed in terms of α and the total concentration of **2x** ($[\mathbf{2x}]_{\mathrm{tot}}$, the measurable quantity by HPLC)

$$[\mathbf{2x}]_{\mathrm{tot}} = [\mathbf{2x}^{\mathrm{SH}}] + [\mathbf{2x}^{\mathrm{S-}}] \tag{S3}$$

$$[\mathbf{2x}^{\mathrm{S-}}] = \frac{\alpha}{\alpha+1}\left([\mathbf{2x}^{\mathrm{S-}}] + [\mathbf{2x}^{\mathrm{SH}}]\right) = \frac{\alpha}{\alpha+1}[\mathbf{2x}]_{\mathrm{tot}} \tag{S4}$$

Since the extent of ionization is determined by the proton concentration of the solution ($[\mathrm{H}^+]$) and the acid dissociation constant (K_a) for the equilibrium $\mathbf{2x}^{\mathrm{SH}} \rightleftharpoons \mathbf{2x}^{\mathrm{S-}} + \mathrm{H}^+$, α can also be related to these quantities by

$$K_a = \frac{[\mathbf{2x}^{\mathrm{S-}}][\mathrm{H}^+]}{[\mathbf{2x}^{\mathrm{SH}}]} = \alpha[\mathrm{H}^+] \tag{S5}$$

Combining **eqs S4** and **S5** into the rate expression of the TAMM reaction, one can derive

$$rate = -k[\mathbf{1}][\mathbf{2x}^{\mathrm{S-}}] = -k\frac{\alpha}{\alpha+1}[\mathbf{1}][\mathbf{2x}]_{\mathrm{tot}} = -k\frac{K_a}{K_a+[\mathrm{H}^+]}[\mathbf{1}][\mathbf{2x}]_{\mathrm{tot}} = -k_{\mathrm{obs}}[\mathbf{1}][\mathbf{2x}]_{\mathrm{tot}} \tag{S6}$$

, where the observed rate constant k_{obs} is related to the pH-independent rate constant k by

$$k_{\mathrm{obs}} = k\frac{K_a}{K_a+[\mathrm{H}^+]} = k\frac{1}{1+10^{(pK_\mathrm{a}-p\mathrm{H})}} \tag{S7}$$

The pK_a of the thiol group in peptide **2x** can therefore be determined by taking the ratio of the k_{obs} at two different pH values. Specifically, the observed rate constants for **1a** and **2x** were found to be $k_{\mathrm{obs}}^{6.0} = 0.25 \pm 0.02$ M^{-1} s^{-1} and $k_{\mathrm{obs}}^{7.4} = 5.31 \pm 0.22$ M^{-1} s^{-1} at pH = 6.0 and 7.4, respectively. Plugging these values to **eq S7** gives pK_a = 8.13 for the cysteine residue in **2x**. Such a pK_a value was used subsequently to calculate k from k_{obs} through **eq S7**.

Activation parameters of the reaction between **1a** and **2x**

Rate constants for the reaction between **1a** and **2x** at pH 7.4 were measured at various temperatures between 5 to 40 °C. Nonlinear fit of these data to the Eyring equation $k = (k_B T/h)\exp(-\Delta G^{\ddagger}/RT)$ yielded the estimated Gibbs energy of activation $\Delta G^{\ddagger}$ = 15.43 ± 0.12 kcal mol^{-1} (with $\Delta H^{\ddagger}$ = 12.87 ± 0.06 kcal mol^{-1} and $\Delta S^{\ddagger}$ = –8.60 ± 0.20 cal mol^{-1} K^{-1}; **Figure S2a**). A similar analysis using the Arrhenius equation $k = A\exp(-E_a/RT)$ gave activation energy E_a = 13.45 ± 0.06 kcal mol^{-1} (**Figure S2b**).

Rate constants and activation parameters for the conversion of **Int-1** (**7**) to **3**

Rate constants for the conversion of **Int-1** (**7**) → **3** at pH 7.4 were estimated using disulfide **9ay** as the starting material at various temperatures between 5 to 40 °C. TCEP was used to reduce **9ay** into **Int-1** (**7ay**) in situ. Monitoring of the reaction commenced 10 minutes post-TCEP addition, ensuring complete conversion of **9ay** to **7ay**. The time evolution of concentration (such as the plot shown in **Figure 1G**) was fitted to a first-order kinetic model to derive the rate constants at specific temperatures. Eyring and Arrhenius analyses yielded $\Delta G^{\ddagger}$ = 21.80 ± 0.39 kcal mol^{-1} and E_a = 18.46 ± 0.20 kcal mol^{-1}, respectively (**Figure S12a**/**b**).

This Gibbs energy of activation, 21.80 kcal mol^{-1}, is close to the calculated free energy barrier of D$G^{\ddagger}_{calc}$ =19.91 kcal mol^{-1} for the step of malononitrile elimination (**Figure S13**), involving an ion-pairing, late transition state.

The rate constant (as opposed to the reaction rate) for the dihydrothiazole formation step can be alternatively estimated for some reactions. For instance, in the reaction **1a** + **2x** → **3ax**, fitting the consumptions data of **1a** or **2x** to $d[\mathbf{1a}]/dt = -k^{\mathrm{bi}}[\mathbf{1a}][\mathbf{2x}]$ and $d[\mathbf{2x}]/dt = -k^{\mathrm{bi}}[\mathbf{1a}][\mathbf{2x}]$ and the formation of **3ax** to $d[\mathbf{3ax}]/dt = +k^{\mathrm{uni}}\{[\mathbf{2x}]_0 - [\mathbf{2x}] - [\mathbf{3ax}]\}$ yielded k^{bi} = 5.1 ± 0.1 M^{-1} s^{-1} and k^{uni} = 0.0028 ± 0.0005 s^{-1} (**Figure S12c**). The bimolecular rate constant is consistent with the value reported in **Figure S3**, where only the consumption of the starting materials was considered. The unimolecular rate constant is slightly larger than the rate constant estimated from the reduction of disulfide dimer (**Figure S12a**/**b**); this small discrepancy likely arises the structural difference in the aminothiol moiety (terminal cysteine of an oligopeptide vs cysteine). However, such analysis is not feasible for fast reactions where Int-2 forms rapidly. Instead, a parameter T_{50}—analogous to a half-life—could be considered to assess the speed of product formation. T_{50} is defined as the time required for the product concentration to reach 50% of its maximum value. Under the condition where [TAMM]$_0$ = 100 μM (TAMM = **1a**, **1i**, or **1j**), [**2x**]$_0$ = 50 μM, T_{50} falls between 1021–2435 s (**Figure S12d**); while the same product (**3ax**) is formed for these reactions, T_{50} appears to be smaller for reactions involving faster TAMM reactants.

However, it is worth pointing out that due to the involvement of multiple reaction steps, including a bimolecular reaction at the beginning, the T_{50} parameter is strongly dependent on the concentration of the reactants. This dependence can be illustrated using a model A + B → C → D reaction in **Figure S12e**. Significant differences in T_{50} are observed despite the identical rate constants for these two steps. Furthermore, as shown in **Figure S12f**, reactions with vastly different k^{uni} values (the rate constant most directly associated with dihydrothiazole formation) can exhibit similar T_{50}, as T_{50} is more influenced by the initial bimolecular step than by subsequent steps. Given these complexities, T_{50} does not provide a reliable or meaningful measure of the intrinsic kinetics of product formation. Instead, given that peptides can be considered "labeled" immediately after the initial bimolecular reaction and that the intermediates

remain unaffected by other aminothiol compounds in solution, we propose that the efficacy of TAMM labeling should be evaluated primarily based on the initial bimolecular rate constants.

Culture and transfection of cell lines

HEK293T, HeLa, MCF7, ND7/23 and SK-OV-3 cells were cultured in complete media (i.e., 10% FBS in DMEM) containing 1% penicillin-streptomycin (Solarbio, #P1400) and maintained in a 5% CO_2 atmosphere at 37 °C. For transient transfection, cells were seeded to a 24-well plate with 3×10^5 cells (for HEK293T) or 8×10^4 cells (for HeLa, MCF7, ND7/23 and SK-OV-3) per well. The plate was pre-treated with 0.1 mg/mL poly-L-lysine (Sangon, #E607015) in sterile water and washed with PBS (Meilunbio, #PWL050). After 24 hours, transfection was conducted using PolyJet (SignaGen, #SL100688) or Lipofectamine 3000 (ThermoFisher, #L3000015) following the manufacturers' protocol. After 8 h, culture media was replaced with the complete media.

- For **CysK** incorporation, cells were co-transfected with *Mm*PylRS/tRNA$_{CUA}$ and cultured in the presence of 1 mM **CysK-OMe** for 48 h. **CysK-OMe** was synthesized according to Reference 28 in the manuscript. Stock solution of CysK-OMe was prepared by dissolving it in sterile water to the final concentration of 100 mM.
- For **BCNK** incorporation, cells were co-transfected with *Mm*PylRS* and Pyl-tRNA$_{CUA}$ or Pyl-tRNA$_{UCCU}$. Cells were then cultured in the presence of 0.1 mM **BCNK** for 48 h. Stock solution of **BCNK** was prepared by dissolving **BCNK** (SiChem, #SC-8014) in 0.1 N NaOH to the final concentration of 100 mM.
- For **PPA** incorporation, cells were co-transfected with *Ec*TyrRS*/tRNA$_{UCA}$ and cultured in the presence of 1 mM **PPA** for 48 h. Stock solution of **PPA** was prepared by dissolving **PPA** (Bidepharm, #BD00784779) in 0.1 N NaOH to the final concentration of 100 mM.
- For **HexC** incorporation, cells were co-transfected with *Ec*LeuRS*/tRNA$_{CUA}$ and cultured in the presence of 0.1 mM **HexC** for 48 h. Stock solution of **HexC** was prepared by dissolving **HexC** (synthesized according to Reference 33 in the manuscript) in sterile water to the final concentration of 100 mM.

Culture and transfection of primary neurons

Primary mouse cortical neurons were obtained from P0 C57BL/6 mice. In general, pups were sacrificed, and cortex intermediately dissected into a cold 1X HBSS solution (Gibco, #14175079). Cells were then digested with 0.25% trypsin (Gibco, #2520072) and plated onto poly-D-lysine (0.1 mg/mL, Sigma, #P7405) coated glass (Citoglas, #1210008CE) and cultured with DMEM (Gibco, #11995065) containing with 10% F12 (Gibco, #11765054) and 10% FBS (Gibco, #10099141) for seeding. After 24 h, half of the media were changed with neurobasal medium containing 2% B27(Gibco, #17504044) and 1% GlutaMax (Gibco, #35050061). Before glia density showing robust increase (usually, 4 days after plating), 0.2% CytoA (Sigma, #251010) was added into the culture medium. Half changing culture medium was performed every 3-5 days in the following culture periods. Neurons at DIV4 or DIV5 (DIV, days in vitro) were transfected with Lipofectamine 2000 (Invitrogen, #11668019) following the manufacturer's protocol. After 2 h, the medium was replaced with the normal culture medium.

Cell viability assay

HEK293T cells were seeded at a density of 5×10^4 cells per well in a 96-well plate. After 24 hours, culture media was changed, and cells in each well were incubated with 100 μL of indicated concentration of malononitrile in LCIS. After one hour at 37 °C, the solution was replaced with a mixture of 10 μL of CCK-8 (GLPBIO, #GK10001) and 90 μL of the complete media. After three hours at 37 °C, cells were analyzed on a BioTek Synergy Neo2 microplate reader. Each data point was calculated from at least three replicates. Values from cell-only (i.e., [malononitrile] = 0) wells were set as 100% viability.

Protein fluorescent labeling

- For labeling with **Cy5-TAMM-SEt**, cells were typically treated with 2 μM of the conjugate and 2 mM DTT in live cell imaging solution (LCIS) for 60 minutes at 37 °C. LCIS contains 140 mM NaCl, 2.5 mM KCl, 1.8 mM $CaCl_2$, 1.0 mM $MgCl_2$ and 20 mM HEPES at pH 7.4 and is used after sterile filtration. For labeling with ultrafast TAMM conjugates (e.g., **Cy5-TAMM-SCH$_2$CF$_3$**), cells were typically treated with 2 μM of the conjugate and 0.5 mM TCEP in LCIS for 10–30 minutes at 37 °C. After TAMM labeling, cells were washed with 1 mM cysteine in the complete media for 15 minutes at 37 °C.
- For TAMM condensation in primary neuronal cell culture, labeling was performed in Tyrode's solution (100 mM NaCl, 5 mM $MgCl_2$, 2 mM $CaCl_2$, 15 mM D-glucose, 10 mM HEPES at pH 7.4) instead of LCIS.
- For labeling two targets by sequential TAMM condensation, cells were labeled with 2 μM of the first TAMM conjugate in LCIS containing 0.5 mM TCEP for 30 minutes at 37 °C. Cells were then washed with 1 mM cysteine in the complete media for 15 min. Proteolytic cleavage was performed with 10 ng/μL Factor Xa protease (NEB, #P8010L) in LCIS for 10 minutes at 37 °C. Cells were then treated with 2 μM of the second TAMM conjugate in LCIS containing 0.5 mM TCEP for 10 minutes at 37 °C. For labeling of two targets on two cell populations, cells were treated with 0.4 mM **PEG-TAMM-SCH$_2$CF$_3$** in LCIS containing 0.5 mM TCEP for 30 minutes at 37 °C to quench any unreacted 1,2-aminothiol groups before proteolytic cleavage.
- For labeling three targets by sequential TAMM condensation, cells expressing **CysK**-HA-Nlgn3 were labeled with 2 μM **Bodipy-TAMM-SCH$_2$CF$_3$** in LCIS containing 0.5 mM TCEP for 10 minutes at 37 °C. Cells were then subjected to a "wash-quench-wash" cycle through incubation with 1 mM cysteine in complete media for 5 min, 0.4 mM **PEG-TAMM-SCH$_2$CF$_3$** in LCIS containing 0.5 mM TCEP for 30 minutes at 37 °C, and 1 mM cysteine in complete media for 5 min. Labeling of cells expressing IDGR-C-HA-Nlgn3 began with proteolytic cleavage by 10 ng/μL Factor Xa protease in LCIS for 10 minutes at 37 °C. Cells were then labeled with 2 μM **Cy5-TAMM-SCH$_2$CF$_3$** in LCIS containing 0.5 mM TCEP for 10 minutes at 37 °C, followed by a wash-quench-wash cycle. Labeling of cells expressing DDDDK-C-HA-Nlgn3 began with proteolytic cleavage by 0.0375 IU/μL enterokinase (Sangon, #C600411-0100) in LCIS for 30 minutes at 37 °C. Cells were then labeled with 2 μM **Cy3-TAMM-SCH$_2$CF$_3$** in LCIS containing 0.5 mM TCEP for 10 minutes at 37 °C, followed incubation with 1 mM cysteine in complete media for 5 min.
- For CuAAC, a solution of $CuSO_4$ (1 mM, 20 μL), ddH_2O (61 μL) and BTTAA (1 mM, 100 μL) was mixed. After one minute, PBS (20x, 10 μL), **FITC-N$_3$** (100 μM, 4 μL), and L-ascorbic acid sodium salt (100 mM, 5 μL) were added to afford the labeling solution (total

volume = 200 μL). Cells were then incubated with this labeling solution for 10 minutes at 37 °C. Afterwards, cells were washed with PBS for two times (10 minutes each).
- For tetrazine ligation, cells were incubated with 2 μM **Cy3-tetrazine** in LCIS for 10 minutes at 37 °C. Afterwards, cells were washed with LCIS for two times.

It is noteworthy that TAMM condensation and tetrazine ligation can be performed at the same time, minimizing handling time.

Confocal imaging

After 24-h post-transfection, around 3×10^6 cells were transferred to an imaging dish (Cellvis, # P24-1.5H-N) pre-treated with 0.1 mg/mL poly-L-lysine. After 24 h, cells were washed with LCIS. After labeling, cells were cultured in the complete media and imaged on a Nikon A1 confocal laser scanning microscope.

- For immunofluorescent staining of cell lines, after TAMM labeling, cells were fixed with 4% paraformaldehyde for 10 minutes at room temperature and washed with PBS for three times. Cells were then treated with 5% BSA in PBS for one hour. Immunofluorescence against HA was achieved using mouse anti HA-antibody (1:1000, ABclonal, #AE008,) in PBS containing 1% BSA for overnight at 4 °C and goat goat anti-mouse IgG DyLight 488 (1 μg/mL, ThermoFisher, #35502) in PBS containing 1% BSA for 1 hour at the room temperature.
- For immunofluorescent staining of primary neurons, fixed neurons were permeabilized and blocked by 0.3% Triton X-100 and 5% BSA. Immunostaining was achieved with primary antibodies against HA (1:1000, rabbit, CST, #C29F4) and MAP2 (1:1000, chicken, Abcam, #ab5392) overnight at 4°C. Cells were then washed with PBS for three times and incubated with the secondary antibodies containing Alex405 (1:1000, anti-rabbit, ThermoFisher, #A31556) and Alex488 (1:1000, anti-chicken, ThermoFisher, #A21103).

In-gel fluorescent analysis

At 48-h post-transfection, cells were washed with LCIS. After labeling, cells in each well were lysed with 50 μL ice-cold RIPA (Sigma, #R0278) containing 1×protease inhibitor (EpiZyme, #GRF101) on ice. After 10 min, cell lysates were collected and centrifuged at 15000 rcf for 10 minutes at 4 °C. The supernatant (39 μL) was mixed with loading dye (13 μL, Invirogen, #NP0008) and heated at 95 °C for 10 minutes. Samples were then electrophoresed on a 4–20% SDS-PAGE (NoninBio, #ET12420Gel) at 100 V for 90 minutes. Fluorescnece imaging was performed using a Bio-Rad ChemiDoc MP system.

Immunoblotting

Proteins on a gel were transferred to a nitrocellulose membrane (Merck, # HATF00010). Blocking and antibody incubation were performed in TBST (Solarbio, #T1081) containing 5% (w/v) skimmed milk powder. HA and GAPDH were detected by mouse anti-HA antibody (1:1000, ABclonal, #AE008) and mouse anti-GAPDH antibody (1:1000, ABclonal, #AC002), respectively. HRP-conjugated goat anti-mouse antibody (1:20000, ABclonal, #AS003,) was used as the secondary antibody. Visualization was achieved using chemiluminescence substrate (UElandy, #S6009M) on a Bio-Rad ChemiDoc MP system.

Fluorescent labeling in living mice

All protocols were approved by the Institutional Animal Care and Use Committee of Shenzhen Bay Lab. NCG mice (6 – 8 week) were subcutaneously grafted with 1×10^7 HEK293T cells functionalized with 1,2-aminothiol (293T-Cys) or bicyclo[6.1.0]non-4-yne (293T-BCN) on the left or right rear limb of each mouse, respectively. 293T-BCN was prepared by incubating HEK293T cells with 100 μM **BCN-NHS** in LCIS at 37 °C for 30 minutes. 293T-Cys was prepared by incubating 293T-BCN cells with 100 μM **tetrazine-Cys** in LCIS at 37 °C for 30 minutes. Mice were then subjected to intraperitoneal injection (100 μL) of **Cy5-TAMM-SCH_2CF_3** (500 μM) and/or **Cy7-tetrazine** (500 μM). After 5 h, fluorescence images were acquired using IVIS Lumina system (Cy5: excitation = 640 nm, emission = 680 nm; Cy7: excitation = 745 nm, emission = 800 nm).

Supplementary Text

Energies and Cartesian coordinates for optimized geometries

Structures were optimized at the level of ωB97X-D/6-31+G(d,p) in vacuum, and the electronic energies in water were calculated at the same level using the SMD model in Gaussian 09. Thermal correction, used for free energy calculation, was evaluated using Shermo 2.3 with a scaling factor for zero-point energy = 0.9523, low-frequency vibration modes (< 100 cm^{-1}) raised to 100 cm^{-1}, and temperature = 298.15 K. Energies are given in Hartrees and coordinates in Ångstroms. Transition states were labelled with 'TS' under the structures.

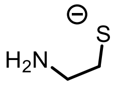

E (vac) = -532.747880958
E (H_2O) = -532.844371663

C	0.158701	0.895039	-0.277006
C	-1.201113	0.511637	0.314075
N	-1.670943	-0.755597	-0.257382
S	1.480129	-0.327900	0.077305
H	-0.841274	-1.355179	-0.294982
H	-2.326393	-1.202999	0.375414
H	0.033181	1.015909	-1.362681
H	0.430087	1.879294	0.128026
H	-1.085533	0.461717	1.410048
H	-1.941049	1.296781	0.086562

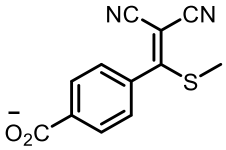

E (vac) = -1119.43458656
E (H_2O) = -1119.53033433

C	2.175395	-1.114194	0.591539
C	0.790152	-1.173841	0.586905
C	0.037756	-0.159160	-0.035731
C	0.719652	0.900588	-0.663915
C	2.105677	0.935756	-0.667669
C	2.851845	-0.067384	-0.041310
C	-1.425445	-0.216311	-0.072664
C	-2.221655	0.897210	0.104528
C	-1.718414	2.119491	0.657879
N	-1.388832	3.128527	1.128193
C	-3.596353	0.950428	-0.280724
N	-4.706291	1.015963	-0.618549
S	-2.049909	-1.812657	-0.431403
C	-3.675785	-1.929395	0.371325
H	2.777411	-1.874587	1.078910
H	0.278216	-1.990919	1.085957

H	0.155880	1.674595	-1.176357
H	2.653168	1.732095	-1.161631
H	-3.826408	-2.996397	0.548613
H	-4.481216	-1.538326	-0.248709
H	-3.660379	-1.407441	1.330120
C	4.405495	-0.029342	-0.059302
O	4.959796	-0.977448	0.539252
O	4.899931	0.941072	-0.672644

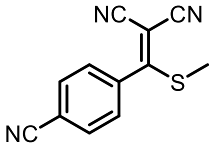

E (vac) = -1023.64968012
E (H_2O) = -1023.66414607

C	2.489193	-0.996953	0.805231
C	1.102286	-1.038626	0.794579
C	0.376961	-0.193071	-0.052378
C	1.058837	0.680889	-0.907077
C	2.445099	0.718742	-0.910792
C	3.162647	-0.119032	-0.050547
C	-1.110395	-0.226286	-0.065415
C	-1.827335	0.928754	0.100895
C	-1.182762	2.145953	0.506059
N	-0.686270	3.138386	0.843203
C	-3.231516	1.049763	-0.149259
N	-4.359451	1.180451	-0.386114
S	-1.722952	-1.829872	-0.349924
C	-3.427390	-1.885172	0.271003
H	3.053116	-1.640297	1.470987
H	0.578127	-1.717669	1.457981
H	0.501618	1.327606	-1.576094
H	2.973672	1.393001	-1.574754
H	-3.627906	-2.949531	0.408226
H	-4.142495	-1.463267	-0.432751
H	-3.502095	-1.381000	1.235506
C	4.600659	-0.083358	-0.049621
N	5.759362	-0.058912	-0.049427

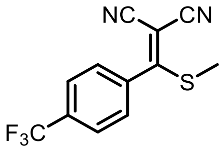

E (vac) = -1268.41267694
E (H_2O) = -1268.42179447

C	1.768722	-0.951916	0.808086
C	0.383099	-1.014520	0.797208
C	-0.352421	-0.175100	-0.048403

C	0.320358	0.708550	-0.896979
C	1.708945	0.762723	-0.896272
C	2.429471	-0.064245	-0.040691
C	-1.838732	-0.226352	-0.064668
C	-2.571780	0.919162	0.099043
C	-1.946734	2.145179	0.508238
N	-1.469627	3.145738	0.849424
C	-3.975993	1.021657	-0.159152
N	-5.104186	1.137157	-0.402892
S	-2.432330	-1.837029	-0.350235
C	-4.135197	-1.912664	0.273031
H	2.336027	-1.591237	1.475782
H	-0.132815	-1.702110	1.458351
H	-0.242491	1.351544	-1.564968
H	2.224680	1.448838	-1.557341
H	-4.321852	-2.978853	0.415527
H	-4.857031	-1.503453	-0.431372
H	-4.215387	-1.404981	1.235291
C	3.937068	-0.041853	-0.031085
F	4.440856	0.967139	-0.759997
F	4.422138	0.083649	1.219404
F	4.446118	-1.187596	-0.530893

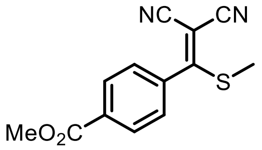

E (vac) = -1159.26225478
E (H_2O) = -1159.27578021

C	1.765920	-0.904705	0.635487
C	0.381319	-1.008570	0.661988
C	-0.404331	-0.138708	-0.102691
C	0.214151	0.821197	-0.912920
C	1.597633	0.912801	-0.951133
C	2.377219	0.054168	-0.174663
C	-1.887486	-0.235366	-0.077336
C	-2.649553	0.871662	0.189354
C	-2.050168	2.082391	0.675056
N	-1.597321	3.068674	1.084228
C	-4.062929	0.948960	-0.023464
N	-5.200939	1.045376	-0.227045
S	-2.443269	-1.840132	-0.459115
C	-4.121341	-2.011806	0.211132
H	2.374295	-1.565677	1.241065
H	-0.094179	-1.753313	1.291113
H	-0.390075	1.487083	-1.519867
H	2.089438	1.647412	-1.579058
H	-4.270152	-3.089976	0.296381
H	-4.878656	-1.586219	-0.444709
H	-4.186329	-1.563464	1.203513
C	3.862809	0.198566	-0.251052

O	4.429157	1.003290	-0.956801
O	4.503549	-0.663757	0.548754
C	5.931626	-0.580649	0.536181
H	6.272872	-1.339734	1.238028
H	6.315774	-0.780561	-0.466276
H	6.256291	0.412309	0.854107

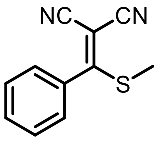

E (vac) = -931.444648305
E (H_2O) = -931.454694372

C	3.103157	-1.061097	0.746531
C	1.713057	-1.091466	0.750822
C	0.989023	-0.215310	-0.067010
C	1.676250	0.671506	-0.905384
C	3.065508	0.687532	-0.916305
C	3.780844	-0.174294	-0.087412
C	-0.495440	-0.227296	-0.067702
C	-1.203178	0.934931	0.101122
C	-0.553651	2.144653	0.519585
N	-0.062795	3.134852	0.871929
C	-2.603416	1.069957	-0.161564
N	-3.728390	1.209408	-0.408958
S	-1.140848	-1.819849	-0.347157
C	-2.830659	-1.850611	0.314831
H	3.657851	-1.730135	1.395924
H	1.184841	-1.779515	1.402424
H	1.120102	1.339323	-1.555066
H	3.589535	1.374841	-1.571758
H	4.865949	-0.155204	-0.092330
H	-3.041492	-2.910973	0.467086
H	-3.558317	-1.425052	-0.373806
H	-2.875578	-1.336554	1.276150

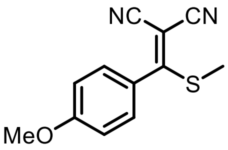

E (vac) = -1045.94153759
E (H_2O) = -1045.95319404

C	-2.276688	-1.280999	-0.803916
C	-0.897029	-1.251808	-0.745682
C	-0.229232	-0.267940	0.004538
C	-0.994145	0.662541	0.715221
C	-2.382396	0.633874	0.676822
C	-3.030881	-0.337843	-0.092056
C	1.244798	-0.219365	0.066682

C	1.925074	0.963898	-0.091483
C	1.271137	2.139554	-0.589712
N	0.776628	3.103427	-1.005412
C	3.299448	1.146011	0.259980
N	4.402666	1.316868	0.577159
S	1.957186	-1.775155	0.404647
C	3.642843	-1.753884	-0.270206
H	-2.798443	-2.023424	-1.397183
H	-0.322622	-1.982111	-1.305592
H	-0.501747	1.414295	1.323186
H	-2.940296	1.366335	1.246516
H	3.887014	-2.806035	-0.429241
H	4.361565	-1.306577	0.414160
H	3.662890	-1.233853	-1.229340
O	-4.370268	-0.449585	-0.207611
C	-5.189937	0.482068	0.475462
H	-6.216242	0.210697	0.231617
H	-5.044244	0.415563	1.559745
H	-4.993710	1.505571	0.136535

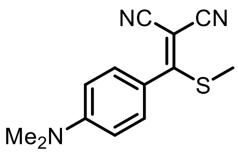

E (vac) = -1065.3879687

E (H_2O) = -1065.40082823

C	2.038342	-1.163228	0.544693
C	0.657175	-1.205747	0.520504
C	-0.098256	-0.188667	-0.084387
C	0.601797	0.863133	-0.698410
C	1.981213	0.911062	-0.697750
C	2.744005	-0.098814	-0.064321
C	-1.565334	-0.218570	-0.095114
C	-2.315570	0.913544	0.135310
C	-1.734147	2.098816	0.693651
N	-1.299849	3.067719	1.162637
C	-3.702285	1.021795	-0.193379
N	-4.819889	1.129343	-0.488810
S	-2.225069	-1.791056	-0.480307
C	-3.843909	-1.896648	0.337620
H	2.570241	-1.960682	1.047627
H	0.149531	-2.032474	1.006434
H	0.054915	1.650039	-1.207155
H	2.468040	1.738996	-1.196711
H	-4.019488	-2.965691	0.472333
H	-4.643140	-1.461341	-0.259930
H	-3.804119	-1.415048	1.316211
N	4.111575	-0.049042	-0.046162
C	4.806143	1.080115	-0.634208
H	5.878798	0.954055	-0.490443
H	4.617223	1.156088	-1.712375

H	4.510786	2.025230	-0.162029
C	4.868920	-1.103503	0.598538
H	5.933474	-0.900288	0.485923
H	4.647949	-1.167368	1.671877
H	4.665475	-2.080494	0.143017

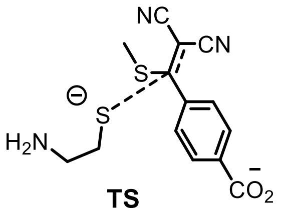

TS

E (vac) = -1652.13707779

E (H_2O) = -1652.37208519

C	-2.507906	-2.281479	-0.881712
C	-1.667045	-1.172766	-0.923474
C	-1.295889	-0.515393	0.256276
C	-1.805200	-1.004452	1.470278
C	-2.650169	-2.109847	1.498283
C	-3.013408	-2.771550	0.324784
C	-0.429876	0.706528	0.259368
C	-0.847809	1.865583	-0.413558
C	-1.808406	1.842636	-1.464762
N	-2.588544	1.909594	-2.327039
C	-0.344875	3.164319	-0.140051
N	0.012896	4.252070	0.083525
S	0.578028	0.815869	1.734675
C	1.993785	1.856512	1.296625
H	-2.783670	-2.802265	-1.793412
H	-1.246426	-0.837705	-1.864422
H	-1.556683	-0.508344	2.404454
H	-3.049452	-2.488173	2.434552
H	2.781330	1.573448	2.001052
H	1.782864	2.922609	1.372550
H	2.298389	1.574605	0.282134
C	1.818442	-1.805848	-0.514229
C	3.093445	-1.802405	0.336517
N	4.335172	-1.524645	-0.396404
S	1.517720	-0.237123	-1.394790
H	4.167621	-0.654586	-0.904440
H	4.435417	-2.229531	-1.124514
H	1.873429	-2.626941	-1.243430
H	0.957600	-2.020077	0.134217
H	2.992054	-1.045654	1.122528
H	3.204441	-2.774685	0.838286
C	-3.939589	-4.012456	0.368907
O	-4.343748	-4.346940	1.510403
O	-4.185081	-4.549424	-0.737589

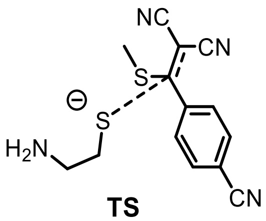

TS

E (vac) = -1556.44107691
E (H_2O) = -1556.50757633

C	-2.401906	-2.311678	-0.828538
C	-1.580604	-1.193285	-0.828574
C	-1.371861	-0.473019	0.351024
C	-2.005596	-0.887622	1.529519
C	-2.828064	-2.005600	1.536812
C	-3.028227	-2.723401	0.353189
C	-0.516238	0.752788	0.381337
C	-0.912671	1.871403	-0.327166
C	-1.929291	1.799820	-1.328164
N	-2.766143	1.777068	-2.134572
C	-0.371670	3.172845	-0.120727
N	0.016429	4.253975	0.060025
S	0.590726	0.767854	1.755023
C	1.992347	1.792543	1.240117
H	-2.552113	-2.871244	-1.745247
H	-1.036872	-0.897100	-1.719651
H	-1.862289	-0.329167	2.449035
H	-3.315581	-2.322188	2.452508
H	2.812926	1.491188	1.895911
H	1.800575	2.859602	1.339625
H	2.227105	1.503766	0.207450
C	1.882896	-1.865152	-0.551548
C	3.171429	-1.795674	0.279186
N	4.393207	-1.509266	-0.473594
S	1.543191	-0.348775	-1.512611
H	4.199589	-0.675273	-1.029171
H	4.535994	-2.250468	-1.156070
H	1.947432	-2.724120	-1.235410
H	1.046173	-2.069901	0.134034
H	3.058190	-1.016519	1.042267
H	3.314485	-2.745834	0.813906
C	-3.874465	-3.885374	0.359122
N	-4.559451	-4.822202	0.372180

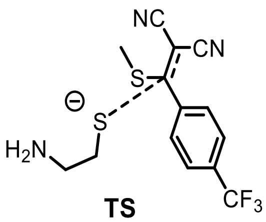

TS

E (vac) =	-1801.20087962		
E (H_2O) =	-1801.26454388		

C	-2.404270	-2.317541	-0.833644
C	-1.574374	-1.201520	-0.835754
C	-1.361722	-0.480101	0.339430
C	-2.002156	-0.889735	1.517033
C	-2.829877	-2.003474	1.521230
C	-3.029848	-2.720840	0.342186
C	-0.500147	0.742730	0.370316
C	-0.902047	1.868624	-0.326812
C	-1.915875	1.802325	-1.330378
N	-2.750066	1.785348	-2.139984
C	-0.364487	3.169325	-0.110116
N	0.021251	4.250164	0.078953
S	0.595888	0.755910	1.754860
C	1.997161	1.790414	1.259513
H	-2.551484	-2.872936	-1.752519
H	-1.031651	-0.910065	-1.728613
H	-1.859009	-0.330932	2.436412
H	-3.318897	-2.311944	2.439600
H	2.813987	1.488530	1.919826
H	1.799705	2.855930	1.364682
H	2.241846	1.511516	0.226669
C	1.892743	-1.849518	-0.554822
C	3.187482	-1.783915	0.266257
N	4.403252	-1.492212	-0.494599
S	1.542915	-0.326694	-1.501187
H	4.205563	-0.653248	-1.041186
H	4.538992	-2.227617	-1.184776
H	1.953005	-2.703186	-1.245733
H	1.060979	-2.059493	0.134914
H	3.079661	-1.008978	1.034273
H	3.335444	-2.736974	0.794497
C	-3.930970	-3.919879	0.374316
F	-5.205908	-3.591164	0.696592
F	-3.987577	-4.570458	-0.803198
F	-3.539968	-4.827544	1.301416

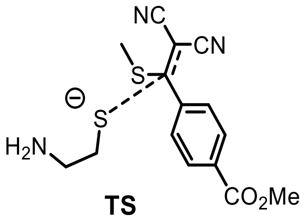

TS

E (vac) =	-1692.04638127		
E (H_2O) =	-1692.11838226		

C	-2.394172	-2.289707	-0.897273
C	-1.585142	-1.161808	-0.884826
C	-1.355749	-0.476835	0.312301

C	-1.955035	-0.941504	1.490742
C	-2.762003	-2.070930	1.478151
C	-2.986210	-2.753535	0.279877
C	-0.515963	0.758133	0.365477
C	-0.915515	1.885374	-0.328724
C	-1.925234	1.831059	-1.337709
N	-2.756371	1.832090	-2.150440
C	-0.387945	3.187376	-0.092910
N	-0.011919	4.268804	0.111351
S	0.580212	0.775439	1.750444
C	1.983269	1.804946	1.249572
H	-2.565789	-2.827223	-1.823431
H	-1.070389	-0.833012	-1.780548
H	-1.797279	-0.412701	2.425449
H	-3.219366	-2.422492	2.395643
H	2.799465	1.502905	1.910689
H	1.789016	2.871490	1.350776
H	2.226512	1.518750	0.217906
C	1.870037	-1.848390	-0.561506
C	3.135506	-1.805087	0.305267
N	4.381943	-1.529759	-0.411584
S	1.573661	-0.316056	-1.511239
H	4.214364	-0.686301	-0.961639
H	4.527875	-2.264438	-1.100600
H	1.944221	-2.698722	-1.255287
H	1.011035	-2.052910	0.095830
H	3.012291	-1.031134	1.072052
H	3.251033	-2.762110	0.834831
C	-3.838310	-3.967490	0.215638
O	-4.093352	-4.590276	-0.794193
O	-4.321248	-4.330080	1.423418
C	-5.154357	-5.484262	1.431020
H	-5.408410	-5.654546	2.477106
H	-6.058390	-5.310290	0.841632
H	-4.624978	-6.347106	1.019619

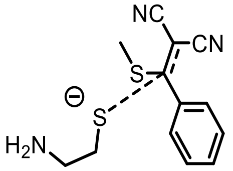

TS

E (vac) = -1464.22450986
E (H_2O) = -1464.29511113

C	-2.444943	-2.295437	-0.841234
C	-1.607731	-1.185014	-0.859918
C	-1.332374	-0.490293	0.321542
C	-1.917901	-0.928615	1.516173
C	-2.755107	-2.041280	1.530427
C	-3.021469	-2.730213	0.350300

C	-0.471716	0.734703	0.335294
C	-0.890940	1.865157	-0.354662
C	-1.908078	1.802142	-1.353459
N	-2.744848	1.796505	-2.161485
C	-0.364207	3.168465	-0.134748
N	0.013400	4.253010	0.054139
S	0.608547	0.787767	1.739423
C	2.007524	1.825525	1.244589
H	-2.638649	-2.826206	-1.768394
H	-1.112040	-0.874535	-1.772302
H	-1.729832	-0.392851	2.441638
H	-3.199744	-2.365254	2.466934
H	-3.672400	-3.600045	0.359508
H	2.821681	1.536408	1.914140
H	1.802547	2.891038	1.335489
H	2.263373	1.539950	0.216986
C	1.854590	-1.845574	-0.520247
C	3.145301	-1.793895	0.307668
N	4.369063	-1.517419	-0.447202
S	1.523381	-0.319577	-1.466310
H	4.186818	-0.671506	-0.988418
H	4.493428	-2.249832	-1.142837
H	1.908778	-2.698958	-1.212172
H	1.014356	-2.044468	0.162021
H	3.041976	-1.017106	1.074145
H	3.279683	-2.748368	0.837286

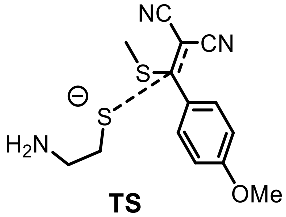

TS

E (vac) = -1578.7192907
E (H_2O) = -1578.79210243

C	-2.732645	-1.989926	-0.582993
C	-1.801429	-0.956438	-0.652301
C	-1.396900	-0.271403	0.491721
C	-1.958002	-0.647185	1.721237
C	-2.886644	-1.673791	1.802987
C	-3.278901	-2.352276	0.647688
C	-0.437562	0.875493	0.435887
C	-0.816951	2.038828	-0.229165
C	-1.899589	2.046164	-1.157342
N	-2.786323	2.093349	-1.909452
C	-0.180180	3.298697	-0.059219
N	0.290015	4.353019	0.091659
S	0.715934	0.847605	1.783621
C	2.150567	1.802720	1.228503
H	-3.007574	-2.499399	-1.498804

H	-1.334611	-0.706569	-1.598284
H	-1.678335	-0.121718	2.629311
H	-3.322052	-1.960092	2.754531
H	2.975495	1.464034	1.860634
H	2.012024	2.877614	1.331320
H	2.344651	1.511289	0.189676
C	1.597586	-1.850596	-0.501349
C	2.975898	-1.936429	0.165772
N	4.122139	-1.822053	-0.738075
S	1.327563	-0.306953	-1.436039
H	3.984110	-0.962858	-1.270981
H	4.067034	-2.568890	-1.427275
H	1.472615	-2.712684	-1.173136
H	0.827555	-1.942347	0.279258
H	3.056831	-1.138159	0.912651
H	3.062991	-2.890057	0.706690
O	-4.194650	-3.351150	0.822941
C	-4.623767	-4.055965	-0.318999
H	-3.788914	-4.572966	-0.809405
H	-5.346435	-4.792800	0.034438
H	-5.109599	-3.389750	-1.043259

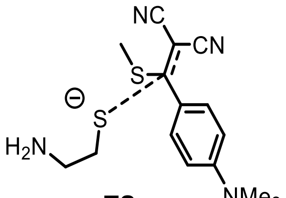

TS

E (vac) = -1598.16011894
E (H_2O) = -1598.23622465

C	2.416116	-0.367543	-1.065190
C	1.046704	-0.550369	-1.000516
C	0.393007	-0.684378	0.232252
C	1.173168	-0.631433	1.386681
C	2.553101	-0.437642	1.327375
C	3.202354	-0.296937	0.096890
C	-1.079123	-0.909484	0.328889
C	-1.658079	-2.003296	-0.309129
C	-0.982672	-2.738750	-1.327250
N	-0.463280	-3.376740	-2.150114
C	-2.954431	-2.500025	0.000538
N	-3.992985	-2.957065	0.260837
S	-1.809577	-0.157622	1.765882
C	-3.523495	0.206605	1.306754
H	2.882276	-0.290397	-2.041842
H	0.458942	-0.540044	-1.909456
H	0.710574	-0.738619	2.363089
H	3.104607	-0.386815	2.258952
H	-3.846888	0.973284	2.015681
H	-4.175096	-0.663635	1.367465

H	-3.495087	0.629969	0.295101
C	-0.697516	2.488749	-0.494584
C	-1.564922	3.477284	0.294387
N	-2.509657	4.262570	-0.502685
S	-1.660297	1.257509	-1.436100
H	-3.047833	3.592350	-1.052777
H	-1.984679	4.801746	-1.187897
H	-0.049013	3.052207	-1.181711
H	-0.033455	1.970398	0.213432
H	-2.140261	2.920418	1.043159
H	-0.918237	4.176454	0.844686
N	4.601853	-0.114551	-0.010964
C	5.370218	-0.229148	1.209249
H	6.434675	-0.233567	0.955751
H	5.192040	0.602263	1.916316
H	5.139555	-1.170485	1.714105
C	5.033420	1.018455	-0.815544
H	6.100903	0.920460	-1.037960
H	4.494975	1.054698	-1.761751
H	4.871362	1.979121	-0.295179

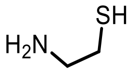

E (vac) = -533.316532174
E (H_2O) = -533.326284395

N	-1.734420	-0.727060	-0.233359
C	-1.209551	0.505850	0.334936
C	0.118251	0.878595	-0.313927
H	-1.075516	0.474567	1.430218
H	-1.923656	1.310162	0.122216
S	1.461619	-0.326607	-0.004905
H	0.445425	1.868591	0.012971
H	-0.000039	0.897688	-1.400439
H	1.588146	-0.080078	1.310509
H	-2.633982	-0.964232	0.167654
H	-1.097539	-1.498242	-0.057194

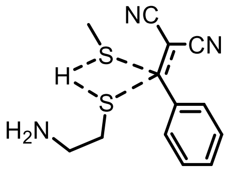

TS

E (vac) = -1464.69385614
E (H_2O) = -1464.71709856

N	0.384158	0.338676	3.161807
C	0.474923	-0.305304	2.193122
C	0.603422	-1.040697	0.995180
C	1.454184	-2.168522	0.958544

N	2.151119	-3.097253	0.838513
C	0.089975	-0.480831	-0.237749
S	1.522327	0.447559	-1.380728
C	2.882273	0.685521	-0.208757
C	2.962816	2.122568	0.299678
N	1.694158	2.536427	0.876507
C	-1.164678	0.348324	-0.112159
C	-1.274300	1.658407	-0.574784
C	-2.480132	2.346091	-0.445373
C	-3.582880	1.732834	0.139305
C	-3.474909	0.425920	0.614171
C	-2.273225	-0.259634	0.493790
S	-0.109095	-1.898997	-1.489370
C	-1.100532	-1.110437	-2.783988
H	1.221633	-1.233999	-1.867223
H	2.720888	-0.014082	0.618395
H	3.801648	0.386960	-0.718427
H	3.813484	2.164420	0.999321
H	3.195734	2.792340	-0.534896
H	1.479691	2.033867	1.735354
H	1.694721	3.525810	1.093694
H	-0.407611	2.153865	-0.994477
H	-2.549463	3.370387	-0.797372
H	-4.519748	2.271719	0.238821
H	-4.323189	-0.055548	1.089588
H	-2.180629	-1.268325	0.885769
H	-0.650616	-0.169935	-3.106721
H	-1.143923	-1.814746	-3.616137
H	-2.102566	-0.930395	-2.393103

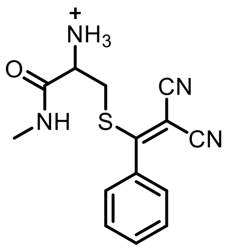

E (vac) = -1234.42685077
E (H_2O) = -1234.53350755

C	3.448871	2.130845	-1.245493
C	2.620187	1.028173	-1.402531
C	2.065791	0.396870	-0.276240
C	2.330233	0.915400	1.003175
C	3.143968	2.031385	1.149912
C	3.709493	2.635399	0.028248
C	1.200414	-0.779018	-0.431100
C	1.221326	-1.866463	0.394348
C	2.228134	-2.083578	1.393492
N	3.026952	-2.288038	2.207327
C	0.150127	-2.822689	0.364895
N	-0.792823	-3.497694	0.394082

H	3.894361	2.596003	-2.117882
H	2.426045	0.636298	-2.395187
H	1.896045	0.453378	1.883989
H	3.340157	2.426838	2.140410
H	4.354097	3.499934	0.146255
C	-1.182571	0.382520	-1.175727
C	-2.064560	-0.230505	-0.074965
N	-2.708155	-1.492133	-0.551550
S	0.059069	-0.788873	-1.814025
H	-2.208142	-1.887214	-1.355910
H	-2.702790	-2.229465	0.161387
H	-1.793315	0.677816	-2.035214
H	-0.687904	1.280626	-0.797686
H	-1.466454	-0.463635	0.809661
H	-3.688967	-1.220856	-0.783760
C	-3.255171	0.698410	0.262799
O	-4.355902	0.401509	-0.194234
N	-2.983996	1.785334	0.986503
H	-2.062188	1.897212	1.382773
C	-4.013421	2.759398	1.343489
H	-4.516397	2.470343	2.269294
H	-3.545202	3.735190	1.469412
H	-4.748006	2.808251	0.540380

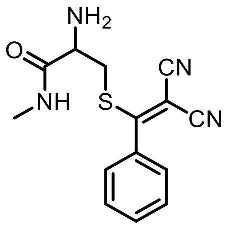

E (vac) = -1234.06141435
E (H_2O) = -1234.08806931

C	-2.457509	0.984114	-1.976498
N	-2.540570	0.001505	-0.911471
C	-1.766349	-1.104792	-0.916286
O	-1.002638	-1.374915	-1.839357
C	-1.878100	-2.045252	0.296870
N	-1.881490	-3.443316	-0.100248
C	-0.688641	-1.846407	1.251773
S	-0.614438	-0.179623	1.990244
C	0.348382	0.703227	0.809133
C	-0.040585	1.964405	0.459551
C	0.789695	2.830622	-0.327585
N	1.435393	3.552095	-0.965548
C	-1.317961	2.496862	0.836742
N	-2.367947	2.916003	1.096491
C	1.588177	0.091340	0.287665
C	2.543165	-0.420600	1.176409
C	3.712757	-0.988825	0.687467
C	3.925270	-1.073878	-0.688377

C	2.968128	-0.585627	-1.573804
C	1.802695	0.000080	-1.091148
H	-1.459871	1.433606	-2.025206
H	-2.662441	0.511512	-2.939427
H	-3.190756	1.767613	-1.787067
H	-3.053449	0.240006	-0.075979
H	-2.803028	-1.838695	0.846763
H	-1.182883	-3.577962	-0.827835
H	-2.778133	-3.700737	-0.498664
H	0.253773	-2.058695	0.739454
H	-0.790008	-2.547173	2.082432
H	2.371024	-0.355603	2.246266
H	4.457388	-1.366720	1.380254
H	4.835001	-1.526632	-1.069474
H	3.121336	-0.667493	-2.644333
H	1.041709	0.344632	-1.782280

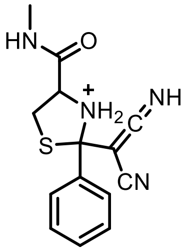

E (vac) = -1234.41015423
E (H_2O) = -1234.51093707

C	5.367885	-0.921704	0.351829
N	3.962863	-1.275684	0.168821
C	2.961915	-0.430185	0.422879
O	3.083074	0.714408	0.853164
C	1.534143	-0.949510	0.133694
C	1.189141	-0.989018	-1.372068
S	0.356082	0.579522	-1.750173
C	-0.513304	0.472419	-0.146029
C	-1.023201	1.828265	0.310335
C	-0.953431	2.923173	-0.450560
N	-1.040940	3.862776	-1.195138
C	-1.574345	1.910468	1.622217
N	-1.951937	1.879457	2.719447
C	-1.594995	-0.609597	-0.080516
C	-2.327258	-0.933072	-1.225611
C	-3.346196	-1.878187	-1.165969
C	-3.649038	-2.511110	0.036672
C	-2.936509	-2.182318	1.185496
C	-1.919189	-1.233743	1.129693
N	0.641866	0.069701	0.767390
H	5.854652	-0.775187	-0.614842
H	5.874465	-1.716030	0.901521
H	5.417858	0.004158	0.923077
H	3.747703	-2.203991	-0.165763
H	1.358160	-1.921416	0.599744

H	2.084765	-1.064685	-1.990734
H	0.525234	-1.826713	-1.593630
H	-0.332508	4.587105	-1.279686
H	-2.103356	-0.446632	-2.168933
H	-3.903577	-2.117895	-2.065010
H	-4.441845	-3.249717	0.080509
H	-3.176516	-2.651328	2.133291
H	-1.433275	-0.972127	2.066882
H	0.300006	-0.239597	1.680377
H	1.289667	0.877411	0.934642

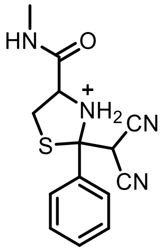

E (vac) = -1234.42045513
E (H_2O) = -1234.53425874

C	-4.145876	0.405897	-0.851740
C	-2.868442	0.823503	-0.496400
C	-1.811808	-0.091221	-0.478600
C	-2.054350	-1.431674	-0.803245
C	-3.334173	-1.841319	-1.165734
C	-4.380011	-0.923827	-1.192418
C	-0.430543	0.361351	-0.045009
C	-0.185930	0.120632	1.497088
C	-1.133681	0.917260	2.283955
N	-1.872473	1.569030	2.887421
C	-0.281964	-1.310495	1.822345
N	-0.306296	-2.454611	1.986253
H	-4.957615	1.124537	-0.859666
H	-2.712142	1.862352	-0.221544
H	-1.273927	-2.186227	-0.743213
H	-3.511651	-2.882594	-1.410373
H	-5.377906	-1.246170	-1.468970
C	1.695849	1.777297	-0.475845
C	1.877793	0.380799	-1.090321
N	0.640747	-0.420609	-0.794199
S	-0.086729	2.095132	-0.506931
H	2.103246	1.832998	0.537465
H	2.190441	2.539990	-1.079514
H	1.983978	0.447918	-2.174809
H	0.828042	0.440627	1.769528
H	1.005223	-1.233103	-0.234014
C	3.027269	-0.419032	-0.441416
O	2.723400	-1.352417	0.300948
N	4.270466	-0.021646	-0.699741
H	4.428497	0.740929	-1.341929
C	5.426053	-0.688310	-0.098624

H	6.329597	-0.287337	-0.554568
H	5.450582	-0.510958	0.978311
H	5.369520	-1.762605	-0.280210
H	0.216357	-0.773452	-1.655530

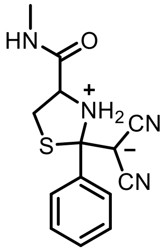

E (vac) = -1234.02225618
E (H_2O) = -1234.07872476

C	2.277915	-2.832495	0.832089
C	1.657738	-1.610804	1.090892
C	1.441304	-0.697693	0.060958
C	1.870367	-1.020623	-1.233252
C	2.480954	-2.241453	-1.492305
C	2.682335	-3.154978	-0.458972
C	0.730101	0.637506	0.268794
C	1.492896	1.805853	-0.200327
C	2.882194	1.738053	-0.417437
N	4.031129	1.660316	-0.607005
C	0.785642	3.016153	-0.336852
N	0.075589	3.936164	-0.464896
H	2.452265	-3.527295	1.647344
H	1.365096	-1.356111	2.103831
H	1.776996	-0.291166	-2.034208
H	2.821604	-2.467256	-2.497298
H	3.169266	-4.104260	-0.657212
C	-1.451248	-0.229122	1.612317
C	-1.523105	-0.433370	0.076108
N	-0.603068	0.540320	-0.545748
S	-0.032438	0.860959	1.959293
H	-1.042615	1.473808	-0.583986
H	-0.385095	0.292852	-1.513583
H	-2.355157	0.255881	1.987217
H	-1.343162	-1.199856	2.101800
H	-1.147001	-1.426593	-0.182903
C	-2.921830	-0.212892	-0.502682
O	-3.157391	0.765312	-1.196867
N	-3.836729	-1.147472	-0.178861
H	-3.570721	-1.918792	0.413131
C	-5.222755	-1.024555	-0.605249
H	-5.752425	-1.937861	-0.334974
H	-5.703421	-0.168438	-0.124779
H	-5.270981	-0.885544	-1.687050

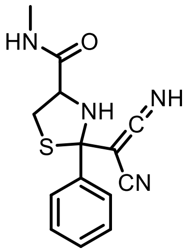

E (vac) =	-1234.0508241
E (H_2O) =	-1234.07236963

C	-3.465280	2.245565	-0.231756
N	-2.569077	1.351743	-0.939639
C	-2.603866	0.020546	-0.737410
O	-3.472884	-0.545107	-0.076819
C	-1.489794	-0.759429	-1.473597
C	-1.351553	-2.206748	-0.946141
S	0.198549	-2.277996	0.014743
C	0.458911	-0.436931	-0.141286
C	-0.234359	0.289748	1.024418
C	-1.097121	-0.304047	1.841818
N	-1.859470	-0.836658	2.617877
C	-0.121274	1.710191	1.090421
N	-0.036553	2.868656	1.083341
C	1.936187	-0.079567	-0.165857
C	2.393779	0.979222	-0.952531
C	3.741643	1.333349	-0.940850
C	4.640933	0.642426	-0.135778
C	4.184905	-0.402337	0.666632
C	2.841288	-0.758211	0.654619
N	-0.211838	-0.056633	-1.379492
H	-4.484685	1.862153	-0.293712
H	-3.186028	2.337855	0.823926
H	-3.417779	3.231433	-0.694756
H	-1.701187	1.721238	-1.304610
H	-1.786621	-0.772423	-2.527232
H	-2.212207	-2.462159	-0.326386
H	-1.274955	-2.918669	-1.769304
H	-2.823046	-0.917977	2.280189
H	1.694034	1.546340	-1.557227
H	4.082471	2.159965	-1.555903
H	5.689975	0.920303	-0.125276
H	4.876765	-0.942135	1.305130
H	2.497858	-1.567905	1.292027
H	0.383869	-0.321640	-2.159010

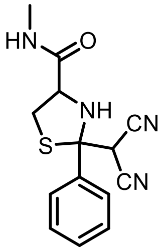

E (vac) = -1234.06616877
E (H_2O) = -1234.09393092

C	-4.347790	-0.005517	0.174716
C	-3.103340	0.505284	-0.187442
C	-1.938505	-0.237784	0.017940
C	-2.055256	-1.515253	0.575985
C	-3.295466	-2.025407	0.940365
C	-4.449225	-1.269863	0.743664
C	-0.557731	0.265588	-0.405139
C	-0.494612	1.843698	-0.384359
C	-0.814183	2.358300	0.957901
N	-1.068698	2.729429	2.023307
C	0.845316	2.308552	-0.771383
N	1.904047	2.667308	-1.067526
H	-5.237888	0.593568	0.013449
H	-3.069208	1.496054	-0.627505
H	-1.173557	-2.133475	0.712523
H	-3.360183	-3.017285	1.375529
H	-5.418016	-1.666503	1.028634
C	1.488350	-0.820999	-1.679541
C	1.401975	-1.211440	-0.202491
N	0.538254	-0.216376	0.423855
S	-0.206859	-0.332250	-2.119000
H	0.217202	-0.495993	1.343484
H	2.179960	0.010776	-1.830667
H	1.790779	-1.668869	-2.294380
H	1.007588	-2.233182	-0.121843
H	-1.207741	2.265038	-1.099883
C	2.812440	-1.245285	0.395284
O	3.522447	-2.228115	0.222239
N	3.193575	-0.135665	1.058236
C	4.543307	0.024716	1.560592
H	4.552023	0.820192	2.306872
H	4.870255	-0.909380	2.021356
H	5.244980	0.274771	0.757889
H	2.552030	0.644803	1.058967

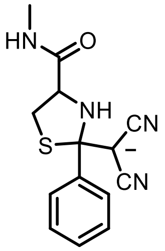

E (vac) =	-1233.54035206
E (H_2O) =	-1233.6247916

C	-4.144373	1.795096	0.365933
N	-2.972054	1.190269	-0.222335
C	-3.026164	0.006616	-0.849723
O	-4.075721	-0.592702	-1.098115
C	-1.688854	-0.608659	-1.267170
C	-1.492794	-1.964343	-0.543841
S	0.296360	-2.206560	-0.454246
C	0.441472	-0.363208	-0.116735
C	1.853811	0.128700	-0.398079
C	2.968401	-0.671091	-0.126480
C	4.257414	-0.181498	-0.314616
C	4.458266	1.118864	-0.774181
C	3.354154	1.929034	-1.028709
C	2.063653	1.441627	-0.837241
C	0.036275	-0.045887	1.319453
C	0.460839	-0.854592	2.385189
N	0.809113	-1.540203	3.269051
C	-0.383159	1.260863	1.584360
N	-0.769424	2.361642	1.728347
N	-0.511406	0.240567	-1.047916
H	-4.712495	1.056452	0.940083
H	-4.808014	2.206592	-0.402770
H	-3.812256	2.593514	1.031153
H	-2.059699	1.557808	0.025791
H	-1.786465	-0.800408	-2.341517
H	-1.906495	-1.927579	0.466462
H	-1.961659	-2.780846	-1.093411
H	2.827037	-1.676748	0.258203
H	5.108363	-0.819636	-0.093702
H	5.464626	1.501373	-0.919184
H	3.494588	2.952154	-1.366184
H	1.210801	2.094237	-0.995480
H	-0.041570	0.374610	-1.935586

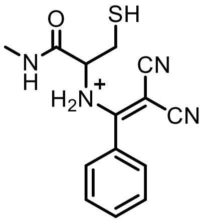

E (vac) = -1234.40567178
E (H_2O) = -1234.50730468

C	-4.255900	-1.937164	-0.840201
N	-3.315650	-1.036020	-0.178934
C	-2.188315	-0.634097	-0.739409
O	-1.782107	-0.974312	-1.862040
C	-1.251232	0.309791	0.064414
C	-1.852954	1.605380	0.619180
S	-2.983003	1.350682	2.024710
N	-0.180102	0.618747	-0.956070
C	1.200123	0.527304	-0.491334
C	1.923722	1.676293	-0.491703
C	1.290932	2.910635	-0.862752
N	0.662654	3.834875	-1.172441
C	3.315161	1.714648	-0.156694
N	4.440330	1.771354	0.110692
C	1.643876	-0.797186	-0.052044
C	1.290377	-1.946433	-0.780617
C	1.729496	-3.193706	-0.358039
C	2.506354	-3.309325	0.794518
C	2.847666	-2.175069	1.528352
C	2.421341	-0.921227	1.110142
H	-5.091188	-1.373874	-1.261903
H	-3.738473	-2.465113	-1.639872
H	-4.630985	-2.654465	-0.109959
H	-3.553206	-0.657607	0.733041
H	-0.772240	-0.268771	0.859847
H	-1.052254	2.285459	0.921684
H	-2.442377	2.116213	-0.148523
H	-2.041558	1.023945	2.926054
H	-0.354777	1.539374	-1.376908
H	-0.463417	-0.110676	-1.704865
H	0.703964	-1.880645	-1.692247
H	1.472049	-4.075886	-0.933583
H	2.847299	-4.286251	1.120302
H	3.445653	-2.264970	2.428216
H	2.681891	-0.044921	1.694077

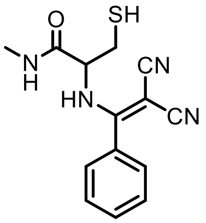

E (vac) = -1234.06770134
E (H_2O) = -1234.09606274

C	3.459359	1.070284	2.710971
N	2.393341	0.817899	1.760262
C	2.606348	0.224995	0.562919
O	3.705345	-0.151872	0.177383

C	1.349473	-0.006025	-0.293780
C	1.668529	-0.051788	-1.796391
S	2.378426	-1.627731	-2.367939
N	0.331442	1.003450	-0.000295
C	-1.007706	0.763997	-0.018740
C	-1.902857	1.779872	-0.289060
C	-1.398838	3.061725	-0.659995
N	-0.881654	4.062694	-0.944816
C	-3.312087	1.609885	-0.150993
N	-4.461317	1.484573	-0.048220
C	-1.475770	-0.603293	0.313575
C	-1.108410	-1.190668	1.529249
C	-1.541787	-2.473524	1.840485
C	-2.334676	-3.179309	0.936452
C	-2.700237	-2.597186	-0.274084
C	-2.277198	-1.308702	-0.585929
H	4.379300	0.653220	2.301031
H	3.593347	2.143842	2.869527
H	3.242390	0.588704	3.668131
H	1.460656	1.144080	1.962888
H	0.951339	-0.980706	0.000915
H	0.729242	0.052993	-2.345898
H	2.320897	0.777907	-2.080782
H	3.516924	-1.515575	-1.664913
H	0.597109	1.951733	-0.251625
H	-0.490985	-0.638041	2.231358
H	-1.262364	-2.922743	2.787543
H	-2.668742	-4.183125	1.177416
H	-3.318778	-3.144192	-0.977301
H	-2.565452	-0.851321	-1.526914

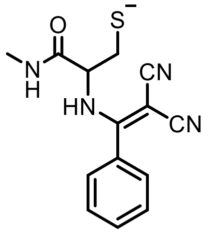

E (vac) = -1233.52417201
E (H_2O) = -1233.61544419

C	3.608244	-1.870152	-1.618304
N	2.579220	-0.861521	-1.479871
C	2.810820	0.256146	-0.735068
O	3.932959	0.572388	-0.367339
C	1.609136	1.171707	-0.489874
C	1.580054	1.913159	0.853457
S	0.264635	3.184909	0.867677
N	0.307763	0.550347	-0.800839
C	-0.448260	-0.262858	-0.034845
C	-0.023306	-1.178548	0.918540
C	-0.985580	-1.944795	1.648484

N	-1.728470	-2.600849	2.256047
C	1.324561	-1.467979	1.285195
N	2.383144	-1.799497	1.632574
C	-1.908254	-0.166794	-0.337917
C	-2.546312	1.064203	-0.154049
C	-3.901885	1.179449	-0.452160
C	-4.613776	0.084096	-0.937081
C	-3.971315	-1.139219	-1.123664
C	-2.619741	-1.267373	-0.820600
H	3.285952	-2.607102	-2.357306
H	4.535062	-1.402159	-1.956623
H	3.801077	-2.367156	-0.660339
H	1.619330	-1.116751	-1.648614
H	1.707851	1.953915	-1.252315
H	2.569825	2.358734	0.978679
H	1.451421	1.191689	1.668972
H	-0.271673	1.207484	-1.307431
H	-1.961827	1.902816	0.237665
H	-4.400546	2.131442	-0.298489
H	-5.671427	0.181298	-1.165416
H	-4.522437	-1.995952	-1.499008
H	-2.118356	-2.221514	-0.951956

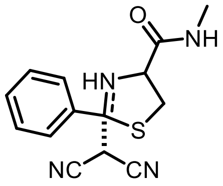

TS

E (vac) = -1234.03179339
E (H_2O) = -1234.05982223

C	-2.965183	-0.635943	-1.323610
C	-1.628779	-0.837073	-1.011591
C	-1.090104	-0.309302	0.169885
C	-1.914478	0.419121	1.032124
C	-3.251783	0.630271	0.707758
C	-3.780430	0.104463	-0.466108
C	0.334504	-0.534721	0.497423
C	1.013083	1.157929	-1.019375
C	0.776705	2.195637	-0.091748
N	0.554071	2.907091	0.806743
C	2.364157	0.855773	-1.298973
N	3.477620	0.531046	-1.432679
H	-3.371885	-1.052395	-2.239129
H	-0.994110	-1.392294	-1.696671
H	-1.529847	0.824368	1.960797
H	-3.878660	1.206677	1.379495
H	-4.824323	0.267479	-0.713041
C	2.592349	-1.653214	0.991098
C	2.093167	-0.745564	2.124513

N	0.869764	-0.102534	1.643242
S	1.130763	-2.004596	-0.050842
H	0.595906	0.798825	2.023497
H	3.356078	-1.191552	0.366081
H	2.974194	-2.594360	1.386798
H	1.832704	-1.344302	3.002735
H	0.307850	1.050665	-1.836333
C	3.080291	0.317592	2.649887
O	3.116159	0.541729	3.853114
N	3.818719	0.979990	1.741959
C	4.663794	2.090674	2.142895
H	5.210012	2.439549	1.266626
H	4.061154	2.909301	2.546618
H	5.371108	1.769485	2.910801
H	3.711450	0.799906	0.751871

Python script for numerical simulation

```
import numpy as np
import matplotlib.pyplot as plt
import ipywidgets as widgets
from ipywidgets import interactive

# Constants (initial values)
k1_initial = 18000  # Initial value for k1
k2_initial = 5500  # Initial value for k2

# Initial concentrations
A0 = 40.0e-6
B0 = 80.0e-6
C0 = 0.0
D0 = 80.0e-6
E0 = 0.0

# Time values
t_max = 1 * 60.0  # Maximum time in seconds; I wrote it this way so the number before 60 is in
minutes
dt = 0.01  # Time step in seconds
t_values = np.arange(0, t_max, dt)

# Initialize concentration arrays
A_values = []
B_values = []
C_values = []
D_values = []
E_values = []

# Function to simulate the time course evolution based on k1 and k2
def simulate_and_plot(k1, k2):
    A = A0
    B = B0
    C = C0
    D = D0
    E = E0

    A_values.clear()
    B_values.clear()
    C_values.clear()
    D_values.clear()
    E_values.clear()

    for t in t_values:
```

```
        dA_dt = -(k1 * A * B + k2 * A * D)
        dB_dt = -k1 * A * B
        dC_dt = k1 * A * B
        dD_dt = -k2 * A * D
        dE_dt = k2 * A * D

        A += dA_dt * dt
        B += dB_dt * dt
        C += dC_dt * dt
        D += dD_dt * dt
        E += dE_dt * dt

        A_values.append(A)
        B_values.append(B)
        C_values.append(C)
        D_values.append(D)
        E_values.append(E)

    # Plot the results
    plt.figure(figsize=(12, 6))

    # Subplot 1: Concentrations of A, B, C, D, and E
    plt.subplot(1, 2, 1)
    plt.plot(t_values, A_values, label='A')
    plt.plot(t_values, B_values, label='B')
    plt.plot(t_values, C_values, label='C')
    plt.plot(t_values, D_values, label='D')
    plt.plot(t_values, E_values, label='E')
    plt.xlabel('Time')
    plt.ylabel('Concentration')
    plt.legend()
    plt.title('Concentration Evolution of A, B, C, D, and E')
    plt.grid(True)

    # Subplot 2: Evolution of C/E and D+E-A  (the plot for D+E-A is disabled)
    plt.subplot(1, 2, 2)
    CE_ratio_values = [C / (E + 1e-20) for C, E in zip(C_values, E_values)]  # Calculate C/E ratio
    # DE_minus_A_values = [D + E - A for A, D, E in zip(A_values, D_values, E_values)]  # Calculate D+E-A
    plt.plot(t_values, CE_ratio_values, label='C/E')
    # plt.plot(t_values, DE_minus_A_values, label='D+E-A')
    plt.xlabel('Time')
    plt.ylabel('Value')
    plt.legend()
    plt.title('Evolution of C/E')
```

```
    plt.grid(True)

    plt.tight_layout()
    plt.show()

    # Calculate final values
    final_A = A_values[-1]
    final_B = B_values[-1]
    final_C = C_values[-1]
    final_D = D_values[-1]
    final_E = E_values[-1]
    CE_ratio_final = final_C / (final_E + 1e-10)  # Calculate C/E ratio at the end

    # Print final values
    print(f'Final A: {final_A}')
    print(f'Final B: {final_B}')
    print(f'Final C: {final_C}')
    print(f'Final D: {final_D}')
    print(f'Final E: {final_E}')
    print(f'Final C/E: {CE_ratio_final}')


# Create interactive widgets for k1 and k2
k1_slider = widgets.FloatSlider(value=k1_initial, min=1000, max=30000, step=500,
description='k1')
k2_slider = widgets.FloatSlider(value=k2_initial, min=1000, max=30000, step=500,
description='k2')

# Create an interactive plot
interactive_plot = interactive(simulate_and_plot, k1=k1_slider, k2=k2_slider)
interactive_plot
```

Sequences of the constructs used in this manuscript

C-HA-Nlgn3
with signal peptide and HA tag colored

- DNA sequence, ATGTGGCTGCAGCCCTCGCTGTCCCTGAGCCCCACGCCCACAGTT
GGCCGGAGCCTGTGCCTCACCTGGGCTTCCTCAGTTTGGTGCTGAGGGCCAGTA
CCTGTCAGTATCCATATGATGTTCCAGATTATGCTGCCCCGGCACCCACAGTCA
ATACTCACTTTGGGAAGCTAAGGGGTGCCAGAGTACCATTGCCCAGTGAAATCC
TGGGTCCTGTGGACCAATACCTGGGGGTACCCTACGCAGCTCCCCCGATCGGCG
AGAAACGTTTCCTGCCCCCTGAACCACCCCCATCCTGGTCGGGCATCCGGAACG
CCACACACTTTCCCCCAGTGTGCCCCCAGAACATCCACACAGCTGTGCCCGAAG
TCATGCTGCCAGTCTGGTTCACTGCCAACTTGGATATCGTCGCCACTTATATCCA
GGAGCCCAACGAAGATTGCCTCTATCTGAATGTGTATGTGCCCACGGAAGATGG
ATCCGGCGCTAAGAAACAGGGCGAGGACTTAGCGGATAATGACGGGGATGAAG
ATGAAGACATCCGAGACAGTGGTGCTAAACCTGTCATGGTCTACATCCACGGAG
GCTCTTACATGGAAGGAACAGGCAACATGATTGATGGCAGTGTTCTTGCAAGTT
ACGGCAACGTCATCGTCATCACCCTCAACTATCGGGTCGGGGTGCTAGGTTTCC
TGAGCACTGGAGATCAGGCTGCCAAGGGCAACTATGGGCTCCTTGATCAAATCC
AGGCCCTTCGCTGGGTGAGTGAGAATATTGCCTTCTTTGGAGGAGATCCCCGTA
GAATTACTGTCTTTGGCTCTGGCATCGGTGCATCCTGTGTCAGTCTCCTTACACT
GTCTCATCATTCTGAGGGGCTTTTCCAGAGGGCCATCATCCAAAGTGGCTCTGCT
CTATCTAGCTGGGCTGTGAACTACCAACCAGTGAAGTATACCAGCTTGCTGGCA
GACAAAGTGGGCTGTAACGTCCTGGACACTGTGGATATGGTGGATTGTCTTCGA
CAAAAGAGTGCCAAGGAGCTGGTAGAACAGGACATTCAGCCAGCCCGCTACCA
TGTGGCTTTTGGCCCTGTGATTGATGGTGATGTCATTCCTGATGACCCTGAGATC
CTTATGGAGCAGGGAGAGTTCCTCAACTATGATATCATGCTAGGCGTCAACCAG
GGTGAGGGTCTCAAGTTTGTGGAAGGGGTGGTGGACCCCGAGGATGGTGTCTCG
GGCACTGACTTTGACTACTCTGTCTCCAATTTTGTGGACAATCTGTATGGCTATC
CTGAGGGTAAGGACACCCTGCGGGAGACTATCAAGTTCATGTATACGGACTGGG
CAGACCGAGACAACCCTGAGACCCGCCGTAAAACACTGGTGGCACTCTTCACTG
ACCACCAGTGGGTGGAGCCTTCAGTGGTGACAGCCGATCTGCACGCCCGCTATG
GCTCACCTACCTACTTCTACGCCTTCTACCATCACTGCCAGAGCCTCATGAAGCC
CGCATGGTCAGATGCAGCACACGGGGATGAAGTGCCCTATGTTTTTGGTGTCCC
TATGGTAGGTCCCACTGACCTTTTCCCCTGCAACTTCTCCAAGAATGATGTTATG
CTCAGTGCTGTCGTCATGACCTATTGGACCAACTTTGCCAAGACCGGGGATCCC
AACAAGCCGGTACCCCAGGATACCAAGTTCATTCACACCAAGGCCAACCGCTTT
GAGGAAGTGGCCTGGTCCAAATACAATCCCCGAGACCAGCTCTACCTTCACATC
GGGCTGAAACCAAGGGTTCGTGATCATTACCGGGCCACAAAGGTAGCCTTTTGG
AAACACCTGGTGCCCCACCTGTACAACCTGCATGACATGTTCCACTATACATCC
ACGACCACCAAAGTGCCGCCCCCGGACACCACCCACAGCTCCCACATCACCCGT
AGGCCCAACGGCAAGACCTGGAGCACCAAGCGGCCGGCGATTTCACCTGCCTA
CAGCAATGAGAATGCCCCTGGGTCCTGGAATGGGGACCAGGATGCGGGGCCAC
TCCTGGTTGAGAACCCTCGAGACTACTCCACTGAATTAAGTGTCACTATCGCTGT
GGGGGCCTCCCTCCTGTTTCTCAATGTGTTGGCCTTTGCTGCCCTCTATTACCGT
AAGGACAAACGGCGCCAGGAGCCCCTGAGGCAGCCTAGCCCCCAAAGGGGAAC
TGGTGCCCCTGAATTGGGAACTGCTCCGGAGGAGGAGCTGGCAGCATTACAGTT

GGGTCCCACTCACCATGAATGTGAGGCCGGTCCCCCACATGACACACTTCGCCT
CACAGCACTGCCCGACTATACCCTGACCCTGCGGCGCTCCCCTGATGACATCCC
ACTCATGACTCCCAACACCATCACTATGATTCCTAATTCCCTGGTTGGGTTGCAG
ACCTTGCACCCCTATAACACCTTTGCCGCAGGGTTCAACAGTACTGGGCTGCCC
CACTCACACTCCACTACCCGTGTA

- Amino acid sequence, MWLQPSLSLSPTPTVGRSLCLTLGFLSLVLRASTCQYPYDVP
DYAAPAPTVNTHFGKLRGARVPLPSEILGPVDQYLGVPYAAPPIGEKRFLPPEPPPS
WSGIRNATHFPPVCPQNIHTAVPEVMLPVWFTANLDIVATYIQEPNEDCLYLNVYV
PTEDGSGAKKQGEDLADNDGDEDEDIRDSGAKPVMVYIHGGSYMEGTGNMIDGSV
LASYGNVIVITLNYRVGVLGFLSTGDQAAKGNYGLLDQIQALRWVSENIAFFGGDP
RRITVFGSGIGASCVSLLTLSHHSEGLFQRAIIQSGSALSSWAVNYQPVKYTSLLADK
VGCNVLDTVDMVDCLRQKSAKELVEQDIQPARYHVAFGPVIDGDVIPDDPEILMEQ
GEFLNYDIMLGVNQGEGLKFVEGVVDPEDGVSGTDFDYSVSNFVDNLYGYPEGKD
TLRETIKFMYTDWADRDNPETRRKTLVALFTDHQWVEPSVVTADLHARYGSPTYF
YAFYHHCQSLMKPAWSDAAHGDEVPYVFGVPMVGPTDLFPCNFSKNDVMLSAVV
MTYWTNFAKTGDPNKPVPQDTKFIHTKANRFEEVAWSKYNPRDQLYLHIGLKPRV
RDHYRATKVAFWKHLVPHLYNLHDMFHYTSTTTKVPPPDTTHSSHITRRPNGKTW
STKRPAISPAYSNENAPGSWNGDQDAGPLLVENPRDYSTELSVTIAVGASLLFLNVL
AFAALYYRKDKRRQEPLRQPSPQRGTGAPELGTAPEEELAALQLGPTHHECEAGPP
HDTLRLTALPDYTLTLRRSPDDIPLMTPNTITMIPNSLVGLQTLHPYNTFAAGFNSTG
LPHSHSTTRV

C-HA-Nlgn3-mCherry
with signal peptide, HA tag and mCherry colored

- DNA sequence, ATGTGGCTGCAGCCCTCGCTGTCCCTGAGCCCCACGCCCACAGT
TGGCCGGAGCCTGTGCCTCACCCTGGGCTTCCTCAGTTTGGTGCTGAGGGCCAG
TACCTGTCAGTATCCATATGATGTTCCAGATTATGCTGCCCCGGCACCCACAGTC
AATACTCACTTTGGGAAGCTAAGGGGTGCCAGAGTACCATTGCCCAGTGAAATC
CTGGGTCCTGTGGACCAATACCTGGGGGTACCCTACGCAGCTCCCCCGATCGGC
GAGAAACGTTTCCTGCCCCCTGAACCACCCCCATCCTGGTCGGGCATCCGGAAC
GCCACACACTTTCCCCCAGTGTGCCCCCAGAACATCCACACAGCTGTGCCCGAA
GTCATGCTGCCAGTCTGGTTCACTGCCAACTTGGATATCGTCGCCACTTATATCC
AGGAGCCCAACGAAGATTGCCTCTATCTGAATGTGTATGTGCCCACGGAAGATG
GATCCGGCGCTAAGAAACAGGGCGAGGACTTAGCGGATAATGACGGGGATGAA
GATGAAGACATCCGAGACAGTGGTGCTAAACCTGTCATGGTCTACATCCACGGA
GGCTCTTACATGGAAGGAACAGGCAACATGATTGATGGCAGTGTTCTTGCAAGT
TACGGCAACGTCATCGTCATCACCCTCAACTATCGGGTCGGGGTGCTAGGTTTC
CTGAGCACTGGAGATCAGGCTGCCAAGGGCAACTATGGGCTCCTTGATCAAATC
CAGGCCCTTCGCTGGGTGAGTGAGAATATTGCCTTCTTTGGAGGAGATCCCCGT
AGAATTACTGTCTTTGGCTCTGGCATCGGTGCATCCTGTGTCAGTCTCCTTACAC
TGTCTCATCATTCTGAGGGGCTTTTCCAGAGGGCCATCATCCAAAGTGGCTCTGC
TCTATCTAGCTGGGCTGTGAACTACCAACCAGTGAAGTATACCAGCTTGCTGGC
AGACAAAGTGGGCTGTAACGTCCTGGACACTGTGGATATGGTGGATTGTCTTCG
ACAAAAGAGTGCCAAGGAGCTGGTAGAACAGGACATTCAGCCAGCCCGCTACC
ATGTGGCTTTTGGCCCTGTGATTGATGGTGATGTCATTCCTGATGACCCTGAGAT
CCTTATGGAGCAGGGAGAGTTCCTCAACTATGATATCATGCTAGGCGTCAACCA

GGGTGAGGGTCTCAAGTTTGTGGAAGGGGTGGTGGACCCCGAGGATGGTGTCTC
GGGCACTGACTTTGACTACTCTGTCTCCAATTTTGTGGACAATCTGTATGGCTAT
CCTGAGGGTAAGGACACCCTGCGGGAGACTATCAAGTTCATGTATACGGACTGG
GCAGACCGAGACAACCCTGAGACCCGCCGTAAAACACTGGTGGCACTCTTCACT
GACCACCAGTGGGTGGAGCCTTCAGTGGTGACAGCCGATCTGCACGCCCGCTAT
GGCTCACCTACCTACTTCTACGCCTTCTACCATCACTGCCAGAGCCTCATGAAGC
CCGCATGGTCAGATGCAGCACACGGGGATGAAGTGCCCTATGTTTTTGGTGTCC
CTATGGTAGGTCCCACTGACCTTTTCCCCTGCAACTTCTCCAAGAATGATGTTAT
GCTCAGTGCTGTCGTCATGACCTATTGGACCAACTTTGCCAAGACCGGGGATCC
CAACAAGCCGGTACCCCAGGATACCAAGTTCATTCACACCAAGGCCAACCGCTT
TGAGGAAGTGGCCTGGTCCAAATACAATCCCCGAGACCAGCTCTACCTTCACAT
CGGGCTGAAACCAAGGGTTCGTGATCATTACCGGGCCACAAAGGTAGCCTTTTG
GAAACACCTGGTGCCCCACCTGTACAACCTGCATGACATGTTCCACTATACATC
CACGACCACCAAAGTGCCGCCCCCGGACACCACCCACAGCTCCCACATCACCCG
TAGGCCCAACGGCAAGACCTGGAGCACCAAGCGGCCGGCGATTTCACCTGCCT
ACAGCAATGAGAATGCCCCTGGGTCCTGGAATGGGGACCAGGATGCGGGGCCA
CTCCTGGTTGAGAACCCTCGAGACTACTCCACTGAATTAAGTGTCACTATCGCTG
TGGGGGCCTCCCTCCTGTTTCTCAATGTGTTGGCCTTTGCTGCCCTCTATTACCGT
AAGGACAAACGGCGCCAGGAGCCCCTGAGGCAGCCTAGCCCCCAAAGGGGAAC
TGGTGCCCCTGAATTGGGAACTGCTCCGGAGGAGGAGCTGGCAGCATTACAGTT
GGGTCCCACTCACCATGAATGTGAGGCCGGTCCCCCACATGACACACTTCGCCT
CACAGCACTGCCCGACTATACCCTGACCCTGCGGCGCTCCCCTGATGACATCCC
ACTCATGACTCCCAACACCATCACTATGATTCCTAATTCCCTGGTTGGGTTGCAG
ACCTTGCACCCCTATAACACCTTTGCCGCAGGGTTCAACAGTACTGGGCTGCCC
CACTCACACTCCACTACCCGTGTATCTAGAGTGAGCAAGGGCGAGGAGGATAA
CATGGCCATCATCAAGGAGTTCATGCGCTTCAAGGTGCACATGGAGGGCTCCGT
GAACGGCCACGAGTTCGAGATCGAGGGCGAGGGCGAGGGCCGCCCCTACGAGG
GCACCCAGACCGCCAAGCTGAAGGTGACCAAGGGTGGCCCCCTGCCCTTCGCCT
GGGACATCCTGTCCCCTCAGTTCATGTACGGCTCCAAGGCCTACGTGAAGCACC
CCGCCGACATCCCCGACTACTTGAAGCTGTCCTTCCCCGAGGGCTTCAAGTGGG
AGCGCGTGATGAACTTCGAGGACGGCGGCGTGGTGACCGTGACCCAGGACTCC
TCCCTGCAGGACGGCGAGTTCATCTACAAGGTGAAGCTGCGCGGCACCAACTTC
CCCTCCGACGGCCCCGTAATGCAGAAGAAGACCATGGGCTGGGAGGCCTCCTCC
GAGCGGATGTACCCCGAGGACGGCGCCCTGAAGGGCGAGATCAAGCAGAGGCT
GAAGCTGAAGGACGGCGGCCACTACGACGCTGAGGTCAAGACCACCTACAAGG
CCAAGAAGCCCGTGCAGCTGCCCGGCGCCTACAACGTCAACATCAAGTTGGAC
ATCACCTCCCACAACGAGGACTACACCATCGTGGAACAGTACGAACGCGCCGA
GGGCCGCCACTCCACCGGCGGCATGGACGAGCTGTACAAG

- Amino acid sequence, MWLQPSLSLSPTPTVGRSLCLTLGFLSLVLRASTCQYPYDVP DYAAPAPTVNTHFGKLRGARVPLPSEILGPVDQYLGVPYAAPPIGEKRFLPPEPPPS WSGIRNATHFPPVCPQNIHTAVPEVMLPVWFTANLDIVATYIQEPNEDCLYLNVYV PTEDGSGAKKQGEDLADNDGDEDEDIRDSGAKPVMVYIHGGSYMEGTGNMIDGSV LASYGNVIVITLNYRVGVLGFLSTGDQAAKGNYGLLDQIQALRWVSENIAFFGGDP RRITVFGSGIGASCVSLLTLSHHSEGLFQRAIIQSGSALSSWAVNYQPVKYTSLLADK VGCNVLDTVDMVDCLRQKSAKELVEQDIQPARYHVAFGPVIDGDVIPDDPEILMEQ GEFLNYDIMLGVNQGEGLKFVEGVVDPEDGVSGTDFDYSVSNFVDNLYGYPEGKD

TLRETIKFMYTDWADRDNPETRRKTLVALFTDHQWVEPSVVTADLHARYGSPTYF
YAFYHHCQSLMKPAWSDAAHGDEVPYVFGVPMVGPTDLFPCNFSKNDVMLSAVV
MTYWTNFAKTGDPNKPVPQDTKFIHTKANRFEEVAWSKYNPRDQLYLHIGLKPRV
RDHYRATKVAFWKHLVPHLYNLHDMFHYTSTTTKVPPPDTTHSSHITRRPNGKTW
STKRPAISPAYSNENAPGSWNGDQDAGPLLVENPRDYSTELSVTIAVGASLLFLNVL
AFAALYYRKDKRRQEPLRQPSPQRGTGAPELGTAPEEELAALQLGPTHHECEAGPP
HDTLRLTALPDYTLTLRRSPDDIPLMTPNTITMIPNSLVGLQTLHPYNTFAAGFNSTG
LPHSHSTTRVSRVSKGEEDNMAIIKEFMRFKVHMEGSVNGHEFEIEGEGEGRPYEGT
QTAKLKVTKGGPLPFAWDILSPQFMYGSKAYVKHPADIPDYLKLSFPEGFKWERV
MNFEDGGVVTVTQDSSLQDGEFIYKVKLRGTNFPSDGPVMQKKTMGWEASSERM
YPEDGALKGEIKQRLKLKDGGHYDAEVKTTYKAKKPVQLPGAYNVNIKLDITSHN
EDYTIVEQYERAEGRHSTGGMDELYK

TAG-HA-Nlgn3
with signal peptide and HA tag colored

- This construct is used together with an orthogonal aminoacyl-tRNA synthetase/tRNA pair for incorporation of the corresponding unnatural amino acid in response to the amber (TAG) codon.
- DNA sequence, ATGTGGCTGCAGCCCTCGCTGTCCCTGAGCCCCACGCCCACAGTT
GGCCGGAGCCTGTGCCTCACCCTGGGCTTCCTCAGTTTGGTGCTGAGGGCCAGT
ACCTAGCAGTATCCATATGATGTTCCAGATTATGCTGCCCCGGCACCCACAGTC
AATACTCACTTTGGGAAGCTAAGGGGTGCCAGAGTACCATTGCCCAGTGAAATC
CTGGGTCCTGTGGACCAATACCTGGGGGTACCCTACGCAGCTCCCCCGATCGGC
GAGAAACGTTTCCTGCCCCCTGAACCACCCCCATCCTGGTCGGGCATCCGGAAC
GCCACACACTTTCCCCCAGTGTGCCCCCAGAACATCCACACAGCTGTGCCCGAA
GTCATGCTGCCAGTCTGGTTCACTGCCAACTTGGATATCGTCGCCACTTATATCC
AGGAGCCCAACGAAGATTGCCTCTATCTGAATGTGTATGTGCCCACGGAAGATG
GATCCGGCGCTAAGAAACAGGGCGAGGACTTAGCGGATAATGACGGGGATGAA
GATGAAGACATCCGAGACAGTGGTGCTAAACCTGTCATGGTCTACATCCACGGA
GGCTCTTACATGGAAGGAACAGGCAACATGATTGATGGCAGTGTTCTTGCAAGT
TACGGCAACGTCATCGTCATCACCCTCAACTATCGGGTCGGGGTGCTAGGTTTC
CTGAGCACTGGAGATCAGGCTGCCAAGGGCAACTATGGGCTCCTTGATCAAATC
CAGGCCCTTCGCTGGGTGAGTGAGAATATTGCCTTCTTTGGAGGAGATCCCCGT
AGAATTACTGTCTTTGGCTCTGGCATCGGTGCATCCTGTGTCAGTCTCCTTACAC
TGTCTCATCATTCTGAGGGGCTTTTCCAGAGGGCCATCATCCAAAGTGGCTCTGC
TCTATCTAGCTGGGCTGTGAACTACCAACCAGTGAAGTATACCAGCTTGCTGGC
AGACAAAGTGGGCTGTAACGTCCTGGACACTGTGGATATGGTGGATTGTCTTCG
ACAAAAGAGTGCCAAGGAGCTGGTAGAACAGGACATTCAGCCAGCCCGCTACC
ATGTGGCTTTTGGCCCTGTGATTGATGGTGATGTCATTCCTGATGACCCTGAGAT
CCTTATGGAGCAGGGAGAGTTCCTCAACTATGATATCATGCTAGGCGTCAACCA
GGGTGAGGGTCTCAAGTTTGTGGAAGGGGTGGTGGACCCCGAGGATGGTGTCTC
GGGCACTGACTTTGACTACTCTGTCTCCAATTTTGTGGACAATCTGTATGGCTAT
CCTGAGGGTAAGGACACCCTGCGGGAGACTATCAAGTTCATGTATACGGACTGG
GCAGACCGAGACAACCCTGAGACCCGCCGTAAAACACTGGTGGCACTCTTCACT
GACCACCAGTGGGTGGAGCCTTCAGTGGTGACAGCCGATCTGCACGCCCGCTAT
GGCTCACCTACCTACTTCTACGCCTTCTACCATCACTGCCAGAGCCTCATGAAGC

CCGCATGGTCAGATGCAGCACACGGGGATGAAGTGCCCTATGTTTTTGGTGTCC
CTATGGTAGGTCCCACTGACCTTTTCCCCTGCAACTTCTCCAAGAATGATGTTAT
GCTCAGTGCTGTCGTCATGACCTATTGGACCAACTTTGCCAAGACCGGGGATCC
CAACAAGCCGGTACCCCAGGATACCAAGTTCATTCACACCAAGGCCAACCGCTT
TGAGGAAGTGGCCTGGTCCAAATACAATCCCCGAGACCAGCTCTACCTTCACAT
CGGGCTGAAACCAAGGGTTCGTGATCATTACCGGGCCACAAAGGTAGCCTTTTG
GAAACACCTGGTGCCCCACCTGTACAACCTGCATGACATGTTCCACTATACATC
CACGACCACCAAAGTGCCGCCCCCGGACACCACCCACAGCTCCCACATCACCCG
TAGGCCCAACGGCAAGACCTGGAGCACCAAGCGGCCGGCGATTTCACCTGCCT
ACAGCAATGAGAATGCCCCTGGGTCCTGGAATGGGGACCAGGATGCGGGGCCA
CTCCTGGTTGAGAACCCTCGAGACTACTCCACTGAATTAAGTGTCACTATCGCTG
TGGGGGCCTCCCTCCTGTTTCTCAATGTGTTGGCCTTTGCTGCCCTCTATTACCGT
AAGGACAAACGGCGCCAGGAGCCCCTGAGGCAGCCTAGCCCCCAAAGGGGAAC
TGGTGCCCCTGAATTGGGAACTGCTCCGGAGGAGGAGCTGGCAGCATTACAGTT
GGGTCCCACTCACCATGAATGTGAGGCCGGTCCCCCACATGACACACTTCGCCT
CACAGCACTGCCCGACTATACCCTGACCCTGCGGCGCTCCCCTGATGACATCCC
ACTCATGACTCCCAACACCATCACTATGATTCCTAATTCCCTGGTTGGGTTGCAG
ACCTTGCACCCCTATAACACCTTTGCCGCAGGGTTCAACAGTACTGGGCTGCCC
CACTCACACTCCACTACCCGTGTA

- Amino acid sequence, MWLQPSLSLSPTPTVGRSLCLTLGFLSLVLRAST*****QYPYDVPDYAAPAPTVNTHFGKLRGARVPLPSEILGPVDQYLGVPYAAPPIGEKRFLPPEPPPSWSGIRNATHFPPVCPQNIHTAVPEVMLPVWFTANLDIVATYIQEPNEDCLYLNVYVPTEDGSGAKKQGEDLADNDGDEDEDIRDSGAKPVMVYIHGGSYMEGTGNMIDGSVLASYGNVIVITLNYRVGVLGFLSTGDQAAKGNYGLLDQIQALRWVSENIAFFGGDPRRITVFGSGIGASCVSLLTLSHHSEGLFQRAIIQSGSALSSWAVNYQPVKYTSLLADKVGCNVLDTVDMVDCLRQKSAKELVEQDIQPARYHVAFGPVIDGDVIPDDPEILMEQGEFLNYDIMLGVNQGEGLKFVEGVVDPEDGVSGTDFDYSVSNFVDNLYGYPEGKDTLRETIKFMYTDWADRDNPETRRKTLVALFTDHQWVEPSVVTADLHARYGSPTYFYAFYHHCQSLMKPAWSDAAHGDEVPYVFGVPMVGPTDLFPCNFSKNDVMLSAVVMTYWTNFAKTGDPNKPVPQDTKFIHTKANRFEEVAWSKYNPRDQLYLHIGLKPRVRDHYRATKVAFWKHLVPHLYNLHDMFHYTSTTTKVPPPDTTHSSHITRRPNGKTWSTKRPAISPAYSNENAPGSWNGDQDAGPLLVENPRDYSTELSVTIAVGASLLFLNVLAFAALYYRKDKRRQEPLRQPSPQRGTGAPELGTAPEEELAALQLGPTHHECEAGPPHDTLRLTALPDYTLTLRRSPDDIPLMTPNTITMIPNSLVGLQTLHPYNTFAAGFNSTGLPHSHSTTRV

TGA-HA-Nlgn3
with signal peptide and HA tag colored

- This construct is used together with EcTyrRS*/$tRNA_{ACU}$ pair for incorporation of **PPA** in response to the opal (UGA) codon on mRNA.
- DNA sequence: ATGTGGCTGCAGCCCTCGCTGTCCCTGAGCCCCACGCCCACAGTTGGCCGGAGCCTGTGCCTCACCCTGGGCTTCCTCAGTTTGGTGCTGAGGGCCAGTACCTGACAGTATCCATATGATGTTCCAGATTATGCTGCCCCGGCACCCACAGTCAATACTCACTTTGGGAAGCTAAGGGGTGCCAGAGTACCATTGCCCAGTGAAATCCTGGGTCCTGTGGACCAATACCTGGGGGTACCCTACGCAGCTCCCCCGATCGGCGAGAAACGTTTCCTGCCCCCTGAACCACCCCCATCCTGGTCGGGCATCCGGA

ACGCCACACACTTTCCCCCAGTGTGCCCCCAGAACATCCACACAGCTGTGCCCG
AAGTCATGCTGCCAGTCTGGTTCACTGCCAACTTGGATATCGTCGCCACTTATAT
CCAGGAGCCCAACGAAGATTGCCTCTATCTGAATGTGTATGTGCCCACGGAAGA
TGGATCCGGCGCTAAGAAACAGGGCGAGGACTTAGCGGATAATGACGGGGATG
AAGATGAAGACATCCGAGACAGTGGTGCTAAACCTGTCATGGTCTACATCCACG
GAGGCTCTTACATGGAAGGAACAGGCAACATGATTGATGGCAGTGTTCTTGCAA
GTTACGGCAACGTCATCGTCATCACCCTCAACTATCGGGTCGGGGTGCTAGGTT
TCCTGAGCACTGGAGATCAGGCTGCCAAGGGCAACTATGGGCTCCTTGATCAAA
TCCAGGCCCTTCGCTGGGTGAGTGAGAATATTGCCTTCTTTGGAGGAGATCCCC
GTAGAATTACTGTCTTTGGCTCTGGCATCGGTGCATCCTGTGTCAGTCTCCTTAC
ACTGTCTCATCATTCTGAGGGGCTTTTCCAGAGGGCCATCATCCAAAGTGGCTCT
GCTCTATCTAGCTGGGCTGTGAACTACCAACCAGTGAAGTATACCAGCTTGCTG
GCAGACAAAGTGGGCTGTAACGTCCTGGACACTGTGGATATGGTGGATTGTCTT
CGACAAAAGAGTGCCAAGGAGCTGGTAGAACAGGACATTCAGCCAGCCCGCTA
CCATGTGGCTTTTGGCCCTGTGATTGATGGTGATGTCATTCCTGATGACCCTGAG
ATCCTTATGGAGCAGGGAGAGTTCCTCAACTATGATATCATGCTAGGCGTCAAC
CAGGGTGAGGGTCTCAAGTTTGTGGAAGGGGTGGTGGACCCCGAGGATGGTGT
CTCGGGCACTGACTTTGACTACTCTGTCTCCAATTTTGTGGACAATCTGTATGGC
TATCCTGAGGGTAAGGACACCCTGCGGGAGACTATCAAGTTCATGTATACGGAC
TGGGCAGACCGAGACAACCCTGAGACCCGCCGTAAAACACTGGTGGCACTCTTC
ACTGACCACCAGTGGGTGGAGCCTTCAGTGGTGACAGCCGATCTGCACGCCCGC
TATGGCTCACCTACCTACTTCTACGCCTTCTACCATCACTGCCAGAGCCTCATGA
AGCCCGCATGGTCAGATGCAGCACACGGGGATGAAGTGCCCTATGTTTTTGGTG
TCCCTATGGTAGGTCCCACTGACCTTTTCCCCTGCAACTTCTCCAAGAATGATGT
TATGCTCAGTGCTGTCGTCATGACCTATTGGACCAACTTTGCCAAGACCGGGGA
TCCCAACAAGCCGGTACCCCAGGATACCAAGTTCATTCACACCAAGGCCAACCG
CTTTGAGGAAGTGGCCTGGTCCAAATACAATCCCCGAGACCAGCTCTACCTTCA
CATCGGGCTGAAACCAAGGGTTCGTGATCATTACCGGGCCACAAAGGTAGCCTT
TTGGAAACACCTGGTGCCCCACCTGTACAACCTGCATGACATGTTCCACTATAC
ATCCACGACCACCAAAGTGCCGCCCCCGGACACCACCCACAGCTCCCACATCAC
CCGTAGGCCCAACGGCAAGACCTGGAGCACCAAGCGGCCGGCGATTTCACCTG
CCTACAGCAATGAGAATGCCCCTGGGTCCTGGAATGGGGACCAGGATGCGGGG
CCACTCCTGGTTGAGAACCCTCGAGACTACTCCACTGAATTAAGTGTCACTATC
GCTGTGGGGGCCTCCCTCCTGTTTCTCAATGTGTTGGCCTTTGCTGCCCTCTATTA
CCGTAAGGACAAACGGCGCCAGGAGCCCCTGAGGCAGCCTAGCCCCCAAAGGG
GAACTGGTGCCCCTGAATTGGGAACTGCTCCGGAGGAGGAGCTGGCAGCATTAC
AGTTGGGTCCCACTCACCATGAATGTGAGGCCGGTCCCCCACATGACACACTTC
GCCTCACAGCACTGCCCGACTATACCCTGACCCTGCGGCGCTCCCCTGATGACA
TCCCACTCATGACTCCCAACACCATCACTATGATTCCTAATTCCCTGGTTGGGTT
GCAGACCTTGCACCCCTATAACACCTTTGCCGCAGGGTTCAACAGTACTGGGCT
GCCCCACTCACACTCCACTACCCGTGTA

- Amino acid sequence: MWLQPSLSLSPTPTVGRSLCLTLGFLSLVLRAST*QYPYDVPDYAAPAPTVNTHFGKLRGARVPLPSEILGPVDQYLGVPYAAPPIGEKRFLPPEPPPSWSGIRNATHFPPVCPQNIHTAVPEVMLPVWFTANLDIVATYIQEPNEDCLYLNVYVPTEDGSGAKKQGEDLADNDGDEDEDIRDSGAKPVMVYIHGGSYMEGTGNMIDGSVLASYGNVIVITLNYRVGVLGFLSTGDQAAKGNYGLLDQIQALRWVSENIAFFGGDP

RRITVFGSGIGASCVSLLTLSHHSEGLFQRAIIQSGSALSSWAVNYQPVKYTSLLADK
VGCNVLDTVDMVDCLRQKSAKELVEQDIQPARYHVAFGPVIDGDVIPDDPEILMEQ
GEFLNYDIMLGVNQGEGLKFVEGVVDPEDGVSGTDFDYSVSNFVDNLYGYPEGKD
TLRETIKFMYTDWADRDNPETRRKTLVALFTDHQWVEPSVVTADLHARYGSPTYF
YAFYHHCQSLMKPAWSDAAHGDEVPYVFGVPMVGPTDLFPCNFSKNDVMLSAVV
MTYWTNFAKTGDPNKPVPQDTKFIHTKANRFEEVAWSKYNPRDQLYLHIGLKPRV
RDHYRATKVAFWKHLVPHLYNLHDMFHYTSTTTKVPPPDTTHSSHITRRPNGKTW
STKRPAISPAYSNENAPGSWNGDQDAGPLLVENPRDYSTELSVTIAVGASLLFLNVL
AFAALYYRKDKRRQEPLRQPSPQRGTGAPELGTAPEEELAALQLGPTHHECEAGPP
HDTLRLTALPDYTLTLRRSPDDIPLMTPNTITMIPNSLVGLQTLHPYNTFAAGFNSTG
LPHSHSTTRV

AGGA-HA-Nlgn3
with signal peptide and HA tag colored

- This construct is used together with *Mm*PylRS*/tRNA$_{UCCU}$ pair for incorporation of **BCNK** in response to quadruplet codon AGGA on mRNA.
- DNA sequence, ATGTGGCTGCAGCCCTCGCTGTCCCTGAGCCCCACGCCCACAGT
TGGCCGGAGCCTGTGCCTCACCCTGGGCTTCCTCAGTTTGGTGCTGAGGGCCAG
TACCAGGACAGTATCCATATGATGTTCCAGATTATGCTGCCCCGGCACCCACAG
TCAATACTCACTTTGGGAAGCTAAGGGGTGCCAGAGTACCATTGCCCAGTGAAA
TCCTGGGTCCTGTGGACCAATACCTGGGGGTACCCTACGCAGCTCCCCCGATCG
GCGAGAAACGTTTCCTGCCCCCTGAACCACCCCCATCCTGGTCGGGCATCCGGA
ACGCCACACACTTTCCCCCAGTGTGCCCCCAGAACATCCACACAGCTGTGCCCG
AAGTCATGCTGCCAGTCTGGTTCACTGCCAACTTGGATATCGTCGCCACTTATAT
CCAGGAGCCCAACGAAGATTGCCTCTATCTGAATGTGTATGTGCCCACGGAAGA
TGGATCCGGCGCTAAGAAACAGGGCGAGGACTTAGCGGATAATGACGGGGATG
AAGATGAAGACATCCGAGACAGTGGTGCTAAACCTGTCATGGTCTACATCCACG
GAGGCTCTTACATGGAAGGAACAGGCAACATGATTGATGGCAGTGTTCTTGCAA
GTTACGGCAACGTCATCGTCATCACCCTCAACTATCGGGTCGGGGTGCTAGGTT
TCCTGAGCACTGGAGATCAGGCTGCCAAGGGCAACTATGGGCTCCTTGATCAAA
TCCAGGCCCTTCGCTGGGTGAGTGAGAATATTGCCTTCTTTGGAGGAGATCCCC
GTAGAATTACTGTCTTTGGCTCTGGCATCGGTGCATCCTGTGTCAGTCTCCTTAC
ACTGTCTCATCATTCTGAGGGGCTTTTCCAGAGGGCCATCATCCAAAGTGGCTCT
GCTCTATCTAGCTGGGCTGTGAACTACCAACCAGTGAAGTATACCAGCTTGCTG
GCAGACAAAGTGGGCTGTAACGTCCTGGACACTGTGGATATGGTGGATTGTCTT
CGACAAAAGAGTGCCAAGGAGCTGGTAGAACAGGACATTCAGCCAGCCCGCTA
CCATGTGGCTTTTGGCCCTGTGATTGATGGTGATGTCATTCCTGATGACCCTGAG
ATCCTTATGGAGCAGGGAGAGTTCCTCAACTATGATATCATGCTAGGCGTCAAC
CAGGGTGAGGGTCTCAAGTTTGTGGAAGGGGTGGTGGACCCCGAGGATGGTGT
CTCGGGCACTGACTTTGACTACTCTGTCTCCAATTTTGTGGACAATCTGTATGGC
TATCCTGAGGGTAAGGACACCCTGCGGGAGACTATCAAGTTCATGTATACGGAC
TGGGCAGACCGAGACAACCCTGAGACCCGCCGTAAAACACTGGTGGCACTCTTC
ACTGACCACCAGTGGGTGGAGCCTTCAGTGGTGACAGCCGATCTGCACGCCCGC
TATGGCTCACCTACCTACTTCTACGCCTTCTACCATCACTGCCAGAGCCTCATGA
AGCCCGCATGGTCAGATGCAGCACACGGGGATGAAGTGCCCTATGTTTTTGGTG
TCCCTATGGTAGGTCCCACTGACCTTTTCCCCTGCAACTTCTCCAAGAATGATGT

TATGCTCAGTGCTGTCGTCATGACCTATTGGACCAACTTTGCCAAGACCGGGGA TCCCAACAAGCCGGTACCCCAGGATACCAAGTTCATTCACACCAAGGCCAACCG CTTTGAGGAAGTGGCCTGGTCCAAATACAATCCCCGAGACCAGCTCTACCTTCA CATCGGGCTGAAACCAAGGGTTCGTGATCATTACCGGGCCACAAAGGTAGCCTT TTGGAAACACCTGGTGCCCCACCTGTACAACCTGCATGACATGTTCCACTATAC ATCCACGACCACCAAAGTGCCGCCCCCGGACACCACCCACAGCTCCCACATCAC CCGTAGGCCCAACGGCAAGACCTGGAGCACCAAGCGGCCGGCGATTTCACCTG CCTACAGCAATGAGAATGCCCCTGGGTCCTGGAATGGGGACCAGGATGCGGGG CCACTCCTGGTTGAGAACCCTCGAGACTACTCCACTGAATTAAGTGTCACTATC GCTGTGGGGGCCTCCCTCCTGTTTCTCAATGTGTTGGCCTTTGCTGCCCTCTATTA CCGTAAGGACAAACGGCGCCAGGAGCCCCTGAGGCAGCCTAGCCCCCAAAGGG GAACTGGTGCCCCTGAATTGGGAACTGCTCCGGAGGAGGAGCTGGCAGCATTAC AGTTGGGTCCCACTCACCATGAATGTGAGGCCGGTCCCCCACATGACACACTTC GCCTCACAGCACTGCCCGACTATACCCTGACCCTGCGGCGCTCCCCTGATGACA TCCCACTCATGACTCCCAACACCATCACTATGATTCCTAATTCCCTGGTTGGGTT GCAGACCTTGCACCCCTATAACACCTTTGCCGCAGGGTTCAACAGTACTGGGCT GCCCCACTCACACTCCACTACCCGTGTA

- Amino acid sequence, MWLQPSLSLSPTPTVGRSLCLTLGFLSLVLRAST(**BCNK**)QYP YDVPDYAAPAPTVNTHFGKLRGARVPLPSEILGPVDQYLGVPYAAPPIGEKRFLPPE PPPSWSGIRNATHFPPVCPQNIHTAVPEVMLPVWFTANLDIVATYIQEPNEDCLYLN VYVPTEDGSGAKKQGEDLADNDGDEDEDIRDSGAKPVMVYIHGGSYMEGTGNMI DGSVLASYGNVIVITLNYRVGVLGFLSTGDQAAKGNYGLLDQIQALRWVSENIAFF GGDPRRITVFGSGIGASCVSLLTLSHHSEGLFQRAIIQSGSALSSWAVNYQPVKYTSL LADKVGCNVLDTVDMVDCLRQKSAKELVEQDIQPARYHVAFGPVIDGDVIPDDPEI LMEQGEFLNYDIMLGVNQGEGLKFVEGVVDPEDGVSGTDFDYSVSNFVDNLYGYP EGKDTLRETIKFMYTDWADRDNPETRRKTLVALFTDHQWVEPSVVTADLHARYGS PTYFYAFYHHCQSLMKPAWSDAAHGDEVPYVFGVPMVGPTDLFPCNFSKNDVML SAVVMTYWTNFAKTGDPNKPVPQDTKFIHTKANRFEEVAWSKYNPRDQLYLHIGL KPRVRDHYRATKVAFWKHLVPHLYNLHDMFHYTSTTTKVPPPDTTHSSHITRRPNG KTWSTKRPAISPAYSNENAPGSWNGDQDAGPLLVENPRDYSTELSVTIAVGASLLFL NVLAFAALYYRKDKRRQEPLRQPSPQRGTGAPELGTAPEEELAALQLGPTHHECEA GPPHDTLRLTALPDYTLTLRRSPDDIPLMTPNTITMIPNSLVGLQTLHPYNTFAAGFN STGLPHSHSTTRV

IDGR-C-HA-Nlgn3
with signal peptide, Factor Xa recognition sequence and HA tag colored

- Treatment with Factor Xa protease affords a protein with N-terminal cysteine for TAMM labeling.
- DNA sequence, ATGTGGCTGCAGCCCTCGCTGTCCCTGAGCCCCACGCCCACA GTTGGCCGGAGCCTGTGCCTCACCCTGGGCTTCCTCAGTTTGGTGCTGAGGGCC AGTACCCAGGACTACAAGGACGACGACGACAAGGGCTCTTCTGGCAGCTCTATC GACGGCAGATGCTATCCATATGATGTTCCAGATTATGCTGCCCCGGCACCCACA GTCAATACTCACTTTGGGAAGCTAAGGGGTGCCAGAGTACCATTGCCCAGTGAA ATCCTGGGTCCTGTGGACCAATACCTGGGGGTACCCTACGCAGCTCCCCCGATC GGCGAGAAACGTTTCCTGCCCCCTGAACCACCCCCATCCTGGTCGGGCATCCGG AACGCCACACACTTTCCCCCAGTGTGCCCCCAGAACATCCACACAGCTGTGCCC

GAAGTCATGCTGCCAGTCTGGTTCACTGCCAACTTGGATATCGTCGCCACTTATA
TCCAGGAGCCCAACGAAGATTGCCTCTATCTGAATGTGTATGTGCCCACGGAAG
ATGGATCCGGCGCTAAGAAACAGGGCGAGGACTTAGCGGATAATGACGGGGAT
GAAGATGAAGACATCCGAGACAGTGGTGCTAAACCTGTCATGGTCTACATCCAC
GGAGGCTCTTACATGGAAGGAACAGGCAACATGATTGATGGCAGTGTTCTTGCA
AGTTACGGCAACGTCATCGTCATCACCCTCAACTATCGGGTCGGGGTGCTAGGT
TTCCTGAGCACTGGAGATCAGGCTGCCAAGGGCAACTATGGGCTCCTTGATCAA
ATCCAGGCCCTTCGCTGGGTGAGTGAGAATATTGCCTTCTTTGGAGGAGATCCC
CGTAGAATTACTGTCTTTGGCTCTGGCATCGGTGCATCCTGTGTCAGTCTCCTTA
CACTGTCTCATCATTCTGAGGGGCTTTTCCAGAGGGCCATCATCCAAAGTGGCTC
TGCTCTATCTAGCTGGGCTGTGAACTACCAACCAGTGAAGTATACCAGCTTGCT
GGCAGACAAAGTGGGCTGTAACGTCCTGGACACTGTGGATATGGTGGATTGTCT
TCGACAAAAGAGTGCCAAGGAGCTGGTAGAACAGGACATTCAGCCAGCCCGCT
ACCATGTGGCTTTTGGCCCTGTGATTGATGGTGATGTCATTCCTGATGACCCTGA
GATCCTTATGGAGCAGGGAGAGTTCCTCAACTATGATATCATGCTAGGCGTCAA
CCAGGGTGAGGGTCTCAAGTTTGTGGAAGGGGTGGTGGACCCCGAGGATGGTG
TCTCGGGCACTGACTTTGACTACTCTGTCTCCAATTTTGTGGACAATCTGTATGG
CTATCCTGAGGGTAAGGACACCCTGCGGGAGACTATCAAGTTCATGTATACGGA
CTGGGCAGACCGAGACAACCCTGAGACCCGCCGTAAAACACTGGTGGCACTCTT
CACTGACCACCAGTGGGTGGAGCCTTCAGTGGTGACAGCCGATCTGCACGCCCG
CTATGGCTCACCTACCTACTTCTACGCCTTCTACCATCACTGCCAGAGCCTCATG
AAGCCCGCATGGTCAGATGCAGCACACGGGGATGAAGTGCCCTATGTTTTTGGT
GTCCCTATGGTAGGTCCCACTGACCTTTTCCCCTGCAACTTCTCCAAGAATGATG
TTATGCTCAGTGCTGTCGTCATGACCTATTGGACCAACTTTGCCAAGACCGGGG
ATCCCAACAAGCCGGTACCCCAGGATACCAAGTTCATTCACACCAAGGCCAACC
GCTTTGAGGAAGTGGCCTGGTCCAAATACAATCCCCGAGACCAGCTCTACCTTC
ACATCGGGCTGAAACCAAGGGTTCGTGATCATTACCGGGCCACAAAGGTAGCCT
TTTGGAAACACCTGGTGCCCCACCTGTACAACCTGCATGACATGTTCCACTATA
CATCCACGACCACCAAAGTGCCGCCCCCGGACACCACCCACAGCTCCCACATCA
CCCGTAGGCCCAACGGCAAGACCTGGAGCACCAAGCGGCCGGCGATTTCACCT
GCCTACAGCAATGAGAATGCCCCTGGGTCCTGGAATGGGGACCAGGATGCGGG
GCCACTCCTGGTTGAGAACCCTCGAGACTACTCCACTGAATTAAGTGTCACTAT
CGCTGTGGGGGCCTCCCTCCTGTTTCTCAATGTGTTGGCCTTTGCTGCCCTCTATT
ACCGTAAGGACAAACGGCGCCAGGAGCCCCTGAGGCAGCCTAGCCCCCAAAGG
GGAACTGGTGCCCCTGAATTGGGAACTGCTCCGGAGGAGGAGCTGGCAGCATT
ACAGTTGGGTCCCACTCACCATGAATGTGAGGCCGGTCCCCCACATGACACACT
TCGCCTCACAGCACTGCCCGACTATACCCTGACCCTGCGGCGCTCCCCTGATGA
CATCCCACTCATGACTCCCAACACCATCACTATGATTCCTAATTCCCTGGTTGGG
TTGCAGACCTTGCACCCCTATAACACCTTTGCCGCAGGGTTCAACAGTACTGGG
CTGCCCCACTCACACTCCACTACCCGTGTA

- Amino acid sequence, MWLQPSLSLSPTPTVGRSLCLTLGFLSLVLRASTQDYKDD DDKGSSGSSIDGRCYPYDVPDYAAPAPTVNTHFGKLRGARVPLPSEILGPVDQYLG VPYAAPPIGEKRFLPPEPPPSWSGIRNATHFPPVCPQNIHTAVPEVMLPVWFTANLDI VATYIQEPNEDCLYLNVYVPTEDGSGAKKQGEDLADNDGDEDEDIRDSGAKPVMV YIHGGSYMEGTGNMIDGSVLASYGNVIVITLNYRVGVLGFLSTGDQAAKGNYGLL DQIQALRWVSENIAFFGGDPRRITVFGSGIGASCVSLLTLSHHSEGLFQRAIIQSGSAL

SSWAVNYQPVKYTSLLADKVGCNVLDTVDMVDCLRQKSAKELVEQDIQPARYHV
AFGPVIDGDVIPDDPEILMEQGEFLNYDIMLGVNQGEGLKFVEGVVDPEDGVSGTD
FDYSVSNFVDNLYGYPEGKDTLRETIKFMYTDWADRDNPETRRKTLVALFTDHQW
VEPSVVTADLHARYGSPTYFYAFYHHCQSLMKPAWSDAAHGDEVPYVFGVPMVG
PTDLFPCNFSKNDVMLSAVVMTYWTNFAKTGDPNKPVPQDTKFIHTKANRFEEVA
WSKYNPRDQLYLHIGLKPRVRDHYRATKVAFWKHLVPHLYNLHDMFHYTSTTTK
VPPPDTTHSSHITRRPNGKTWSTKRPAISPAYSNENAPGSWNGDQDAGPLLVENPRD
YSTELSVTIAVGASLLFLNVLAFAALYYRKDKRRQEPLRQPSPQRGTGAPELGTAPE
EELAALQLGPTHHECEAGPPHDTLRLTALPDYTLTLRRSPDDIPLMTPNTITMIPNSL
VGLQTLHPYNTFAAGFNSTGLPHSHSTTRV

DDDDK-C-HA-Nlgn3
with signal peptide, enterokinase recognition sequence and HA tag colored

- Treatment with enterokinase (EK) affords a protein with N-terminal cysteine for TAMM labeling.
- DNA sequence, ATGTGGCTGCAGCCCTCGCTGTCCCTGAGCCCCACGCCCACAGT
TGGCCGGAGCCTGTGCCTCACCCTGGGCTTCCTCAGTTTGGTGCTGAGGGCCAG
TACCCAGGACTACAAGGACGACGACGACAAGTGTTATCCATATGATGTTCCAGA
TTATGCTGCCCCGGCACCCACAGTCAATACTCACTTTGGGAAGCTAAGGGGTGC
CAGAGTACCATTGCCCAGTGAAATCCTGGGTCCTGTGGACCAATACCTGGGGGT
ACCCTACGCAGCTCCCCCGATCGGCGAGAAACGTTTCCTGCCCCCTGAACCACC
CCCATCCTGGTCGGGCATCCGGAACGCCACACACTTTCCCCCAGTGTGCCCCCA
GAACATCCACACAGCTGTGCCCGAAGTCATGCTGCCAGTCTGGTTCACTGCCAA
CTTGGATATCGTCGCCACTTATATCCAGGAGCCCAACGAAGATTGCCTCTATCTG
AATGTGTATGTGCCCACGGAAGATGGATCCGGCGCTAAGAAACAGGGCGAGGA
CTTAGCGGATAATGACGGGGATGAAGATGAAGACATCCGAGACAGTGGTGCTA
AACCTGTCATGGTCTACATCCACGGAGGCTCTTACATGGAAGGAACAGGCAACA
TGATTGATGGCAGTGTTCTTGCAAGTTACGGCAACGTCATCGTCATCACCCTCAA
CTATCGGGTCGGGGTGCTAGGTTTCCTGAGCACTGGAGATCAGGCTGCCAAGGG
CAACTATGGGCTCCTTGATCAAATCCAGGCCCTTCGCTGGGTGAGTGAGAATAT
TGCCTTCTTTGGAGGAGATCCCCGTAGAATTACTGTCTTTGGCTCTGGCATCGGT
GCATCCTGTGTCAGTCTCCTTACACTGTCTCATCATTCTGAGGGGCTTTTCCAGA
GGGCCATCATCCAAAGTGGCTCTGCTCTATCTAGCTGGGCTGTGAACTACCAAC
CAGTGAAGTATACCAGCTTGCTGGCAGACAAAGTGGGCTGTAACGTCCTGGACA
CTGTGGATATGGTGGATTGTCTTCGACAAAAGAGTGCCAAGGAGCTGGTAGAAC
AGGACATTCAGCCAGCCCGCTACCATGTGGCTTTTGGCCCTGTGATTGATGGTG
ATGTCATTCCTGATGACCCTGAGATCCTTATGGAGCAGGGAGAGTTCCTCAACT
ATGATATCATGCTAGGCGTCAACCAGGGTGAGGGTCTCAAGTTTGTGGAAGGGG
TGGTGGACCCCGAGGATGGTGTCTCGGGCACTGACTTTGACTACTCTGTCTCCA
ATTTTGTGGACAATCTGTATGGCTATCCTGAGGGTAAGGACACCCTGCGGGAGA
CTATCAAGTTCATGTATACGGACTGGGCAGACCGAGACAACCCTGAGACCCGCC
GTAAAACACTGGTGGCACTCTTCACTGACCACCAGTGGGTGGAGCCTTCAGTGG
TGACAGCCGATCTGCACGCCCGCTATGGCTCACCTACCTACTTCTACGCCTTCTA
CCATCACTGCCAGAGCCTCATGAAGCCCGCATGGTCAGATGCAGCACACGGGG
ATGAAGTGCCCTATGTTTTTGGTGTCCCTATGGTAGGTCCCACTGACCTTTTCCC
CTGCAACTTCTCCAAGAATGATGTTATGCTCAGTGCTGTCGTCATGACCTATTGG

ACCAACTTTGCCAAGACCGGGGATCCCAACAAGCCGGTACCCCAGGATACCAA
GTTCATTCACACCAAGGCCAACCGCTTTGAGGAAGTGGCCTGGTCCAAATACAA
TCCCCGAGACCAGCTCTACCTTCACATCGGGCTGAAACCAAGGGTTCGTGATCA
TTACCGGGCCACAAAGGTAGCCTTTTGGAAACACCTGGTGCCCCACCTGTACAA
CCTGCATGACATGTTCCACTATACATCCACGACCACCAAAGTGCCGCCCCCGGA
CACCACCCACAGCTCCCACATCACCCGTAGGCCCAACGGCAAGACCTGGAGCA
CCAAGCGGCCGGCGATTTCACCTGCCTACAGCAATGAGAATGCCCCTGGGTCCT
GGAATGGGGACCAGGATGCGGGGCCACTCCTGGTTGAGAACCCTCGAGACTAC
TCCACTGAATTAAGTGTCACTATCGCTGTGGGGGCCTCCCTCCTGTTTCTCAATG
TGTTGGCCTTTGCTGCCCTCTATTACCGTAAGGACAAACGGCGCCAGGAGCCCC
TGAGGCAGCCTAGCCCCCAAAGGGGAACTGGTGCCCCTGAATTGGGAACTGCTC
CGGAGGAGGAGCTGGCAGCATTACAGTTGGGTCCCACTCACCATGAATGTGAG
GCCGGTCCCCCACATGACACACTTCGCCTCACAGCACTGCCCGACTATACCCTG
ACCCTGCGGCGCTCCCCTGATGACATCCCACTCATGACTCCCAACACCATCACT
ATGATTCCTAATTCCCTGGTTGGGTTGCAGACCTTGCACCCCTATAACACCTTTG
CCGCAGGGTTCAACAGTACTGGGCTGCCCCACTCACACTCCACTACCCGTGTAT
AA

- Amino acid sequence, MWLQPSLSLSPTPTVGRSLCLTLGFLSLVLRASTQDYKDDDDK CYPYDVPDYAAPAPTVNTHFGKLRGARVPLPSEILGPVDQYLGVPYAAPPIGEKRFL PPEPPPSWSGIRNATHFPPVCPQNIHTAVPEVMLPVWFTANLDIVATYIQEPNEDCLY LNVYVPTEDGSGAKKQGEDLADNDGDEDEDIRDSGAKPVMVYIHGGSYMEGTGN MIDGSVLASYGNVIVITLNYRVGVLGFLSTGDQAAKGNYGLLDQIQALRWVSENIA FFGGDPRRITVFGSGIGASCVSLLTLSHHSEGLFQRAIIQSGSALSSWAVNYQPVKYT SLLADKVGCNVLDTVDMVDCLRQKSAKELVEQDIQPARYHVAFGPVIDGDVIPDD PEILMEQGEFLNYDIMLGVNQGEGLKFVEGVVDPEDGVSGTDFDYSVSNFVDNLY GYPEGKDTLRETIKFMYTDWADRDNPETRRKTLVALFTDHQWVEPSVVTADLHAR YGSPTYFYAFYHHCQSLMKPAWSDAAHGDEVPYVFGVPMVGPTDLFPCNFSKNDV MLSAVVMTYWTNFAKTGDPNKPVPQDTKFIHTKANRFEEVAWSKYNPRDQLYLHI GLKPRVRDHYRATKVAFWKHLVPHLYNLHDMFHYTSTTTKVPPPDTTHSSHITRRP NGKTWSTKRPAISPAYSNENAPGSWNGDQDAGPLLVENPRDYSTELSVTIAVGASL LFLNVLAFAALYYRKDKRRQEPLRQPSPQRGTGAPELGTAPEEELAALQLGPTHHE CEAGPPHDTLRLTALPDYTLTLRRSPDDIPLMTPNTITMIPNSLVGLQTLHPYNTFAA GFNSTGLPHSHSTTRV

TAG-HA-CHRM3
with signal peptide and HA tag colored

- This construct is used together with an orthogonal aminoacyl-tRNA synthetase/tRNA pair for incorporation of the corresponding unnatural amino acid in response to the amber (TAG) codon.
- DNA sequence, ATGGCCTTACCAGTGACCGCCTTGCTCCTGCCGCTGGCCTTGCT GCTCCACGCCGCCAGGCCGTAGTATCCATATGATGTTCCAGATTATGCTACGCT GCATAATAATTCAACCACAAGCCCTCTGTTCCCTAACATCAGTAGTAGCTGGAT ACACTCTCCTAGCGACGCCGGACTTCCTCCCGGAACCGTGACCCACTTTGGGTC ATATAACGTCTCTCGGGCCGCAGGCAATTTTTCTTCCCCAGATGGGACAACCGA CGATCCGCTCGGCGGCCACACTGTATGGCAGGTGGTTTTCATAGCCTTTCTGACC GGCATTCTGGCTCTGGTCACAATCATCGGCAATATTCTGGTAATTGTGTCCTTCA

AGGTAAACAAACAACTGAAGACTGTCAATAACTACTTTCTGCTTAGTTTGGCAT
GCGCCGATCTGATTATCGGAGTGATAAGTATGAATCTCTTCACTACTTACATCAT
TATGAACAGATGGGCTCTGGGTAACTTGGCTTGCGACCTGTGGCTCGCAATTGA
CTATGTCGCAAGCAACGCCAGTGTGATGAACCTTCTGGTTATTAGCTTCGACCG
CTATTTTAGCATTACACGCCCTCTGACCTACAGAGCGAAACGCACTACAAAGCG
GGCAGGAGTTATGATCGGCCTGGCCTGGGTCATTTCCTTCGTGCTCTGGGCACC
AGCCATCTTGTTCTGGCAGTATTTCGTCGGTAAGAGGACAGTCCCACCAGGCGA
GTGCTTCATCCAATTTTTGTCCGAGCCAACCATAACATTCGGCACAGCCATCGC
GGCATTTTACATGCCAGTGACTATCATGACCATTCTGTACTGGAGAATCTACAA
AGAAACCGAAAAGCGGACAAAGGAGTTGGCAGGGCTTCAGGCATCAGGCACAG
AGGCTGAAACAGAAAACTTCGTGCACCCAACAGGGAGTTCACGCAGTTGCAGC
TCCTACGAGCTGCAGCAGCAGAGTATGAAGCGCTCAAACAGAAGAAAGTACGG
CAGGTGCCACTTTTGGTTTACTACAAAGAGCTGGAAGCCAAGCTCTGAACAGAT
GGATCAAGATCATAGCTCATCAGACTCATGGAATAATAATGACGCCGCCGCTAG
TCTGGAGAACAGTGCCAGTAGCGATGAGGAGGACATCGGTAGTGAAACCAGGG
CGATATATAGTATCGTGCTTAAGCTGCCAGGTCATTCAACTATACTGAATTCTAC
CAAACTTCCCAGTAGCGATAATCTTCAGGTGCCTGAGGAGGAGCTTGGCATGGT
CGACTTGGAGCGCAAGGCGGATAAACTGCAGGCTCAGAAGTCAGTTGACGATG
GAGGCTCTTTCCCTAAGTCATTTTCCAAACTCCCAATCCAGTTGGAGAGCGCCGT
CGACACCGCAAAAACGAGCGATGTGAATTCATCCGTTGGCAAAAGCACAGCAA
CTTTGCCTCTTTCCTTCAAAGAGGCCACACTCGCTAAGCGATTCGCACTCAAAAC
CCGGTCACAGATAACCAAGCGCAAGCGAATGTCACTCGTGAAAGAAAAAAAAG
CAGCACAGACATTGAGCGCCATCTTGCTCGCTTTTATCATAACATGGACACCCT
ACAATATCATGGTCCTCGTGAACACTTTCTGCGACTCCTGTATTCCTAAGACTTT
CTGGAACCTCGGCTACTGGCTGTGCTATATCAACAGTACAGTTAACCCTGTGTG
CTACGCTCTCTGTAACAAGACCTTTAGGACCACGTTCAAGATGCTCCTCCTGTGC
CAGTGTGACAAAAAGAAACGACGAAAGCAGCAATATCAGCAGCGACAGAGTGT
GATCTTTCATAAACGGGCGCCAGAACAAGCGCTCATCGATACCGGTGGACGCAC
CCCACCCAGCCTGGGTCCCCAAGATGAGTCCTGCACCACCGCCAGCTCCTCCCT
GGCCAAGGACACTTCATCGACCGGTGAGAACCTGTACTTCCAGCTAAGATTAGA
TAAAAGTAAAGTGATTAACAGCGCATTAGAGCTGCTTAATGAGGTCGGAATCGA
AGGTTTAACAACCCGTAAACTCGCCCAGAAGCTAGGTGTAGAGCAGCCTACATT
GTATTGGCATGTAAAAAATAAGCGGGCTTTGCTCGACGCCTTAGCCATTGAGAT
GTTAGATAGGCACCATACTCACTTTTGCCCTTTAGAAGGGGAAAGCTGGCAAGA
TTTTTTACGTAATAACGCTAAAAGTTTTAGATGTGCTTTACTAAGTCATCGCGAT
GGAGCAAAAGTACATTTAGGTACACGGCCTACAGAAAAACAGTATGAAACTCT
CGAAAATCAATTAGCCTTTTTATGCCAACAAGGTTTTTCACTAGAGAATGCATTA
TATGCACTCAGCGCTGTGGGGCATTTTACTTTAGGTTGCGTATTGGAAGATCAA
GAGCATCAAGTCGCTAAAGAAGAAAGGGAAACACCTACTACTGATAGTATGCC
GCCATTATTACGACAAGCTATCGAATTATTTGATCACCAAGGTGCAGAGCCAGC
CTTCTTATTCGGCCTTGAATTGATCATATGCGGATTAGAAAAACAACTTAAATGT
GAAAGTGGGTCCGCGTACAGCCGCGCGCGTACGAAAAACAATTACGGGTCTAC
CATCGAGGGCCTGCTCGATCTCCCGGACGACGACGCCCCCGAAGAGGCGGGGC
TGGCGGCTCCGCGCCTGTCCTTTCTCCCCGCGGGACACACGCGCAGACTGTCGA
CGGCCCCCCCGACCGATGTCAGCCTGGGGGACGAGCTCCACTTAGACGGCGAG
GACGTGGCGATGGCGCATGCCGACGCGCTAGACGATTTCGATCTGGACATGTTG

GGGGACGGGGATTCCCCGGGTCCGGGATTTACCCCCACGACTCCGCCCCCTAC
GGCGCTCTGGATATGGCCGACTTCGAGTTTGAGCAGATGTTTACCGATGCCCTT
GGAATTGACGAGTACGGTGGG

- Amino acid sequence, MALPVTALLLPLALLLHAARP*YPYDVPDYATLHNNSTTS PLFPNISSSWIHSPSDAGLPPGTVTHFGSYNVSRAAGNFSSPDGTTDDPLGGHTVWQ VVFIAFLTGILALVTIIGNILVIVSFKVNKQLKTVNNYFLLSLACADLIIGVISMNLFTT YIIMNRWALGNLACDLWLAIDYVASNASVMNLLVISFDRYFSITRPLTYRAKRTTK RAGVMIGLAWVISFVLWAPAILFWQYFVGKRTVPPGECFIQFLSEPTITFGTAIAAFY MPVTIMTILYWRIYKETEKRTKELAGLQASGTEAETENFVHPTGSSRSCSSYELQQQ SMKRSNRRKYGRCHFWFTTKSWKPSSEQMDQDHSSSDSWNNNDAAASLENSASS DEEDIGSETRAIYSIVLKLPGHSTILNSTKLPSSDNLQVPEEELGMVDLERKADKLQA QKSVDDGGSFPKSFSKLPIQLESAVDTAKTSDVNSSVGKSTATLPLSFKEATLAKRF ALKTRSQITKRKRMSLVKEKKAAQTLSAILLAFIITWTPYNIMVLVNTFCDSCIPKTF WNLGYWLCYINSTVNPVCYALCNKTFRTTFKMLLLCQCDKKKRRKQQYQQRQSVI FHKRAPEQALIDTGGRTPPSLGPQDESCTTASSSLAKDTSSTGENLYFQLRLDKSKVI NSALELLNEVGIEGLTTRKLAQKLGVEQPTLYWHVKNKRALLDALAIEMLDRHHT HFCPLEGESWQDFLRNNAKSFRCALLSHRDGAKVHLGTRPTEKQYETLENQLAFLC QQGFSLENALYALSAVGHFTLGCVLEDQEHQVAKEERETPTTDSMPPLLRQAIELFD HQGAEPAFLFGLELIICGLEKQLKCESGSAYSRARTKNNYGSTIEGLLDLPDDDAPEE AGLAAPRLSFLPAGHTRRLSTAPPTDVSLGDELHLDGEDVAMAHADALDDFDLDM LGDGDSPGPGFTPHDSAPYGALDMADFEFEQMFTDALGIDEYGG

C-HA-Trop2
with signal peptide and HA tag colored

- DNA sequence, ATGGCCTTACCAGTGACCGCCTTGCTCCTGCCGCTGGCCTTGCTG CTCCACGCCGCCAGGCCGTGTCACACGGCCGCGCAGGACAACTGCACGTGTCCC ACCAACAAGATGACCGTGTGCAGCCCCGACGGCCCCGGCGGCCGCTGCCAGTGC CGCGCGCTGGGCTCGGGCATGGCGGTCGACTGCTCCACGCTGACCTCCAAGTGT CTGCTGCTCAAGGCGCGCATGAGCGCCCCCAAGAACGCCCGCACGCTGGTGCGG CCGAGTGAGCACGCGCTCGTGGACAACGATGGCCTCTACGACCCCGACTGCGAC CCCGAGGGCCGCTTCAAGGCGCGCCAGTGCAACCAGACGTCGGTGTGCTGGTGC GTGAACTCGGTGGGCGTGCGCCGCACGGACAAGGGCGACCTGAGCCTACGCTG CGATGAGCTGGTGCGCACCCACCACATCCTCATTGACCTGCGCCACCGCCCCACC GCCGGCGCCTTCAACCACTCAGACCTGGACGCCGAGCTGAGGCGGCTCTTCCGC GAGCGCTATCGGCTGCACCCCAAGTTCGTGGCGGCCGTGCACTACGAGCAGCCC ACCATCCAGATCGAGCTGCGGCAGAACACGTCTCAGAAGGCCGCCGGTGACGTG GATATCGGCGATGCCGCCTACTACTTCGAGAGGGACATCAAGGGCGAGTCTCTAT TCCAGGGCCGCGGCGGCCTGGACTTGCGCGTGCGCGGAGAACCCCTGCAGGTG GAGCGCACGCTCATCTATTACCTGGACGAGATTCCCCCGAAGTTCTCCATGAAGC GCCTCACCGCCGGCCTCATCGCCGTCATCGTGGTGGTCGTGGTGGCCCTCGTCGC CGGCATGGCCGTCCTGGTGATCACCAACCGGAGAAAGTCGGGGAAGTACAAGA AGGTGGAGATCAAGGAACTGGGGGAGTTGAGAAAGGAACCGAGCTTGGGACAA GGACAAAAGTCGTACCCATACGATGTTCCAGATTACGCT
- Amino acid sequence, MALPVTALLLPLALLLHAARPCHTAAQDNCTCPTNKMTVCS PDGPGGRCQCRALGSGMAVDCSTLTSKCLLLKARMSAPKNARTLVRPSEHALVDND GLYDPDCDPEGRFKARQCNQTSVCWCVNSVGVRRTDKGDLSLRCDELVRTHHILID

LRHRPTAGAFNHSDLDAELRRLFRERYRLHPKFVAAVHYEQPTIQIELRQNTSQKAA
GDVDIGDAAYYFERDIKGESLFQGRGGLDLRVRGEPLQVERTLIYYLDEIPPKFSMK
RLTAGLIAVIVVVVVALVAGMAVLVITNRRKSGKYKKVEIKELGELRKEPSLGQGQK
SYPYDVPDYA

BFP-P2A-C-HA-EGFR
with P2A, signal peptide and HA tag colored

- DNA sequence, ATGGTGAGCAAGGGCGAGGAGCTGTTCACCGGGGTGGTGCCCA
TCCTGGTCGAGCTGGACGGCGACGTAAACGGCCACAAGTTCAGCGTGAGGGGC
GAGGGCGAGGGCGATGCCACCAACGGCAAGCTGACCCTGAAGTTCATCAGTAC
TACCGGCAAGCTGCCCGTGCCCTGGCCCACCCTCGTGACCACCCTGAGCCACGG
CGTGCAGGTGTTCGCCCGCTACCCCGACCACATGAAGCAGCACGACTTCTTCAA
GTCCGCCATGCCCGAAGGCTACGTCCAGGAGCGCACCATCTTCTTCAAGGACGA
CGGCACCTACAAGACCCGCGCCGAGGTGAAGTTCGAGGGCGACACCCTGGTGA
ACCGCATCGAGCTGAAGGGCGTCGACTTCAAGGAGGACGGCAACATCCTGGGG
CACAAGCTGGAGTACAACTTCAACAGCCACAACATCTATATCATGGCCGTCAAG
CAGAAGAACGGCATCAAGGCCAACTTCAAGATCCGCCACAACGTGGAGGACGG
CAGCGTGCAGCTCGCCGACCACTACCAGCAGAACACCCCCATCGGCGACGGCC
CCGTGCTGCTGCCCGACAGCCACTACCTGAGCACCCAGTCCAAGCTTAGCAAAG
ACCCCAACGAGAAGCGCGATCACATGGTCCTGCTGGAGTTCCGCACCGCCGCCG
GGATCACTCTCGGCATGGACGAGCTGTACAAGAGGCGGAAGAGAGGATCCGGA
GCCACCAATTTCAGCCTGCTGAAACAGGCTGGCGACGTGGAAGAGAACCCTGG
ACCTATGCGACCCTCCGGGACGGCCGGGGCAGCGCTCCTGGCGCTGCTGGCTGC
GCTCTGCCCGGCGAGTCGGGCTTGTGCGGCCGCATATCCTTATGATGTTCCAGAT
TATGCTCTGGAGGAAAAGAAAGTTTGCCAAGGCACGAGTAACAAGCTCACGCA
GTTGGGCACTTTTGAAGATCATTTTCTCAGCCTCCAGAGGATGTTCAATAACTGT
GAGGTGGTCCTTGGGAATTTGGAAATTACCTATGTGCAGAGGAATTATGATCTT
TCCTTCTTAAAGACCATCCAGGAGGTGGCTGGTTATGTCCTCATTGCCCTCAACA
CAGTGGAGCGAATTCCTTTGGAAAACCTGCAGATCATCAGAGGAAATATGTACT
ACGAAAATTCCTATGCCTTAGCAGTCTTATCTAACTATGATGCAAATAAAACCG
GACTGAAGGAGCTGCCCATGAGAAATTTACAGGAAATCCTGCATGGCGCCGTGC
GGTTCAGCAACAACCCTGCCCTGTGCAACGTGGAGAGCATCCAGTGGCGGGAC
ATAGTCAGCAGTGACTTTCTCAGCAACATGTCGATGGACTTCCAGAACCACCTG
GGCAGCTGCCAAAAGTGTGATCCAAGCTGTCCCAATGGGAGCTGCTGGGGTGCA
GGAGAGGAGAACTGCCAGAAACTGACCAAAATCATCTGTGCCCAGCAGTGCTC
CGGGCGCTGCCGTGGCAAGTCCCCCAGTGACTGCTGCCACAACCAGTGTGCTGC
AGGCTGCACAGGCCCCCGGGAGAGCGACTGCCTGGTCTGCCGCAAATTCCGAG
ACGAAGCCACGTGCAAGGACACCTGCCCCCCACTCATGCTCTACAACCCCACCA
CGTACCAGATGGATGTGAACCCCGAGGGCAAATACAGCTTTGGTGCCACCTGCG
TGAAGAAGTGTCCCCGTAATTATGTGGTGACAGATCACGGCTCGTGCGTCCGAG
CCTGTGGGGCCGACAGCTATGAGATGGAGGAAGACGGCGTCCGCAAGTGTAAG
AAGTGCGAAGGGCCTTGCCGCAAAGTGTGTAACGGAATAGGTATTGGTGAATTT
AAAGACTCACTCTCCATAAATGCTACGAATATTAAACACTTCAAAAACTGCACC
TCCATCAGTGGCGATCTCCACATCCTGCCGGTGGCATTTAGGGGTGACTCCTTCA
CACATACTCCTCCTCTGGATCCACAGGAACTGGATATTCTGAAAACCGTAAAGG
AAATCACAGGGTTTTTGCTGATTCAGGCTTGGCCTGAAAACAGGACGGACCTCC

ATGCCTTTGAGAACCTAGAAATCATACGCGGCAGGACCAAGCAACATGGTCAGT
TTTCTCTTGCAGTCGTCAGCCTGAACATAACATCCTTGGGATTACGCTCCCTCAA
GGAGATAAGTGATGGAGATGTGATAATTTCAGGAAACAAAAATTTGTGCTATGC
AAATACAATAAACTGGAAAAAACTGTTTGGGACCTCCGGTCAGAAAACCAAAA
TTATAAGCAACAGAGGTGAAAACAGCTGCAAGGCCACAGGCCAGGTCTGCCAT
GCCTTGTGCTCCCCCGAGGGCTGCTGGGGCCCGGAGCCCAGGGACTGCGTCTCT
TGCCGGAATGTCAGCCGAGGCAGGGAATGCGTGGACAAGTGCAACCTTCTGGA
GGGTGAGCCAAGGGAGTTTGTGGAGAACTCTGAGTGCATACAGTGCCACCCAG
AGTGCCTGCCTCAGGCCATGAACATCACCTGCACAGGACGGGGACCAGACAAC
TGTATCCAGTGTGCCCACTACATTGACGGCCCCCACTGCGTCAAGACCTGCCCG
GCAGGAGTCATGGGAGAAAACAACACCCTGGTCTGGAAGTACGCAGACGCCGG
CCATGTGTGCCACCTGTGCCATCCAAACTGCACCTACGGATGCACTGGGCCAGG
TCTTGAAGGCTGTCCAACGAATGGGCCTAAGATCCCGTCCATCGCCACTGGGAT
GGTGGGGGCCCTCCTCTTGCTGCTGGTGGTGGCCCTGGGGATCGGCCTCTTCATG
CGAAGGCGCCACATCGTTCGGAAGCGCACGCTGCGGAGGCTGCTGCAGGAGAG
GGAGCTTGTGGAGCCTCTTACACCCAGTGGAGAAGCTCCCAACCAAGCTCTCTT
GAGGATCTTGAAGGAAACTGAATTCAAAAAGATCAAAGTGCTGGGCTCCGGTG
CGTTCGGCACGGTGTATAAGGGACTCTGGATCCCAGAAGGTGAGAAAGTTAAA
ATTCCCGTCGCTATCAAGGAATTAAGAGAAGCAACATCTCCGAAAGCCAACAA
GGAAATCCTCGATGAAGCCTACGTGATGGCCAGCGTGGACAACCCCCACGTGTG
CCGCCTGCTGGGCATCTGCCTCACCTCCACCGTGCAGCTCATCACGCAGCTCAT
GCCCTTCGGCTGCCTCCTGGACTATGTCCGGGAACACAAAGACAATATTGGCTC
CCAGTACCTGCTCAACTGGTGTGTGCAGATCGCAAAGGGCATGAACTACTTGGA
GGACCGTCGCTTGGTGCACCGCGACCTGGCAGCCAGGAACGTACTGGTGAAAA
CACCGCAGCATGTCAAGATCACAGATTTTGGGCTGGCCAAACTGCTGGGTGCGG
AAGAGAAAGAATACCATGCAGAAGGAGGCAAAGTGCCTATCAAGTGGATGGCA
TTGGAATCAATTTTACACAGAATCTATACCCACCAGAGTGATGTCTGGAGCTAC
GGGGTGACTGTTTGGGAGTTGATGACCTTTGGATCCAAGCCATATGACGGAATC
CCTGCCAGCGAGATCTCCTCCATCCTGGAGAAAGGAGAACGCCTCCCTCAGCCA
CCCATATGTACCATCGATGTCTACATGATCATGGTCAAGTGCTGGATGATAGAC
GCAGATAGTCGCCCAAAGTTCCGTGAGTTGATCATCGAATTCTCCAAAATGGCC
CGAGACCCCCAGCGCTACCTTGTCATTCAGGGGGATGAAAGAATGCATTTGCCA
AGTCCTACAGACTCCAACTTCTACCGTGCCCTGATGGATGAAGAAGACATGGAC
GACGTGGTGGATGCCGACGAGTACCTCATCCCACAGCAGGGCTTCTTCAGCAGC
CCCTCCACGTCACGGACTCCCCTCCTGAGCTCTCTGAGTGCAACCAGCAACAAT
TCCACCGTGGCTTGCATTGATAGAAATGGGCTGCAAAGCTGTCCCATCAAGGAA
GACAGCTTCTTGCAGCGATACAGCTCAGACCCCACAGGCGCCTTGACTGAGGAC
AGCATAGACGACACCTTCCTCCCAGTGCCTGAATACATAAACCAGTCCGTTCCC
AAAAGGCCCGCTGGCTCTGTGCAGAATCCTGTCTATCACAATCAGCCTCTGAAC
CCCGCGCCCAGCAGAGACCCACACTACCAGGACCCCCACAGCACTGCAGTGGG
CAACCCCGAGTATCTCAACACTGTCCAGCCCACCTGTGTCAACAGCACATTCGA
CAGCCCTGCCCACTGGGCCCAGAAAGGCAGCCACCAAATTAGCCTGGACAACC
CTGACTACCAGCAGGACTTCTTTCCCAAGGAAGCCAAGCCAAATGGCATCTTTA
AGGGCTCCACAGCTGAAAATGCAGAATACCTAAGGGTCGCGCCACAAAGCAGT
GAATTTATTGGAGCA

- Amino acid sequence, MVSKGEELFTGVVPILVELDGDVNGHKFSVRGEGEGDATNG KLTLKFISTTGKLPVPWPTLVTTLSHGVQVFARYPDHMKQHDFFKSAMPEGYVQER TIFFKDDGTYKTRAEVKFEGDTLVNRIELKGVDFKEDGNILGHKLEYNFNSHNIYIM AVKQKNGIKANFKIRHNVEDGSVQLADHYQQNTPIGDGPVLLPDSHYLSTQSKLSK DPNEKRDHMVLLEFRTAAGITLGMDELYKRRKRGSGATNFSLLKQAGDVEENPGP MRPSGTAGAALLALLAALCPASRACAAAYPYDVPDYALEEKKVCQGTSNKLTQLG TFEDHFLSLQRMFNNCEVVLGNLEITYVQRNYDLSFLKTIQEVAGYVLIALNTVERI PLENLQIIRGNMYYENSYALAVLSNYDANKTGLKELPMRNLQEILHGAVRFSNNPA LCNVESIQWRDIVSSDFLSNMSMDFQNHLGSCQKCDPSCPNGSCWGAGEENCQKL TKIICAQQCSGRCRGKSPSDCCHNQCAAGCTGPRESDCLVCRKFRDEATCKDTCPPL MLYNPTTYQMDVNPEGKYSFGATCVKKCPRNYVVTDHGSCVRACGADSYEMEED GVRKCKKCEGPCRKVCNGIGIGEFKDSLSINATNIKHFKNCTSISGDLHILPVAFRGD SFTHTPPLDPQELDILKTVKEITGFLLIQAWPENRTDLHAFENLEIIRGRTKQHGQFSL AVVSLNITSLGLRSLKEISDGDVIISGNKNLCYANTINWKKLFGTSGQKTKIISNRGE NSCKATGQVCHALCSPEGCWGPEPRDCVSCRNVSRGRECVDKCNLLEGEPREFVE NSECIQCHPECLPQAMNITCTGRGPDNCIQCAHYIDGPHCVKTCPAGVMGENNTLV WKYADAGHVCHLCHPNCTYGCTGPGLEGCPTNGPKIPSIATGMVGALLLLLVVAL GIGLFMRRRHIVRKRTLRRLLQERELVEPLTPSGEAPNQALLRILKETEFKKIKVLGS GAFGTVYKGLWIPEGEKVKIPVAIKELREATSPKANKEILDEAYVMASVDNPHVCR LLGICLTSTVQLITQLMPFGCLLDYVREHKDNIGSQYLLNWCVQIAKGMNYLEDRR LVHRDLAARNVLVKTPQHVKITDFGLAKLLGAEEKEYHAEGGKVPIKWMALESIL HRIYTHQSDVWSYGVTVWELMTFGSKPYDGIPASEISSILEKGERLPQPPICTIDVYM IMVKCWMIDADSRPKFRELIIEFSKMARDPQRYLVIQGDERMHLPSPTDSNFYRAL MDEEDMDDVVDADEYLIPQQGFFSSPSTSRTPLLSSLSATSNNSTVACIDRNGLQSC PIKEDSFLQRYSSDPTGALTEDSIDDTFLPVPEYINQSVPKRPAGSVQNPVYHNQPLN PAPSRDPHYQDPHSTAVGNPEYLNTVQPTCVNSTFDSPAHWAQKGSHQISLDNPDY QQDFFPKEAKPNGIFKGSTAENAEYLRVAPQSSEFIGA

***Mm*PylRS**

- This orthogonal synthetase can use **CysK** for aminoacylation.
- DNA sequence, ATGGACAAGAAGCCCCTGAACACCCTGATCAGCGCCACAGGA CTGTGGATGTCCAGAACCGGCACCATCCACAAGATCAAGCACCACGAGGTGTCC CGGTCCAAAATCTACATCGAGATGGCCTGCGGCGATCACCTGGTCGTCAACAAC AGCAGAAGCAGCCGGACAGCCAGAGCCCTGCGGCACCACAAGTACAGAAAGAC CTGCAAGCGGTGCAGAGTGTCCGACGAGGACCTGAACAAGTTCCTGACCAAGG CCAACGAGGACCAGACCAGCGTGAAAGTGAAGGTGGTGTCCGCCCCCACCCGG ACCAAGAAAGCCATGCCCAAGAGCGTGGCCAGAGCCCCCAAGCCCCTGGAAAA CACCGAAGCCGCTCAGGCCCAGCCCAGCGGCAGCAAGTTCAGCCCCGCCATCCC CGTGTCTACCCAGGAAAGCGTCAGCGTCCCCGCCAGCGTGTCCACCAGCATCTC TAGCATCTCAACCGGCGCCACAGCTTCTGCCCTGGTCAAGGGCAACACCAACCC CATCACCAGCATGTCTGCCCCTGTGCAGGCCTCTGCCCCAGCCCTGACCAAGTC CCAGACCGACCGGCTGGAAGTGCTCCTGAACCCCAAGGACGAGATCAGCCTGA ACAGCGGCAAGCCCTTCCGGGAGCTGGAAAGCGAGCTGCTGAGCCGGCGGAAG AAGGACCTCCAGCAAATCTACGCCGAGGAACGGGAGAACTACCTGGGCAAGCT GGAAAGAGAGATCACCCGGTTCTTCGTGGACCGGGGCTTCCTGGAAATCAAGA GCCCCATCCTGATCCCCCTGGAGTACATCGAGCGGATGGGCATCGACAACGACA

CCGAGCTGAGCAAGCAGATTTTCCGGGTGGACAAGAACTTCTGCCTGCGGCCCA
TGCTGGCCCCCAACCTGTACAACTACCTGCGGAAACTGGATCGCGCTCTGCCCG
ACCCCATCAAGATTTTCGAGATCGGCCCCTGCTACCGGAAAGAGAGCGACGGC
AAAGAGCACCTGGAAGAGTTTACAATGCTGAACTTTTGCCAGATGGGCAGCGGC
TGCACCAGAGAGAACCTGGAATCCATCATCACCGACTTTCTGAACCACCTGGGG
ATCGACTTCAAGATCGTGGGCGACAGCTGCATGGTGTACGGCGACACCCTGGAC
GTGATGCACGGCGACCTGGAACTGTCTAGCGCCGTCGTGGGACCCATCCCTCTG
GACCGGGAGTGGGGCATCGATAAGCCCTGGATCGGAGCCGGCTTCGGCCTGGA
ACGGCTGCTGAAAGTCAAGCACGACTTTAAGAACATCAAGCGGGCTGCCAGAA
GCGAGAGCTACTACAACGGCATCAGCACCAACCTG

- Amino acid sequence, MDKKPLNTLISATGLWMSRTGTIHKIKHHEVSRSKIYIEMAC GDHLVVNNSRSSRTARALRHHKYRKTCKRCRVSDEDLNKFLTKANEDQTSVKVKV VSAPTRTKKAMPKSVARAPKPLENTEAAQAQPSGSKFSPAIPVSTQESVSVPASVST SISSISTGATASALVKGNTNPITSMSAPVQASAPALTKSQTDRLEVLLNPKDEISLNSG KPFRELESELLSRRKKDLQQIYAEERENYLGKLEREITRFFVDRGFLEIKSPILIPLEYI ERMGIDNDTELSKQIFRVDKNFCLRPMLAPNLYNYLRKLDRALPDPIKIFEIGPCYRK ESDGKEHLEEFTMLNFCQMGSGCTRENLESIITDFLNHLGIDFKIVGDSCMVYGDTL DVMHGDLELSSAVVGPIPLDREWGIDKPWIGAGFGLERLLKVKHDFKNIKRAARSE SYYNGISTNL

Mm*PylRS

- This orthogonal synthetase can use **BCNK** for aminoacylation.
- DNA sequence, ATGGACAAGAAGCCCCTGAACACCCTGATCAGCGCCACAGGACT GTGGATGTCCAGAACCGGCACCATCCACAAGATCAAGCACCACGAGGTGTCCC GGTCCAAAATCTACATCGAGATGGCCTGCGGCGATCACCTGGTCGTCAACAACA GCAGAAGCAGCCGGACAGCCAGAGCCCTGCGGCACCACAAGTACAGAAAGACC TGCAAGCGGTGCAGAGTGTCCGACGAGGACCTGAACAAGTTCCTGACCAAGGC CAACGAGGACCAGACCAGCGTGAAAGTGAAGGTGGTGTCCGCCCCCACCCGGA CCAAGAAAGCCATGCCCAAGAGCGTGGCCAGAGCCCCCAAGCCCCTGGAAAAC ACCGAAGCCGCTCAGGCCCAGCCCAGCGGCAGCAAGTTCAGCCCCGCCATCCCC GTGTCTACCCAGGAAAGCGTCAGCGTCCCCGCCAGCGTGTCCACCAGCATCTCT AGCATCTCAACCGGCGCCACAGCTTCTGCCCTGGTCAAGGGCAACACCAACCCC ATCACCAGCATGTCTGCCCCTGTGCAGGCCTCTGCCCCAGCCCTGACCAAGTCC CAGACCGACCGGCTGGAAGTGCTCCTGAACCCCAAGGACGAGATCAGCCTGAA CAGCGGCAAGCCCTTCCGGGAGCTGGAAAGCGAGCTGCTGAGCCGGCGGAAGA AGGACCTCCAGCAAATCTACGCCGAGGAACGGGAGAACTACCTGGGCAAGCTG GAAAGAGAGATCACCCGGTTCTTCGTGGACCGGGGCTTCCTGGAAATCAAGAGC CCCATCCTGATCCCCCTGGAGTACATCGAGCGGATGGGCATCGACAACGACACC GAGCTGAGCAAGCAGATTTTCCGGGTGGACAAGAACTTCTGCCTGCGGCCCATG CTGGCCCCCAACCTGGCCAACTACCTGCGGAAACTGGATCGCGCTCTGCCCGAC CCCATCAAGATTTTCGAGATCGGCCCCTGCTACCGGAAAGAGAGCGACGGCAA AGAGCACCTGGAAGAGTTTACAATGCTGAACTTTTGCCAGATGGGCAGCGGCTG CACCAGAGAGAACCTGGAATCCATCATCACCGACTTTCTGAACCACCTGGGGAT CGACTTCAAGATCGTGGGCGACAGCTGCATGGTGTTCGGCGACACCCTGGACGT GATGCACGGCGACCTGGAACTGTCTAGCGCCGTCGTGGGACCCATCCCTCTGGA CCGGGAGTGGGGCATCGATAAGCCCTGGATCGGAGCCGGCTTCGGCCTGGAAC

GGCTGCTGAAAGTCAAGCACGACTTTAAGAACATCAAGCGGGCTGCCAGAAGC GAGAGCTACTACAACGGCATCAGCACCAACCTG

- Amino acid sequence, MDKKPLNTLISATGLWMSRTGTIHKIKHHEVSRSKIYIEMA CGDHLVVNNSRSSRTARALRHHKYRKTCKRCRVSDEDLNKFLTKANEDQTSVKVK VVSAPTRTKKAMPKSVARAPKPLENTEAAQAQPSGSKFSPAIPVSTQESVSVPASVS TSISSISTGATASALVKGNTNPITSMSAPVQASAPALTKSQTDRLEVLLNPKDEISLNS GKPFRELESELLSRRKKDLQQIYAEERENYLGKLEREITRFFVDRGFLEIKSPILIPLEY IERMGIDNDTELSKQIFRVDKNFCLRPMLAPNLANYLRKLDRALPDPIKIFEIGPCYR KESDGKEHLEEFTMLNFCQMGSGCTRENLESIITDFLNHLGIDFKIVGDSCMVFGDT LDVMHGDLELSSAVVGPIPLDREWGIDKPWIGAGFGLERLLKVKHDFKNIKRAARS ESYYNGISTNL

Pyl-tRNA$_{\mathbf{CUA}}$

- This orthogonal tRNA is the partner of *Mm*PylRS and its engineered variants. It decodes the amber codon (UAG) on the mRNA.
- DNA sequence, GGAAACCTGATCATGTAGATCGAACGGACTCTAAATCCGTTCAG CCGGGTTAGATTCCCGGGGTTTCCG

Pyl-tRNA$_{\mathbf{UCCU}}$

- This orthogonal tRNA is the partner of *Mm*PylRS and its engineered variants. It decodes quadruplet codon AGGA on the mRNA.
- DNA sequence, GGAAACCTGATCATGTAGATCGAAGGGGCTTCCTATCCGCTTCA GCCGGGTTAGATTCCCGGGGTTTCCG

Ec*TyrRS

- This orthogonal synthetase can use **PPA** for aminoacylation.
- DNA sequence, ATGGCAAGCAGTAACTTGATTAAACAATTGCAAGAGCGGGGGCT GGTAGCCCAGGTGACGGACGAGGAAGCGTTAGCAGAGCGACTGGCGCAAGGCC CGATCGCACTCAGCTGTGGCTTCGATCCTACCGCTGACAGCTTGCATTTGGGGC ATCTTGTTCCATTGTTATGCCTGAAACGCTTCCAGCAGGCGGGCCACAAGCCGG TTGCGCTGGTAGGCGGCGCGACGGGTCTGATTGGCGACCCGAGCTTCAAAGCTG CCGAGCGTAAGCTGAACACCGAAGAAACTGTTCAGGAGTGGGTGGACAAAATC CGTAAGCAGGTTGCCCCGTTCCTCGATTTCGACTGTGGAGAAAACTCTGCTATC GCGGCCAATAATTATGACTGGTTCGGCAATATGAATGTGCTGACCTTCCTGCGC GATATTGGCAAACACTTCTCCGTTAACCAGATGATCAACAAAGAAGCGGTTAAG CAGCGTCTCAACCGTGAAGATCAGGGGATTTCGTTCACTGAGTTTTCCTACAAC CTGCTGCAGGGTTATACTATGGCCTGTGTGAACAAACAGTACGGTGTGGTGCTG CAAATTGGTGGTTCTGACCAGTGGGGTAACATCACTTCTGGTATCGACCTGACC CGTCGTCTGCATCAGAATCAGGTGTTTGGCCTGACCGTTCCGCTGATCACTAAA GCAGATGGCACCAAATTTGGTAAAACTGAAGGCGGCGCAGTCTGGTTGGATCCG AAGAAAACCAGCCCGTACAAATTCTACCAGTTCTGGATCAACACTGCGCGTGCC GACGTTTACCGCTTCCTGAAGTTCTTCACCTTTATGAGCATTGAAGAGATCAACG CCCTGGAAGAAGAAGATAAAAACAGCGGTAAAGCACCGCGCGCCCAGTATGTA CTGGCGGAGCAGGTGACTCGTCTGGTTCACGGTGAAGAAGGTTTACAGGCGGCA AAACGTATTACCGAATGCCTGTTCAGCGGTTCTTTGAGTGCGCTGAGTGAAGCG GACTTCGAACAGCTGGCGCAGGACGGCGTACCGATGGTTGAGATGGAAAAGGG

CGCAGACCTGATGCAGGCACTGGTCGATTCTGAACTGCAACCTTCCCGTGGTCA GGCACGTAAAACTATCGCCTCCAATGCCATCACCATTAACGGTGAAAAACAGTC CGATCCTGAATACTTCTTTAAAGAAGAAGATCGTCTGTTTGGTCGTTTTACCTTA CTGCGTCGCGGTAAAAAGAATTACTGTCTGATTTGCTGGAAAGGGCCCGTT

- Amino acid sequence, MASSNLIKQLQERGLVAQVTDEEALAERLAQGPIALSCGFDP TADSLHLGHLVPLLCLKRFQQAGHKPVALVGGATGLIGDPSFKAAERKLNTEETVQ EWVDKIRKQVAPFLDFDCGENSAIAANNYDWFGNMNVLTFLRDIGKHFSVNQMIN KEAVKQRLNREDQGISFTEFSYNLLQGYTMACVNKQYGVVLQIGGSDQWGNITSGI DLTRRLHQNQVFGLTVPLITKADGTKFGKTEGGAVWLDPKKTSPYKFYQFWINTAR ADVYRFLKFFTFMSIEEINALEEEDKNSGKAPRAQYVLAEQVTRLVHGEEGLQAAK RITECLFSGSLSALSEADFEQLAQDGVPMVEMEKGADLMQALVDSELQPSRGQARK TIASNAITINGEKQSDPEYFFKEEDRLFGRFTLLRRGKKNYCLICWKGPV

Tyr-tRNA$_{\mathbf{UCA}}$

- This orthogonal tRNA is the partner of *Ec*TyrRS* and decode the opal codon (UGA) on the mRNA.
- DNA sequence, GGAGGGGTAGCGAAGTGGCTAAACGCGGCGGACTTTCAAATCC GCTCCCTTT GGGTTCGGCGGTTCGAATCCGTCCCCCTCCA

Ec*LeuRS

- This orthogonal synthetase can use **HexC** for aminoacylation.
- DNA sequence, ACTGAAGAGCAATACCGCCCGGAAGAGATAGAATCCAAAGTAC AGCTTCATTGGGATGAGAAGCGCACATTTGAAGTAACCGAAGACGAGAGCAAA GAGAAGTATTACTGCCTCAGCATCCTGCCCTATCCTTCTGGTCGACTACACATGG GCCACGTACGTAACTACACCATCGGTGACGTGATCGCCCGCTACCAGCGTATGC TGGGCAAAAACGTCCTGCAGCCGATCGGCTGGGACGCGTTTGGTCTGCCTGCGG AAGGCGCGGCGGTGAAAAACAACACCGCTCCGGCACCGTGGACGTACGACAAC ATCGCGTATATGAAAAACCAGCTCAAAATGCTGGGCTTTGGTTATGACTGGAGC CGCGAGCTGGCAACCTGTACGCCGGAATACTACCGTTGGGAACAGAAATTCTTC ACCGAGCTGTATAAAAAAGGCCTGGTATATAAGAAGACTTCTGCGGTCAACTGG TGTCCGAACGACCAGACCGTACTGGCGAACGAACAAGTTATCGACGGCTGCTGC TGGCGCTGCGATACCAAAGTTGAACGTAAAGAGATCCCGCAGTGGTTTATCAAA ATCACTGCTTACGCTGACGAGCTGCTCAACGATCTGGATAAACTGGATCACTGG CCTGACACCGTTAAAACCATGCAGCGTAACTGGATCGGTCGTTCCGAAGGCGTG GAGATCACCTTCAACGTTAACGACTATGACAACACGCTGACCGTTTACACTACC CGCCCGGACGCCTTTATGGGTTGTACCTACCTGGCGGTAGCTGCGGGTCATCCG CTGGCGCAGAAAGCGGCGGAAAATAATCCTGAACTGGCGGCCTTTATTGACGA ATGCCGTAACACCAAAGTTGCCGAAGCTGAAATGGCGACGATGGAGAAAAAAG GCGTCGATACTGGCTTTAAAGCGGTTCACCCATTAACGGGCGAAGAAATTCCCG TTTGGGCAGCAAACTTCGTATTGATGGAGTACGGCACGGGCGCAGTTATGGCGG TACCGGGGCACGACCAGCGCGACTACGAGTTTGCCTCTAAATACGGCCTGAACA TCAAACCGGTTATCCTGGCAGCTGACGGCTCTGAGCCAGATCTTTCTCAGCAAG CCCTGACTGAAAAAGGCGTGCTGTTCAACTCTGGCGAGTTCAACGGTCTTGACC ATGAAGCGGCCTTCAACGCCATCGCCGATAAACTGACTGCGATGGGCGTTGGCG AGCGTAAAGTGAACTACCGCCTGCGCGACTGGGGTGTTTCCCGTCAGCGTTACT GGGGCGCGCCGATTCCGATGGTGACTCTAGAAGACGGTACCGTAATGCCGACCC

CGGACGACCAGCTGCCGGTGATCCTGCCGGAGGATGTGGTAATGGACGGCATT
ACCAGCCCGATTAAAGCAGATCCGGAGTGGGCGAAAACTACCGTTAACGGTAT
GCCAGCACTGCGTGAAACCGACACTTTCGACACCTTTATGGAGTCCTCCTGGAT
CTACGCGCGCTACACTTGCCCGCAGTACAAAGAAGGTATGCTGGATTCCGAAGC
GGCTAACTACTGGCTGCCGGTGGATATCGCGATTGGTGGTATTGAACACGCCAT
TATGGGACTGCTCTACTTTCGCTTCTTCCACAAACTGATGCGTGATGCAGGCATG
GTGAACTCTGACGAACCAGCCAAACAGTTGCTGTGTCAGGGTATGGTGCTGGCA
GATGCCTTCTACTATGTTGGCGAAAACGGCGAACGTAACTGGGTTTCCCCGGTT
GATGCTATCGTTGAACGTGACGAGAAAGGCCGTATCGTGAAAGCGAAAGATGC
GGCAGGCCATGAACTGGTTTATACCGGCATGAGCAAAATGTCCAAGTCGAAGA
ACAACGGTATCGACCCGCAGGTGATGGTTGAACGTTACGGCGCGGACACCGTTC
GTCTGTTTATGATGTTTGCTTCTCCGGCTGATATGACTCTCGAATGGCAGGAATC
CGGTGTGGAAGGGGCTAACCGCTTCCTGAAACGTGTCTGGAAACTGGTTTACGA
GCACACAGCAAAAGGAGATGTTGCGGCACTGAACGTTGATGCGCTGACTGAAA
ATCAGAAAGCGCTGCGTCGCGATGTGCATAAAACGATCGCTAAAGTGACCGAT
GATATCGGCCGTCGTCAGACCTTCAACACCGCAATTGCGGCGATTATGGAGCTG
ATGAACAAACTGGCGAAAGCACCAACCGATGGCGAGCAGGATCGCGCTCTGAT
GCAGGAAGCACTGCTGGCCGTTGTCCGTATGCTTAACCCGTTCACCCCGCACAT
CTGCTTCACGCTGTGGCAGGAACTGAAAGGCGAAGGCGATATCGACAACGCGC
CGTGGCCGGTTGCTGACGAAAAAGCGATGGTGGAAGACTCCACGCTGGTCGTG
GTGCAGGTTAACGGTAAAGTCCGTGCCAAAATCACCGTTCCGGTGGACGCAACG
GAAGAACAGGTTCGCGAACGTGCTGGCCAGGAACATCTGGTAGCAAAATATCT
TGATGGCGTTACTGTACGTAAAGTGATTTACGTACCAGGTAAACTCCTCAATCT
GGTCGTTGGCGGGCCCGTT

- Amino acid sequence, MEEQYRPEEIESKVQLHWDEKRTFEVTEDESKEKYYCLSILPY
PSGRLHMGHVRNYTIGDVIARYQRMLGKNVLQPIGWDAFGLPAEGAAVKNNTAPA
PWTYDNIAYMKNQLKMLGFGYDWSRELATCTPEYYRWEQKFFTELYKKGLVYKK
TSAVNWCPNDQTVLANEQVIDGCCWRCDTKVERKEIPQWFIKITAYADELLNDLD
KLDHWPDTVKTMQRNWIGRSEGVEITFNVNDYDNTLTVYTTRPDAFMGCTYLAV
AAGHPLAQKAAENNPELAAFIDECRNTKVAEAEMATMEKKGVDTGFKAVHPLTG
EEIPVWAANFVLMEYGTGAVMAVPGHDQRDYEFASKYGLNIKPVILAADGSEPDL
SQQALTEKGVLFNSGEFNGLDHEAAFNAIADKLTAMGVGERKVNYRLRDWGVSR
QRYWGAPIPMVTLEDGTVMPTPDDQLPVILPEDVVMDGITSPIKADPEWAKTTVNG
MPALRETDTFDTFMESSWIYARYTCPQYKEGMLDSEAANYWLPVDIAIGGIEHAIM
GLLYFRFFHKLMRDAGMVNSDEPAKQLLCQGMVLADAFYYVGENGERNWVSPV
DAIVERDEKGRIVKAKDAAGHELVYTGMSKMSKSKNNGIDPQVMVERYGADTVR
LFMMFASPADMTLEWQESGVEGANRFLKRVWKLVYEHTAKGDVAALNVDALTE
NQKALRRDVHKTIAKVTDDIGRRQTFNTAIAAIMELMNKLAKAPTDGEQDRALMQ
EALLAVVRMLNPFTPHICFTLWQELKGEGDIDNAPWPVADEKAMVEDSTLVVVQV
NGKVRAKITVPVDATEEQVRERAGQEHLVAKYLDGVTVRKVIYVPGKLLNLVVGG
PV

Leu-tRNA$_{CUA}$

- This orthogonal tRNA is the partner of *Ec*LeuRS*. It decodes the amber codon (UAG) on the mRNA.
- DNA sequence, GCCCGGATGGTGGAATCGGTAGACACAAGGGATTCTAAATCCCT CGGCGTTCGCGCTGTGCGGGTTCAAGTCCCGCTCCGGGTA

Supplementary Figures

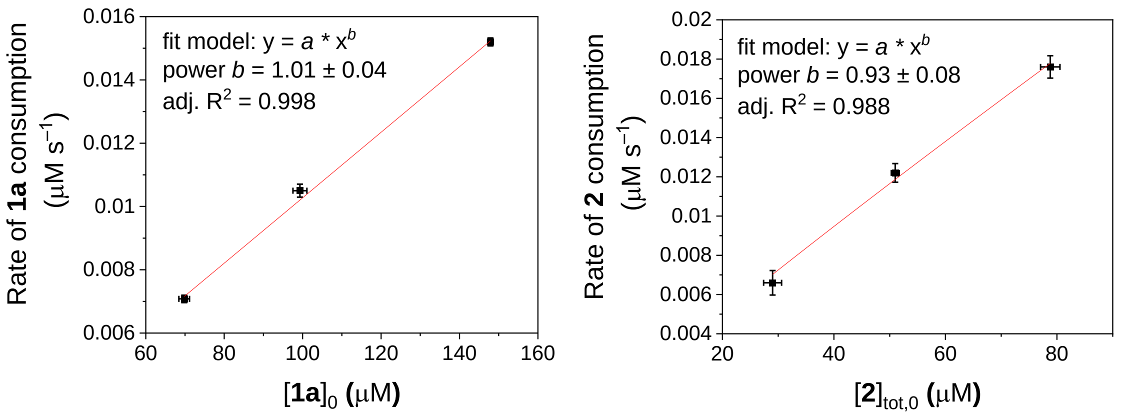

Fig. S1. Determination of the reaction order.
Fit of the rate dependence on the initial concentration to the power law equation $y = ax^b$. Exponents of 1.01 and 0.93 were found for [**1a**] and [**2x**], respectively.

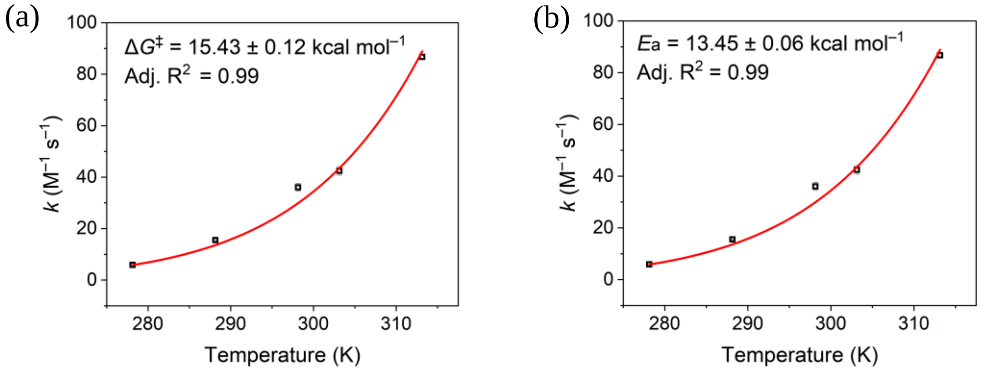

Fig. S2. Eyring (a) and Arrhenius (b) plots for the reaction of **1a** and **2x** at pH 7.4.

(a)

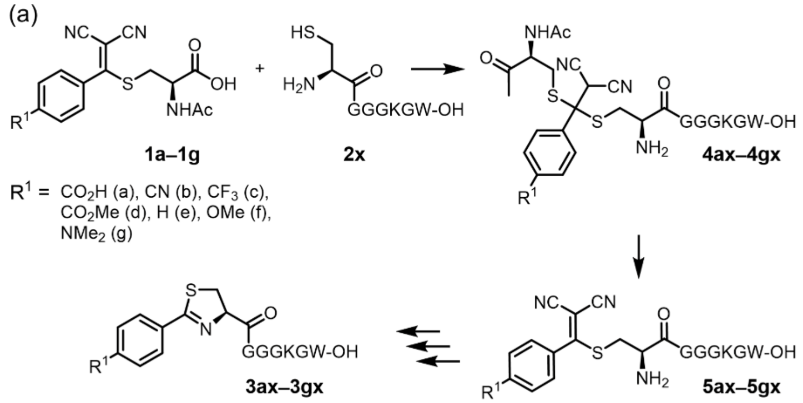

(b)

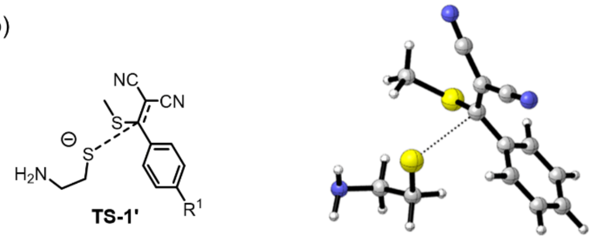

R^1	$k_{obs}^{7.4}$ (M^{-1} s^{-1})	k (M^{-1} s^{-1})	$\Delta G^{‡}_{calc}$ (kcal mol^{-1})
CN	25.0 ± 0.4	170.8 ± 2.6	12.30
CO_2Me	19.2 ± 0.3	130.8 ± 2.0	12.61
CF_3	11.4 ± 0.4	77.8 ± 2.8	12.73
CO_2^-	5.30 ± 0.22	33.5 ± 1.5	14.09
H	3.14 ± 0.09	19.8 ± 0.6	14.23
OMe	3.12 ± 0.03	21.3 ± 0.2	15.87
NMe_2	0.92 ± 0.04	6.3 ± 0.3	17.48

Fig. S3. Effect of TAMM *para* substitution (R^1) on reaction kinetics.

(a) The stepwise addition-elimination reaction between **1** and **2x** to form **5** as the initial product of the thiolate exchange step. (b) Structure of the transition state (**TS-1’**) for the reaction between truncated **1’** (MeS^- as the thiolate leaving group) and **2’** (2-aminoethane-1-thiolate) and the comparison of the experimental **1** + **2x** rate constants (*k*) and the computed **1’** + **2’** activation free energies ($\Delta G^{‡}_{calc}$).

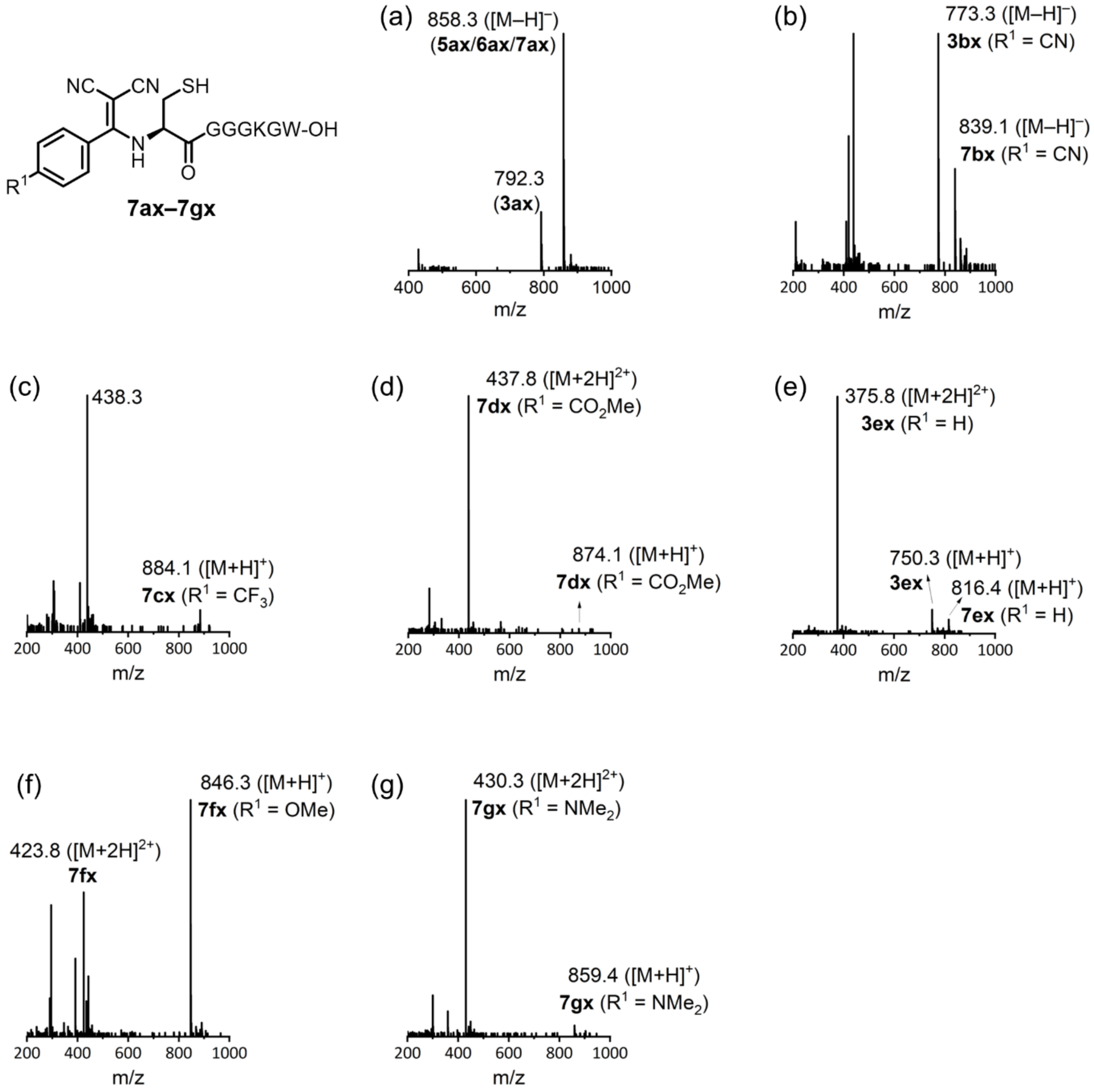

Fig. S4. LC-MS results of reactions involving intermediates.
(a) **7ax** (MW = 859.9 Da), (b) **7bx** (MW = 840.9 Da), (c) **7cx** (MW = 883.9 Da), (d) **7dx** (MW = 873.9 Da), (e) **7ex** (MW = 815.9 Da), (f) **7fx** (MW = 845.9 Da), and (g) **7gx** (MW = 859.9 Da). Freshly prepared stock solutions were mixed at 25 °C to afford the reaction mixture containing TAMM **1** (100 μM), peptide **2x** (50 μM), TCEP (400 μM) and PBS (0.1 M, pH 7.4). Temperature was maintained using Pel-tier-controlled thermostat (Agilent G1330B). At 42 minutes, a sample was analyzed by LC-MS (Agilent 1260 system, column = 5 μm C18 110 Å 250 x 4.6 mm, mobile phase = water and MeCN containing 0.1% v/v formic acid, flow rate = 0.3 mL/min). The mass spectra of the corresponding **Int-1** (**7**) are shown.

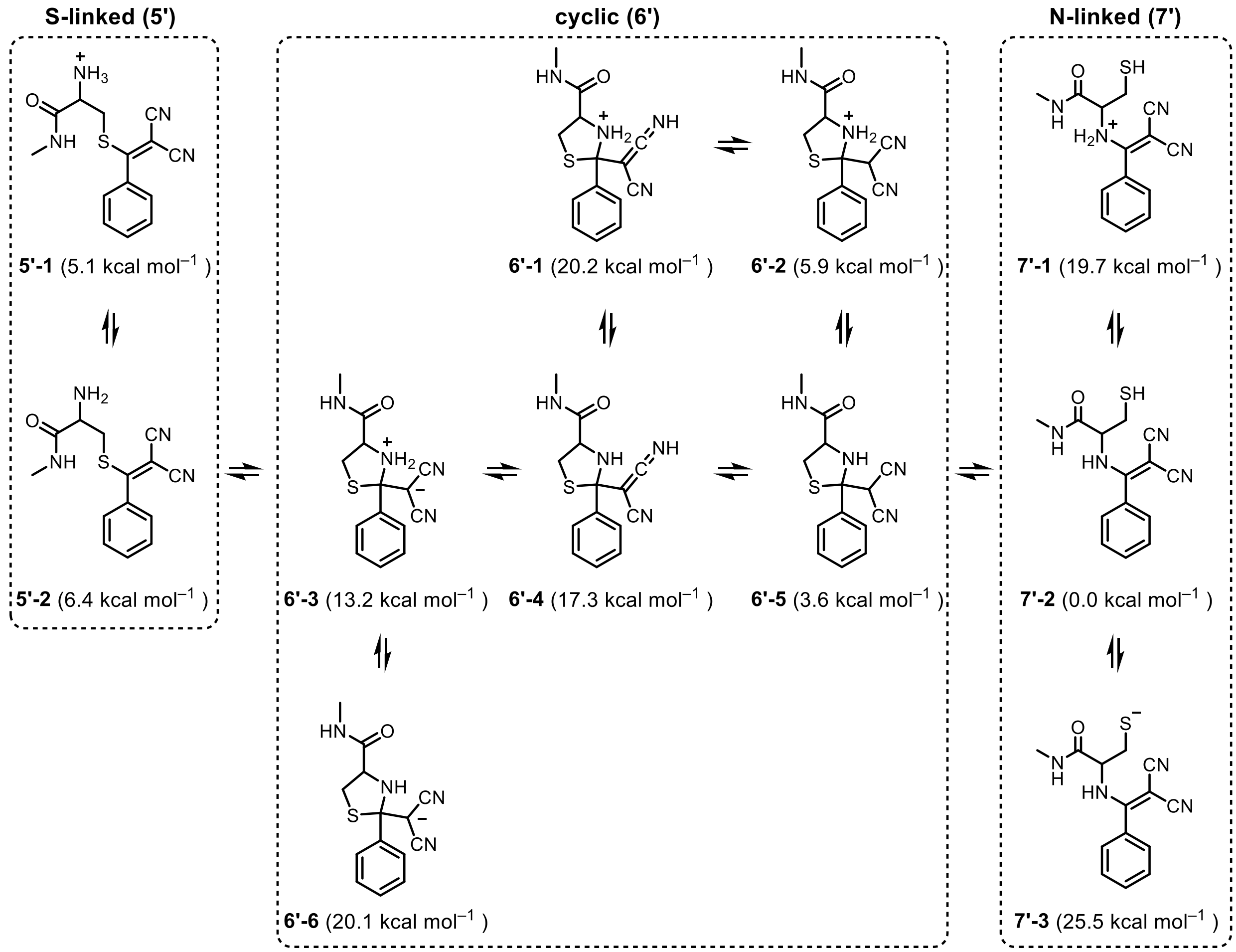

Fig. S5. Calculated free energy of **5'–7'**.

Free energies of structurally optimized species were calculated at SMD(H_2O)-ωB97X-D/6-31+G(d,p)//ωB97X-D/6-31+G(d,p) level of theory. Values were reported in kcal mol^{-1} relative to the *N*-lined neutral form (7'-2).

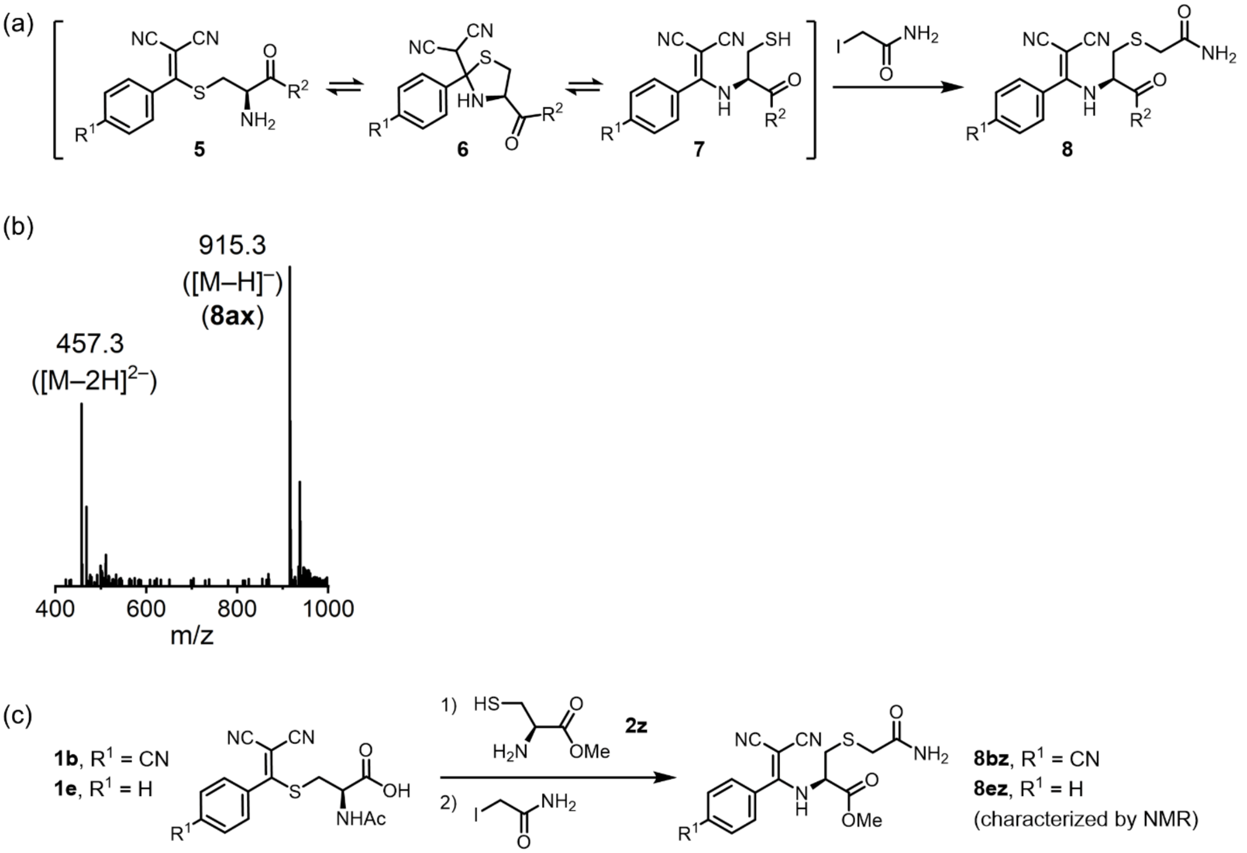

<u>Fig. S6. Characterization of the intermediate in TAMM condensation.</u>
(a) Possible structures of the intermediate as either **5**, **6** or **7**. Iodoacetamide, a thiol-trapping reagent, can react with **7** to form **8**. (b) Mass spectrum of **8ax** consistent with the adduct of **7ax** reacted with iodoacetamide. (c) Trapping of the intermediate of the reaction between **1b** or **1e** and cysteine methyl ester **2z** by iodoacetamide to form **8bz** or **8ez** for NMR characterization (see **Figures S7–S9**).

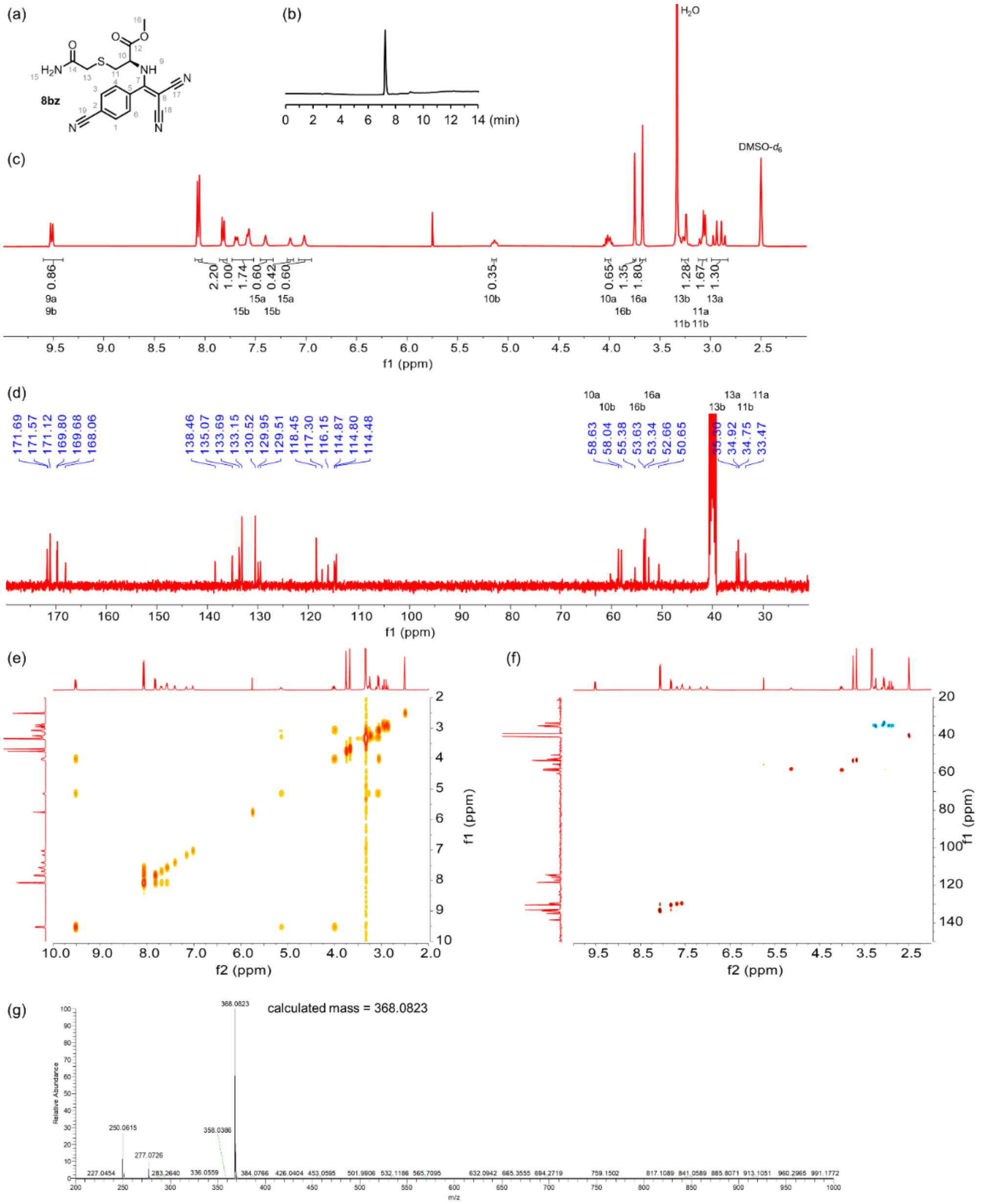

Fig. S7. Structural characterization of **8bz**.

(a) Structure and numbering of **8bz**. (b) HPLC chromatogram of UV absorbance at 254 nm using a C18 column (Agilent, #770450-902) with water (containing 0.1% v/v formic acid) and methanol as the mobile phases at the flow rate of 2 mL/min. The chromatogram began with 10% to 70% methanol from 0 to 6 minutes, 70% to 90% methanol from 6 to 9 minutes, 90% methanol for 3 minutes, followed by 90% to 10% methanol from 12 to 14 minutes. The detector was set at 254 nm. (c-g) ^{1}H, ^{13}C, COSY and HSQC NMR spectra in DMSO-d_6 at 25 °C. The molecule exists as two isomers (annotated as a and b) in a ratio of 56:44 (based on proton 10). It is noteworthy that each ArH is shown as an individual peak in ^{1}H NMR (i.e., 1 and 3 have different chemical shifts). (g) HR-MS.

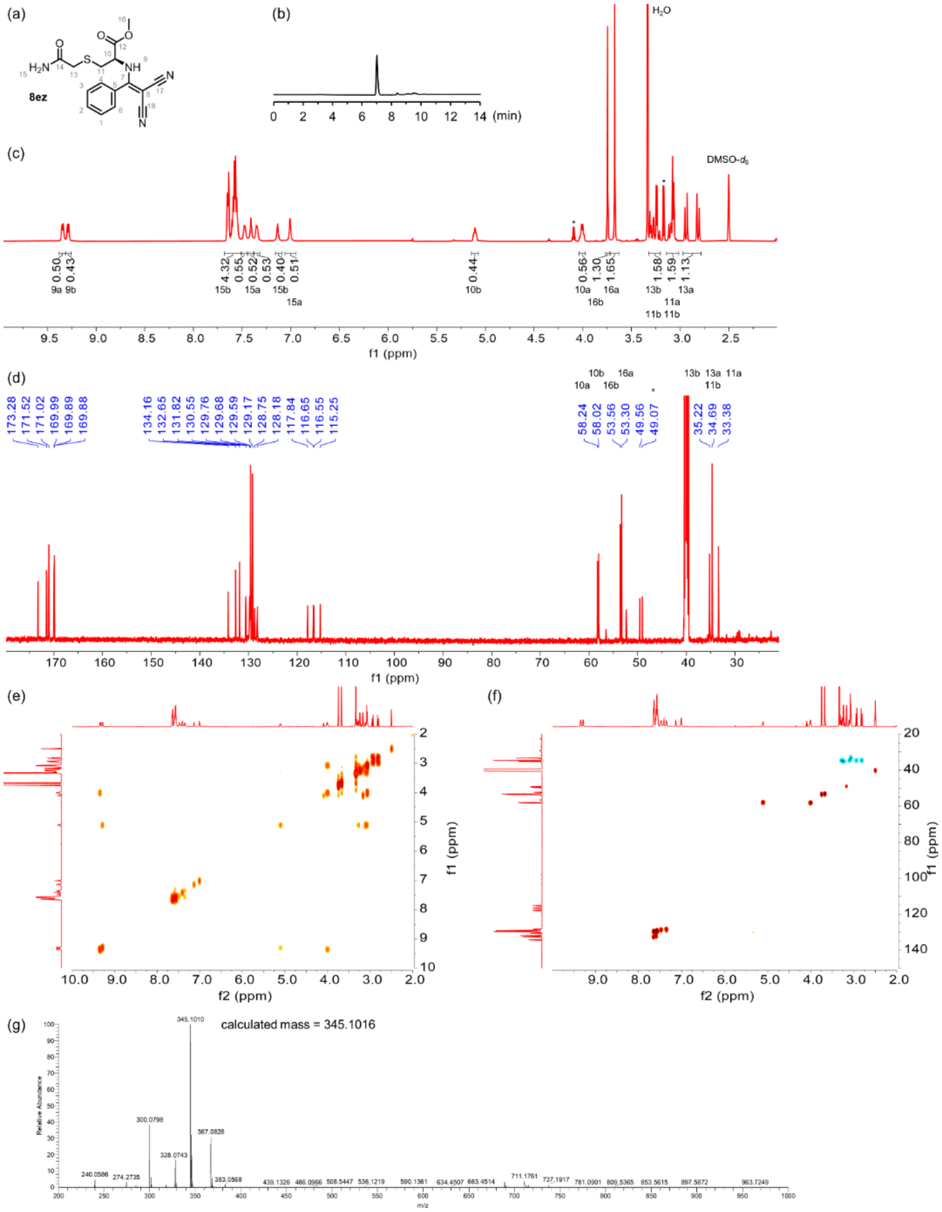

<u>Fig. S8. Structural characterization of **8ez**.</u>

(a) Structure and numbering of **8ez**. (b) HPLC chromatogram of UV absorbance at 254 nm using a C18 column (Agilent, #770450-902) with water (containing 0.1% v/v formic acid) and methanol as the mobile phases at the flow rate of 2 mL/min. The chromatogram began with 10% to 70% methanol from 0 to 6 minutes, 70% to 90% methanol from 6 to 9 minutes, 90% methanol for 3 minutes, followed by 90% to 10% methanol from 12 to 14 minutes. The detector was set at 254 nm. (c-g) ^{1}H, ^{13}C, COSY and HSQC NMR spectra in DMSO-d_6 at 25 °C. The molecule exists as two isomers (annotated as a and b) in a ratio of 56:44 (based on proton 10). It is noteworthy that each ArH is shown as an individual peak in ^{1}H NMR (i.e., 1 and 3 have different chemical shifts). (g) HR-MS.

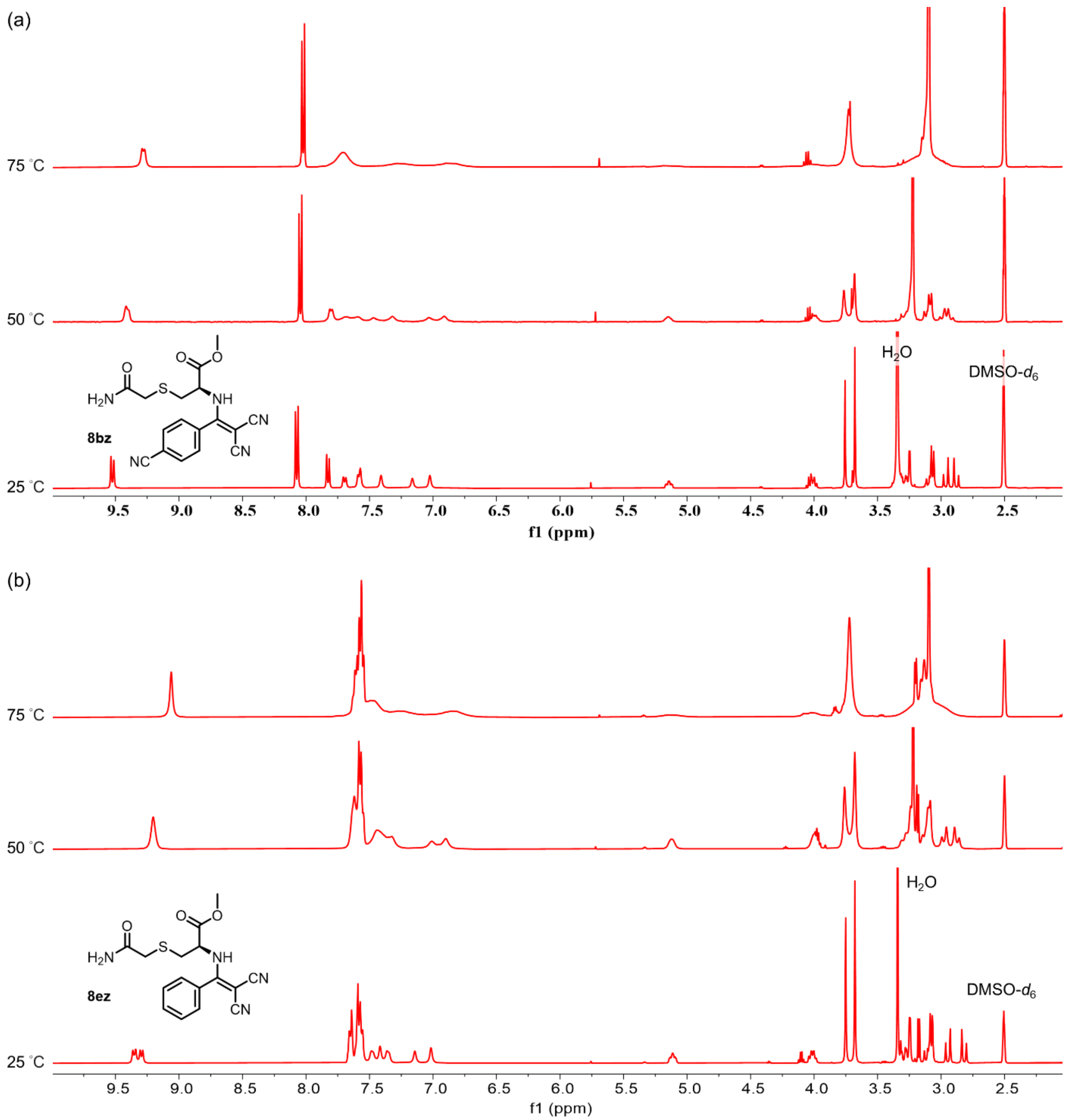

Fig. S9. ^{1}H NMR spectra of **8bz** (a) and **8ez** (b) in DMSO-d_6 at 25, 50 or 75 °C.

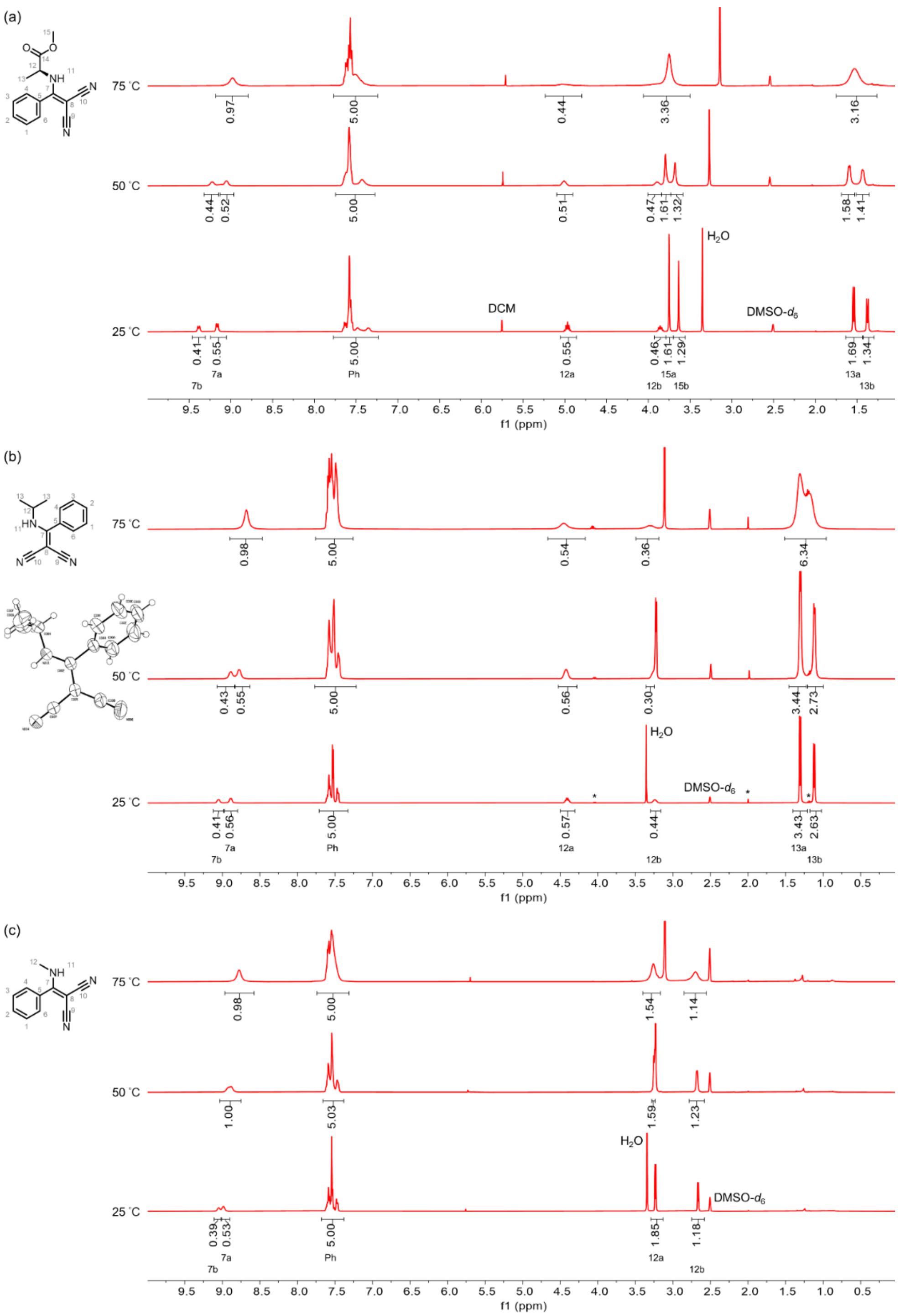

Fig. S10. Characterization of N-linked dicyanovinyl molecules.

These molecules are analogues of **7**. ^1H NMR spectra in DMSO-d_6 at 25, 50 or 75 °C are shown for the amino nucleophile as alanine methyl ester (a), isopropyl amine (b) or methyl amine (c). The identity of the isopropyl amine analogue (b) is also supported by X-ray crystallography (CCDC deposit number: 2347073).

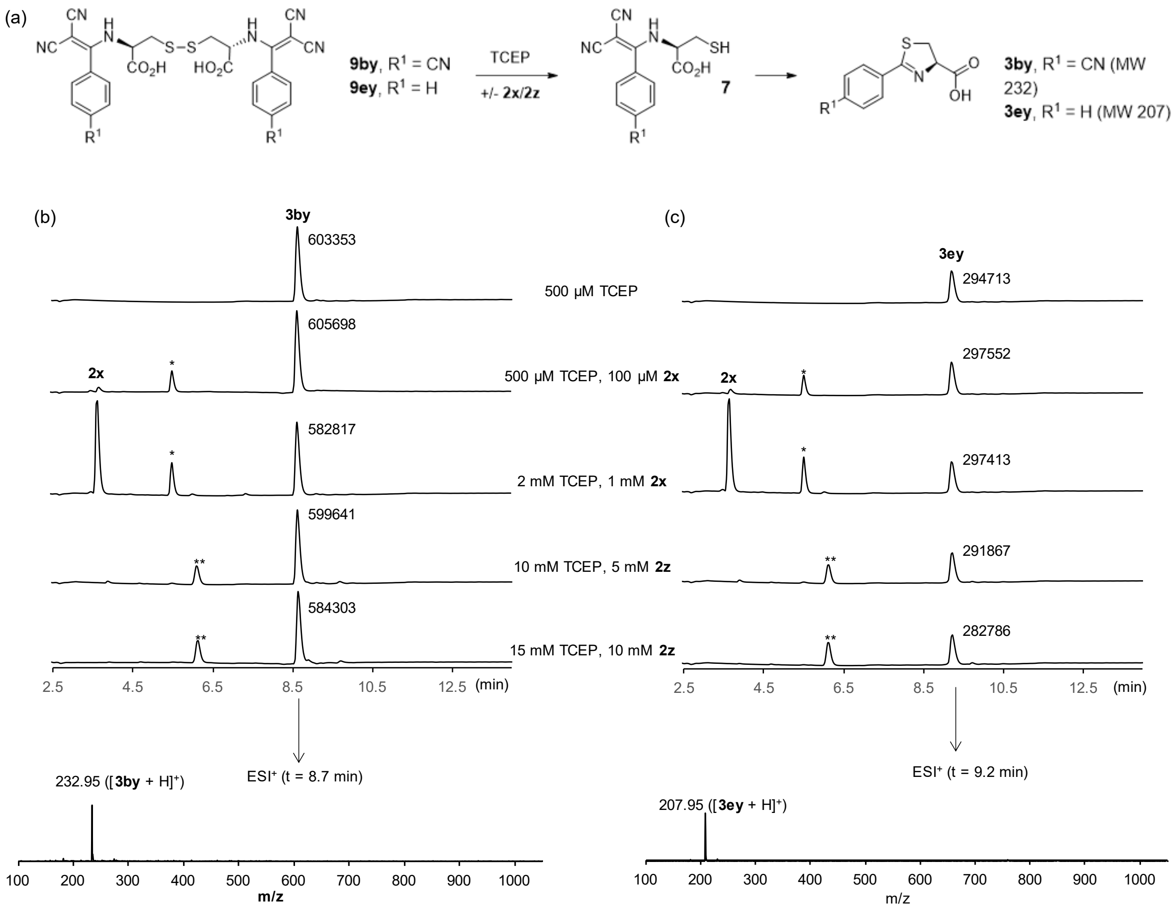

<u>Fig. S11. Transformation of **Int-1** to **3** unaffected by other 1,2-aminothiol molecules.</u>
The depicted disulfide dimer **9** (50 µM) was mixed with or without peptide **2x** or H-Cys-OMe **2z** before TCEP addition. As one molecule of **9** leads to two molecules of **7**. The competing 1,2-aminothiol was tested at 1 (100 µM), 10 (1 mM), 50 (5 mM) or 100 (10 mM) equivalents. After 12 hours, the reaction mixtures were analyzed by LC-MS, and the integration of the product peaks (absorbance at 254 nm) was shown.

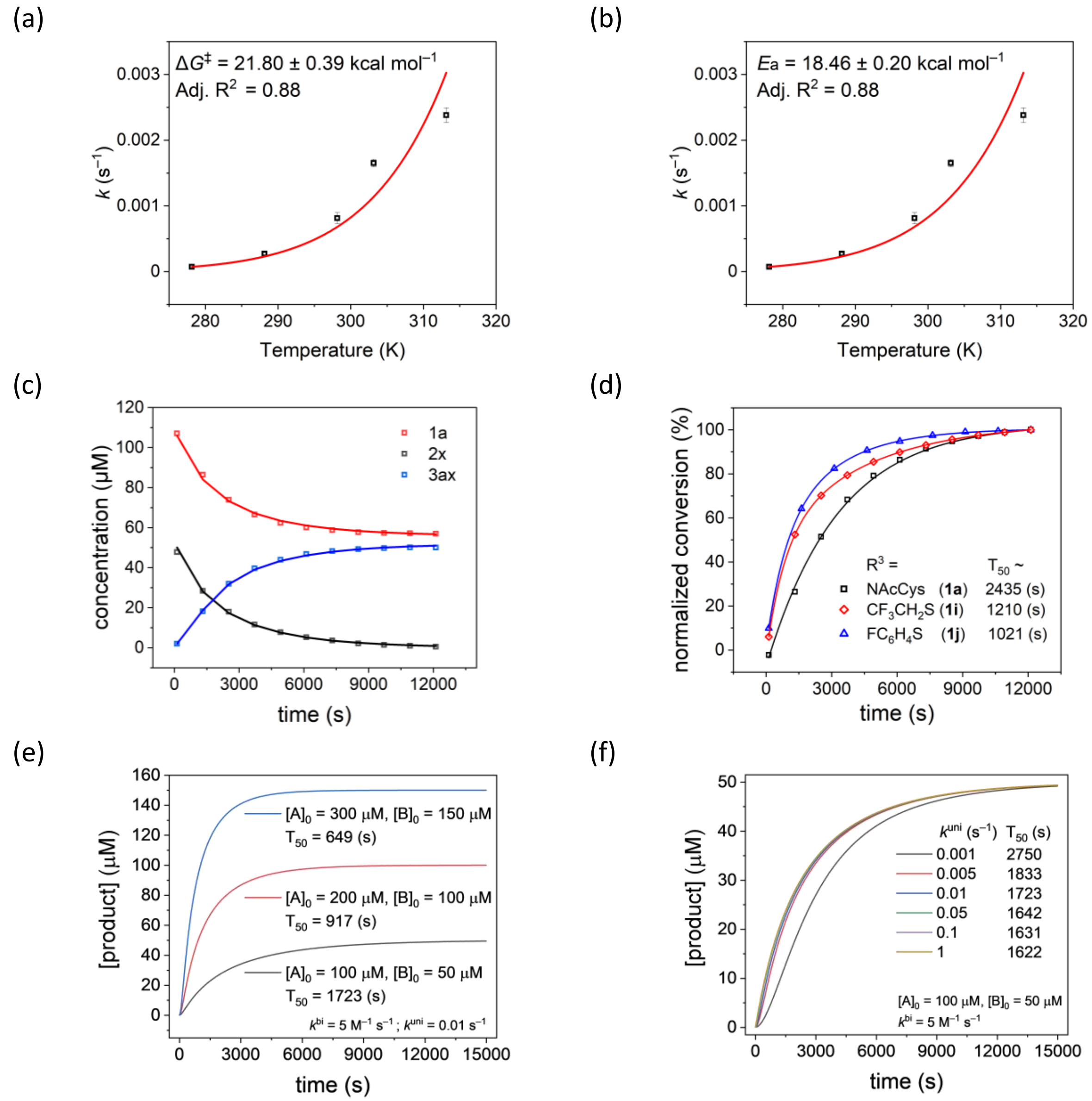

Fig. S12. Kinetic parameters.

Eyring (a) and Arrhenius (b) plots for the reaction of **Int-1** → **3** at pH 7.4. (c) Time evolution of [**1a**], [**2x**], and [**3ax**] (empty squares: data points, solid lines: fitting curves to the model of a two-step **1a** + **2x** → Int → **3ax** reaction). (d) Time evolution of **3ax** yields from reactions between **2x** and TAMM **1a**, **1i**, or **1j**. (e, f) Simulated time profiles of the product concentration for a model reaction $\mathbf{A} + \mathbf{B} \xrightarrow{k^{bi}} \mathbf{C} \xrightarrow{k^{uni}} \mathbf{D}$. Here, species A, B, C, and D correspond to the TAMM reagent, peptide, Int-1 intermediate, and dihydrothiazole product, respectively, of the labeling reaction in the current study. The parameter T_{50} is defined as the time required for the product concentration to reach 50% of its maximum value. (e) Time profiles for reactions with $k^{bi} = 5\ M^{-1}\ s^{-1}$, $k^{uni} = 0.01\ s^{-1}$, and initial concentrations $[A]_0 = 100$ μM, $[B]_0 = 50$ μM, as well as reactions with two- or three-fold higher starting material concentrations. (f) Time profiles for reactions with $k^{bi} = 5\ M^{-1}\ s^{-1}$, initial concentrations $[A]_0 = 100$ μM, $[B]_0 = 50$ μM , and k^{uni} varying from 0.001 to 1 s^{-1}.

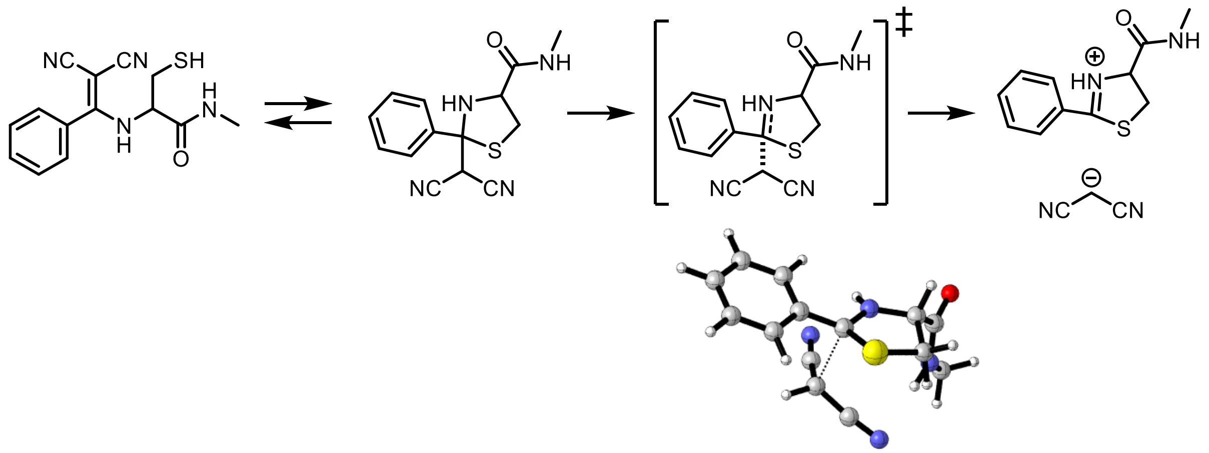

Fig. S13. Conversion of **Int-1** to dihydrothiazole **3**.

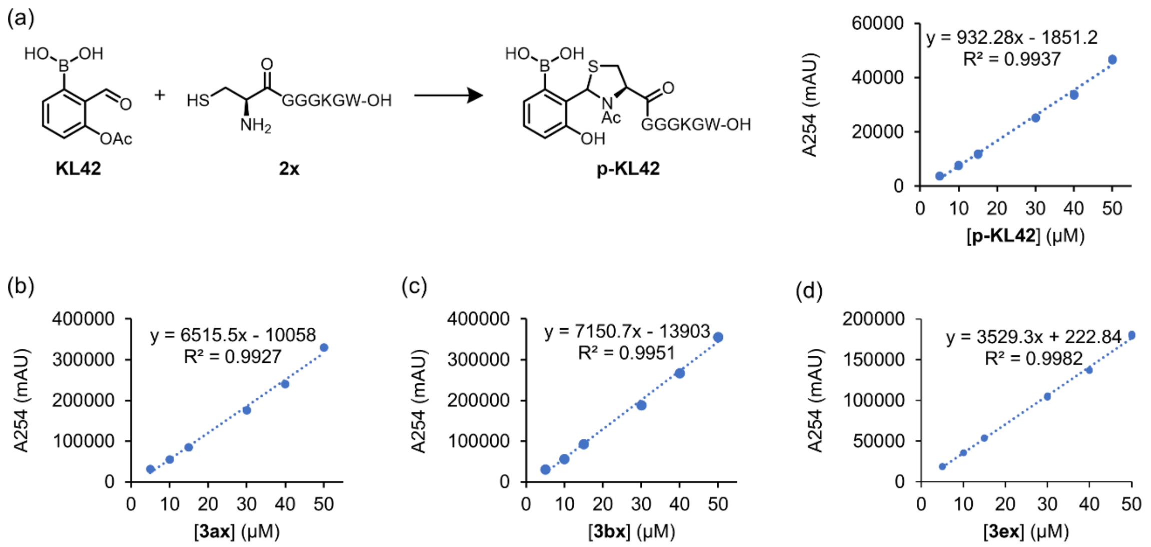

Fig. S14. Standard curve of **p-KL42** (a), **3ax** (b), **3bx** (c) and 3**bex** (d).
Structure of **p-KL42** from the reaction of peptide **2x** and **KL42** is also shown in (a).

(a)

[**1b**]/[**KL42**]	[**3bx**]/[**p−KL42**]	k_{obs} (M^{-1} s^{-1})
2	0.0720±0.0003	~ 919
3	0.105±0.002	~ 935
4	0.136±0.002	~ 957

k_{obs} of **KL42** at pH 7.4 ~ 937±16 M^{-1} s^{-1}

(b)

[**1i**]/[**KL42**]	[**3ax**]/[**p−KL42**]	k_{obs} (M^{-1} s^{-1})
1	1.75±0.10	~ 1790
2/3	1.15±0.07	~ 1740
1/2	0.85±0.04	~ 1690

k_{obs} of **1i** at pH 7.4 ~ 1740±40 M^{-1} s^{-1}

(c)

[**1j**]/[**KL42**]	[**3ax**]/[**p−KL42**]	k_{obs} (M^{-1} s^{-1})
1	1.04±0.02	~ 984
2/3	0.67±0.03	~ 941
1/2	0.54±0.01	~ 1020

k_{obs} of **1j** at pH 7.4 ~ 982±32 M^{-1} s^{-1}

(d)

[**1k**]/[**KL42**]	[**3ex**]/[**p−KL42**]	k_{obs} (M^{-1} s^{-1})
1	1.35±0.01	~ 1330
2/3	0.92±0.02	~ 1350
1/2	0.59±0.01	~ 1140

k_{obs} of **1k** at pH 7.4 ~ 1270±95 M^{-1} s^{-1}

(e)

[**1l**]/[**KL42**]	[**3bx**]/[**p−KL42**]	k_{obs} (M^{-1} s^{-1})
1	4.45±0.02	~ 5110
2/3	3.32±0.16	~ 5660
1/2	2.54±0.17	~ 5690

k_{obs} of **1l** at pH 7.4 ~ 5490±270 M^{-1} s^{-1}

(f)

[**1m**]/[**KL42**]	[**3bx**]/[**p−KL42**]	k_{obs} (M^{-1} s^{-1})
1	4.94±0.06	~ 5720
2/3	3.32±0.08	~ 5660
1/2	2.42±0.07	~ 5380

k_{obs} of **1l** at pH 7.4 ~ 5590±150 M^{-1} s^{-1}

(g)

[**1n**]/[**KL42**]	[**3bx**]/[**p−KL42**]	k_{obs} (M^{-1} s^{-1})
1/6	2.25±0.03	~ 15300
1/9	1.51±0.04	~ 15000
1/12	1.13±0.01	~ 14600

k_{obs} of **1l** at pH 7.4 ~ 15000±300 M^{-1} s^{-1}

Fig. S15. Rate constant determination
(a) Determining the rate constants ($k_{obs}^{7.4}$) of (3-acetoxy-2-formylphenyl)boronic acid (**KL42**) through competition with TAMM **1b**. (b-g) Determining the rate constants ($k_{obs}^{7.4}$) of TAMM **1i**–**1n** through competition with **KL42**. Product ratios are shown as mean ± standard deviation calculated from three experiments.

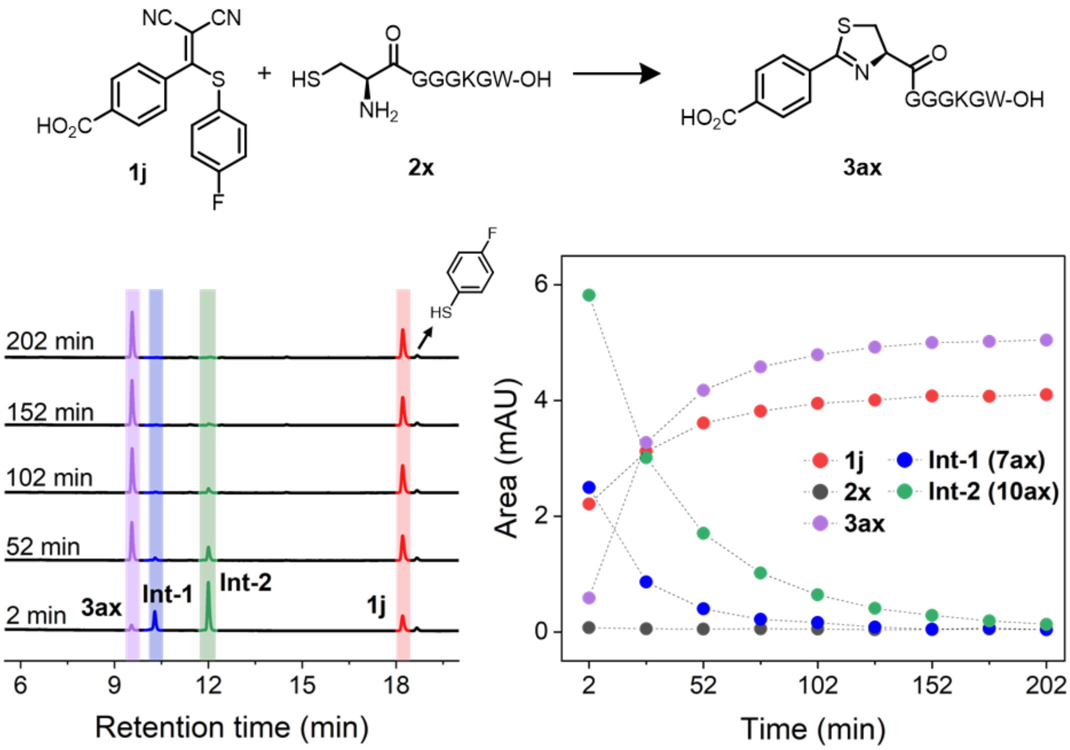

Fig. S16. Reaction of TAMM **1j** and peptide **2x** to form dihydrothiazole **3ax**.
Bottom left: HPLC chromatograms for the reaction mixture analyzed at the time specified.
Bottom right: Time course of the HPLC peak intensities. The areas under the peaks, as opposed to the concentrations are shown here as the calibration of **Int-1**, an unisolable compound, is not available. The dashed lines connecting data points serve as a guide to the eye.

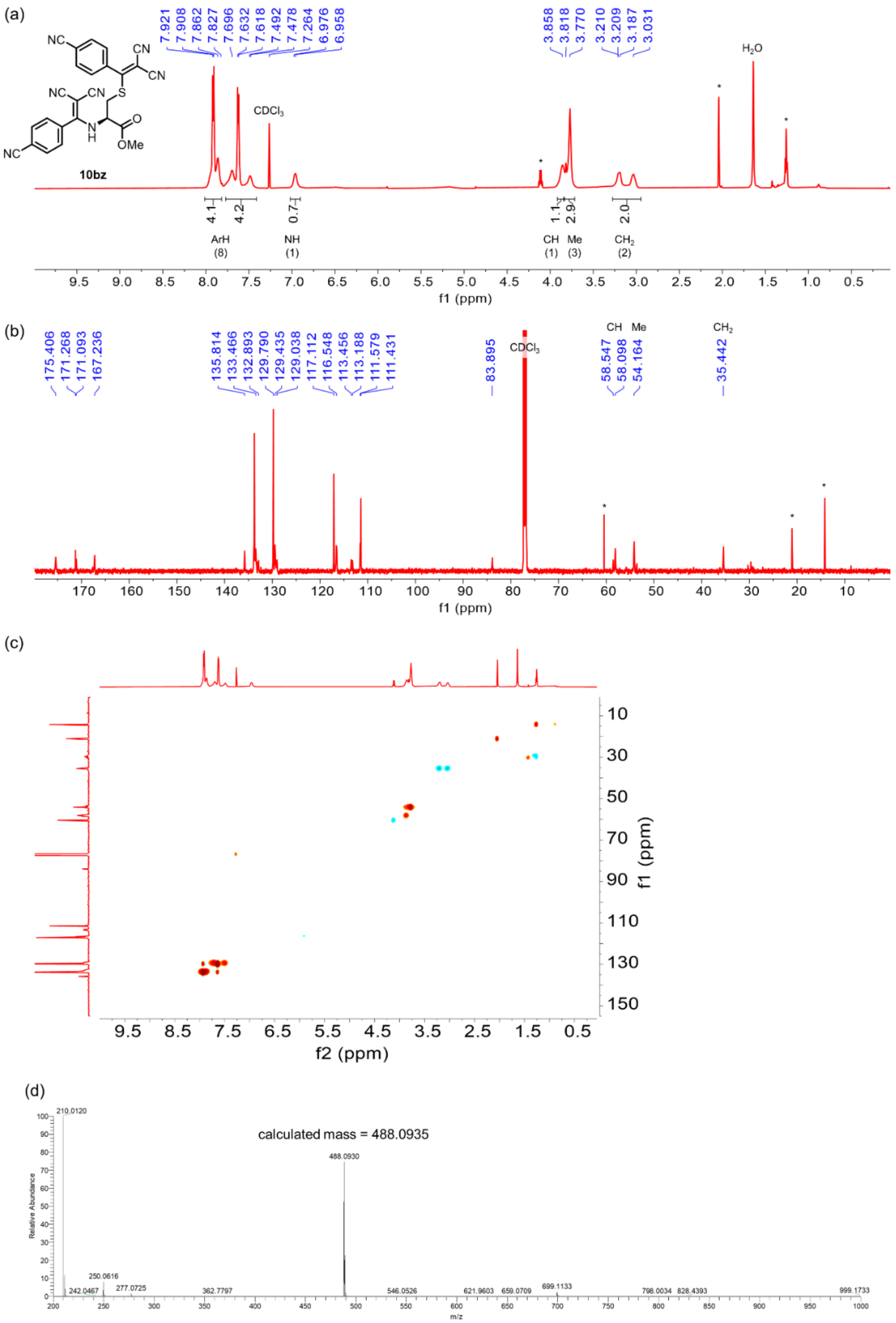

Fig. S17. Characterization of **10bz** from reaction of **1l** and **2z**.
(a-c) ^{1}H, ^{13}C and HSQC NMR spectra in $CDCl_3$ at 25 °C. * indicate peaks of EtOAc. (d) HR-MS.

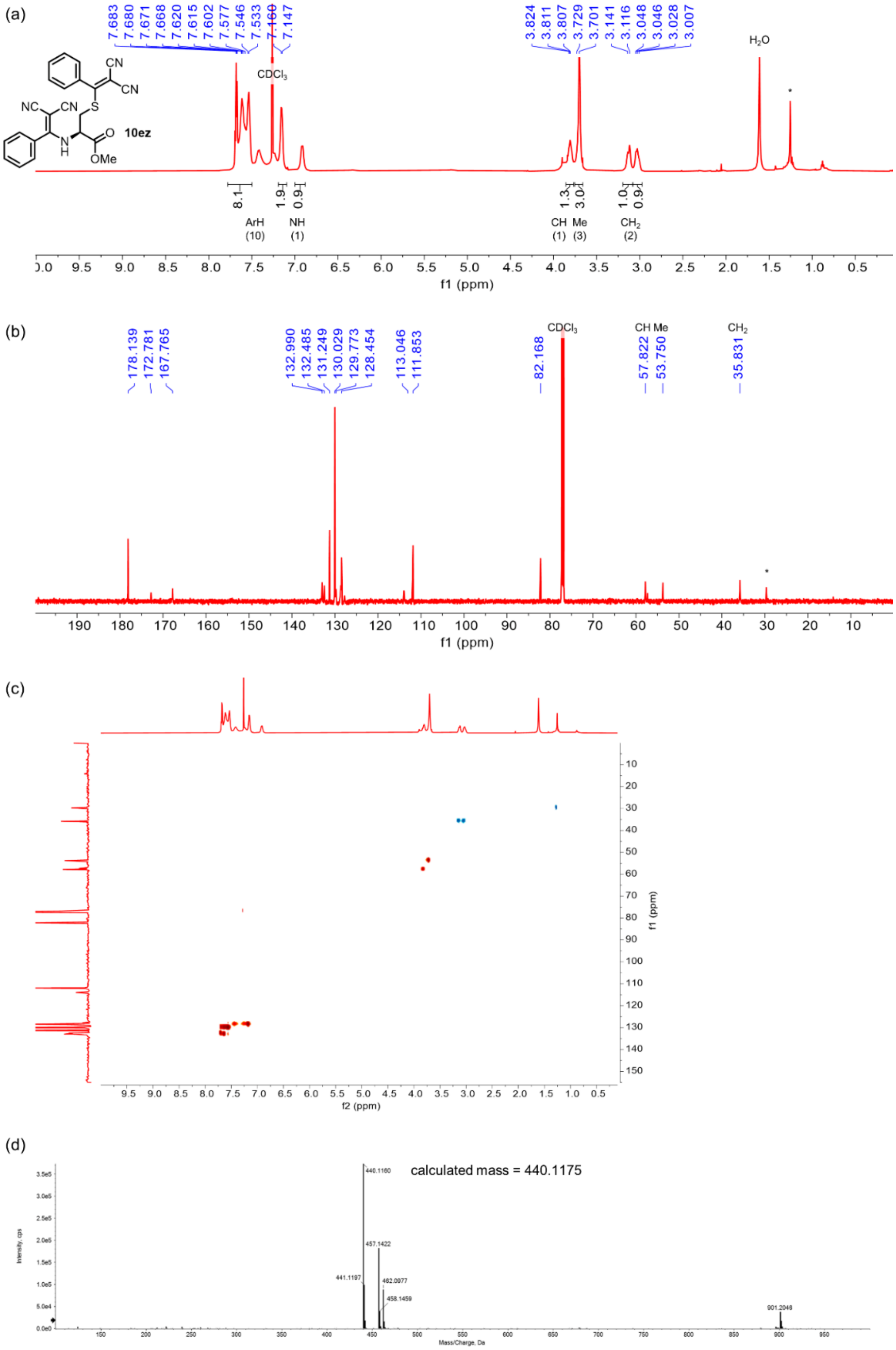

Fig. S18. Characterization of **10ez** from reaction of **1k** and **2z**.

(a-c) ^{1}H, ^{13}C and HSQC NMR spectra in $CDCl_3$ at 25 °C. * indicate peaks of grease. (d) HR-MS.

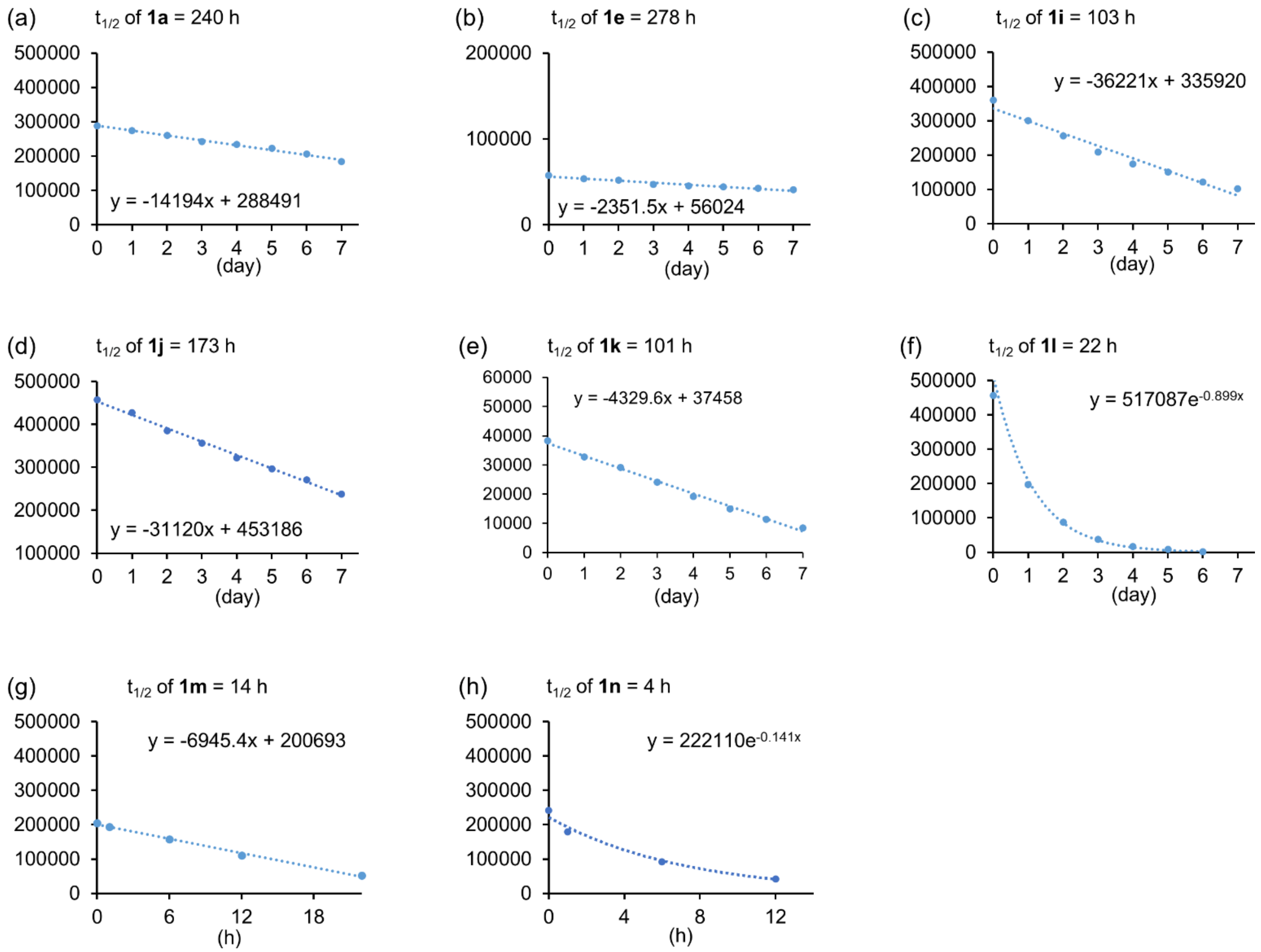

<u>Fig. S19. Aqueous stability of TAMM molecules.</u>

Aqueous stability of TAMM molecules **1a** (a), **1e** (b), **1i** (c), **1j** (d), **1k** (e), **1l** (f), **1m** (g) and **1n** (h). Amount of a given TAMM **1** (100 μM) in PBS (0.01 M, pH 7.4) at 37 °C was quantified at the indicated time points by HPLC. The integration of the peak area (y-axis) was plotted against individual time points (x-axis), and a fitting curve was obtained for calculating the half-life ($t_{1/2}$), at which time point [**1**] = $[\mathbf{1}]_0/2$.

(a)

[malononitrile] (μM)	0	500	100	50	10	5	1
Cell viability (%)	100.0±11	107.4±12.9	102.2±9.7	102.3±2.3	111.4±5.9	101.7±4.2	100.7±4.5
P value in compassion to the blank	N/A	0.406	0.770	0.703	0.199	0.782	0.913
Statistical significance	N/A	No	No	No	No	No	No

(b)

[**PEG-TAMM-SCH$_2$CF$_3$**] (μM)	0	500	100	50	10	5	1
Cell viability (%)	100.0±4.2	90.5±2.4	104.3±2.5	108.7±0.8	103.3±1.1	104.0±1.8	102.4±0.9
P value in compassion to the blank	N/A	0.051	0.280	0.096	0.346	0.289	0.476
Statistical significance	N/A	No	No	No	No	No	No

Fig. S20. Effects of malononitrile and **PEG-TAMM-SCH$_2$CF$_3$** on cell viability.

Effects of malononitrile (a) and **PEG-TAMM-SCH$_2$CF$_3$** (b) on HEK293T cell viability determined using CCK-8. **PEG-TAMM-SCH$_2$CF$_3$**, a cell-impermeable molecule, was used as a model TAMM molecule to assess the impact of cell-impermeable TAMM conjugates on cell viability. N/A = not applicable.

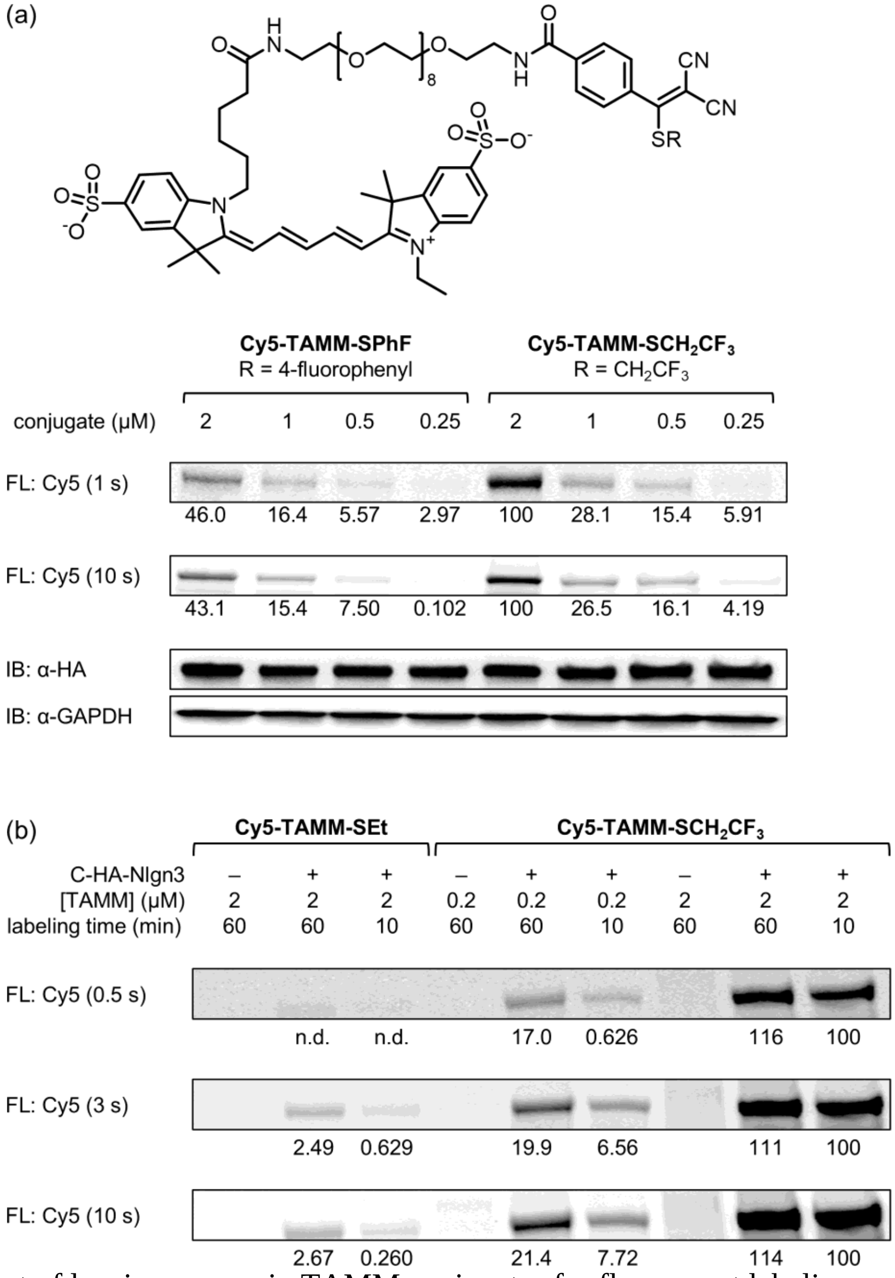

Fig. S21. Effect of leaving groups in TAMM conjugates for fluorescent labeling.

Comparison of different leaving groups in TAMM conjugates for fluorescent labeling of a cell-surface protein on live mammalian cells. HEK293T cells were transiently transfected with a plasmid for overexpressing C-HA-Nlgn3. After 48 hours, cells were incubated with the indicated concentration of the TAMM conjugate in LCIS. Cells were then washed and lysed. Proteins were collected, denatured and separated on a 4-20% SDS-PAGE before fluorescence imaging. (a) In-gel fluorescence analysis of C-HA-Nlgn3 labeled with **Cy5-TAMM-SPhF** or **Cy5-TAMM-SCH_2CF_3** for 10 minutes. (b) Quantification of the in-gel fluorescence of **Fig. 2B**. Relative quantification of the fluorescence signals was shown at the bottom. Fluorescence intensity of samples labeled with 2 μM **Cy5-TAMM-SCH_2CF_3** for 10 minutes was defined as 100 (n.d. = not detectable by the Image Lab software).

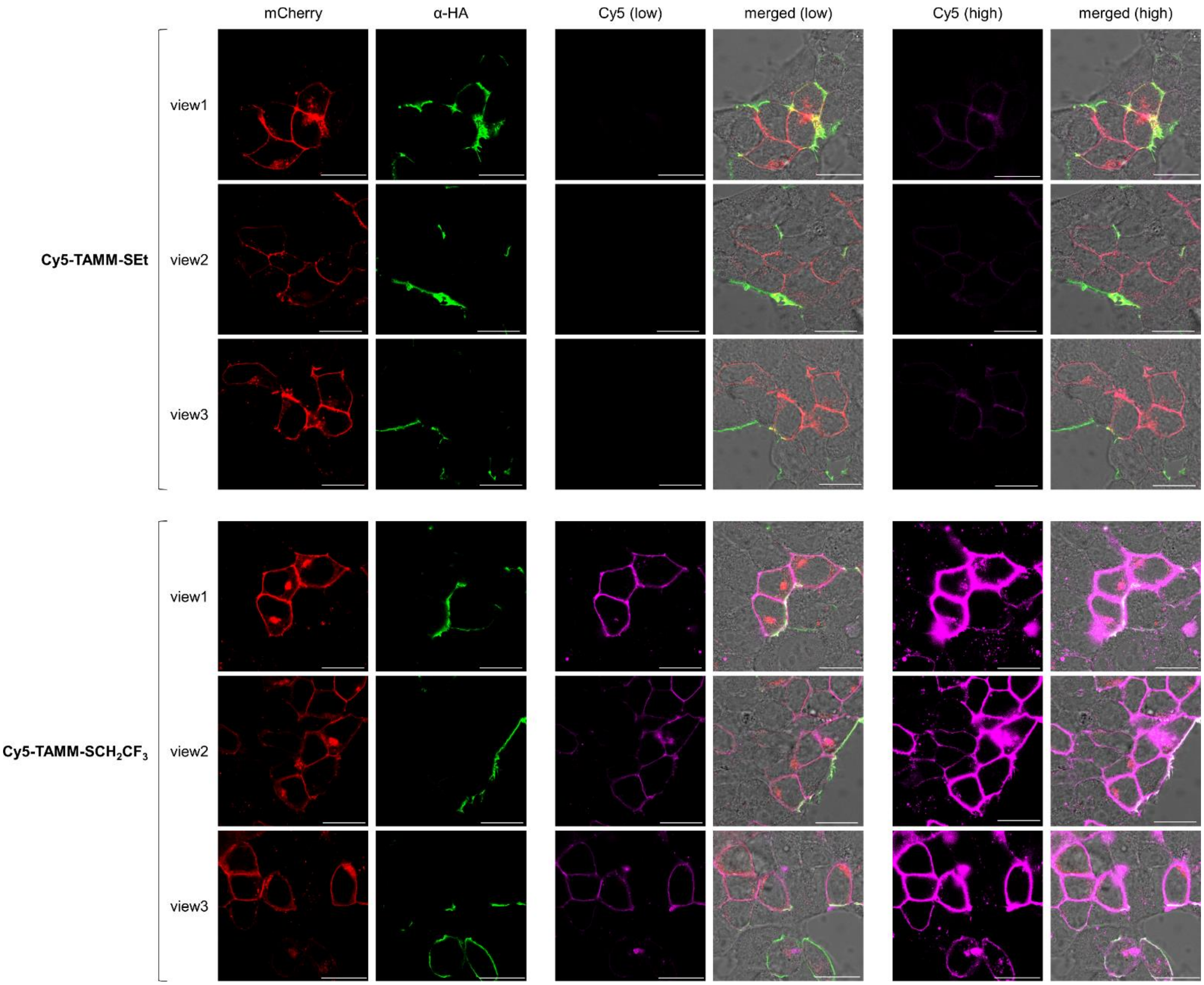

Fig. S22. Representative images of labeling C-HA-Nlgn3-mCherry.

Representative confocal microscopy images of C-HA-Nlgn3-mCherry labeled with **Cy5-TAMM-SEt** or **Cy5-TAMM-SCH$_2$CF$_3$**. HEK293T cells were transiently transfected with a plasmid for overexpressing C-HA-Nlgn3-mCherry. After 48 hours, cells were incubated with 2 μM **Cy5-TAMM-SEt** or **Cy5-TAMM-SCH$_2$CF$_3$** in live cell imaging solution for 60 minutes, washed and analyzed by confocal microscopy. Scale bar = 20 μm.

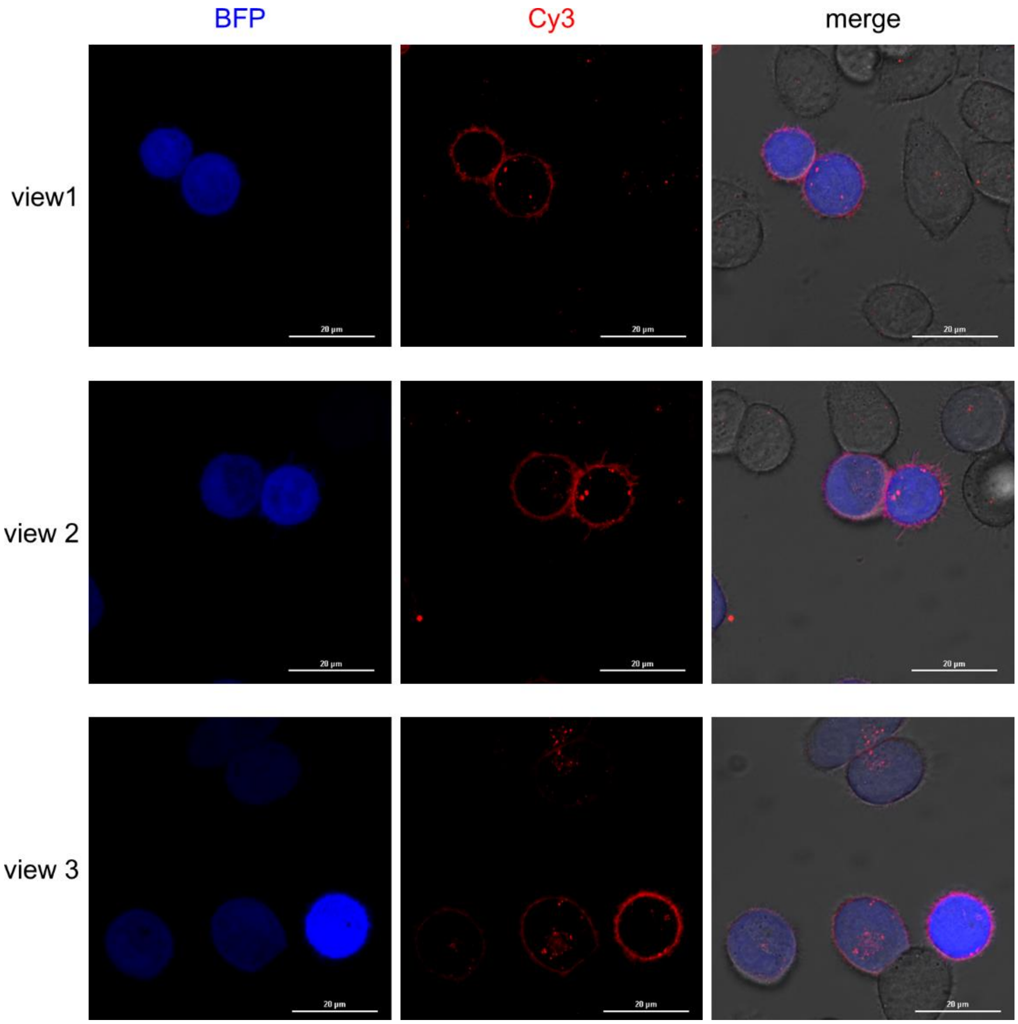

Fig. S23. Representative images of labeling in HeLa cells.

Representative confocal microscopy images of HeLa cells overexpressing BFP-P2A-C-HA-EGFR labeled with **Cy5-TAMM-SCH_2CF_3**. Scale bar = 20 µm.

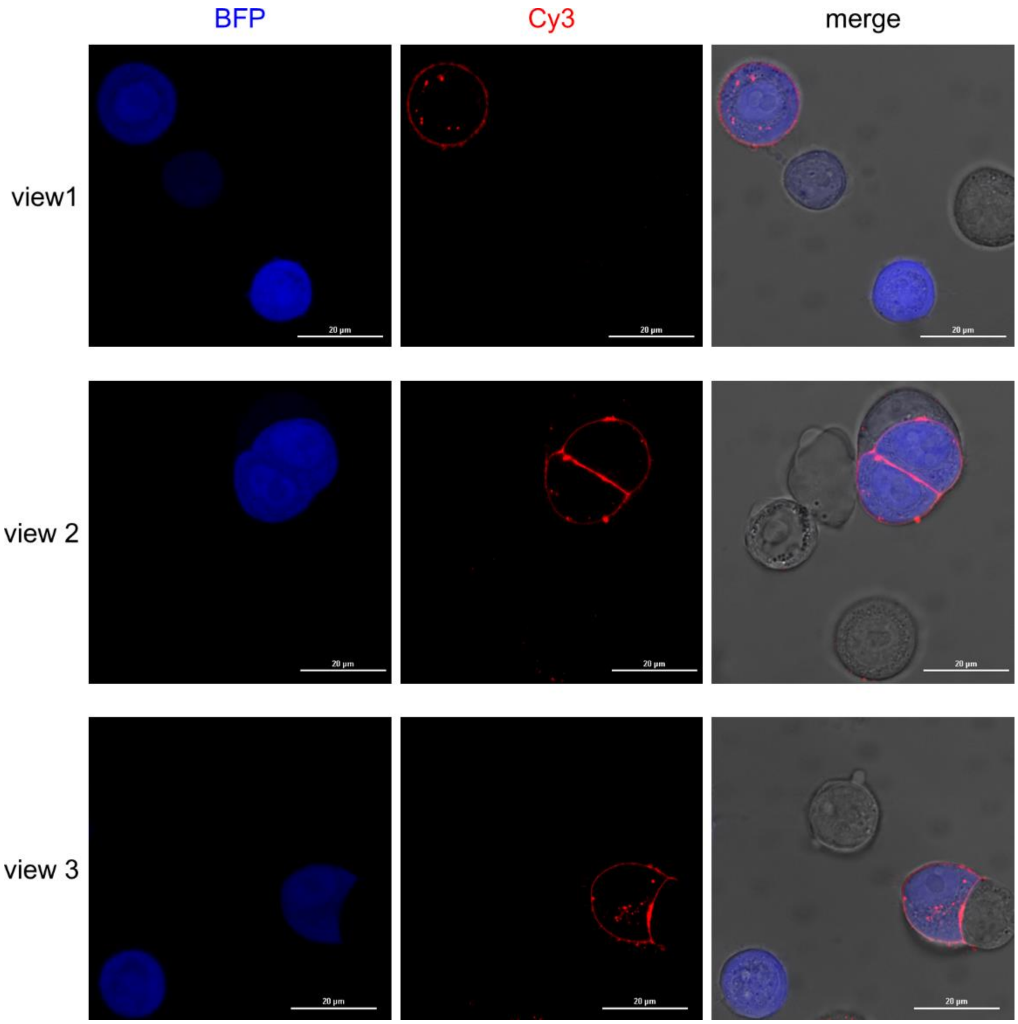

Fig. S24. Representative images of labeling in MCF7 cells.

Representative confocal microscopy images of MCF7 cells overexpressing BFP-P2A-C-HA-EGFR labeled with **Cy5-TAMM-SCH$_2$CF$_3$**. Scale bar = 20 μm.

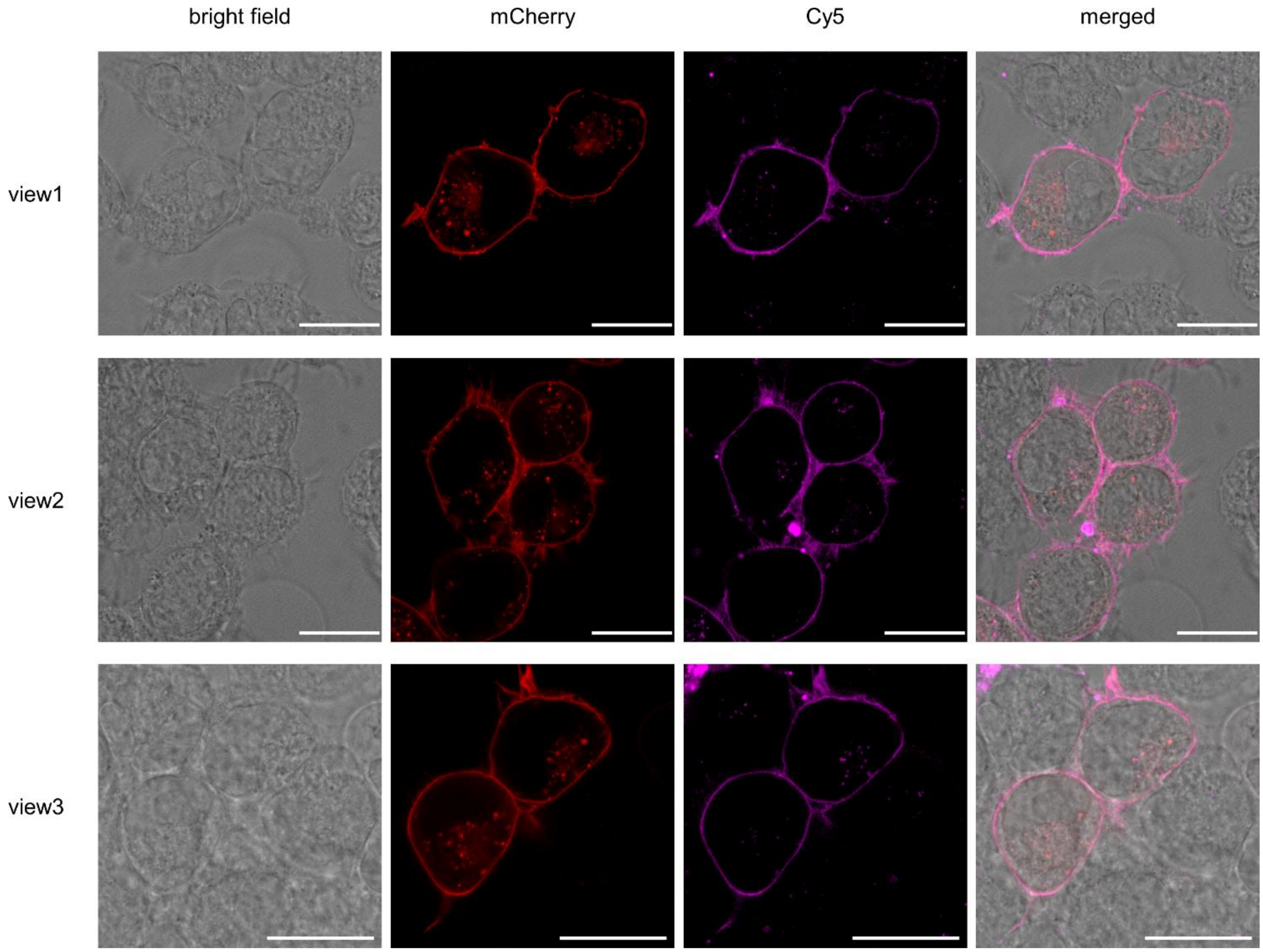

<u>Fig. S25. Representative images of labeling in ND7/23 cells.</u>

Representative confocal microscopy images of ND7/23 cells overexpressing C-HA-Nlgn3-mCherry labeled with **Cy5-TAMM-SCH$_2$CF$_3$**. Scale bar = 20 μm.

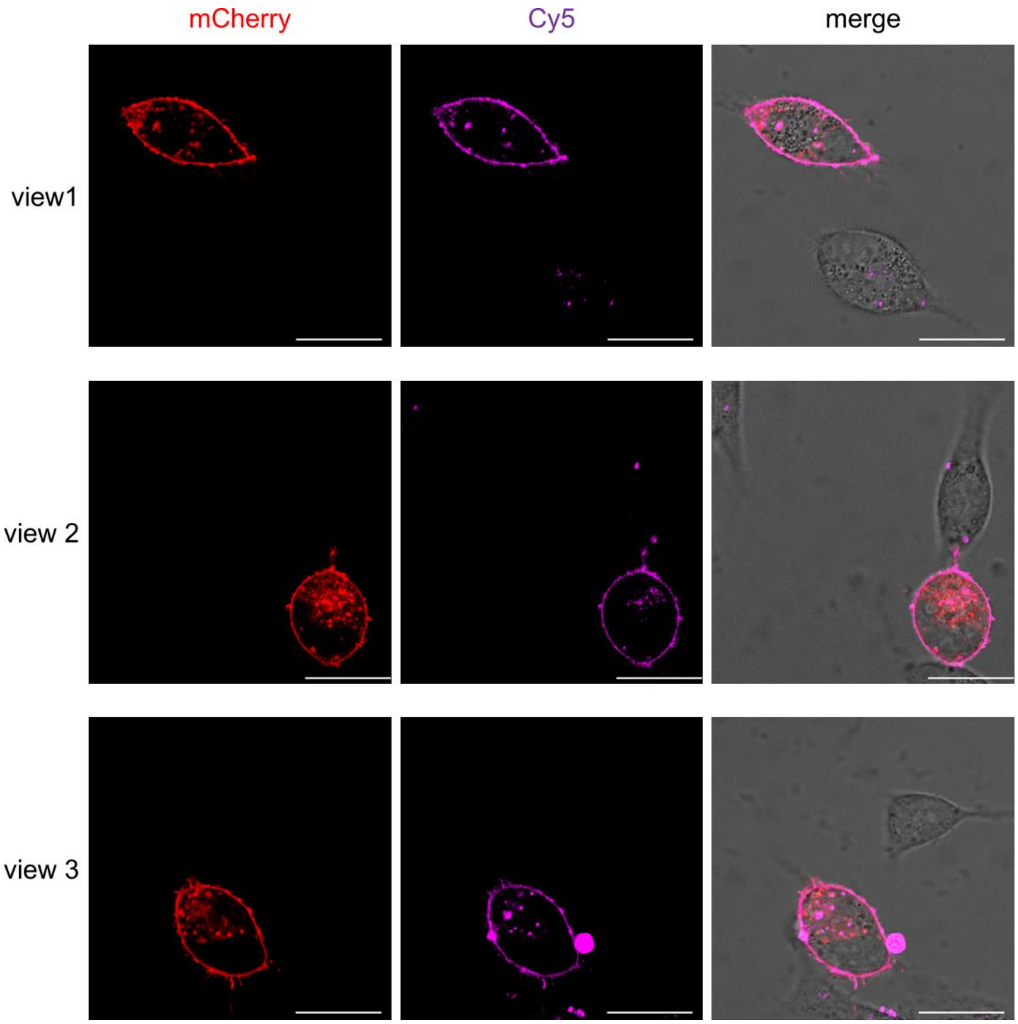

Fig. S26. Representative images of labeling in SK-OV-3 cells.

Representative confocal microscopy images of SK-OV-3 cells overexpressing C-HA-EGFR-mCherry labeled with **Cy5-TAMM-SCH_2CF_3**. Scale bar = 20 μm.

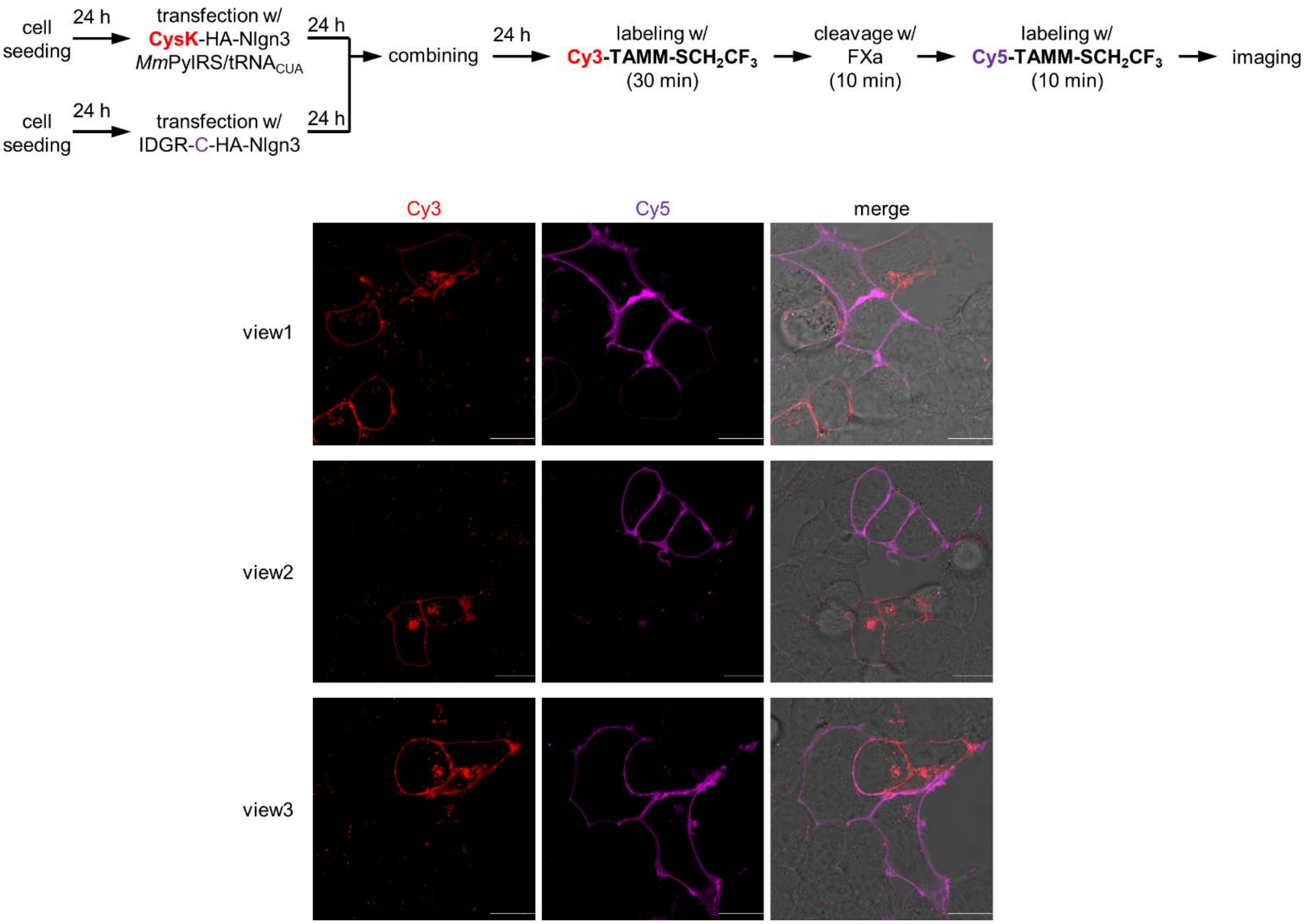

<u>Fig. S27. Dual TAMM condensation for labeling two populations of cells.</u>

Representative images of sequential TAMM condensation for dual-color fluorescent labeling of two different target proteins expressed on two different populations of HEK293T cells. Cells were transiently transfected with plasmid for expressing IDGR-C-HA-Nlgn3 or **CysK**-HA-CHRM3. After 24 hours, the two sets of cells were mixed and further cultured for another 24 hours. For expression of **CysK**-HA-CHRM3, plasmids encoding TAG-HA-CHRM3 and *Mm*PylRS/$tRNA_{CUA}$ were used, and cells were cultured in the presence of 1 mM **CysK-OMe** for 48 hours before fluorescent labeling with 2 μM **Cy3-TAMM-SCH_2CF_3** for 30 minutes. Cells were then treated with Factor Xa protease for 10 minutes to expose the N-terminal cysteine residue of Nlgn3, followed by labeling with 2 μM **Cy5-TAMM-SCH_2CF_3** for 10 minutes before imaging. Scale bar = 20 μm.

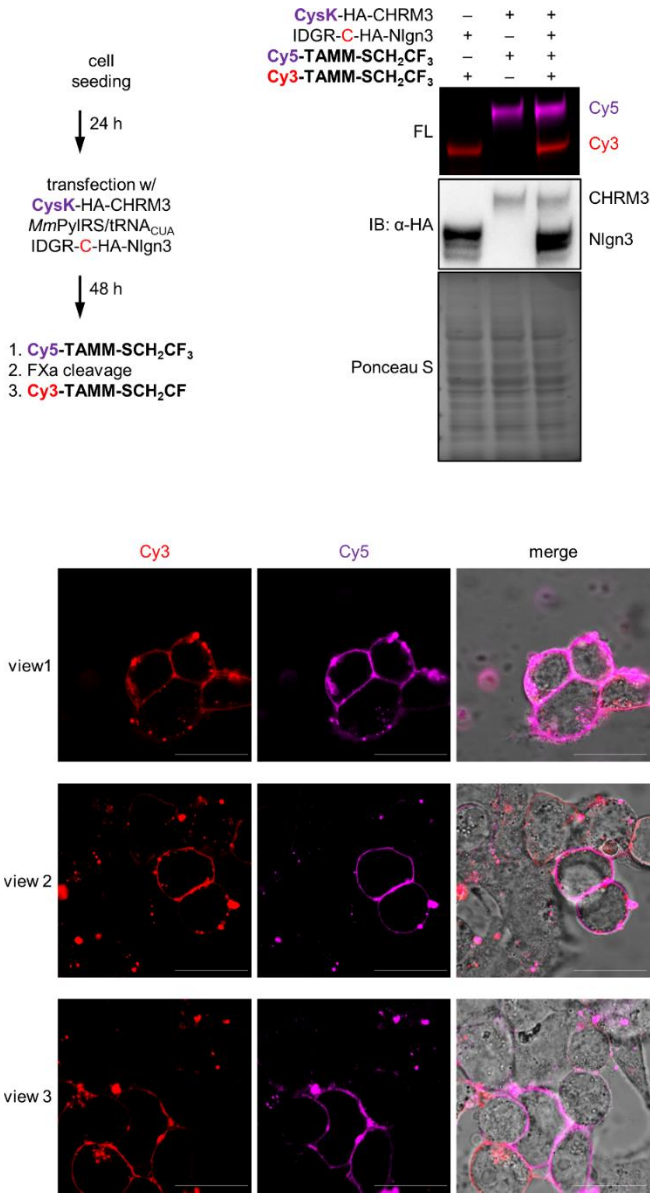

Fig. S28. Dual TAMM condensation for labeling two targets on the same cells.

Representative images of sequential TAMM condensation for dual-color fluorescent labeling of two different target proteins expressed on the same cells. HEK293T cells were transiently transfected with plasmid for expressing IDGR-C-HA-Nlgn3, **CysK**-HA-CHRM3, as well as *Methanosarcina mazei* pyrrolysyl-tRNA synthetase (*Mm*PylRS) and its cognate $tRNA_{CUA}$ for decoding the amber codon (UAG) to **CysK**. Cells were cultured in the presence of 1 mM **CysK-OMe** for 48 hours before fluorescent labeling with 2 μM **Cy5-TAMM-SCH$_2$CF$_3$** for 30 minutes. Cells were then treated with Factor Xa protease for 10 minutes to expose the N-terminal cysteine residue of Nlgn3, followed by labeling with 2 μM **Cy3-TAMM-SCH$_2$CF$_3$** for 10 minutes before imaging. After imaging, proteins were collected, denatured and separated on a 4-20% SDS-PAGE before in-gel fluorescent analysis. Ponceaus S staining is shown as the loading control. Scale bar = 20 μm.

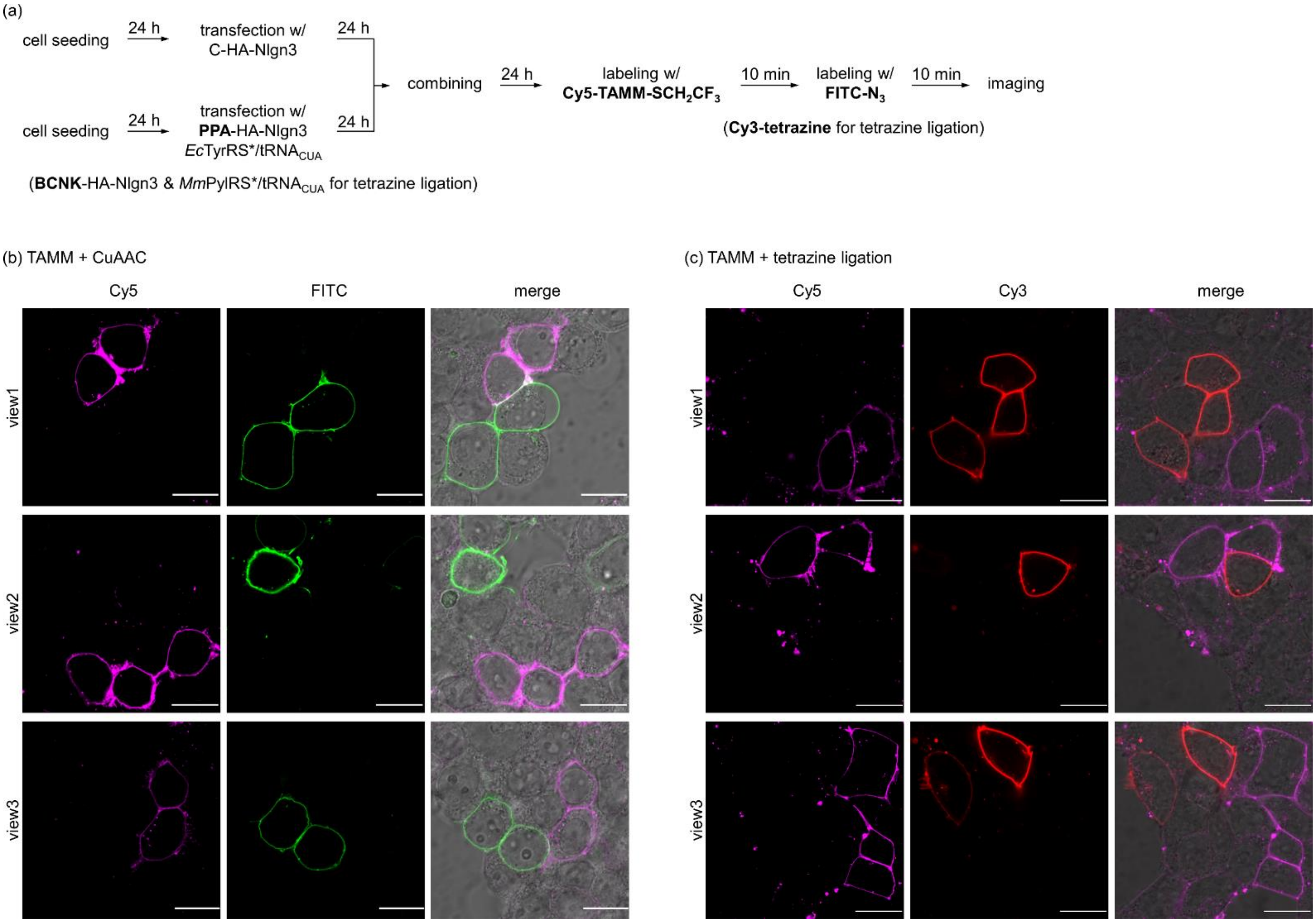

Fig. S29. Compatibility of TAMM condensation to CuAAC and tetrazine ligation

Using TAMM condensation and CuAAC or tetrazine ligation for dual-color fluorescent labeling. (a) Schematic presentation of the experimental flow. (b-c) Representative confocal microscopy images of dual labeling by TAMM condensation and CuAAC (b) or tetrazine ligation (c). Scale bar = 20 μm.

(a) 1 μM **Cy3-tetrazine**

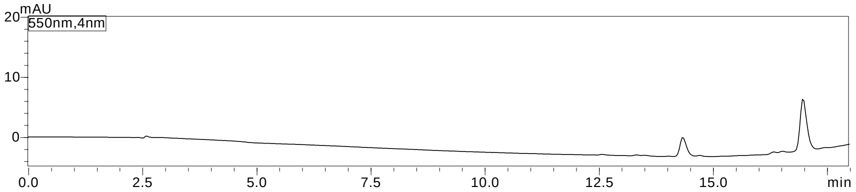

(b) 1 μM **Cy3-tetrazine**, 1.5 μM **BCNK**, PBS (pH 7.4), 37 °C, 10 minutes.

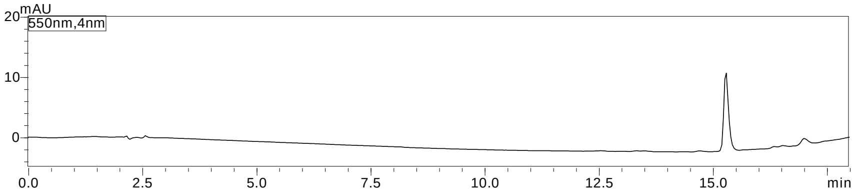

(c) 1 μM **FITC-N3**

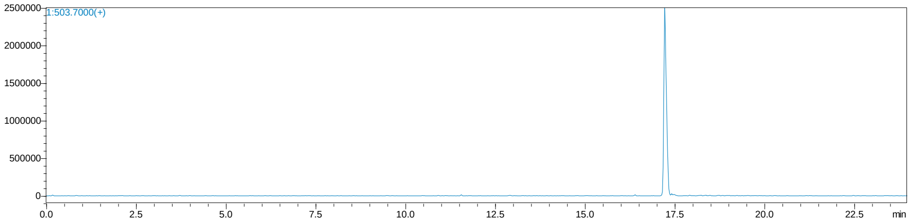

(d) 1 μM **FITC-N3**, 1.5 μM **BCNK**, PBS (pH 7.4), 37 °C, 10 minutes.

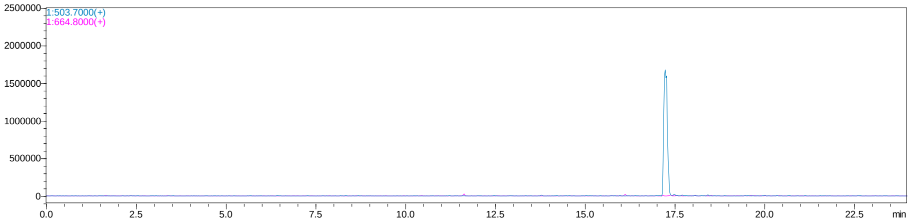

Fig. S30. Verification of the orthogonality of **BCNK** and **FITC-N3.**

Verification of the orthogonality of **BCNK** used in tetrazine ligation and **FITC-N3** used in CuAAC. Chromatograms of **Cy3-tetrazine** (a), mixture of **Cy3-tetrazine** and **BCNK** (b), **FITC-N3** (c), mixture of **FITC-N3** and **BCNK** (d). Chromatography condition: C18 column (Agilent, #770450-902) with water (containing 0.1% v/v formic acid) and methanol as the mobile phases at the flow rate of 1 mL/min, 10% to 40% methanol from 0 to 8 minutes, 40% to 50% methanol from 8 to 12 minutes, 50% to 90% methanol from 12 to 16 minutes, 90% methanol for 2 minutes, 90% to 10% methanol from 18 to 19 minutes, 10% methanol from 19 to 24 minutes. (a, b) Chromatograms of visible absorbance at 550 nm (i.e., absorbance of Cy3). The identity of **Cy3-tetrazine** (retention time = 14.4 and 16.9 minutes, exact mass = 959.3790) was confirmed by m/z of 959.4 found for $[M+H]^+$. The adduct of **Cy3-tetrazine** and **BCNK** with exact mass of 1250.5403 was eluted at 15.3 minutes with m/z of 627.3 found for $[M+2H]^{2+}$. (c-d) TIC chromatograms of 503.7 and 664.8, corresponding to **FITC-N3** (exact mass = 1005.3790) and the SPAAC adduct of **FITC-N3** and BCNK (exact mass = 1327.5682), respectively. FITC has low absorbance under acidic conditions (resulted from the presence of formic acid in the eluent), and no peak was observed by the photo diode array detector.

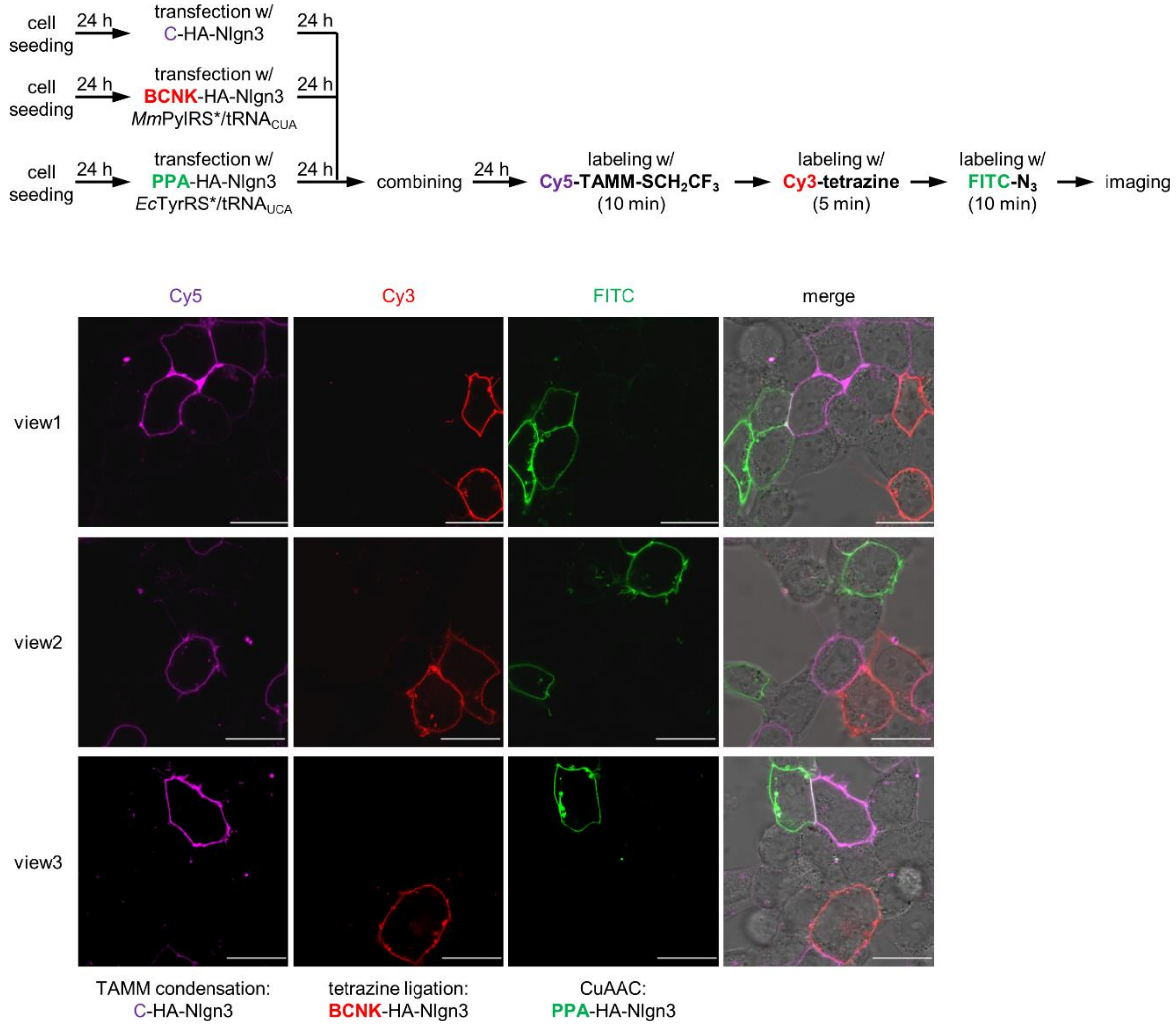

Fig. S31. Labeling three populations of cells.

Using TAMM condensation, CuAAC and tetrazine ligation to label three target proteins on three different populations of HEK293T cells. Schematic presentation of the experimental flow is shown at the top. Two sets of confocal microscopy images are shown at the bottom. HEK293T cells were transiently transfected with plasmids for expressing C-HA-Nlgn3, **BCNK**-HA-Nlgn3 or **PPA**-HA-Nlgn3. After 24 hours, the three sets of cells were mixed and further cultured for another 24 hours. For **BCNK** or **PPA** incorporation, an engineered *M. mazei* pyrrolysyl-tRNA synthetase (*Mm*PylRS*)/$tRNA_{CUA}$ pair or an engineered *E. coli* tyrosyl-tRNA synthetase (*Ec*TyrRS*)/$tRNA_{UCA}$ pair was used, respectively. After transfection, cells were cultured in the presence of the required unnatural amino acid(s) for 48 hours before labeling and fluorescent imaging. Scale bar = 20 μm.

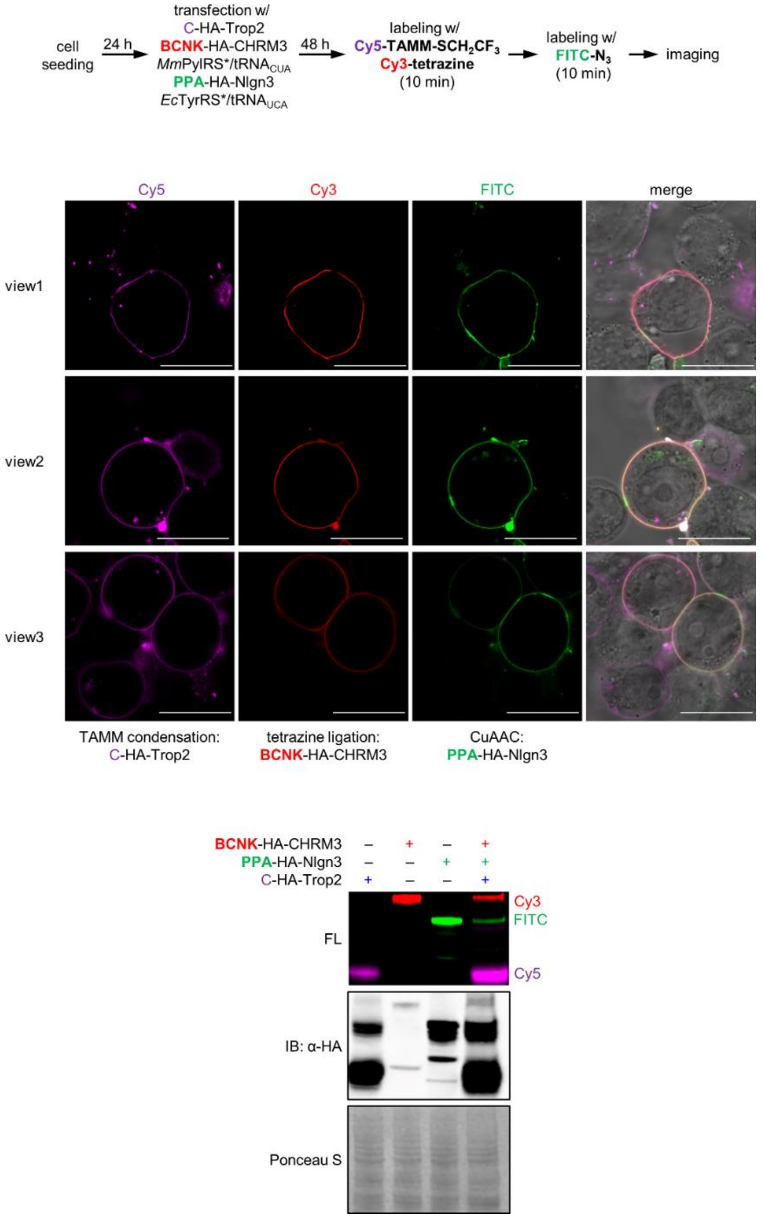

Fig. S32. Labeling three targets on the same cells.

Using TAMM condensation, CuAAC and tetrazine ligation to label three target proteins on the same HEK293T cells. Schematic presentation of the experimental flow is shown at the top. Two sets of confocal microscopy images are shown at the bottom. HEK293T cells were transfected with plasmids for expressing C-HA-Trop2, **BCNK**-HA-CHRM3 and **PPA**-HA-Nlgn3. For **BCNK** or **PPA** incorporation, an engineered *M. mazei* pyrrolysyl-tRNA synthetase (*Mm*PylRS*)/$tRNA_{CUA}$ pair or an engineered *E. coli* tyrosyl-tRNA synthetase (*Ec*TyrRS*)/$tRNA_{UCA}$ pair was used, respectively. After transfection, cells were cultured in the presence of the required unnatural amino acids for 48 hours before labeling and fluorescent imaging. Proteins were also collected for in-gel fluorescent analysis. Scale bar = 20 μm.

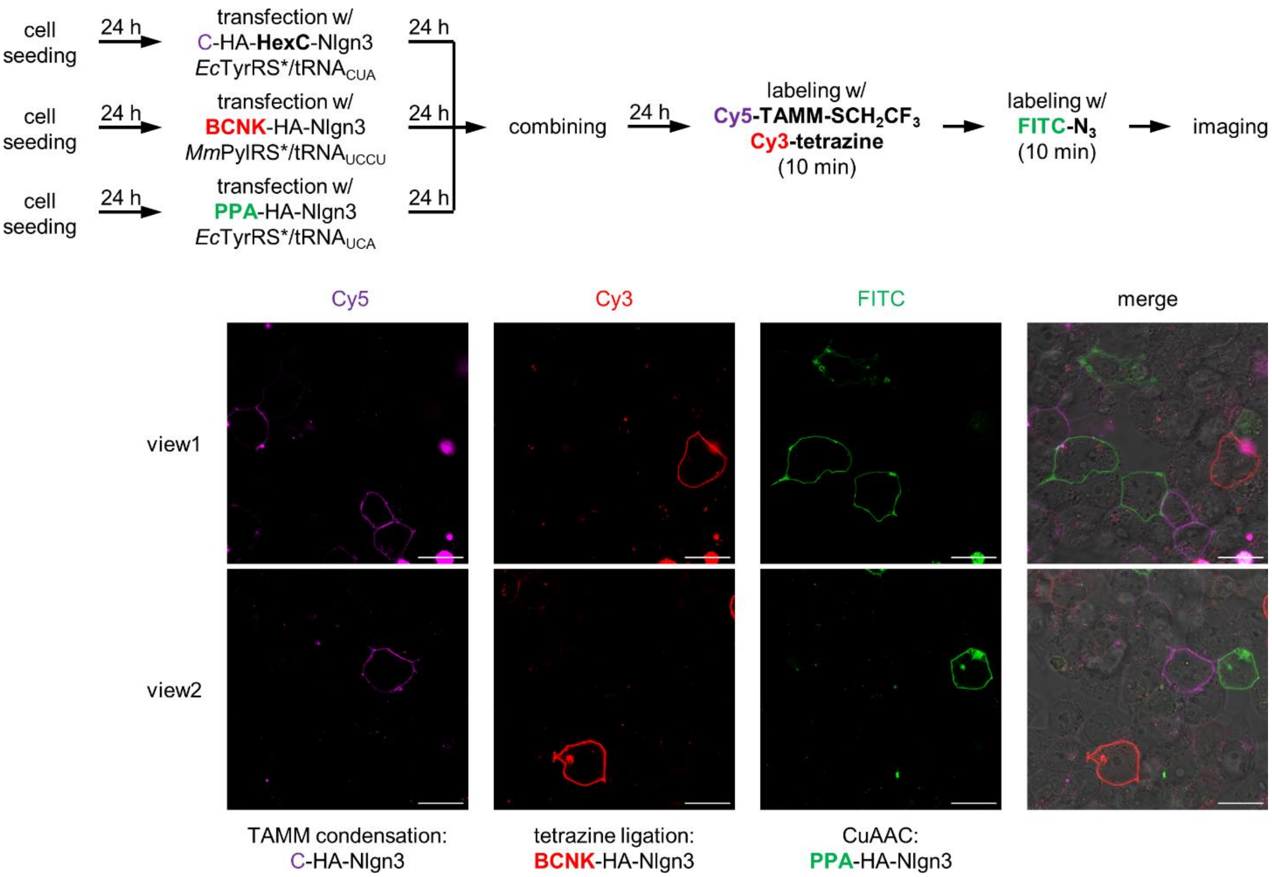

<u>Fig. S33. Using three unnatural amino acids to control the bioorthogonal reactions.</u>

Using three unnatural amino acids to control the bioorthogonal reactions for labeling three target proteins on different HEK293T cells. See also **Fig. 4D**. Schematic presentation of the experimental flow is shown at the top. Representative confocal microscopy images are shown at the bottom. Scale bar = 20 μm.

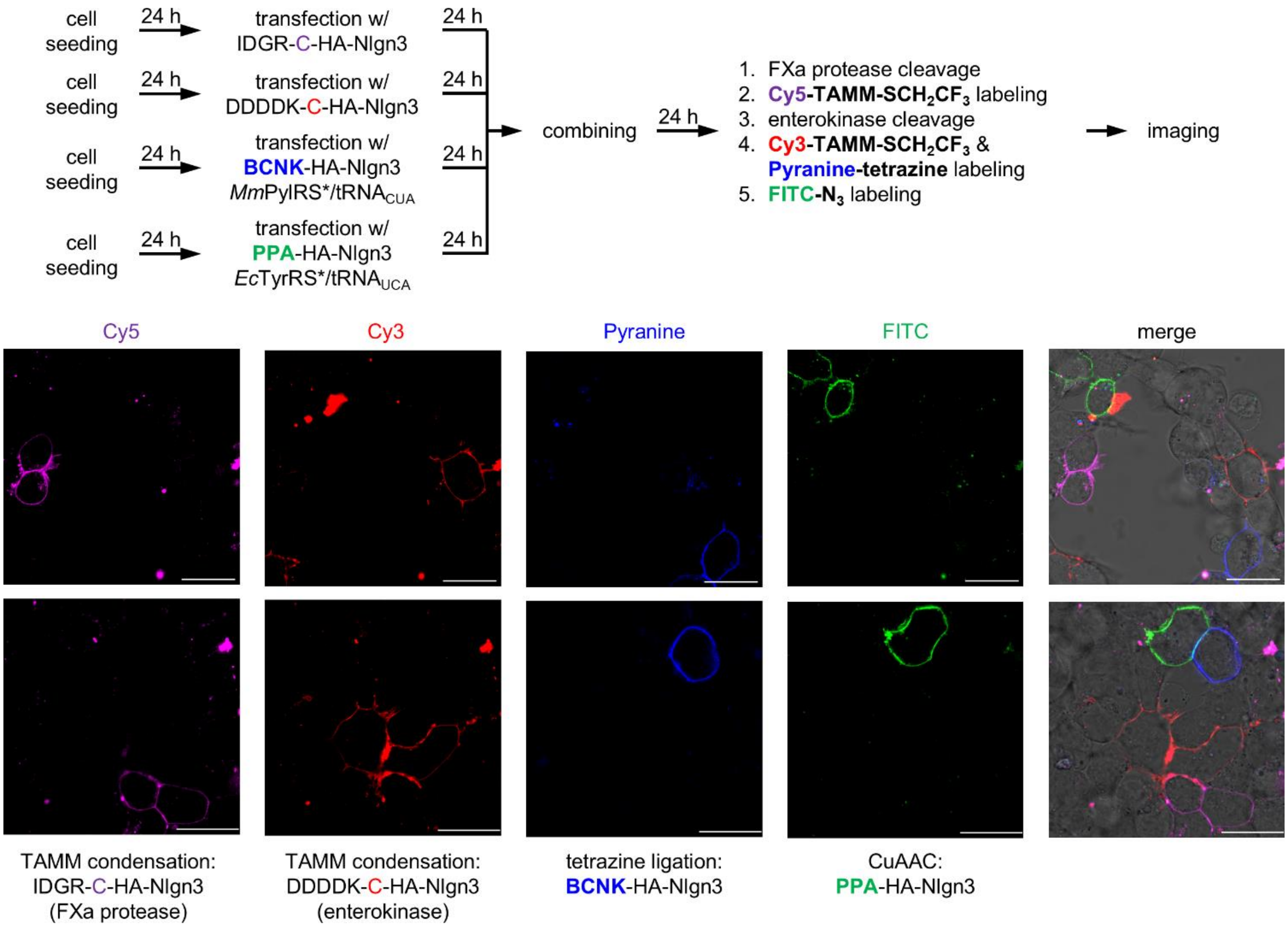

Fig. S34. Four-color fluorescence labeling.

Using two sequence specific proteases for iterative TAMM condensation in combination with tetrazine ligation and CuAAC for labeling four different target proteins on HEK293T cells. See also **Fig. 4E**. Schematic presentation of the experimental flow is shown at the top. Representative confocal microscopy images are shown at the bottom. Scale bar = 20 µm.

Supplementary Tables

Table S1. Initial rates of **1a** and **2x**

Initial rates of reactions involving TAMM **1a** and peptide **2x** as a function of concentration at pH 7.4 and 25 °C.

[**1a**]$_0$ (μM)	[**2x**]$_{tot,0}$ (μM)	initial rate (μM s^{-1})
69.8	48.9	(7.08±0.12) × 10^{-3}
99.3	48.9	(1.05±0.02) × 10^{-2}
147.9	48.9	(1.52±0.01) × 10^{-2}
99.1	29.0	(6.60±0.63) × 10^{-3}
99.1	51.0	(1.22±0.05) × 10^{-2}
99.1	78.8	(1.76±0.06) × 10^{-2}

Table S2. Rate constants at different temperatures.

Rate constants for reactions involving **1a** and **2x** at pH 7.4 at specified temperatures.

**Temperature (K)**	***k* (M^{-1} s^{-1})**
278.15	5.84 ± 0.01
288.15	15.48 ± 0.21
298.15	35.98 ± 1.57
303.15	42.43 ± 1.68
313.15	86.69 ± 0.96

Table S3. Rate constants for **Int-1** to **3**

Rate constants for the conversion of **Int-1** → **3** at pH 7.4 at specified temperatures.

**Temperature (K)**	***k* (s^{-1})**
278.15	7.21E-05 ± 7.78E-07
288.15	0.272E-03 ± 1.55E-05
298.15	0.813E-03 ± 8.63E-05
303.15	1.65E-03 ± 5.66E-05
313.15	2.38E-03 ± 11.31E-05

NMR Spectra

Synthesis of **1a**

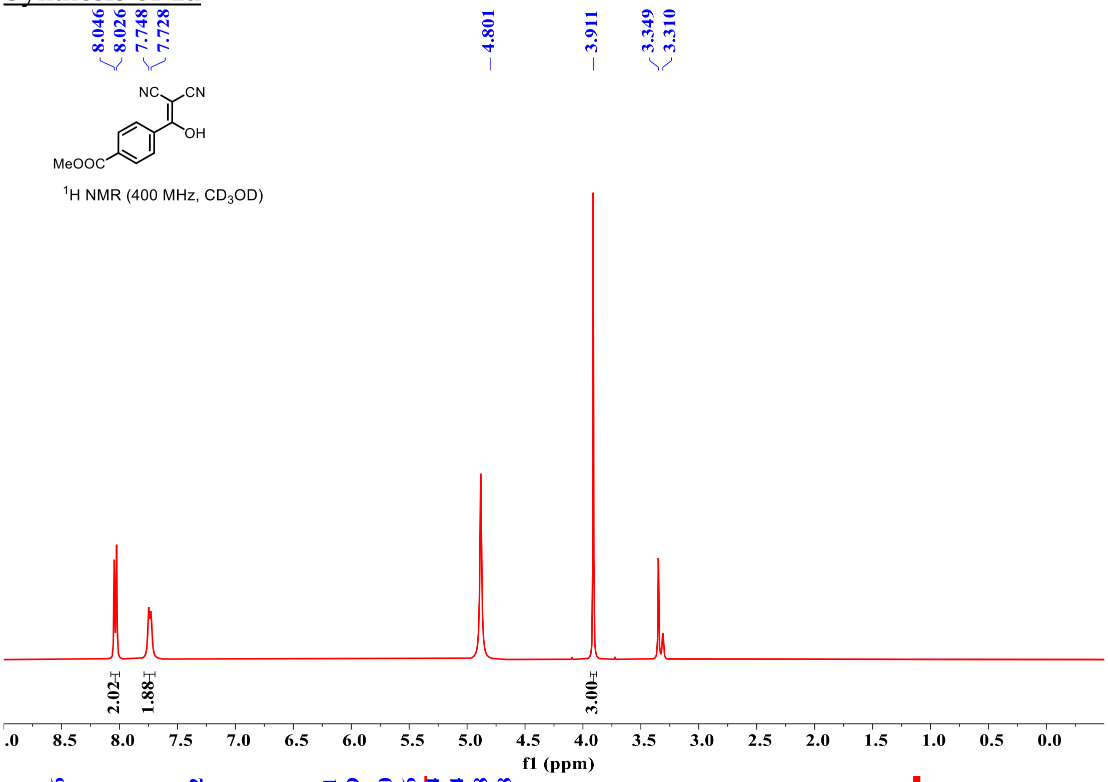

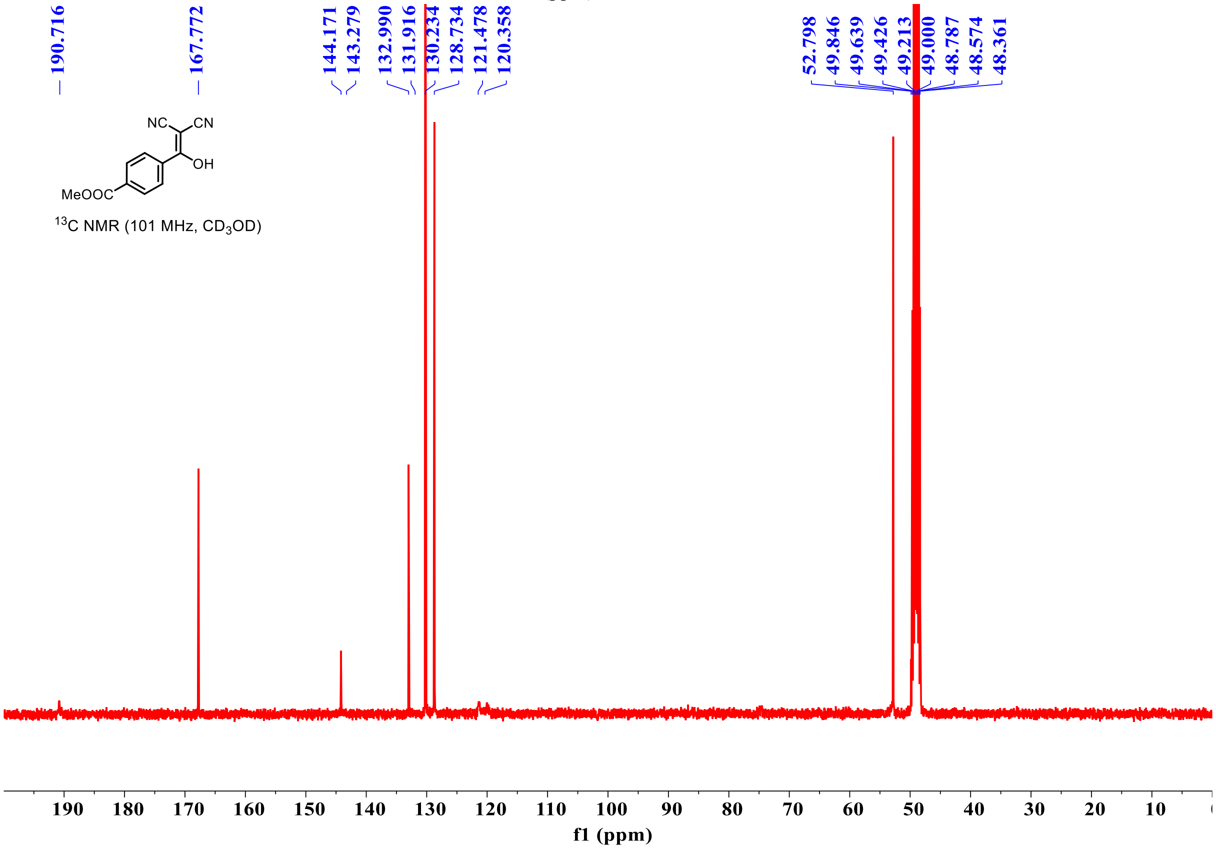

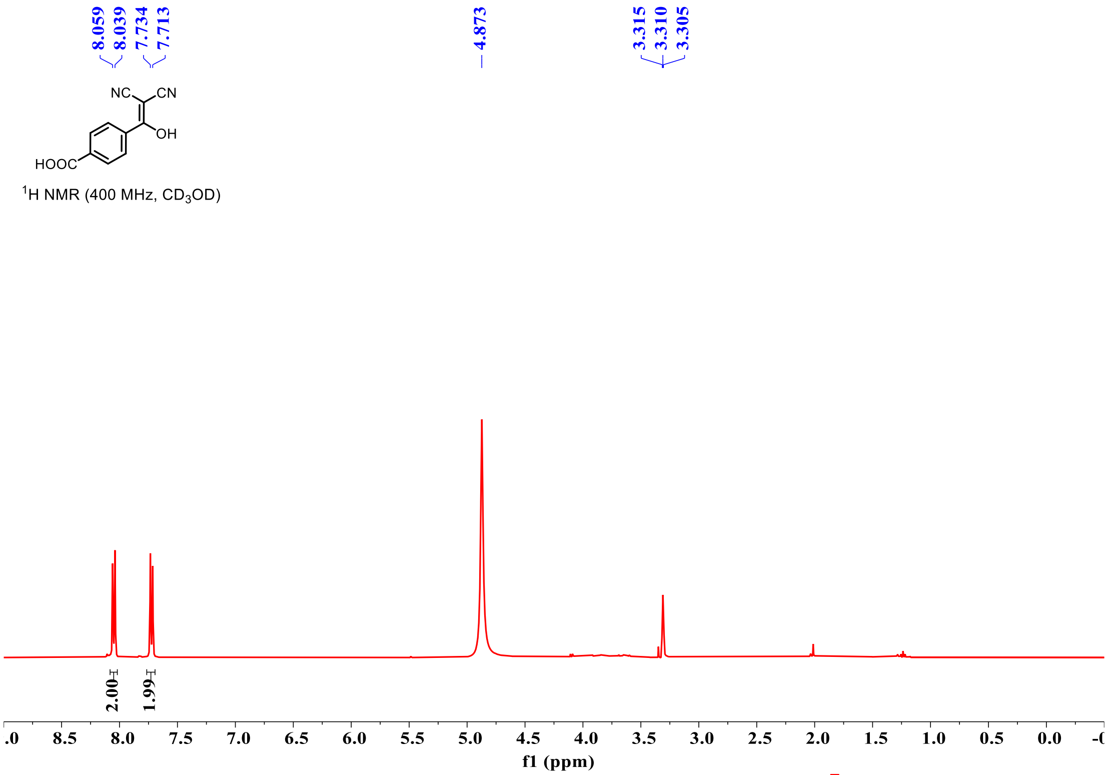
8.059
8.039
7.734
7.713
4.873
3.315
3.310
3.305
NC
CN
OH
HOOC
1H NMR (400 MHz, CD3OD)
2.00
1.99
f1 (ppm)

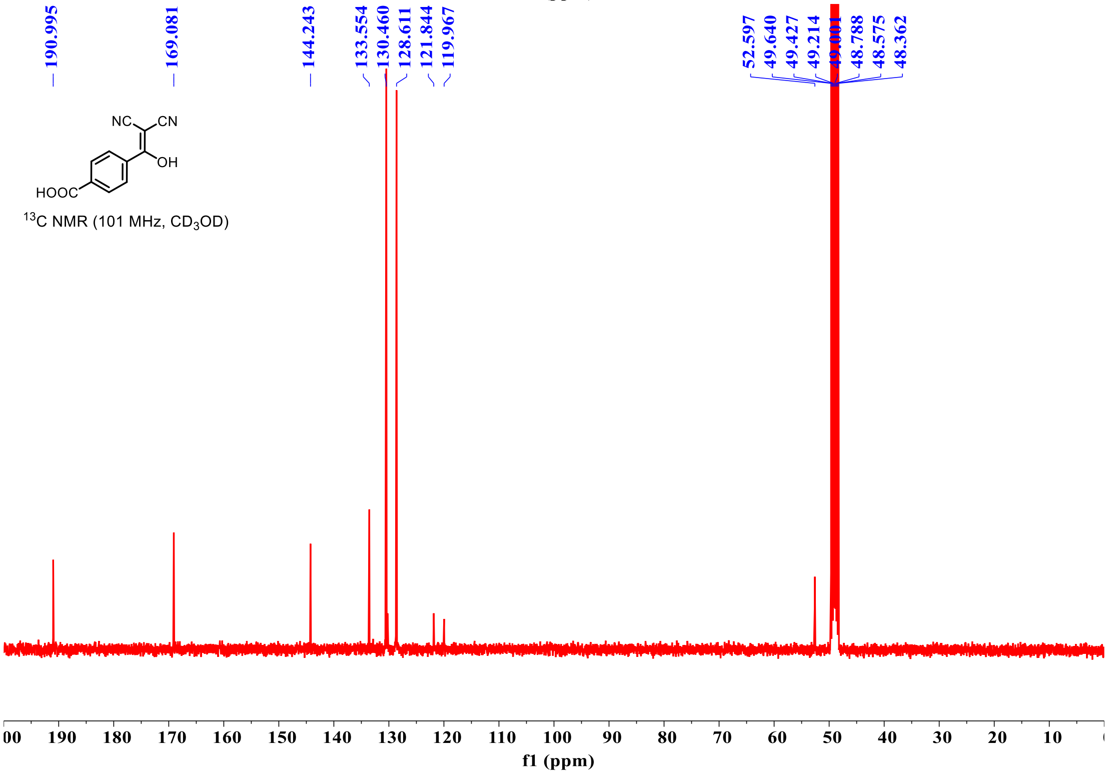
190.995
169.081
144.243
133.554
130.460
128.611
121.844
119.967
52.597
49.640
49.427
49.214
49.001
48.788
48.575
48.362
NC
CN
OH
HOOC
13C NMR (101 MHz, CD3OD)
f1 (ppm)

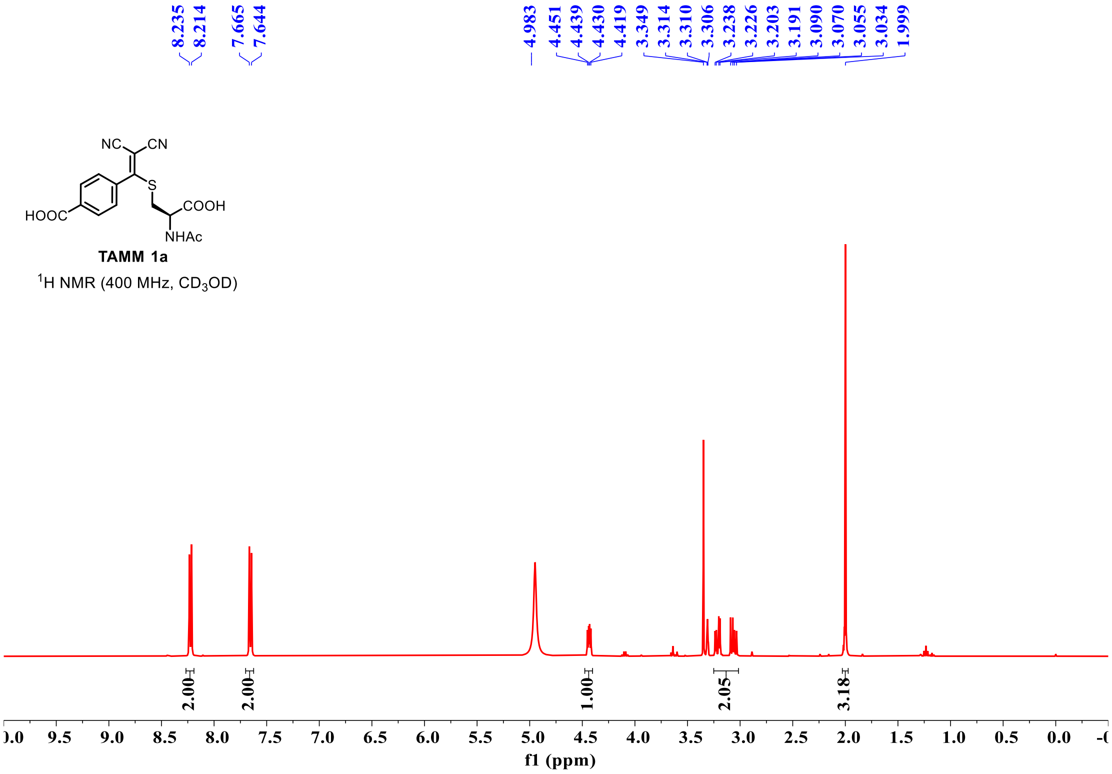
8.235
8.214
7.665
7.644
4.983
4.451
4.439
4.430
4.419
3.349
3.314
3.310
3.306
3.238
3.226
3.203
3.191
3.090
3.070
3.055
3.034
1.999
NC
CN
S
HOOC
COOH
NHAc
TAMM 1a
1H NMR (400 MHz, CD3OD)
2.00
2.00
1.00
2.05
3.18
f1 (ppm)

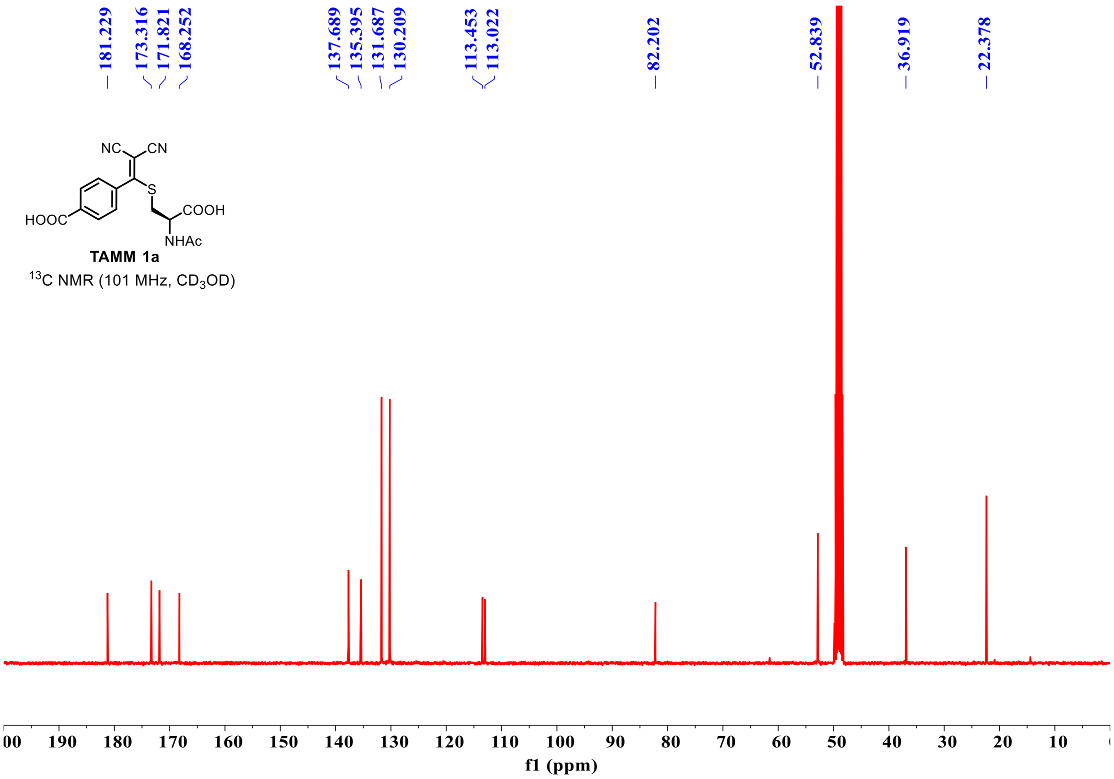
181.229
173.316
171.821
168.252
137.689
135.395
131.687
130.209
113.453
113.022
82.202
52.839
36.919
22.378
NC
CN
S
HOOC
COOH
NHAc
TAMM 1a
13C NMR (101 MHz, CD3OD)
f1 (ppm)

Synthesis of **1b**

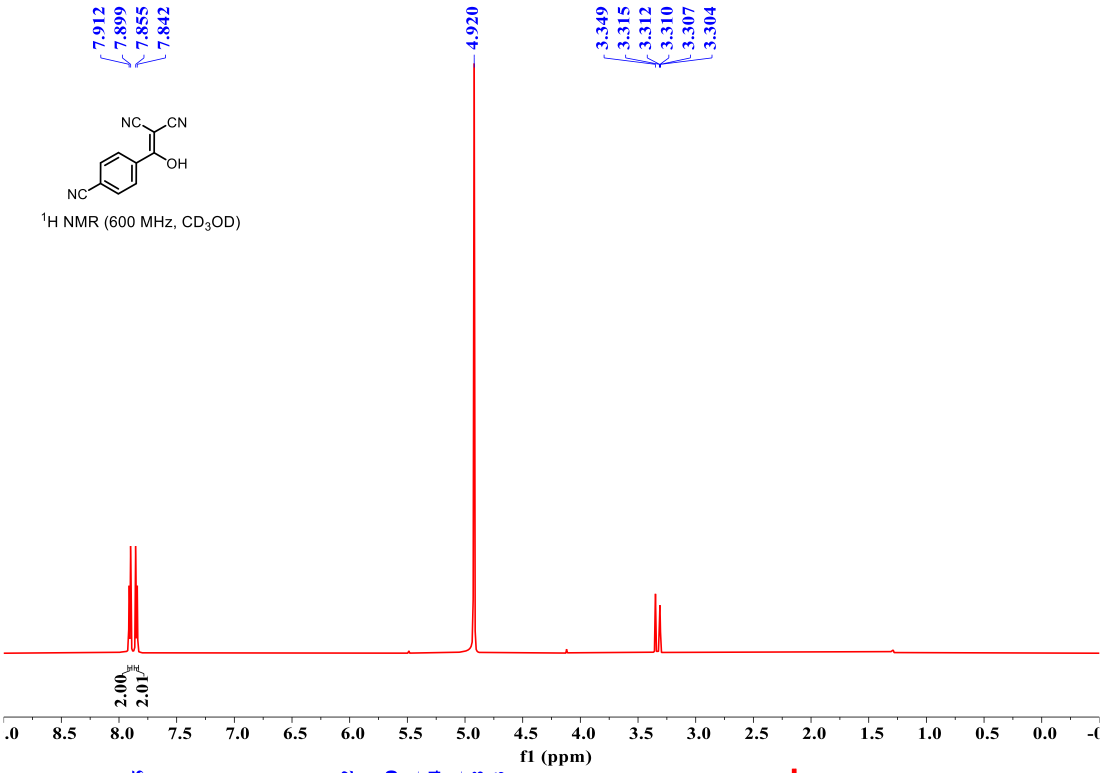

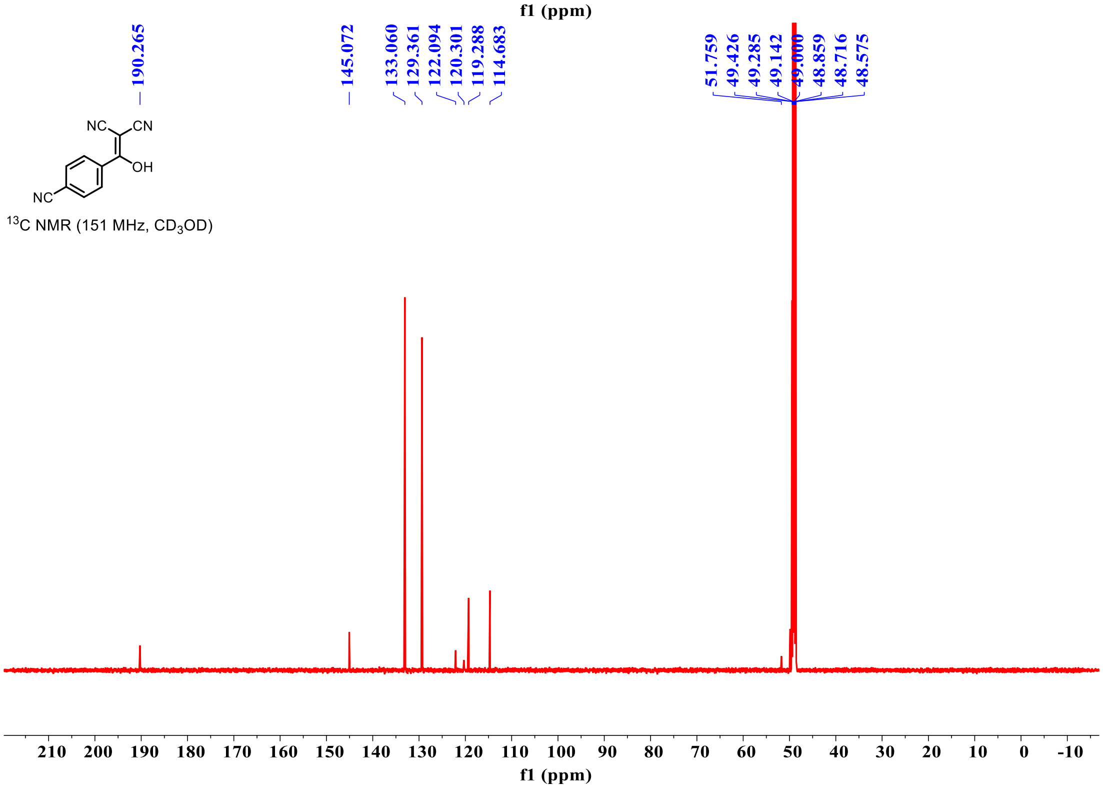

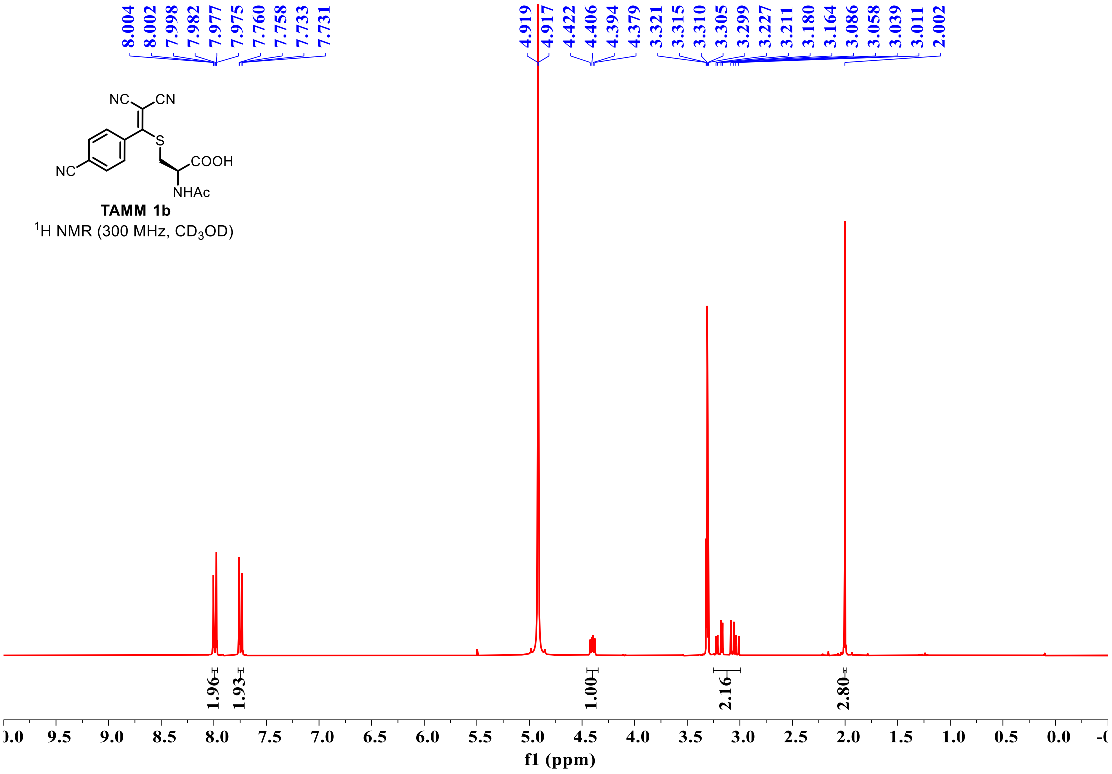
8.004 8.002 7.998 7.982 7.977 7.975 7.760 7.758 7.733 7.731
4.919 4.917 4.422 4.406 4.394 4.379 3.321 3.315 3.310 3.305 3.299 3.227 3.211 3.180 3.164 3.086 3.058 3.039 3.011 2.002
NC CN
S
COOH
NC
NHAc
TAMM 1b
1H NMR (300 MHz, CD3OD)
1.96
1.93
1.00
2.16
2.80
f1 (ppm)

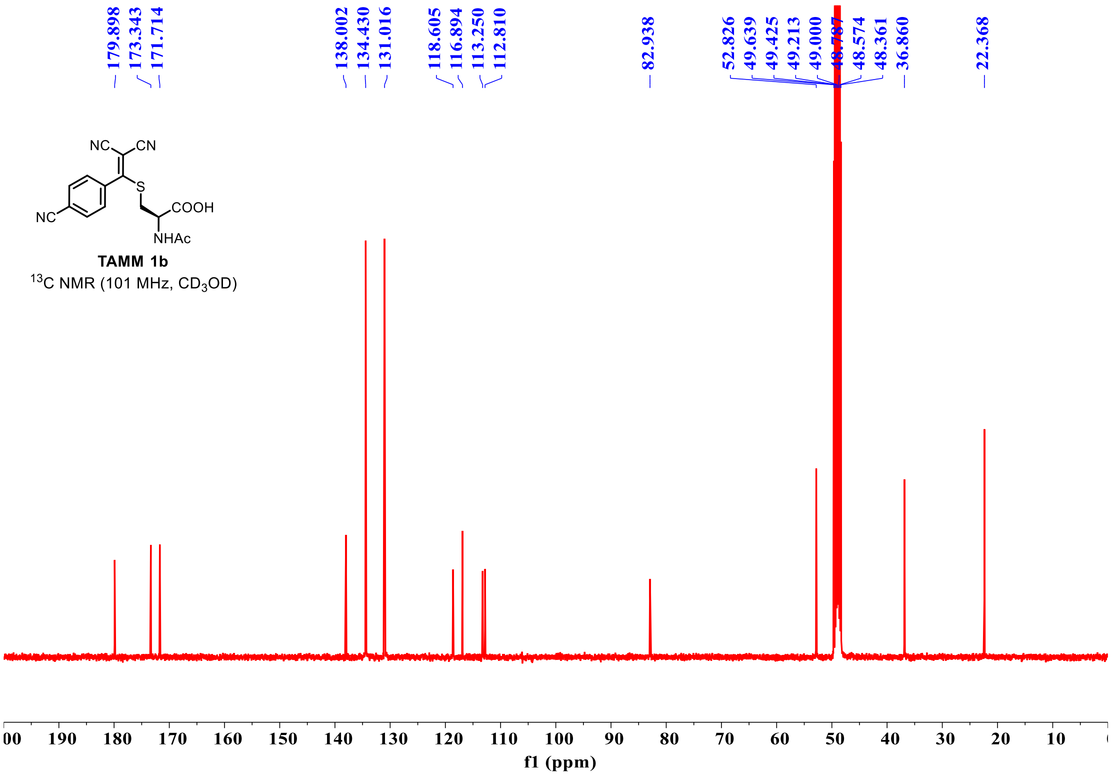
179.898 173.343 171.714
138.002 134.430 131.016
118.605 116.894 113.250 112.810
82.938
52.826 49.639 49.425 49.213 49.000 48.787 48.574 48.361 36.860
22.368
NC CN
S
COOH
NC
NHAc
TAMM 1b
13C NMR (101 MHz, CD3OD)
f1 (ppm)

Synthesis of **1c**

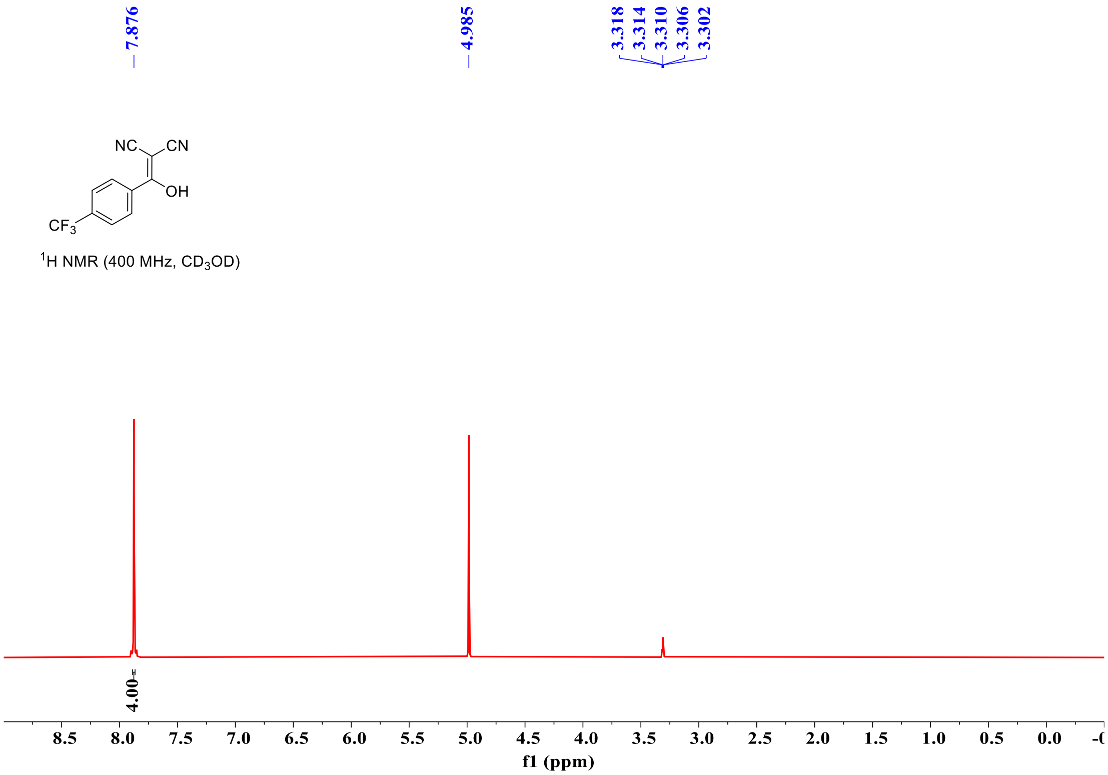

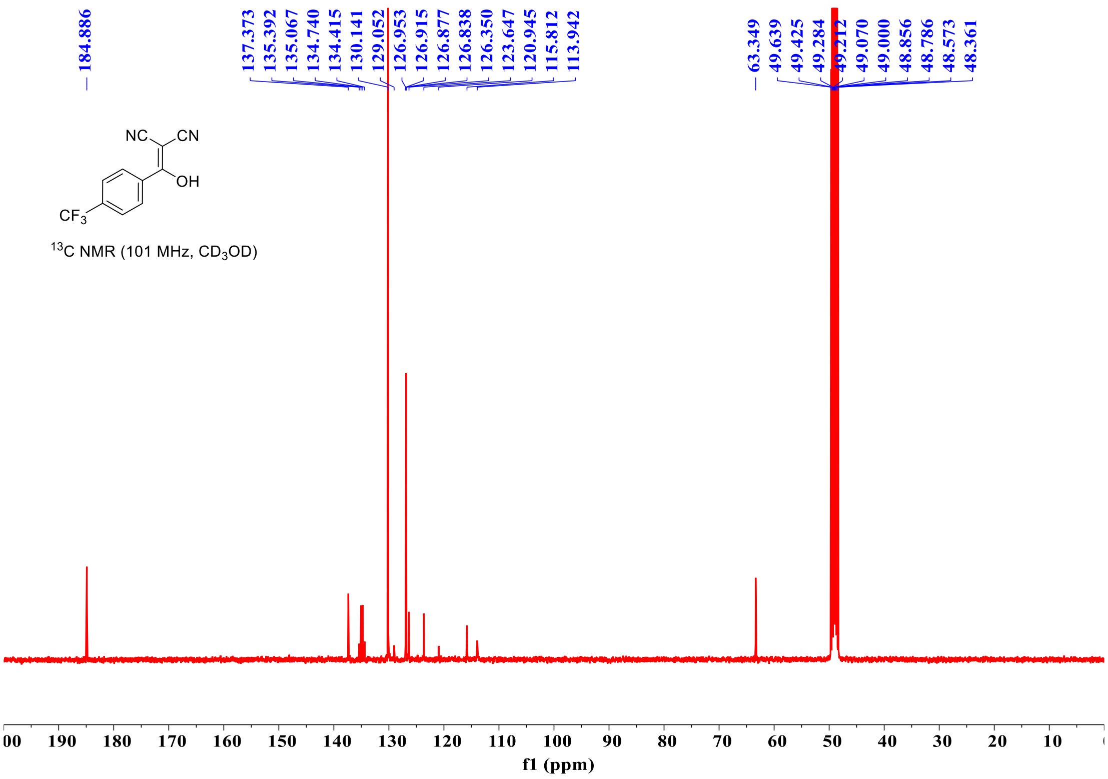

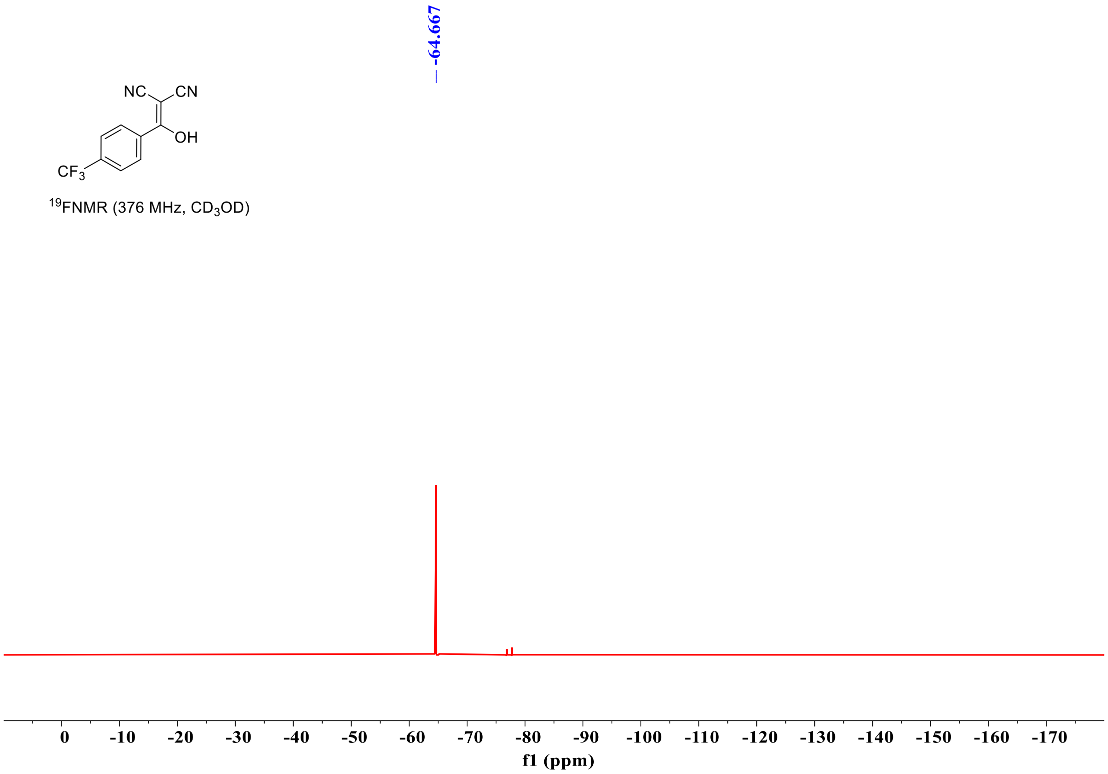

-64.667
NC CN OH CF3
^{19}FNMR (376 MHz, CD$_3$OD)
0 -10 -20 -30 -40 -50 -60 -70 -80 -90 -100 -110 -120 -130 -140 -150 -160 -170
f1 (ppm)

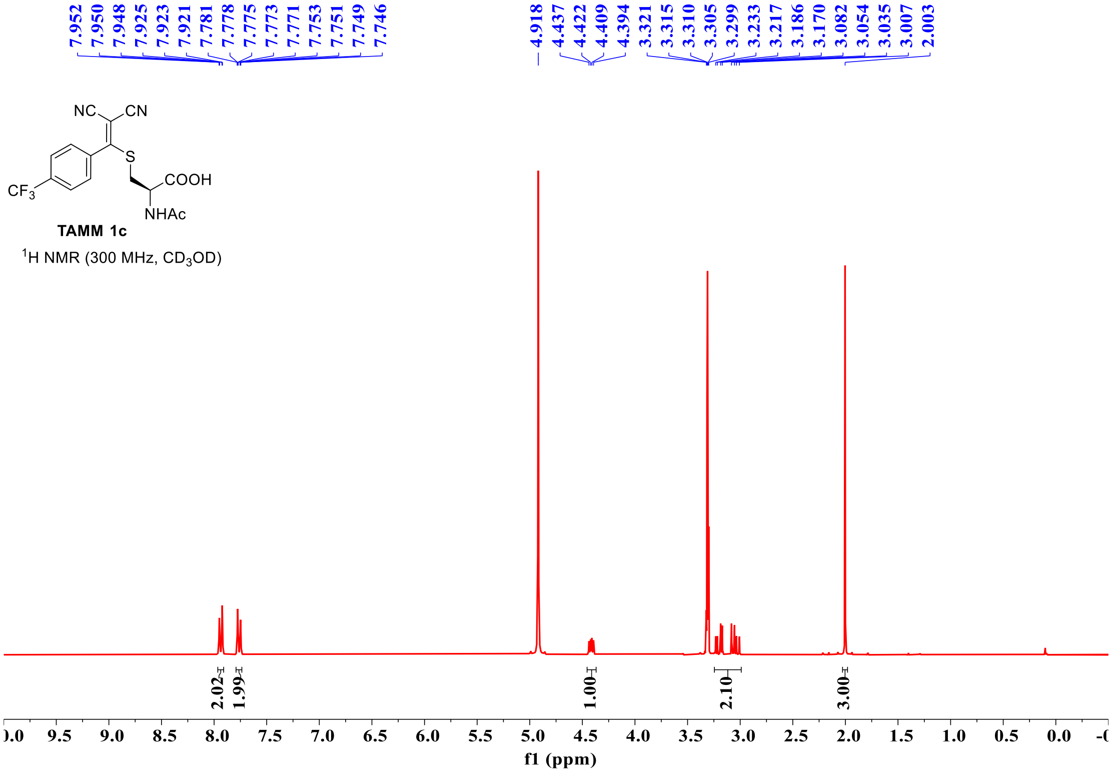

7.952 7.950 7.948 7.925 7.923 7.921 7.781 7.778 7.775 7.773 7.771 7.753 7.751 7.749 7.746
4.918 4.437 4.422 4.409 4.394 3.321 3.315 3.310 3.305 3.299 3.233 3.217 3.186 3.170 3.082 3.054 3.035 3.007 2.003
NC CN S COOH NHAc CF3
TAMM 1c
^{1}H NMR (300 MHz, CD$_3$OD)
2.02 1.99 1.00 2.10 3.00
9.5 9.0 8.5 8.0 7.5 7.0 6.5 6.0 5.5 5.0 4.5 4.0 3.5 3.0 2.5 2.0 1.5 1.0 0.5 0.0
f1 (ppm)

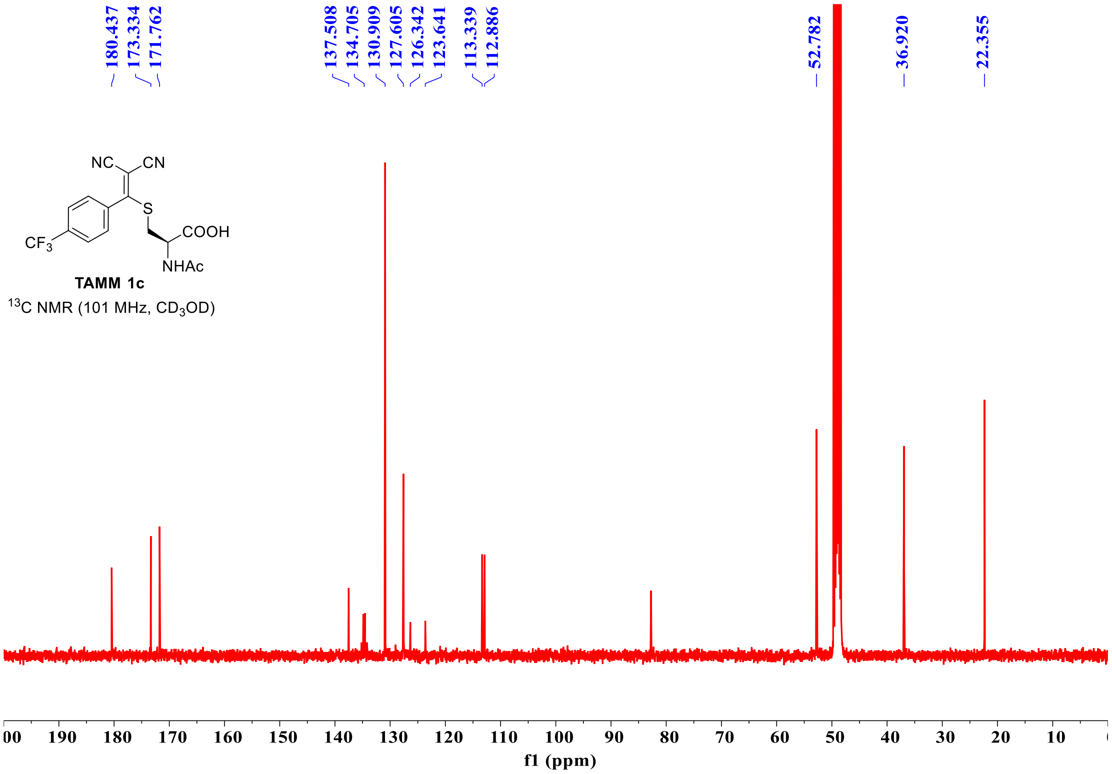
180.437
173.334
171.762
137.508
134.705
130.909
127.605
126.342
123.641
113.339
112.886
52.782
36.920
22.355
NC
CN
S
CF3
COOH
NHAc
TAMM 1c
13C NMR (101 MHz, CD3OD)
f1 (ppm)

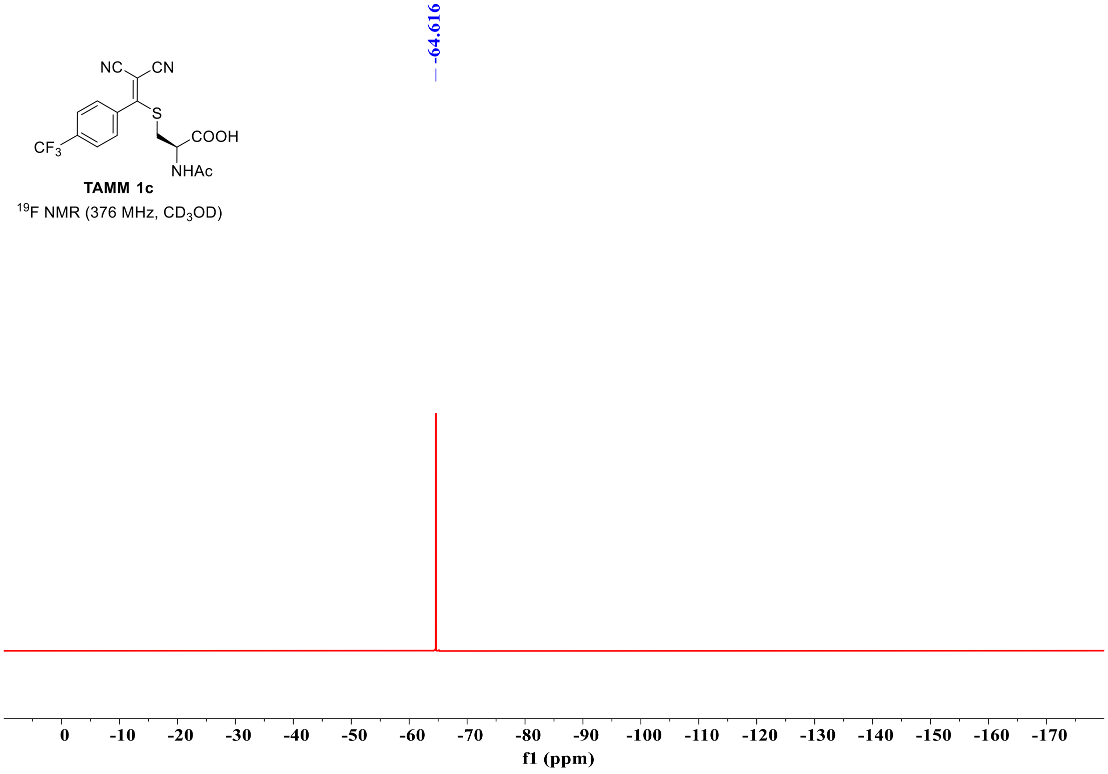
-64.616
NC
CN
S
CF3
COOH
NHAc
TAMM 1c
19F NMR (376 MHz, CD3OD)
f1 (ppm)

Synthesis of **1d**

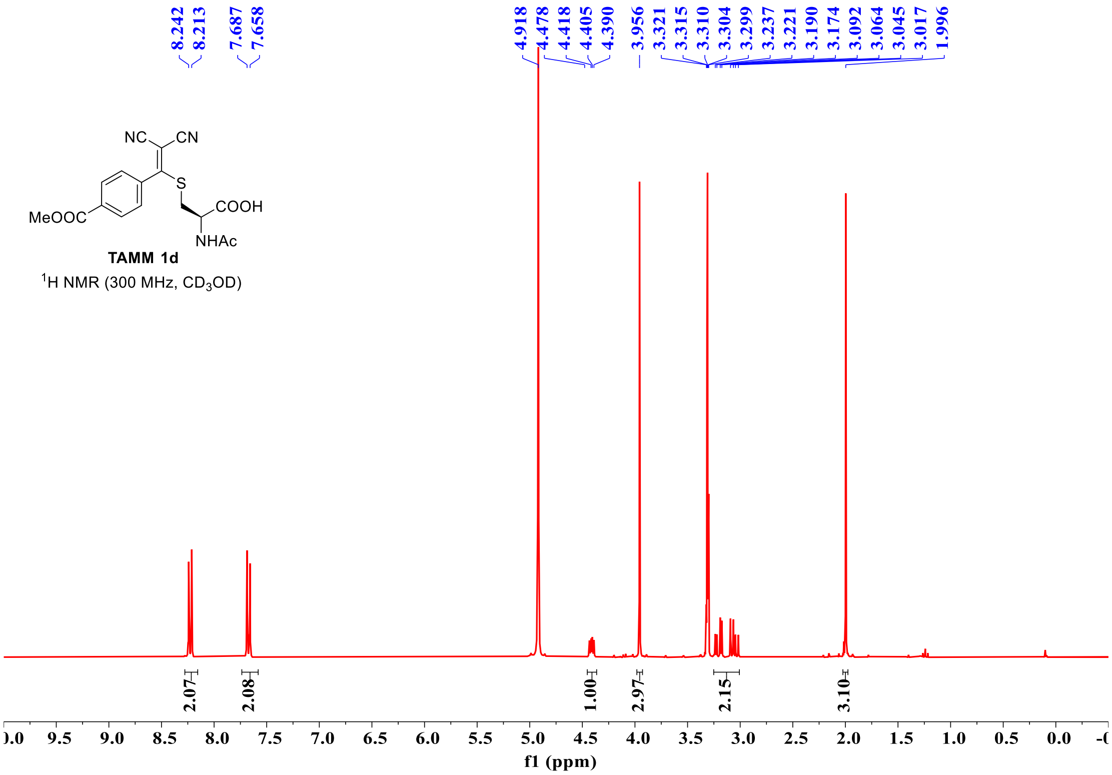

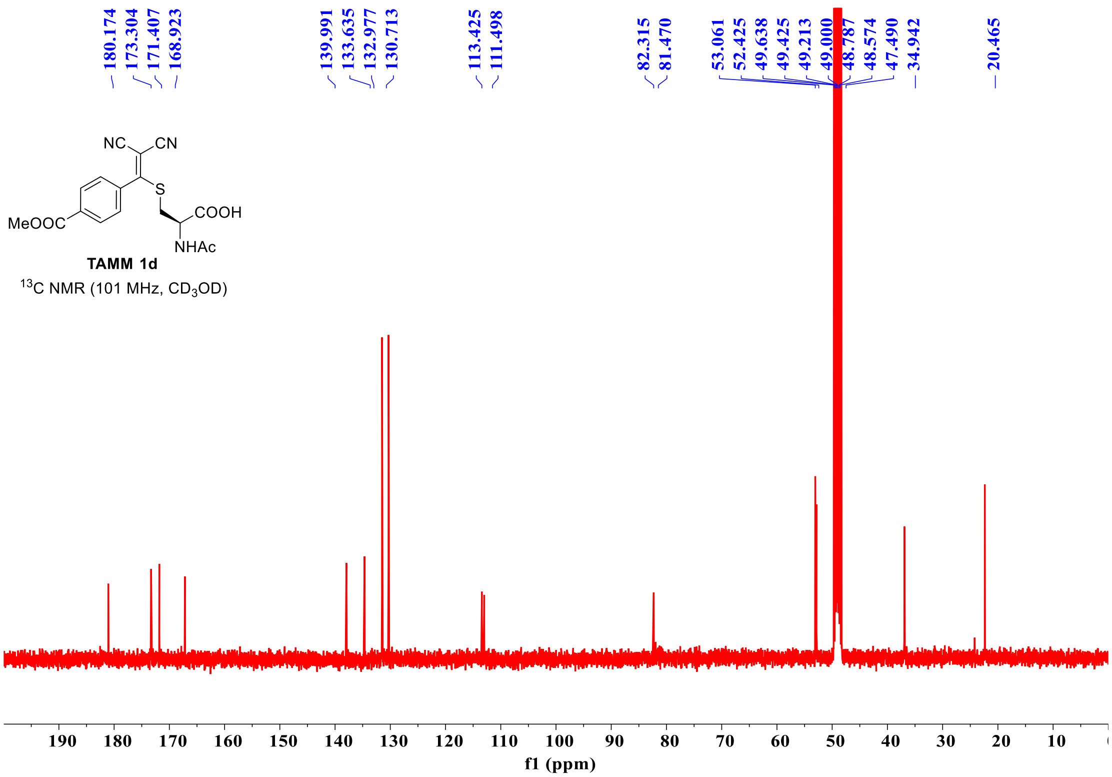

Synthesis of **1e**

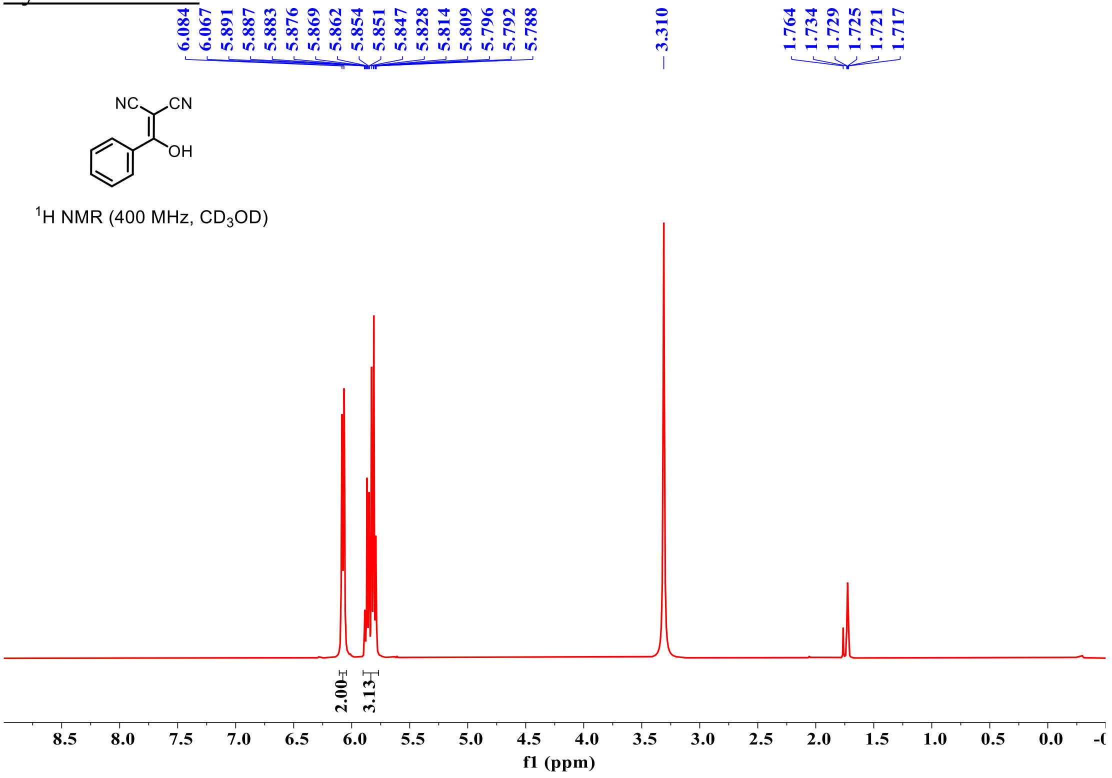

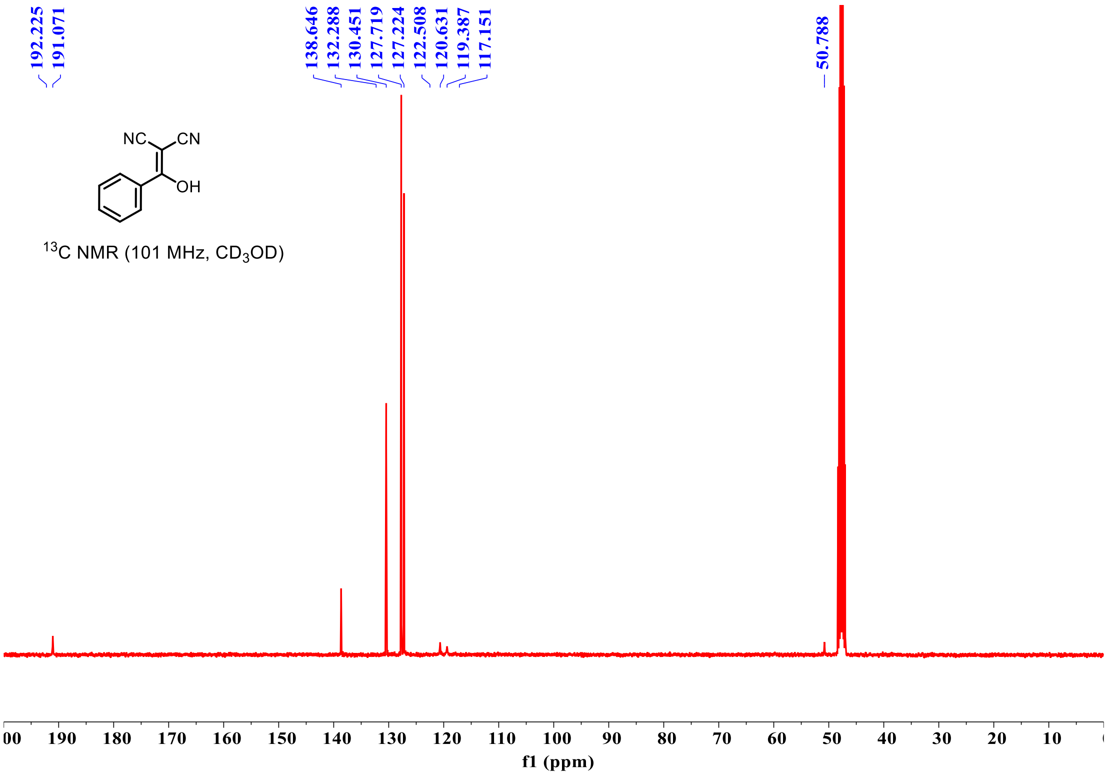

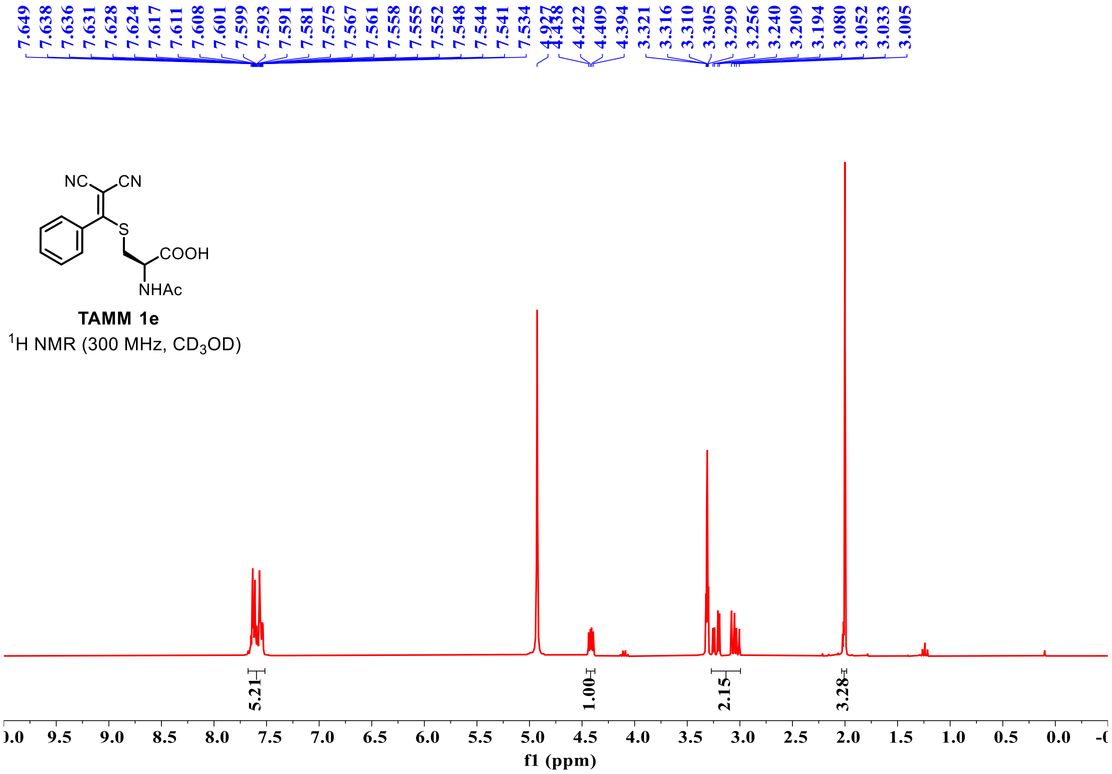

NC
CN
S
COOH
NHAc
TAMM 1e
1H NMR (300 MHz, CD3OD)
5.21
1.00
2.15
3.28
f1 (ppm)

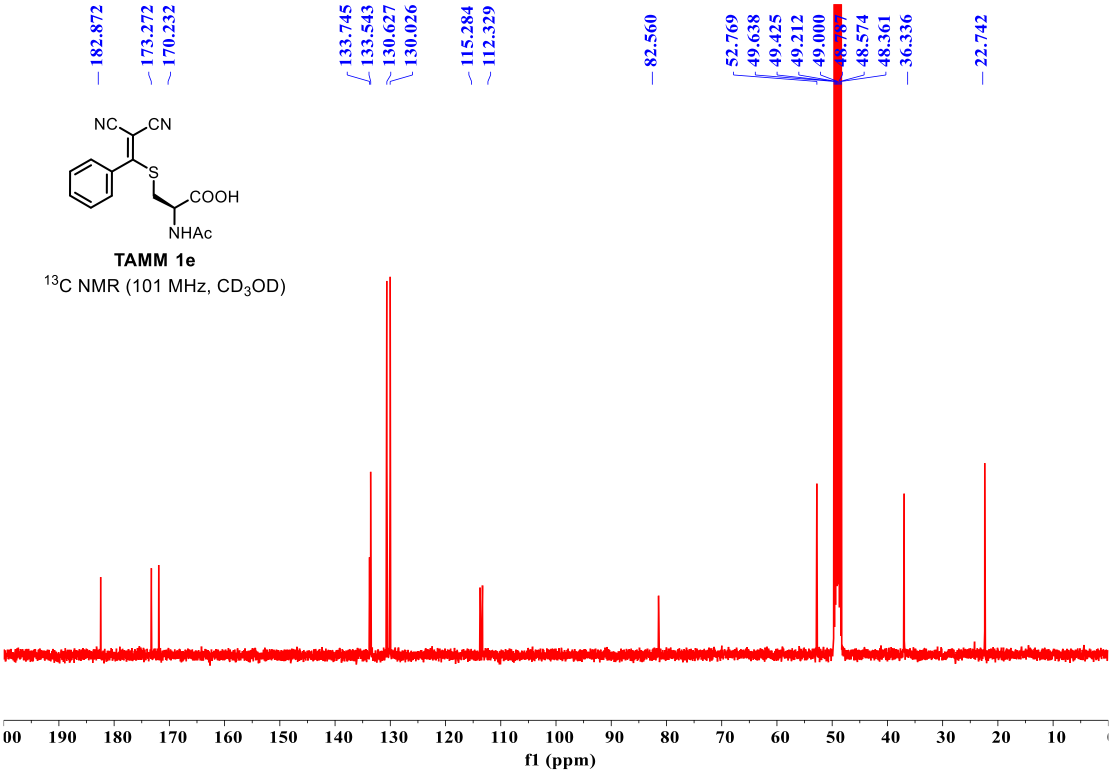

182.872
173.272
170.232
133.745
133.543
130.627
130.026
115.284
112.329
82.560
52.769
49.638
49.425
49.212
49.000
48.574
48.361
36.336
22.742
NC
CN
S
COOH
NHAc
TAMM 1e
13C NMR (101 MHz, CD3OD)
f1 (ppm)

Synthesis of **1f**

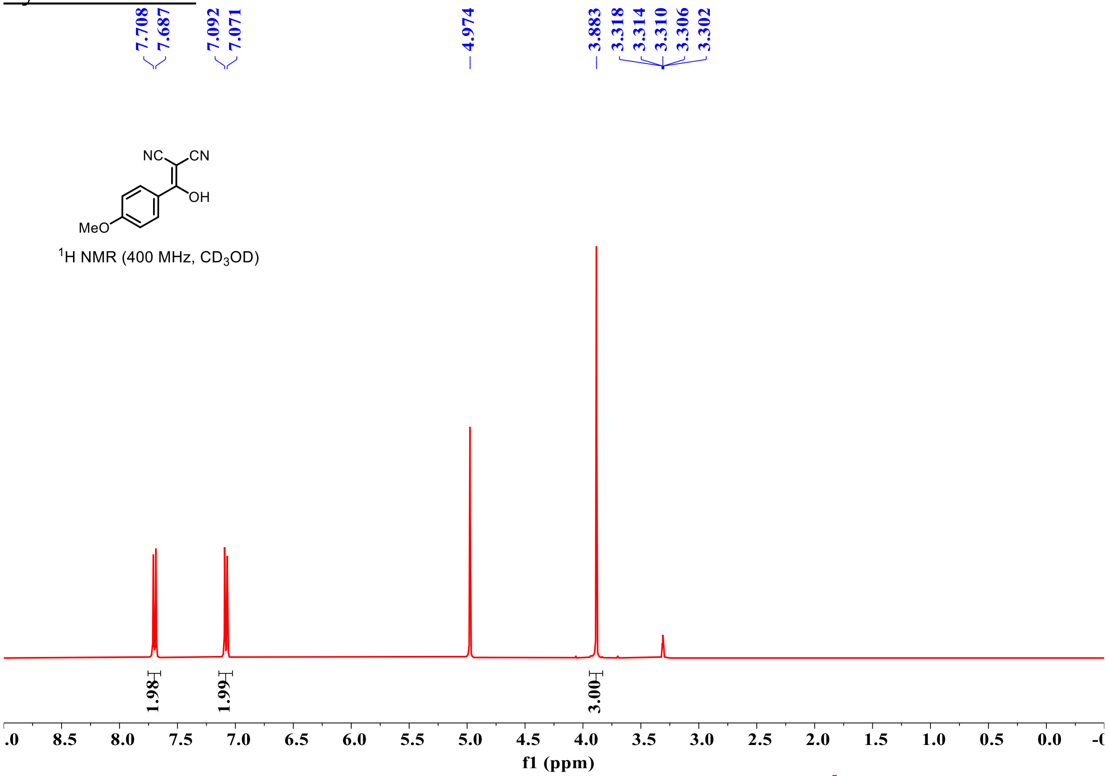

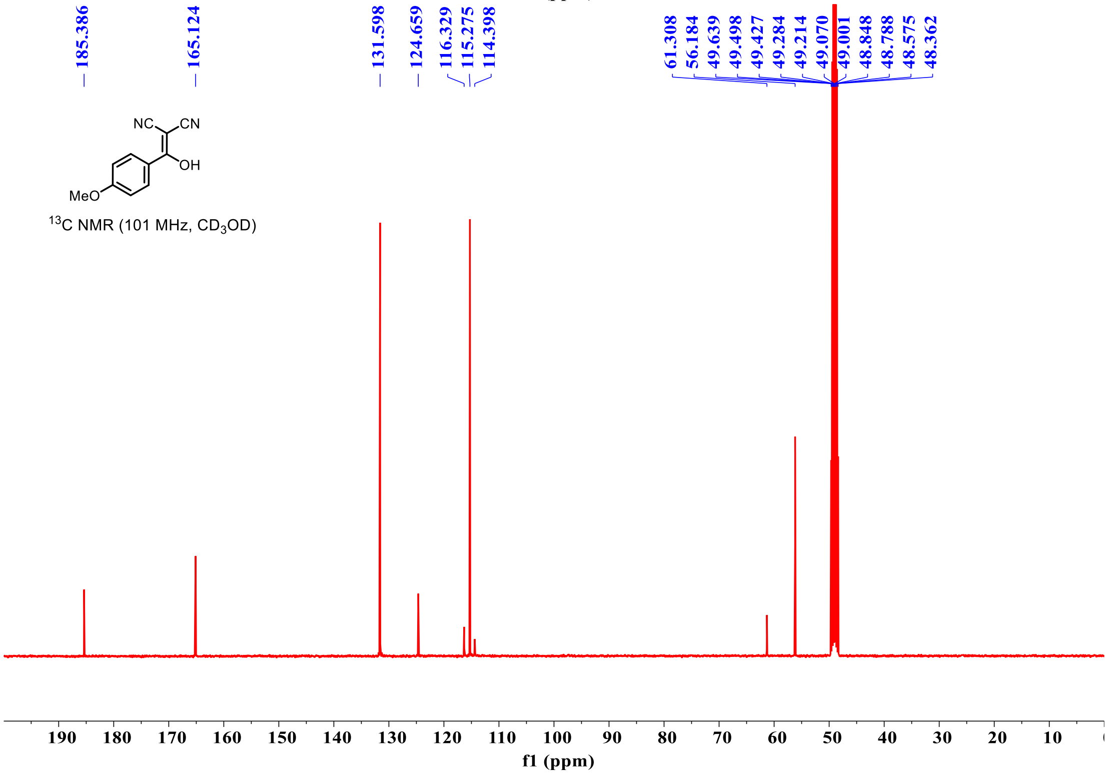

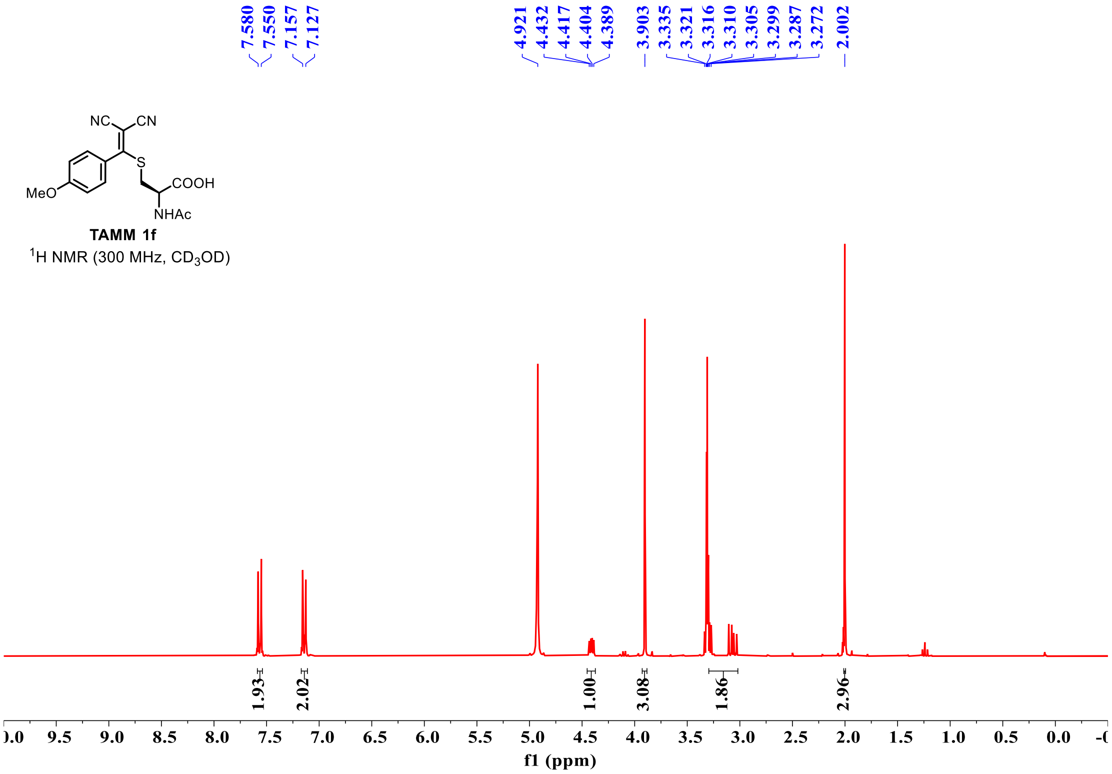
7.580
7.550
7.157
7.127
4.921
4.432
4.417
4.404
4.389
3.903
3.335
3.321
3.316
3.310
3.305
3.299
3.287
3.272
2.002
NC CN
S
COOH
MeO
NHAc
TAMM 1f
1H NMR (300 MHz, CD3OD)
1.93
2.02
1.00
3.08
1.86
2.96
9.5 9.0 8.5 8.0 7.5 7.0 6.5 6.0 5.5 5.0 4.5 4.0 3.5 3.0 2.5 2.0 1.5 1.0 0.5 0.0
f1 (ppm)

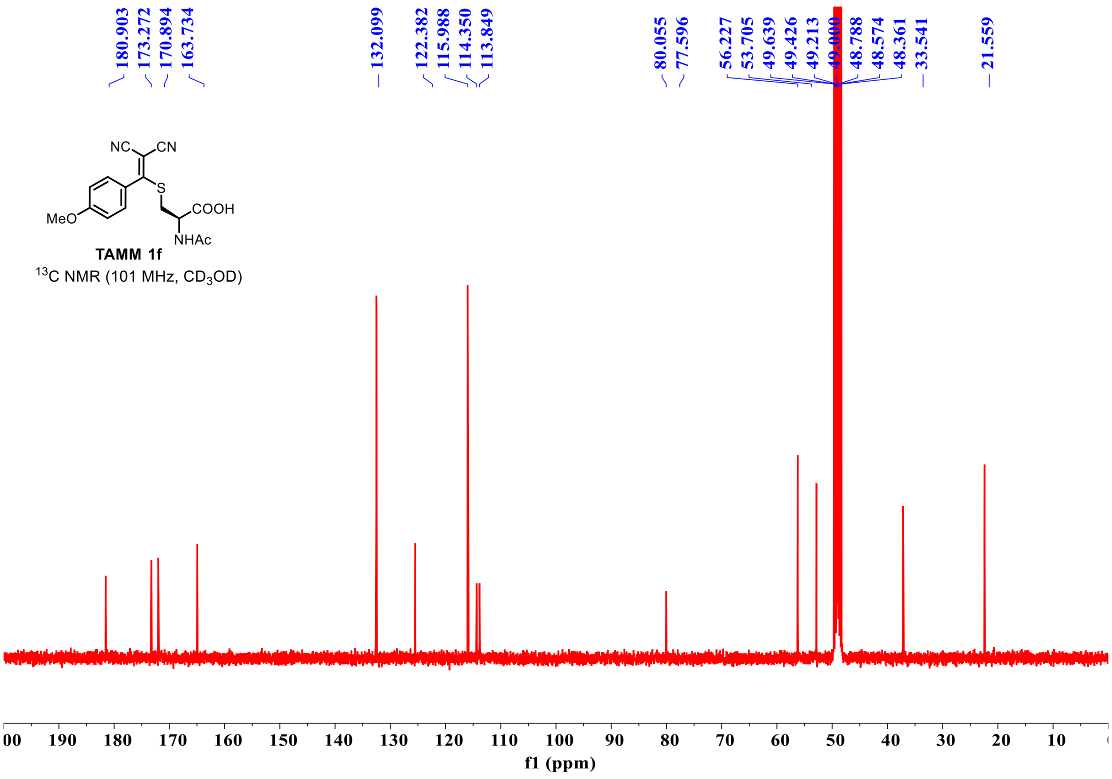
180.903
173.272
170.894
163.734
132.099
122.382
115.988
114.350
113.849
80.055
77.596
56.227
53.705
49.639
49.426
49.213
49.000
48.788
48.574
48.361
33.541
21.559
NC CN
S
COOH
MeO
NHAc
TAMM 1f
13C NMR (101 MHz, CD3OD)
190 180 170 160 150 140 130 120 110 100 90 80 70 60 50 40 30 20 10
f1 (ppm)

Synthesis of **1g**

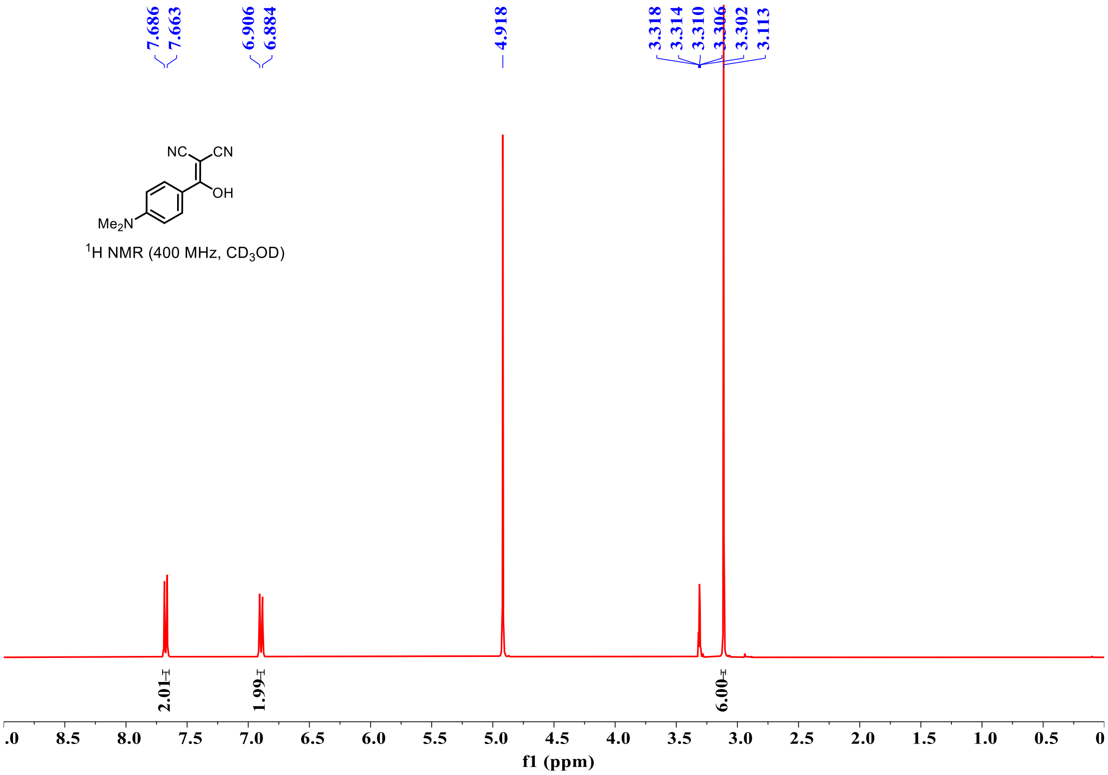

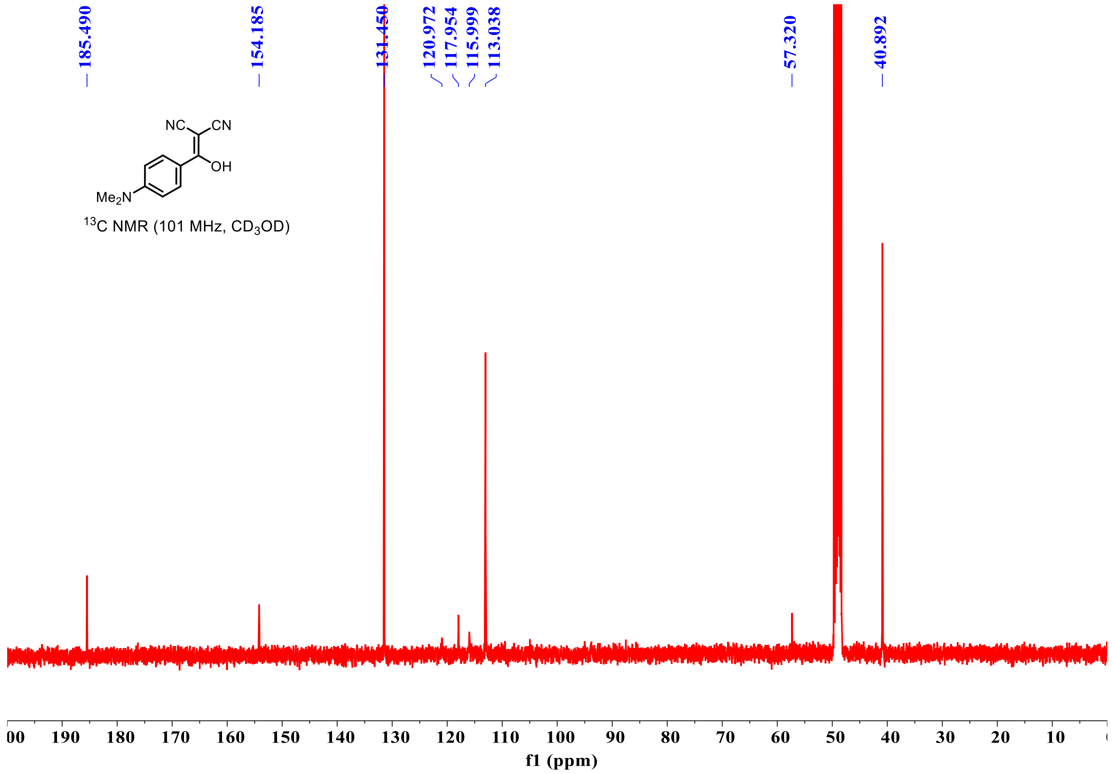

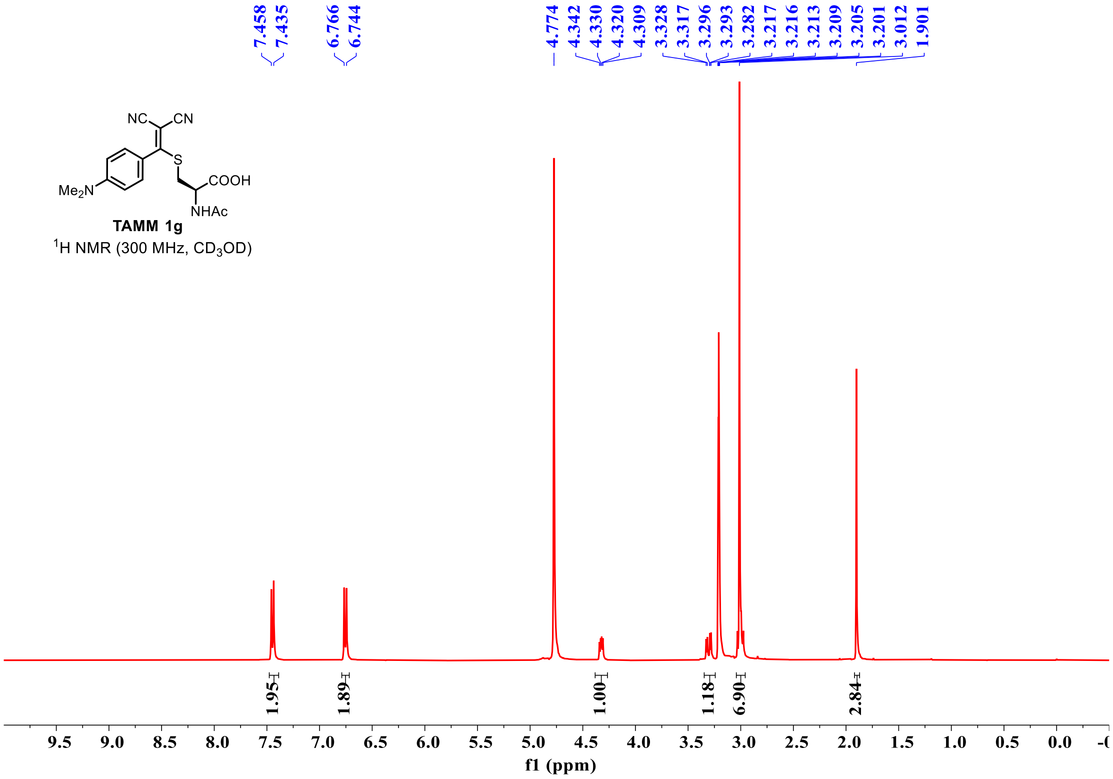

NC
CN
S
Me2N
COOH
NHAc
TAMM 1g
1H NMR (300 MHz, CD3OD)
7.458
7.435
6.766
6.744
4.774
4.342
4.330
4.320
4.309
3.328
3.317
3.296
3.293
3.282
3.217
3.216
3.213
3.209
3.205
3.201
3.012
1.901
1.95
1.89
1.00
1.18
6.90
2.84
9.5
9.0
8.5
8.0
7.5
7.0
6.5
6.0
5.5
5.0
4.5
4.0
3.5
3.0
2.5
2.0
1.5
1.0
0.5
0.0
f1 (ppm)

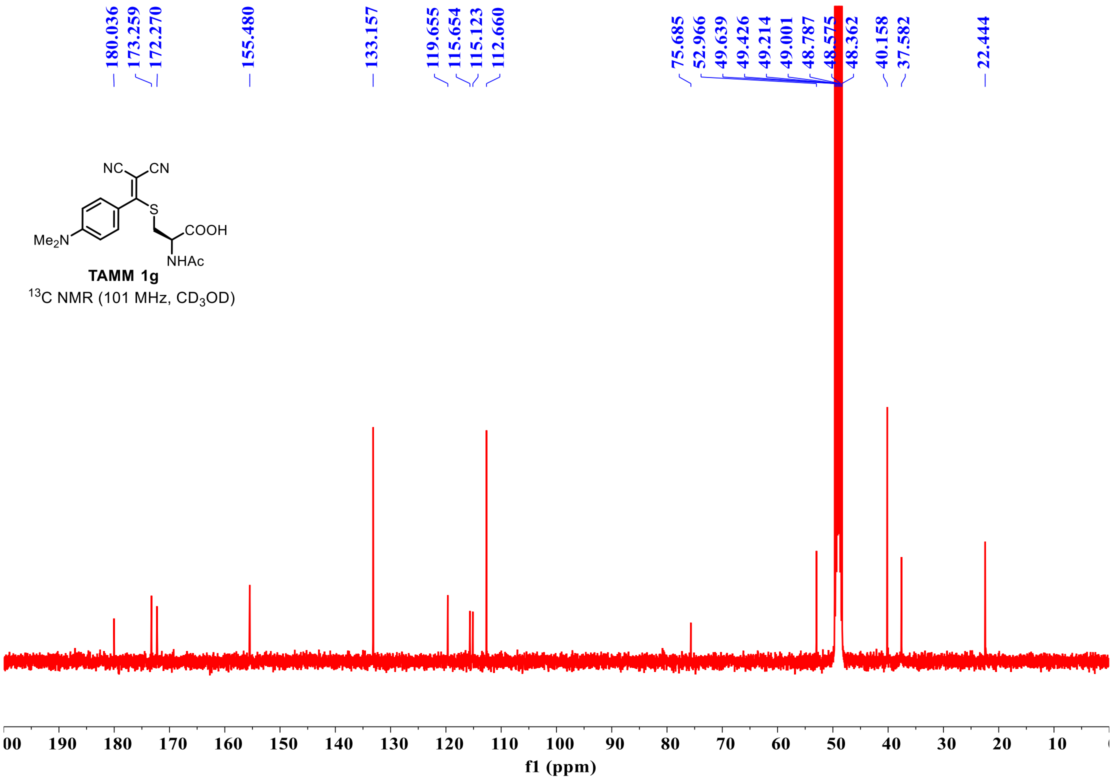

NC
CN
S
Me2N
COOH
NHAc
TAMM 1g
13C NMR (101 MHz, CD3OD)
180.036
173.259
172.270
155.480
133.157
119.655
115.654
115.123
112.660
75.685
52.966
49.639
49.426
49.214
49.001
48.787
48.575
48.362
40.158
37.582
22.444
190
180
170
160
150
140
130
120
110
100
90
80
70
60
50
40
30
20
10
f1 (ppm)

Synthesis of **1h**

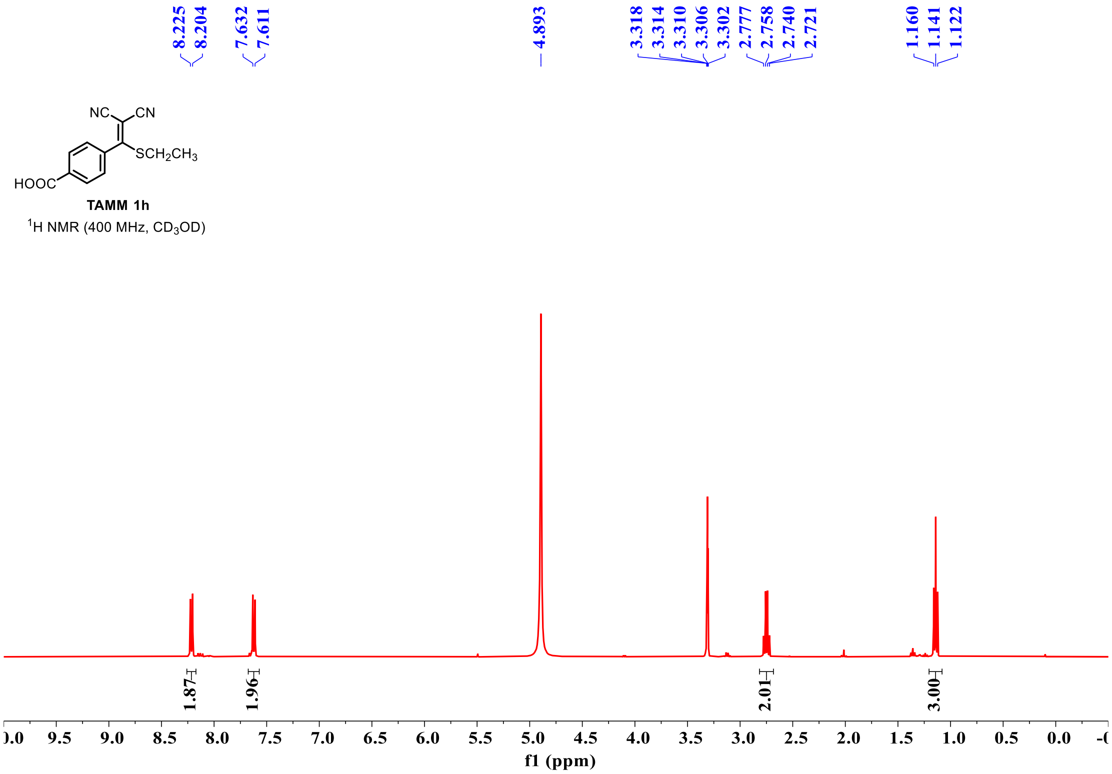

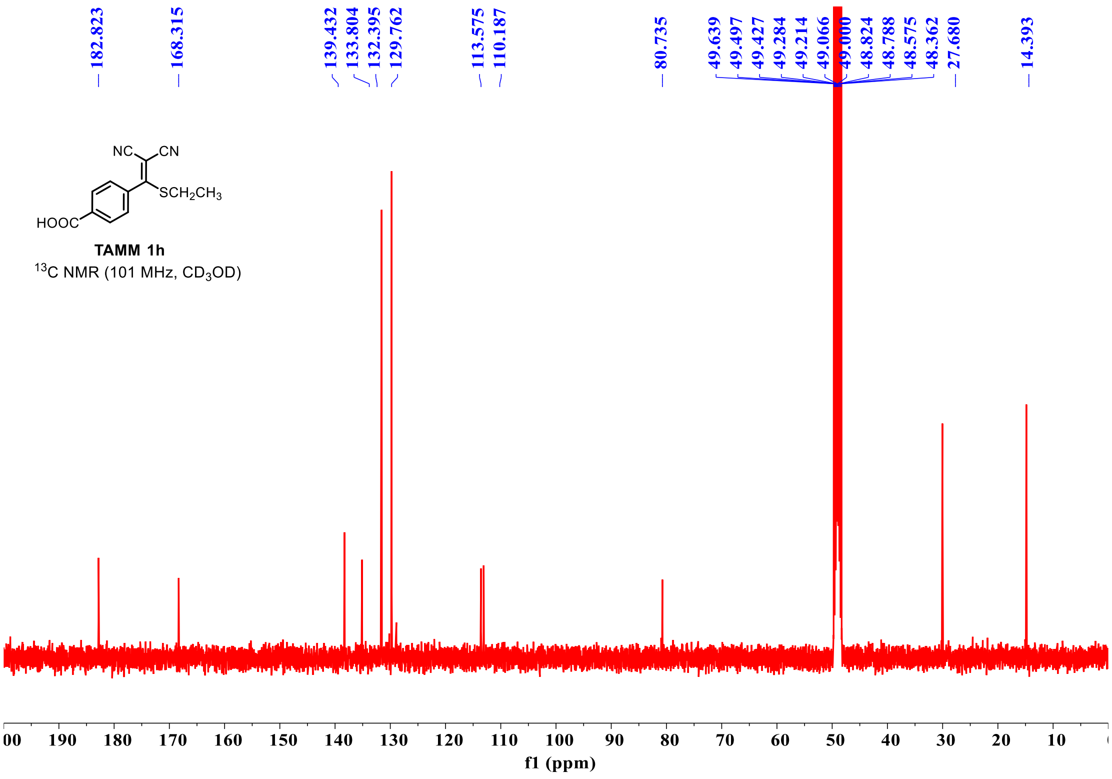

Synthesis of **1i**

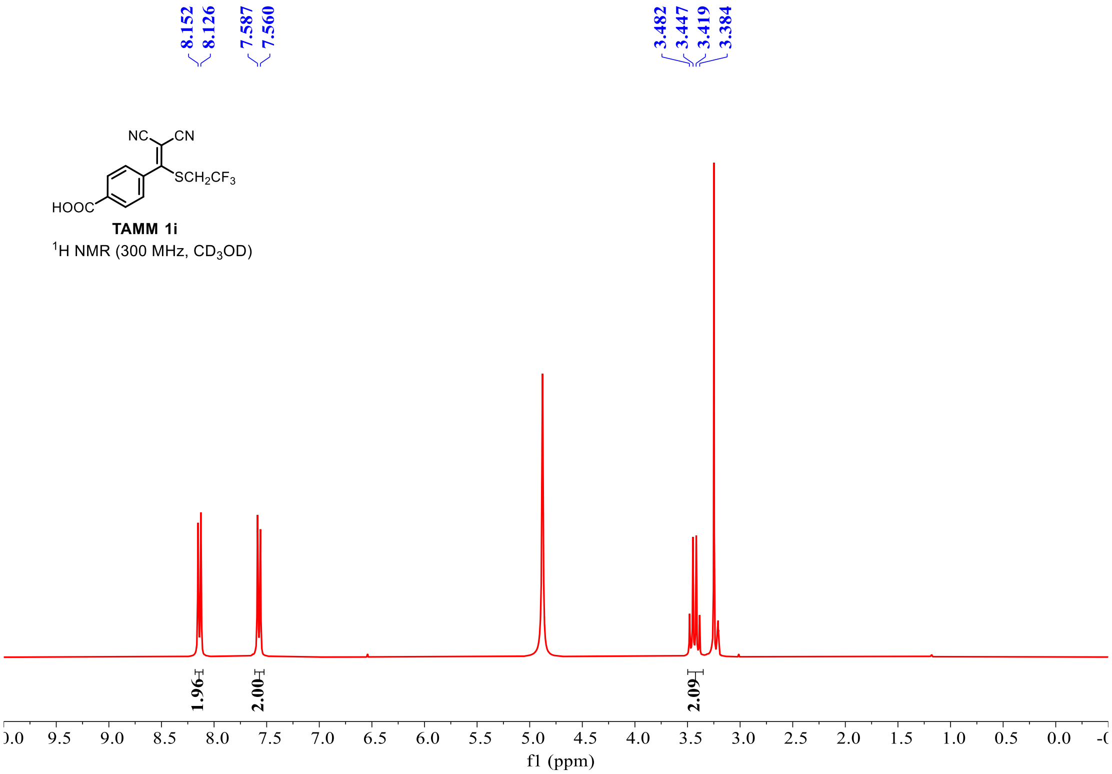

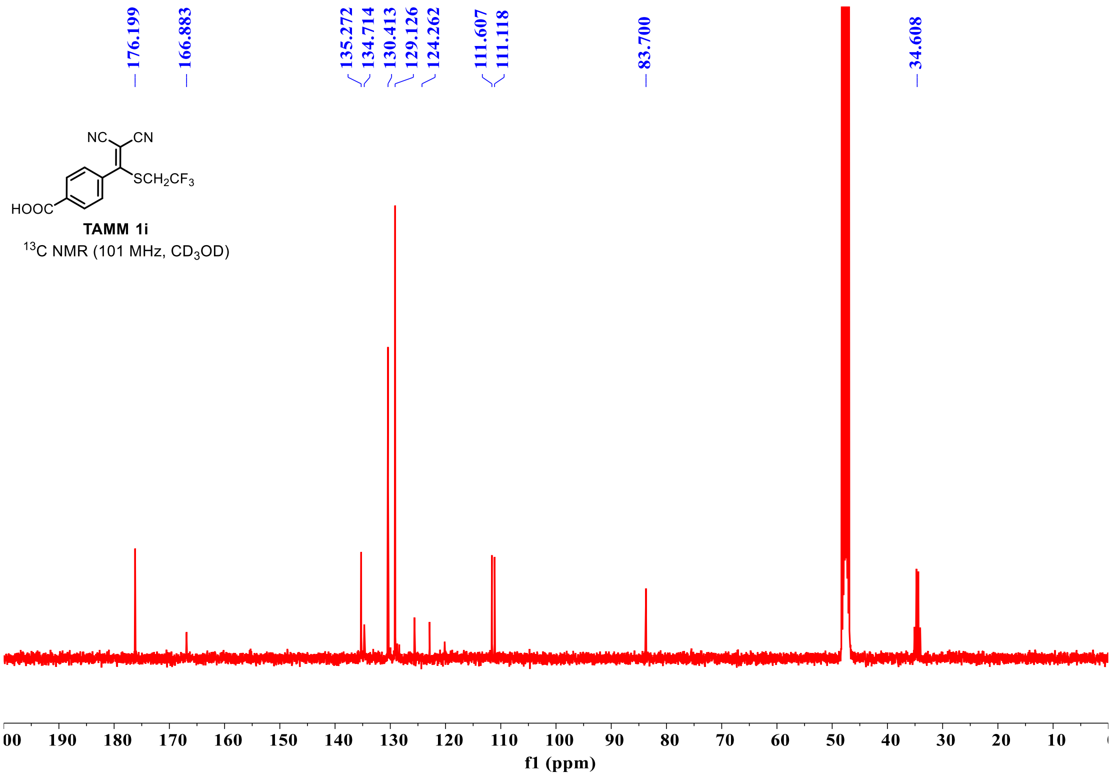

TAMM 1i

^{19}F NMR (376 MHz, CD_3OD)

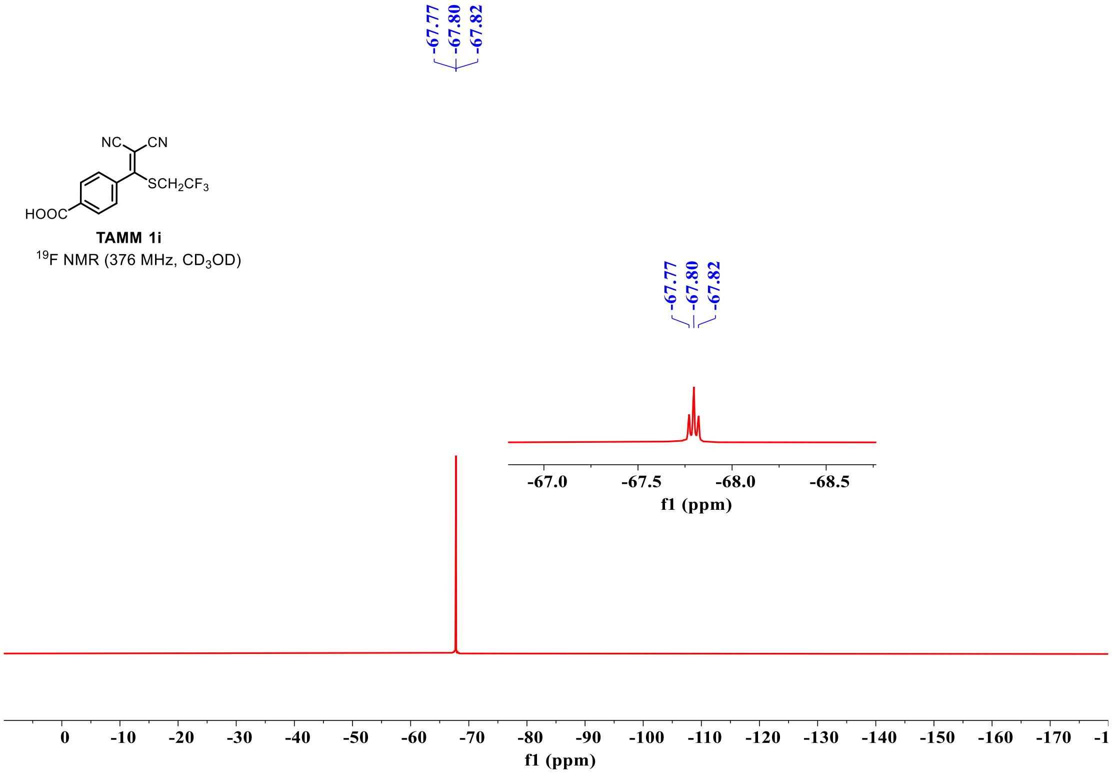

TAMM 1j

^{1}H NMR (300 MHz, Acetone-d_6)

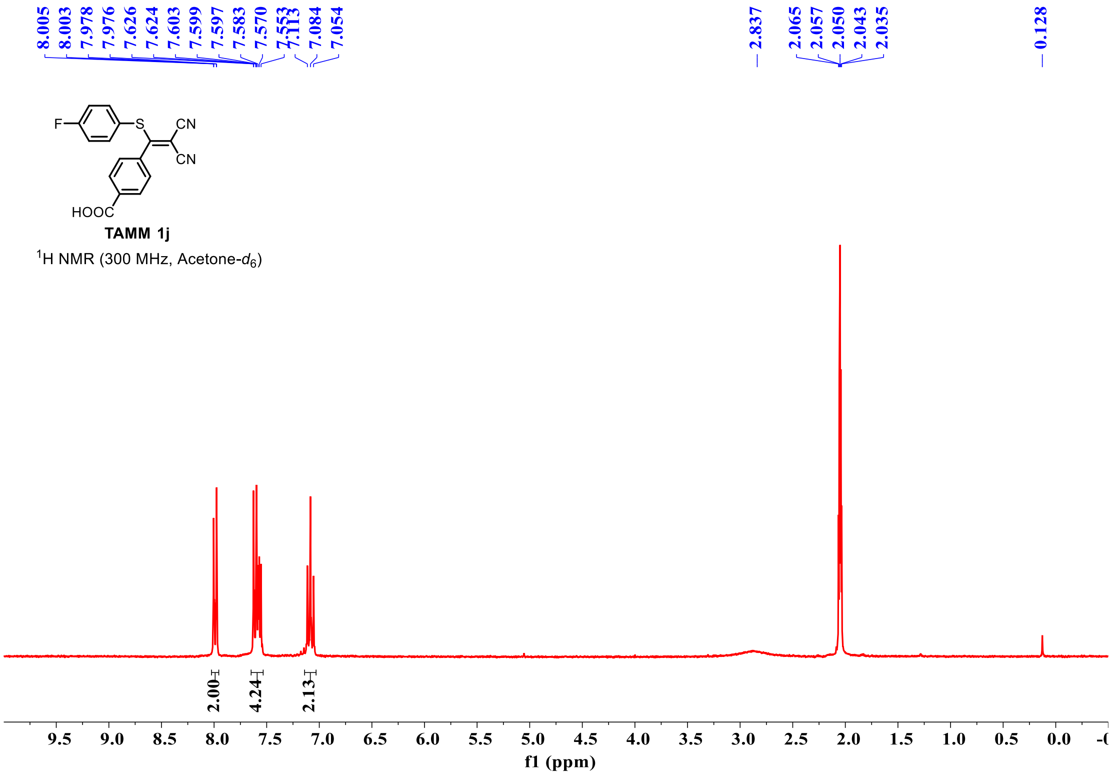

Synthesis of **1j**

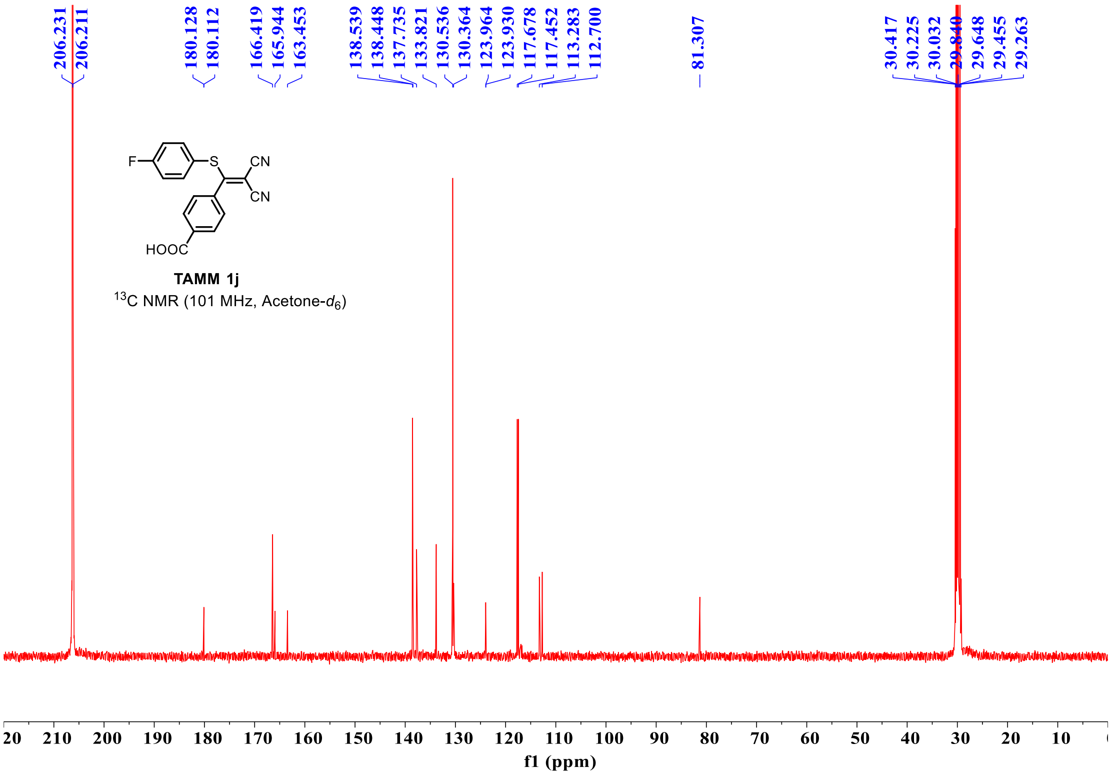

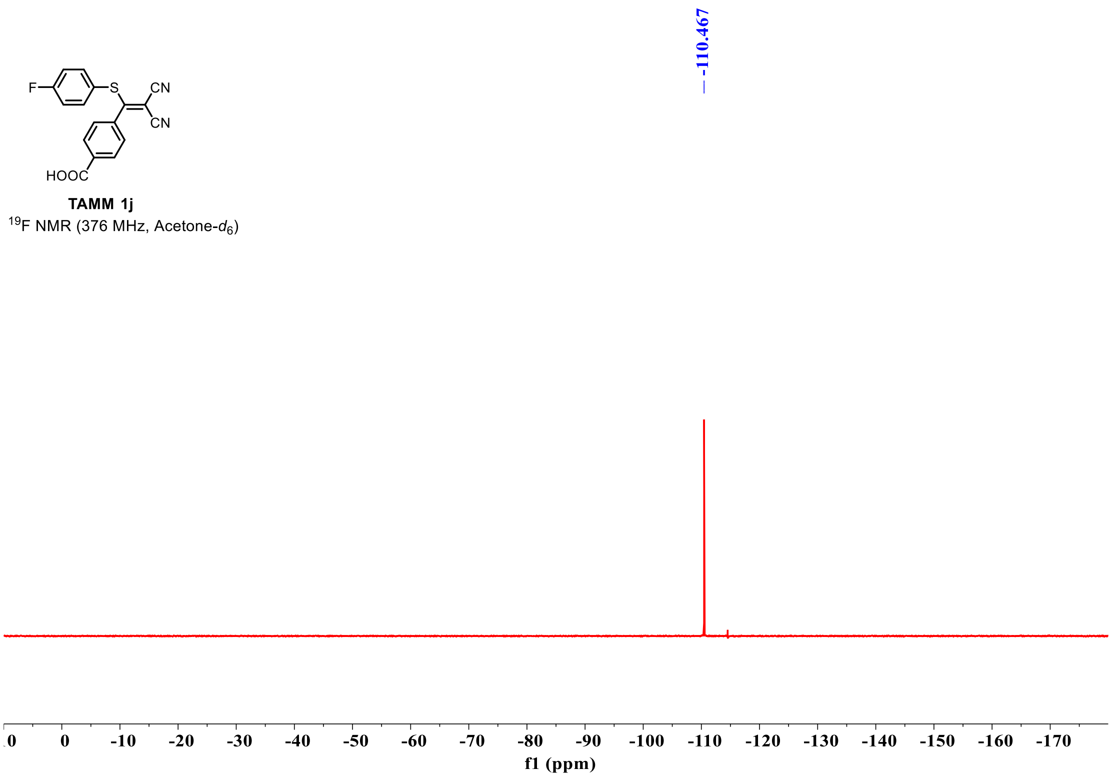

Synthesis of **1k**

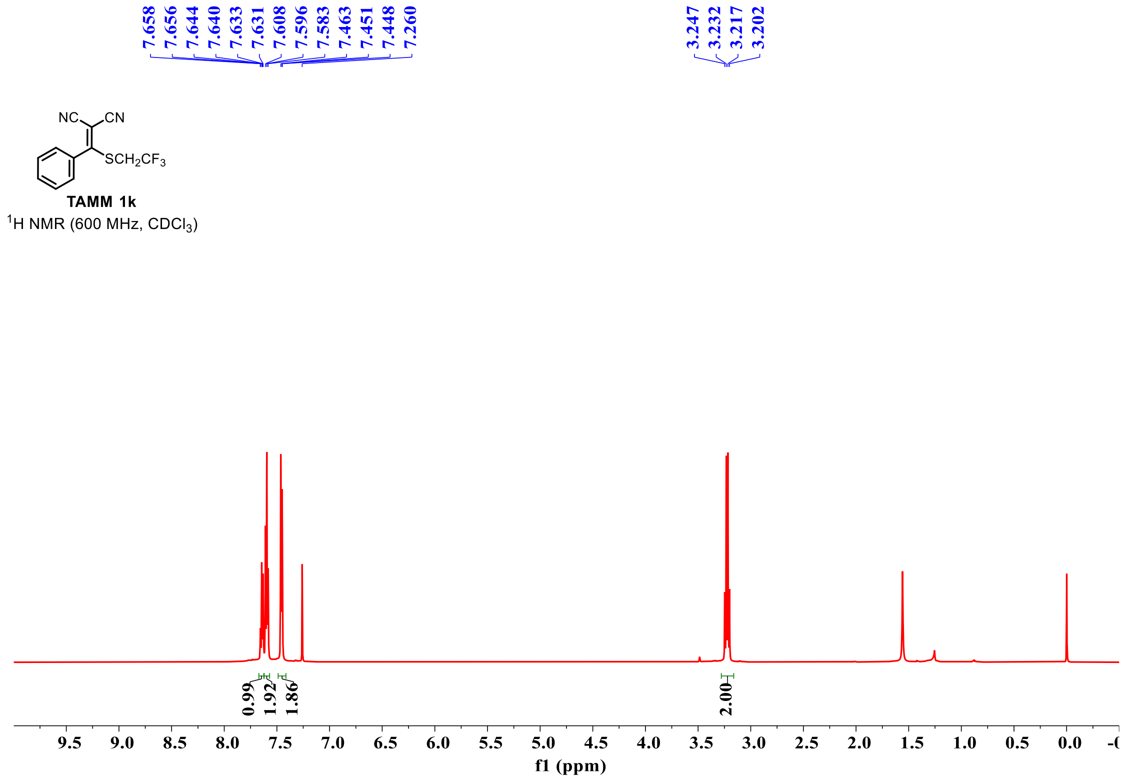

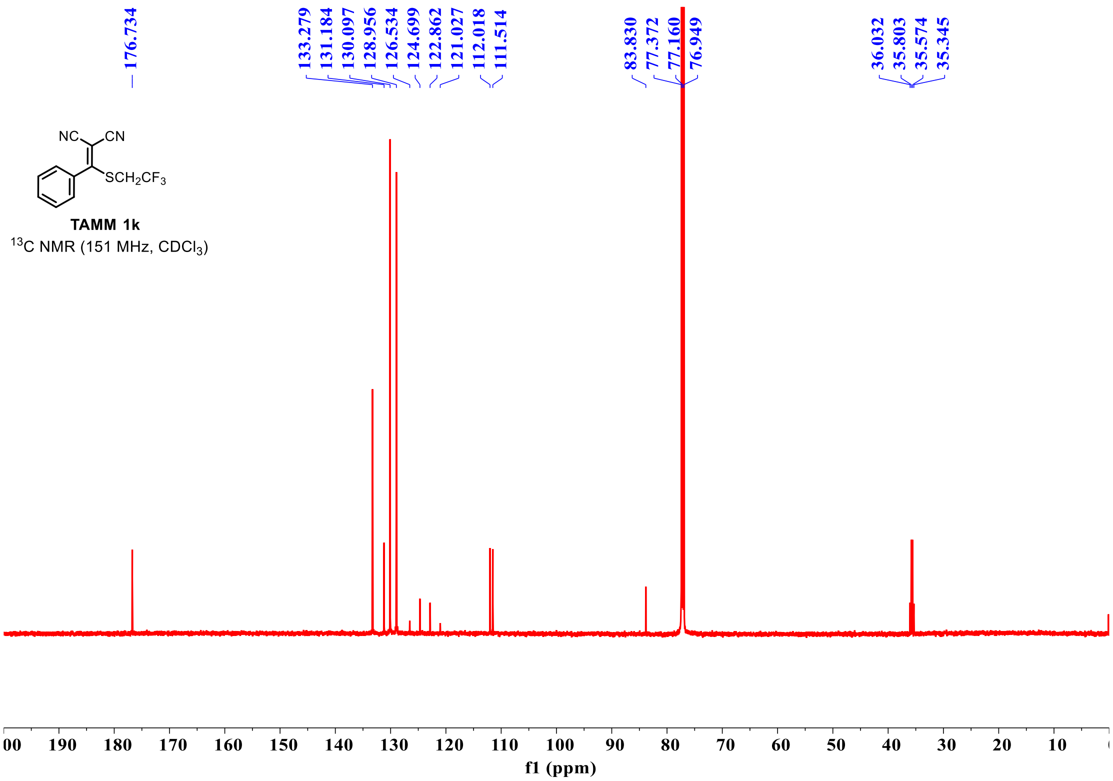

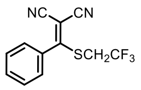

TAMM 1k

^{19}F NMR (376 MHz, $CDCl_3$)

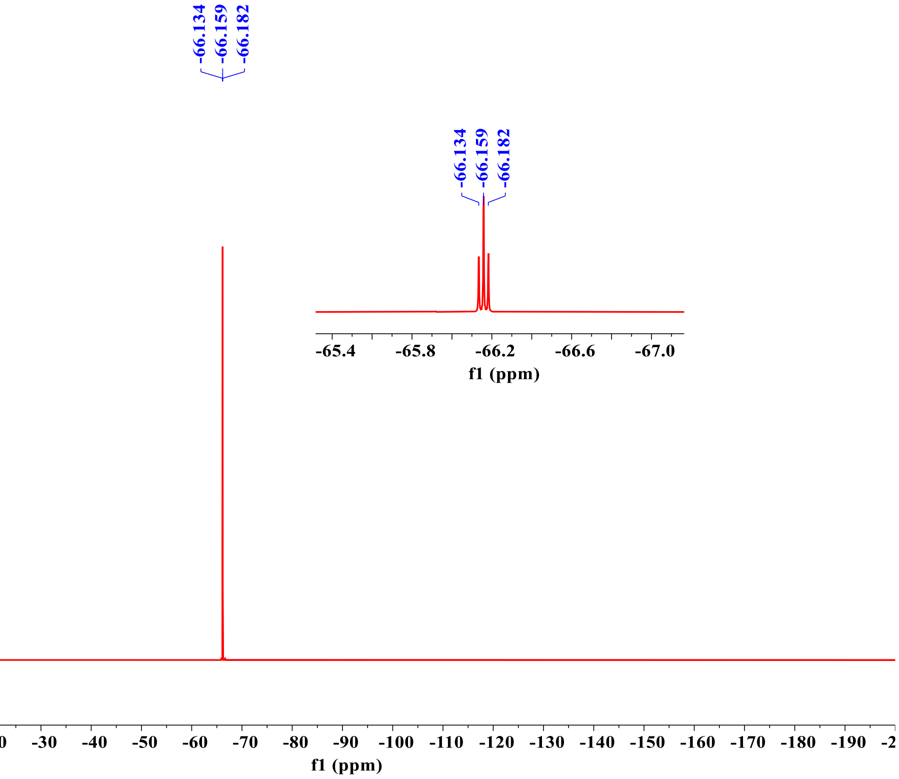

Synthesis of **1l**

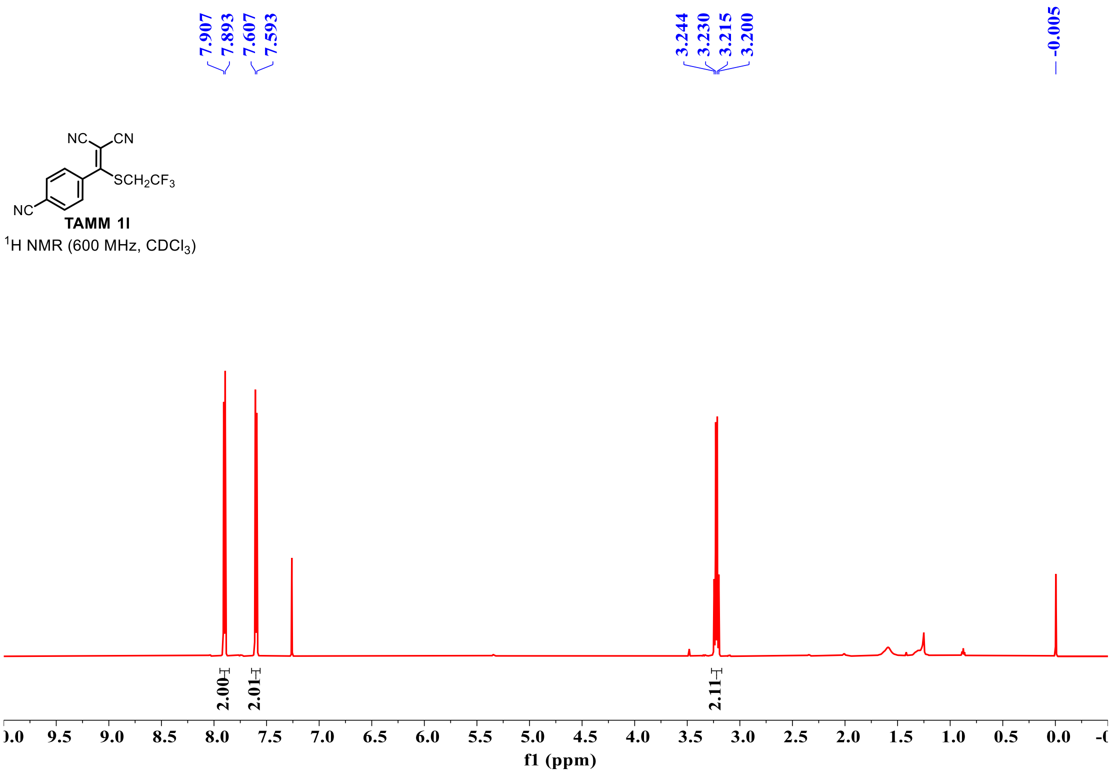

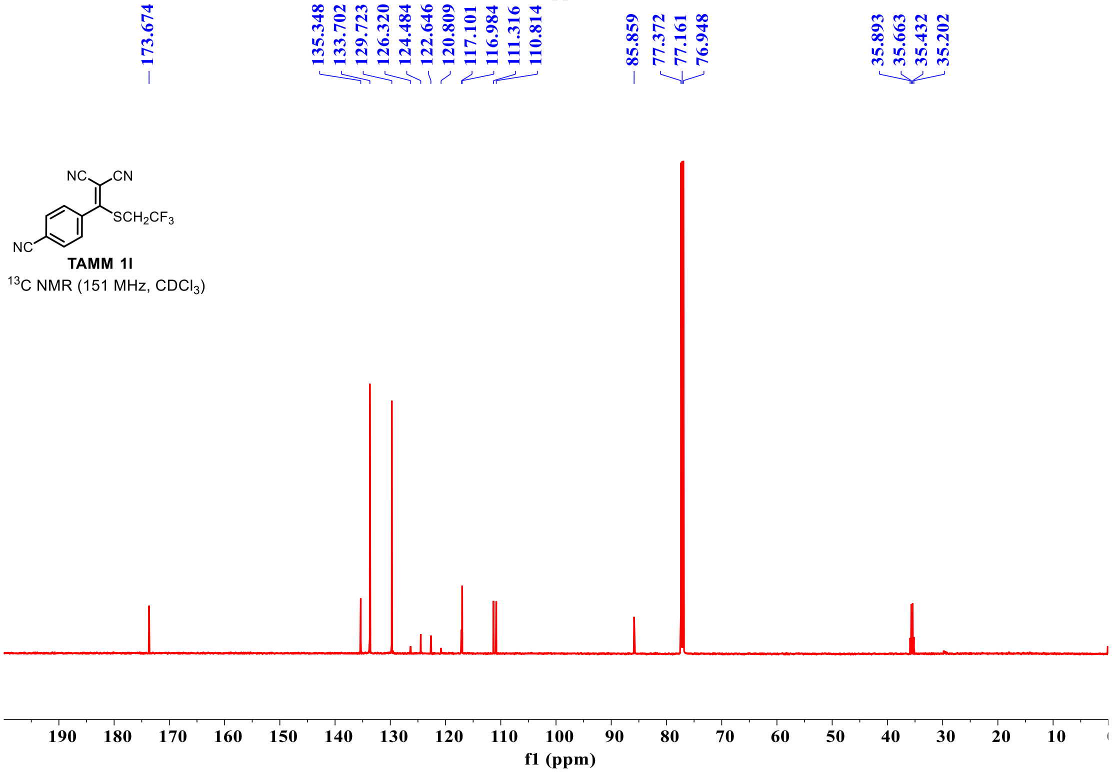

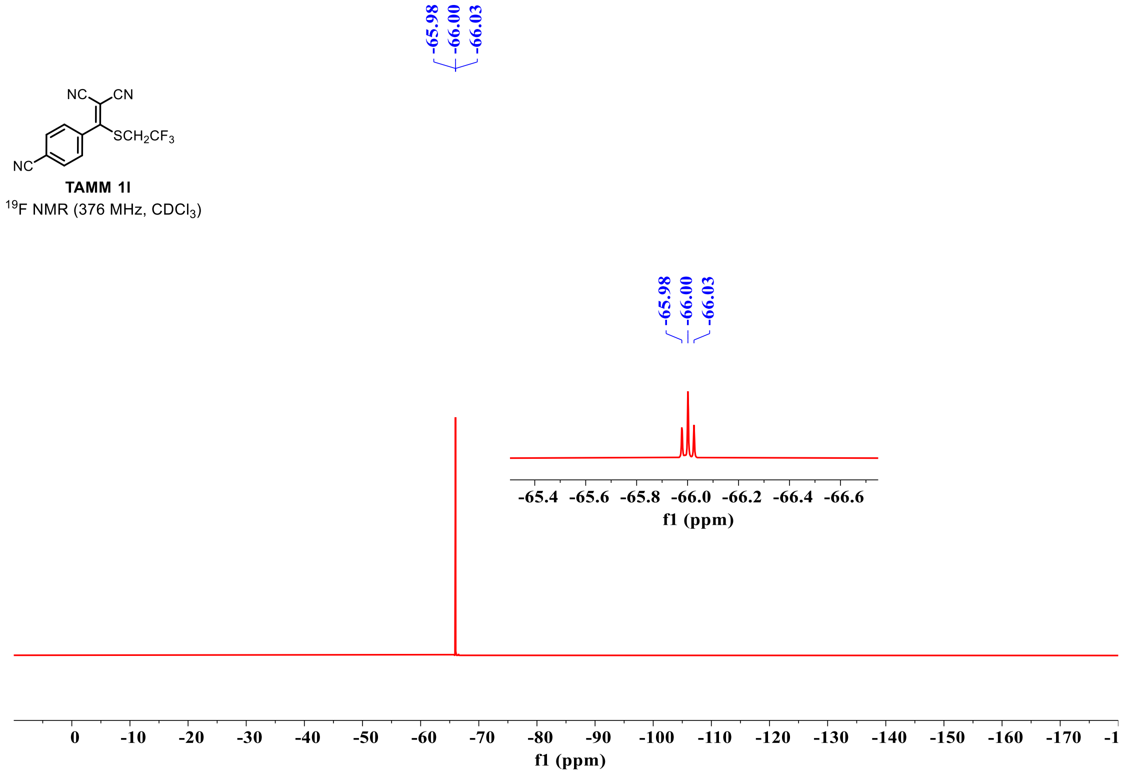
-65.98
-66.00
-66.03
NC
CN
SCH2CF3
NC
TAMM 1l
19F NMR (376 MHz, CDCl3)
-65.98
-66.00
-66.03
-65.4 -65.6 -65.8 -66.0 -66.2 -66.4 -66.6
f1 (ppm)
0 -10 -20 -30 -40 -50 -60 -70 -80 -90 -100 -110 -120 -130 -140 -150 -160 -170 -1
f1 (ppm)

Synthesis of **1m**

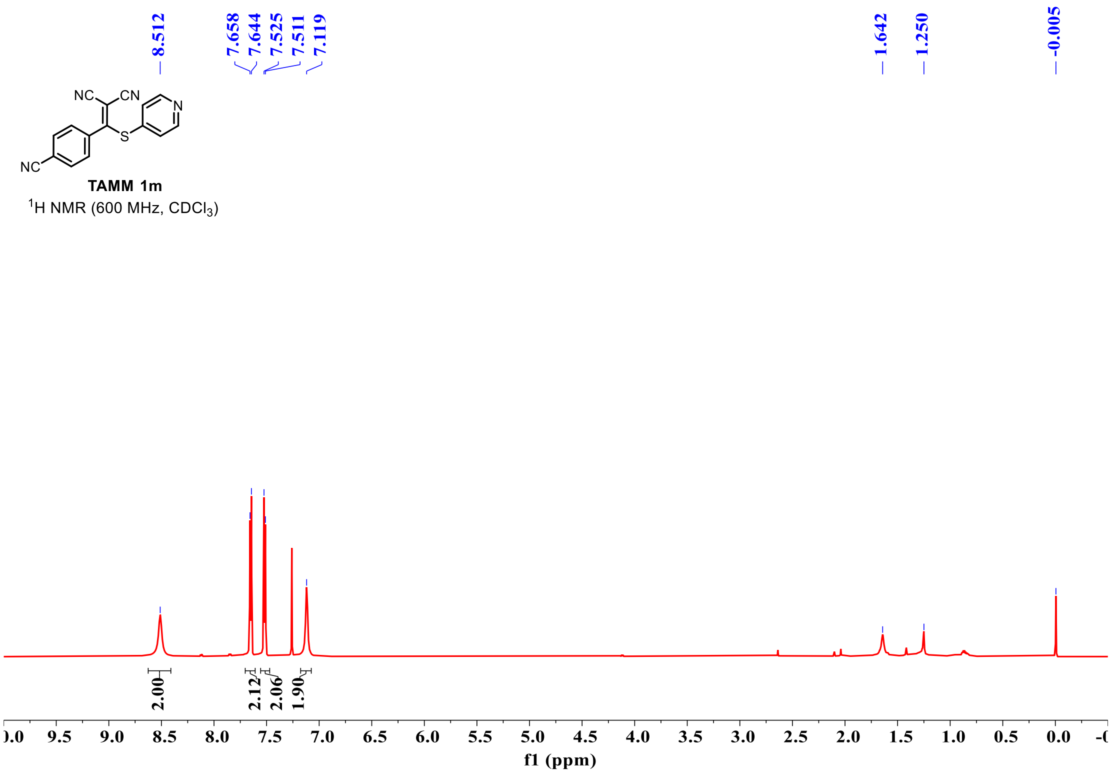

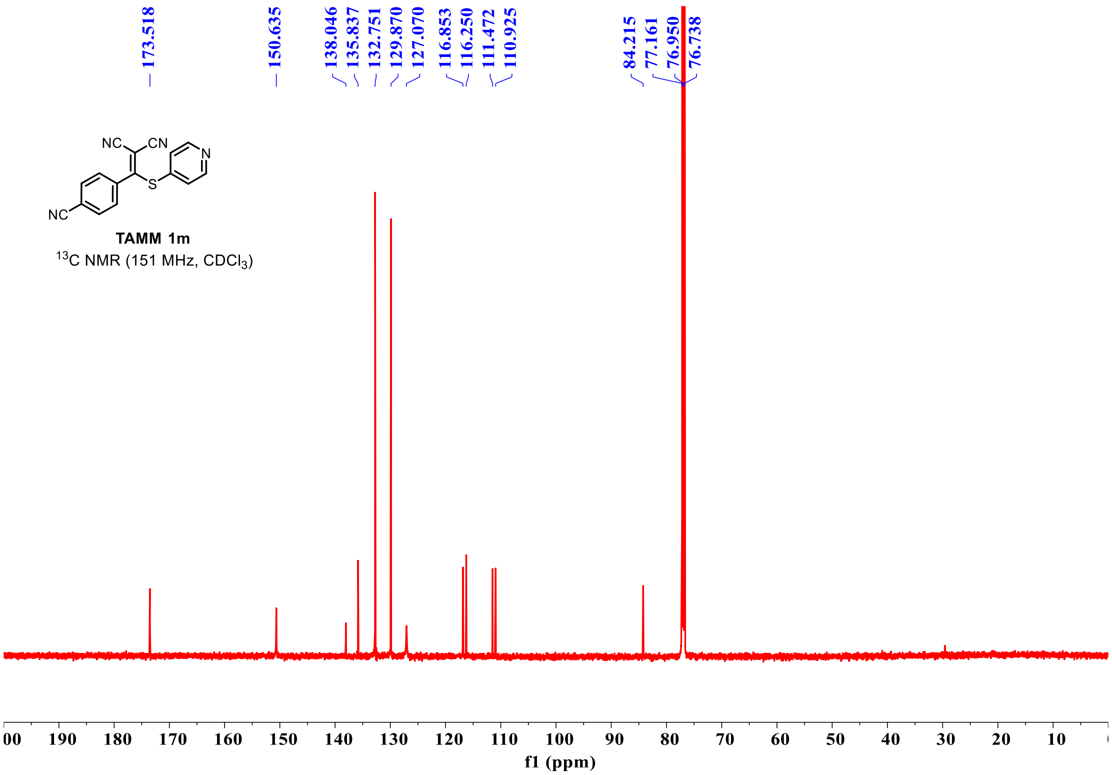

Synthesis of **1n**

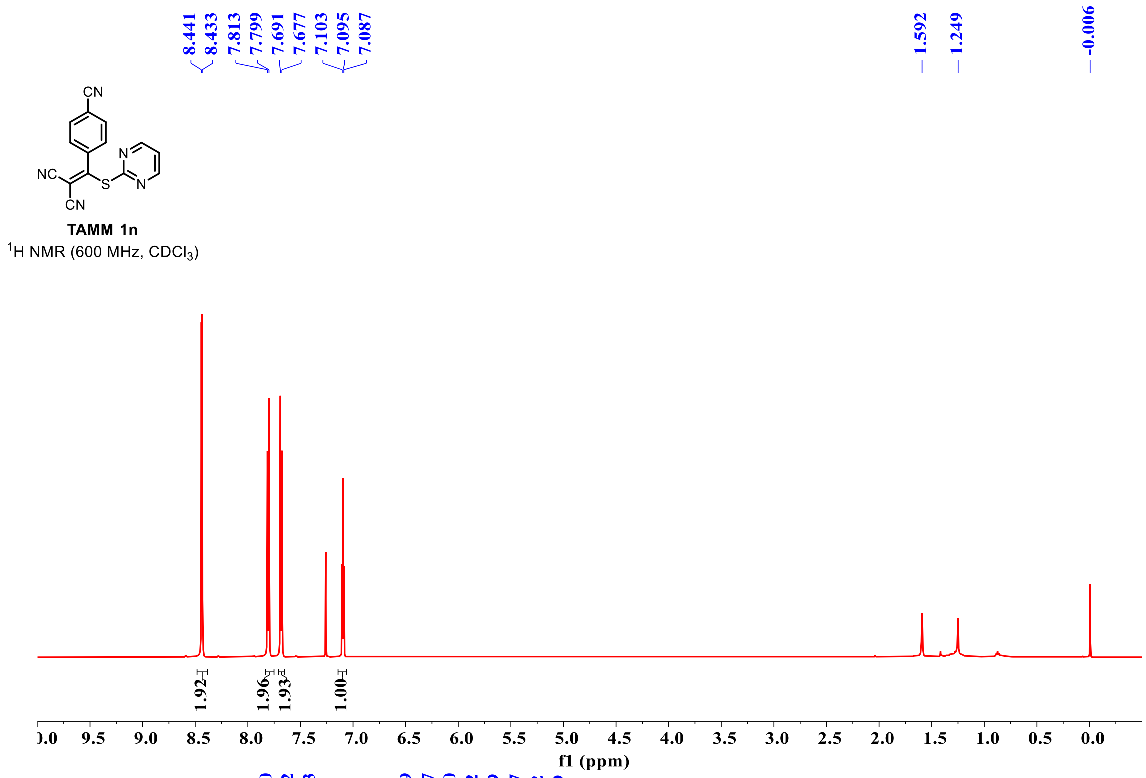

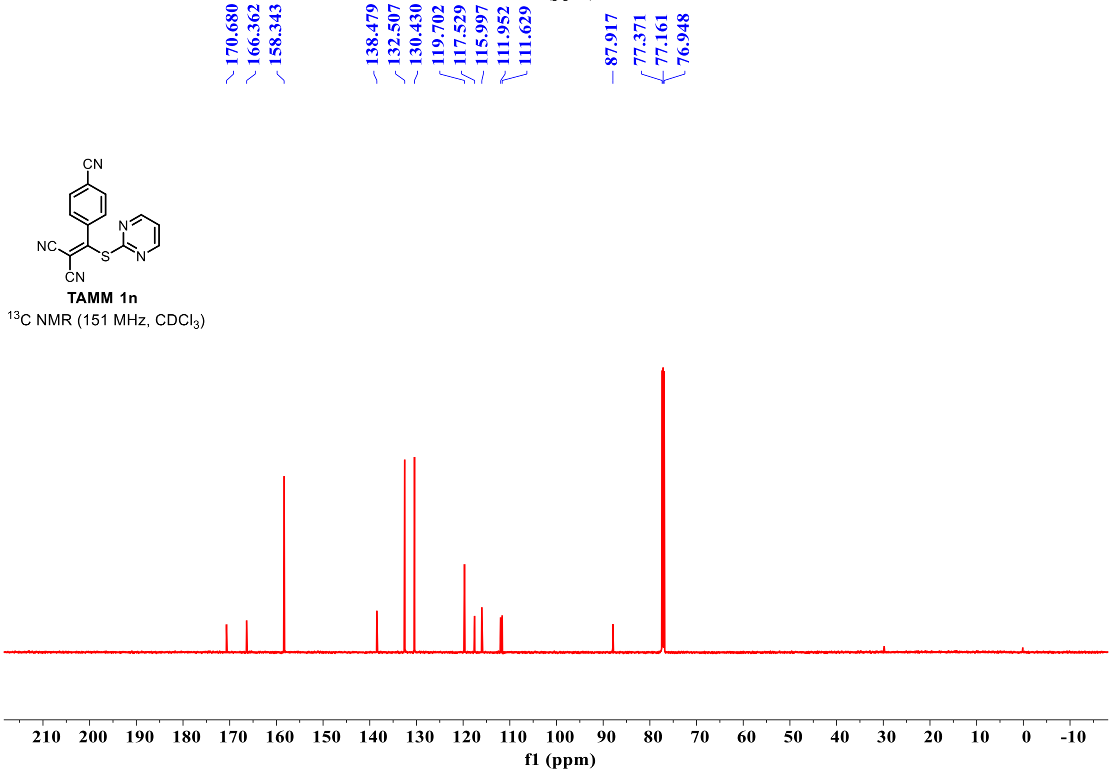

Synthesis of **Cy5-TAMM-SCH_2CF_3**

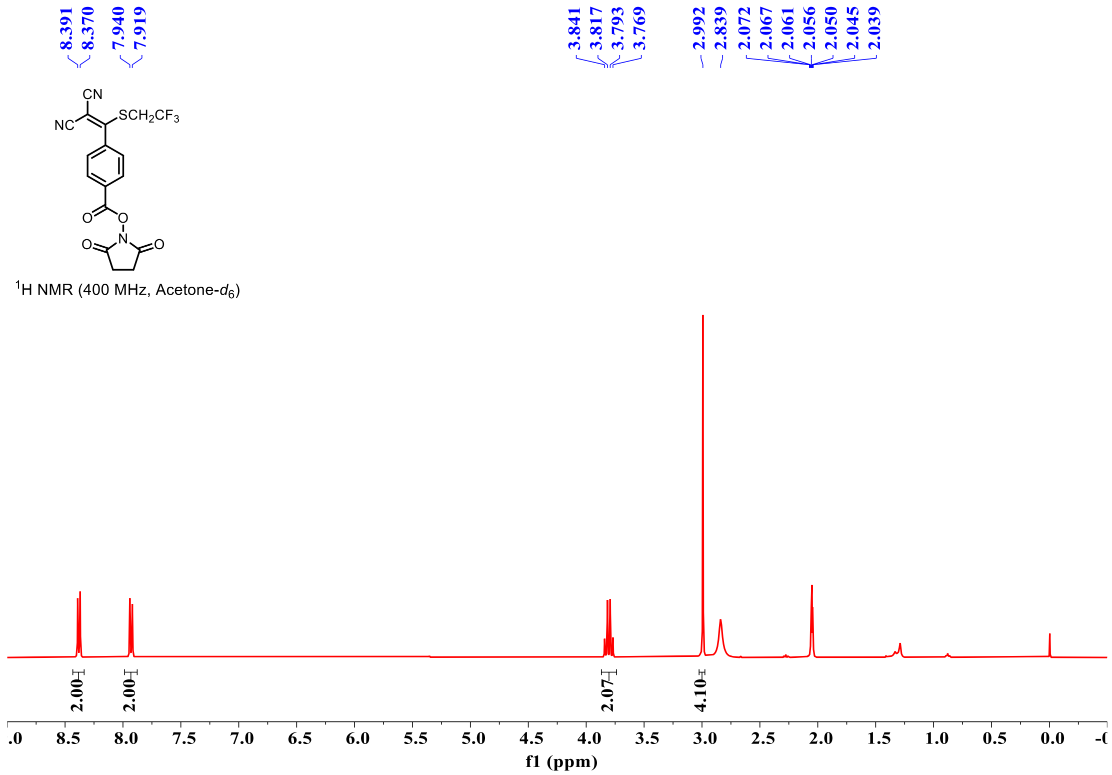

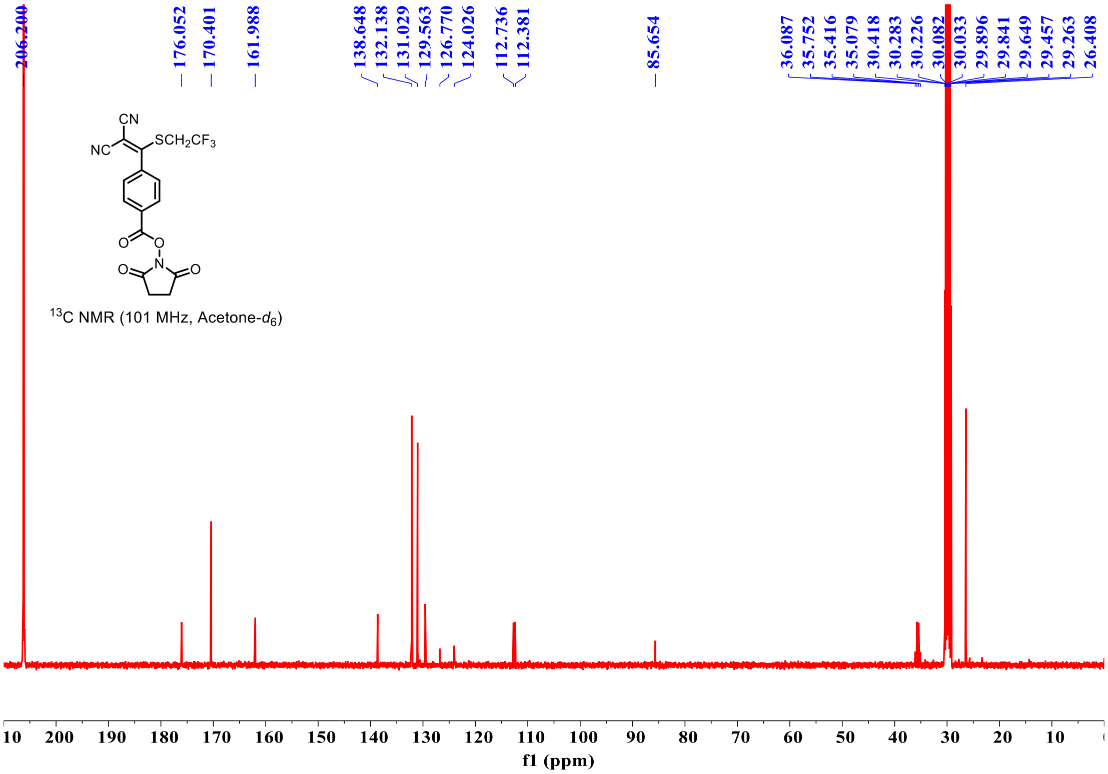

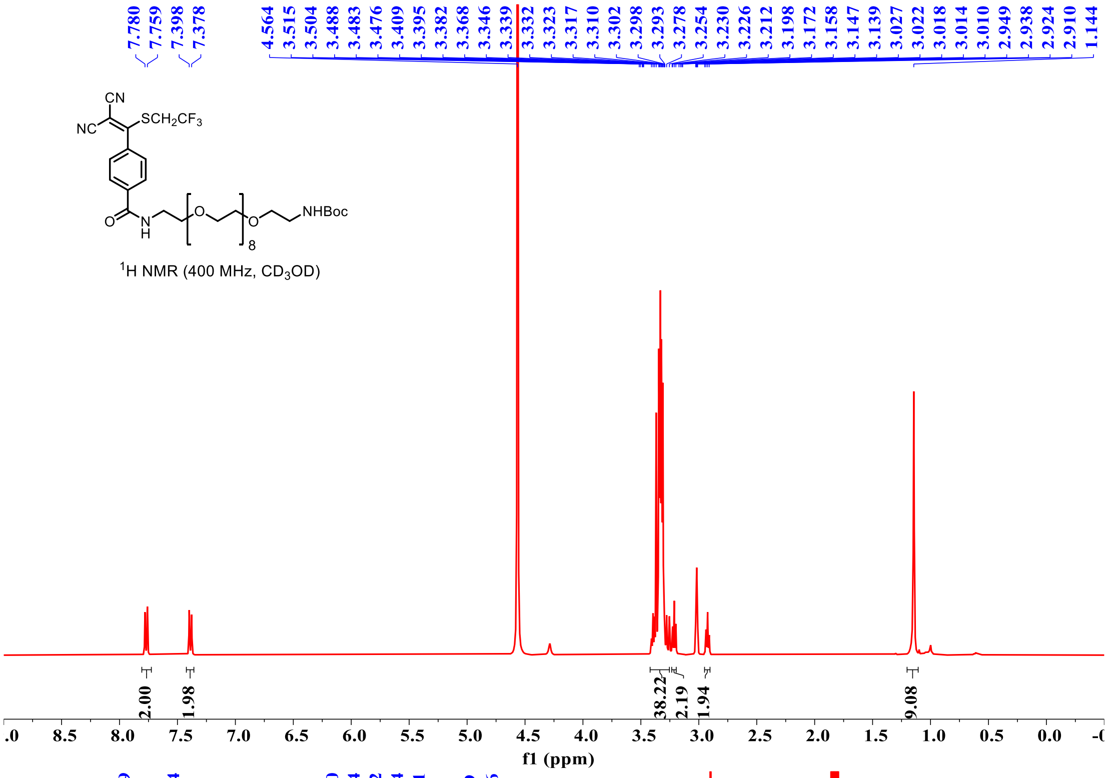
CN
NC
SCH_2CF_3
O
N
H
O
8
O
NHBoc
^1H NMR (400 MHz, CD_3OD)
f1 (ppm)

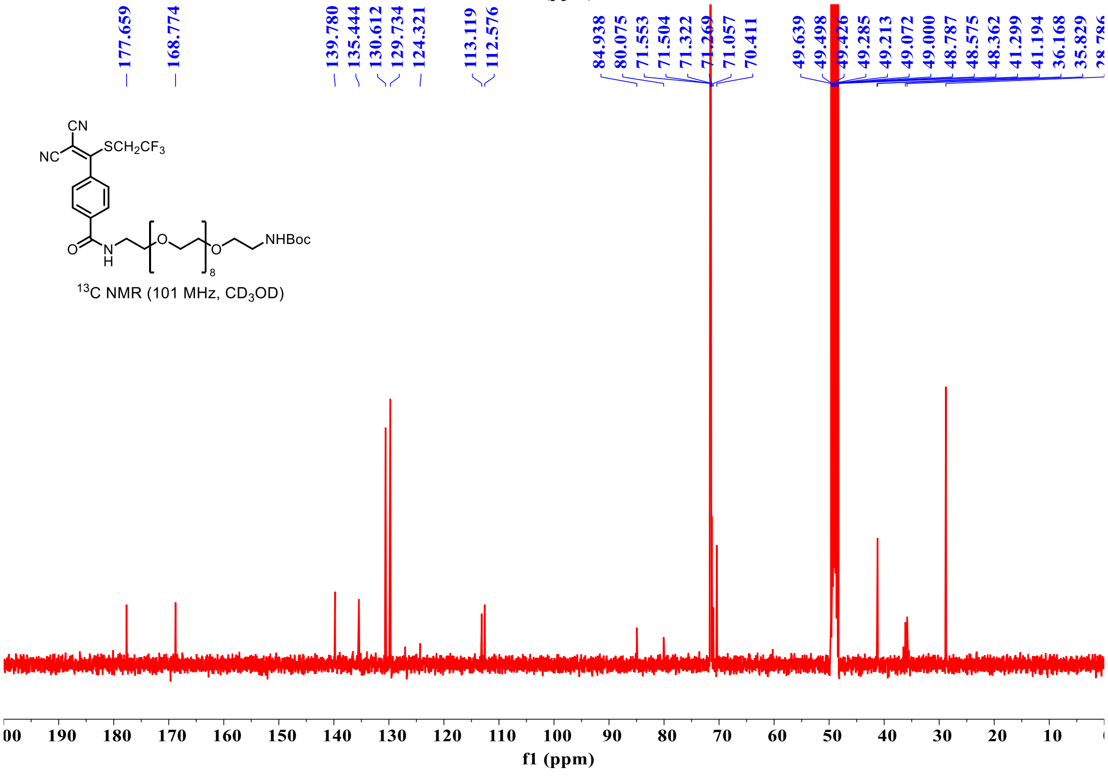
CN
NC
SCH_2CF_3
O
N
H
O
8
O
NHBoc
^{13}C NMR (101 MHz, CD_3OD)
f1 (ppm)

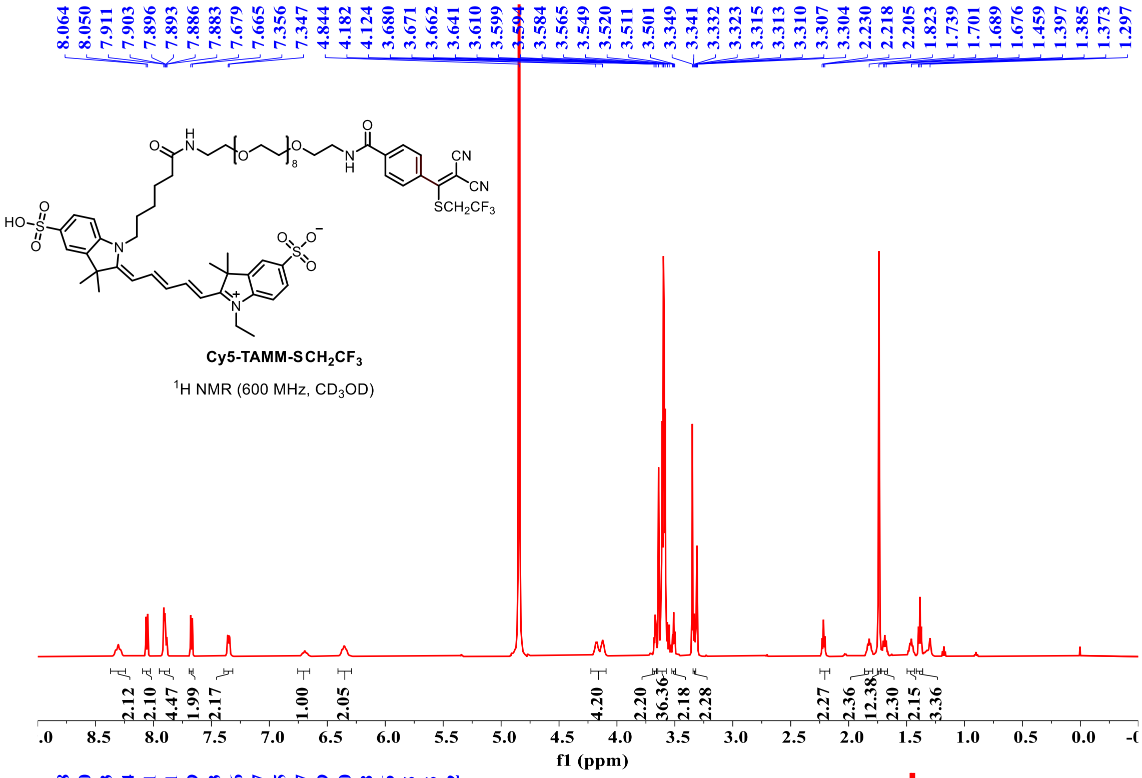

Cy5-TAMM-SCH$_2$CF$_3$

^{1}H NMR (600 MHz, CD$_3$OD)

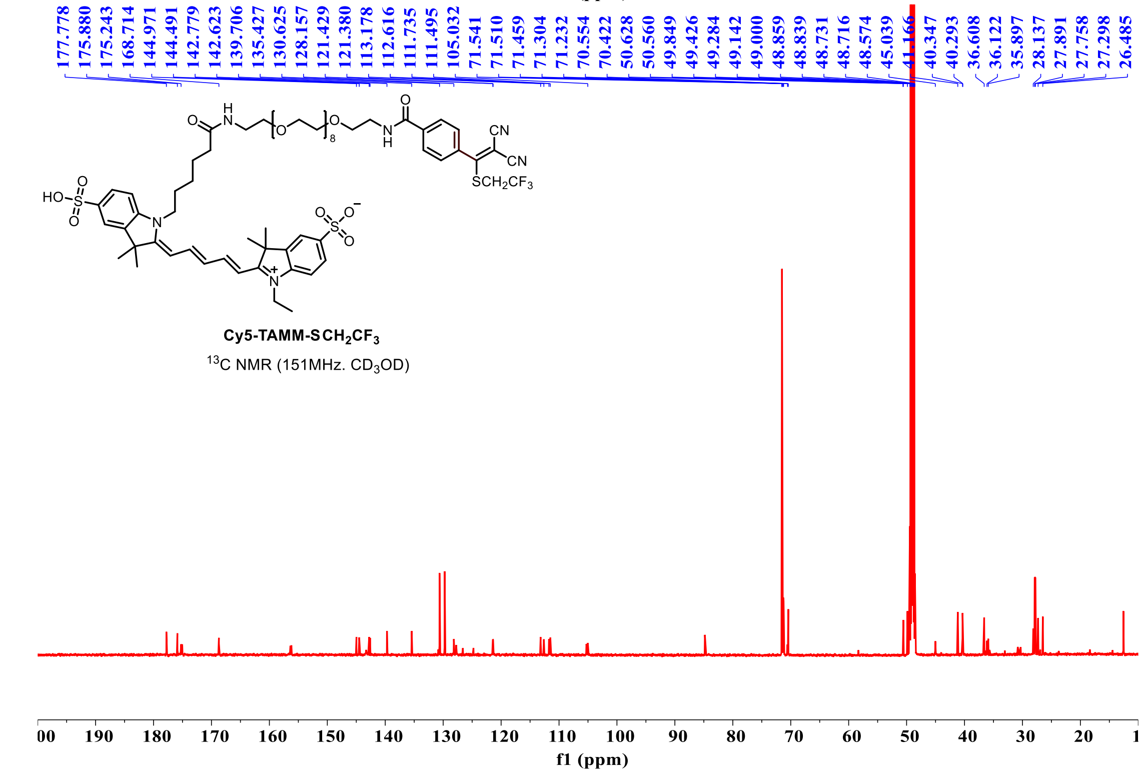

Cy5-TAMM-SCH$_2$CF$_3$

^{13}C NMR (151MHz. CD$_3$OD)

Synthesis of **Cy3-TAMM-SCH2CF3**

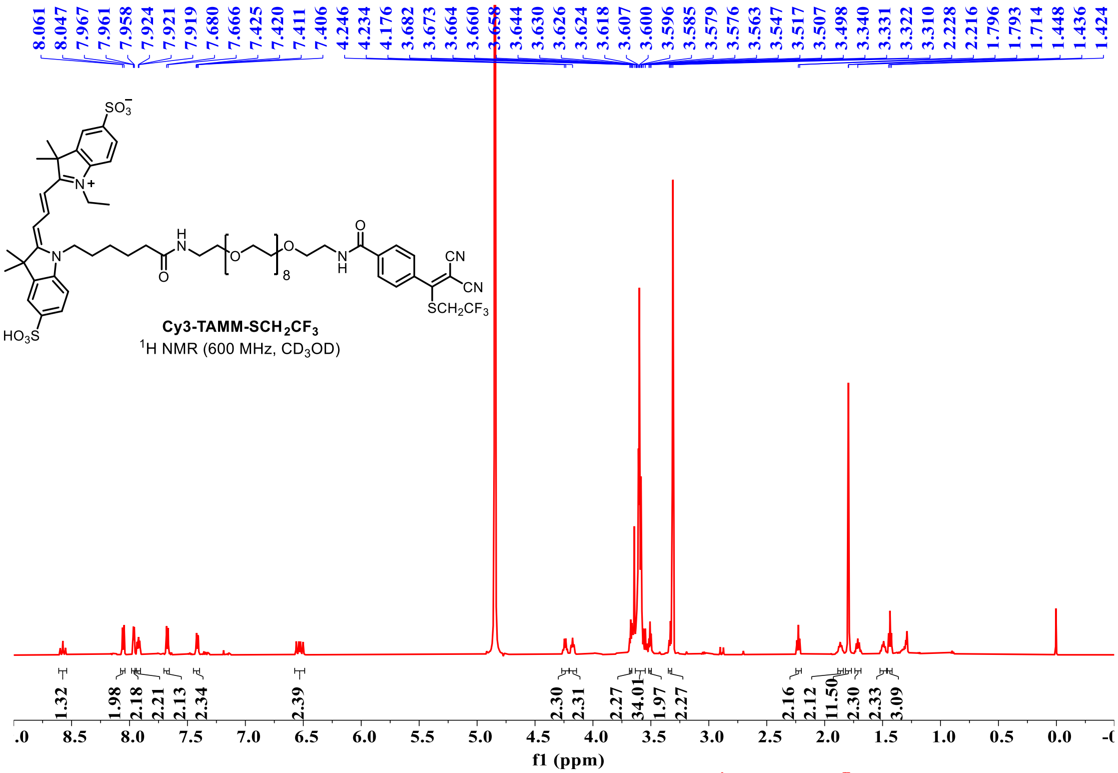

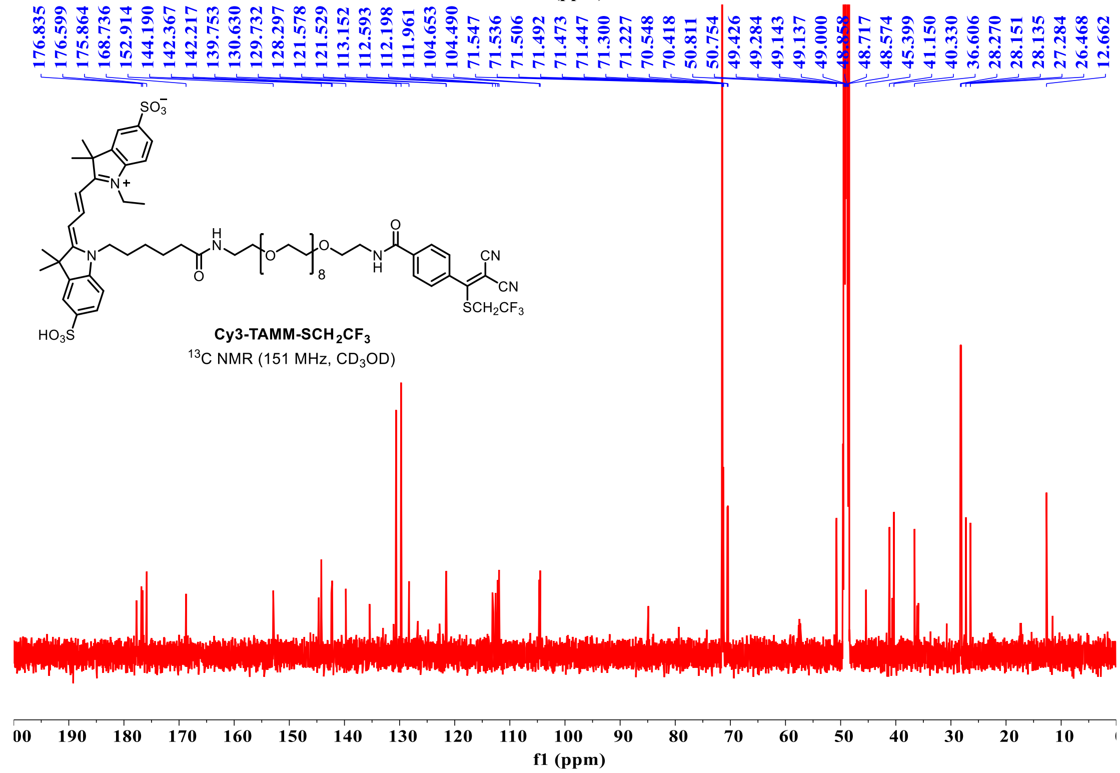

Synthesis of **Cy5-TAMM-SEt**

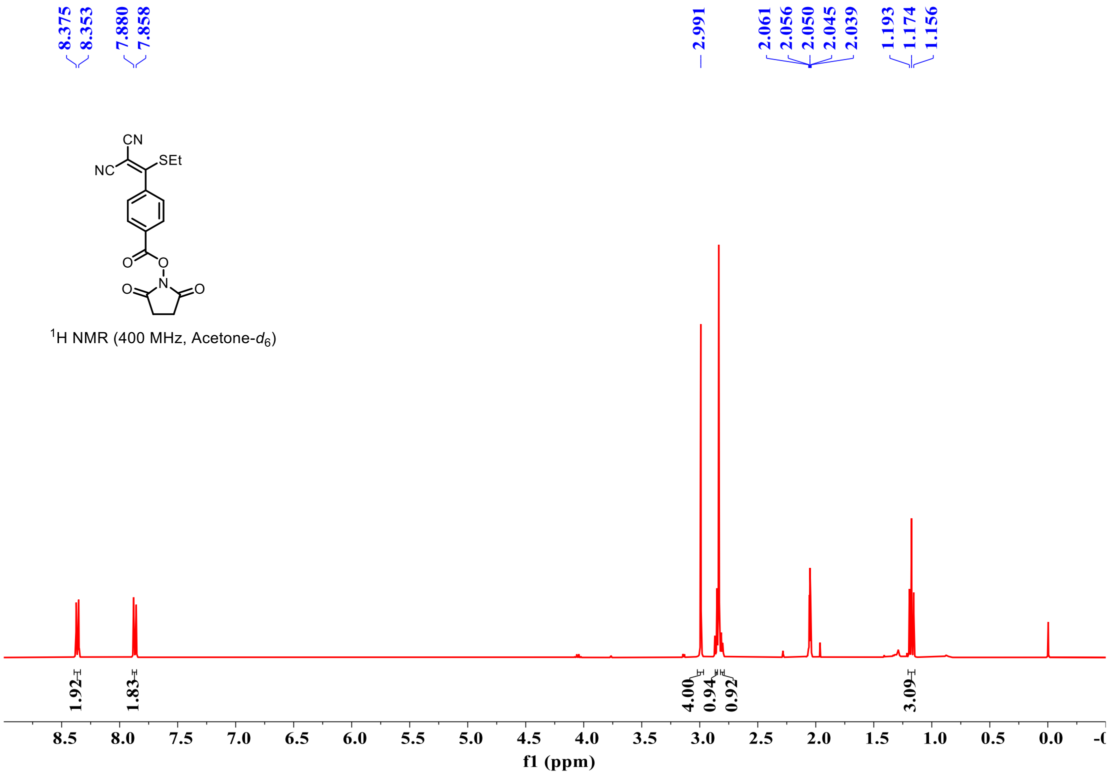

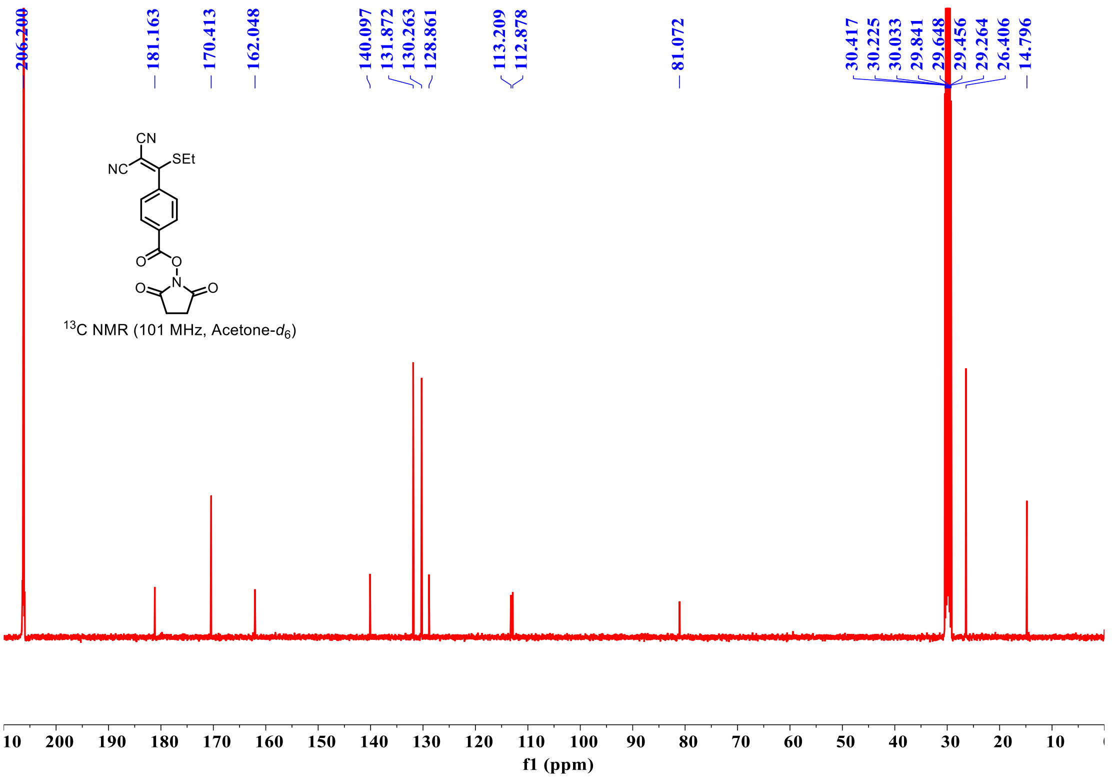

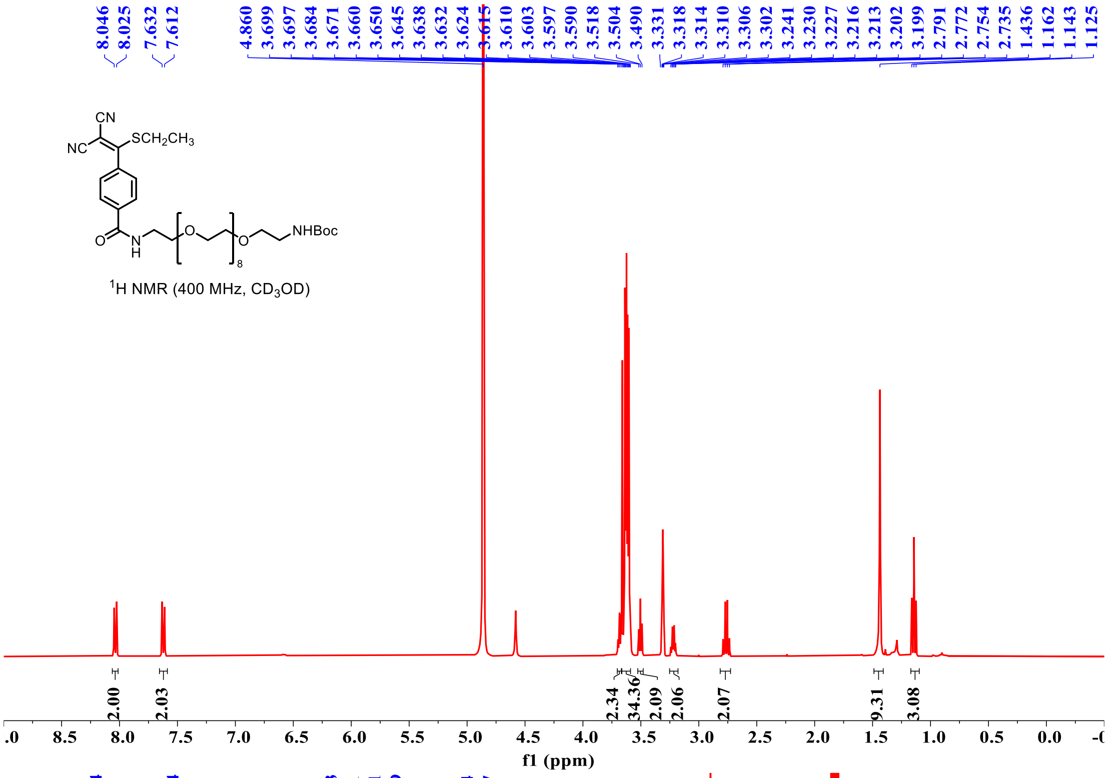
8.046
8.025
7.632
7.612
4.860
3.699
3.697
3.684
3.671
3.660
3.650
3.645
3.638
3.632
3.624
3.615
3.610
3.603
3.597
3.590
3.518
3.504
3.490
3.331
3.318
3.314
3.310
3.306
3.302
3.241
3.230
3.227
3.216
3.213
3.202
3.199
2.791
2.772
2.754
2.735
1.436
1.162
1.143
1.125
CN
SCH_2CH_3
NC
O
N
H
O
O
8
NHBoc
^1H NMR (400 MHz, CD_3OD)
2.00
2.03
2.34
34.36
2.09
2.06
2.07
9.31
3.08
f1 (ppm)

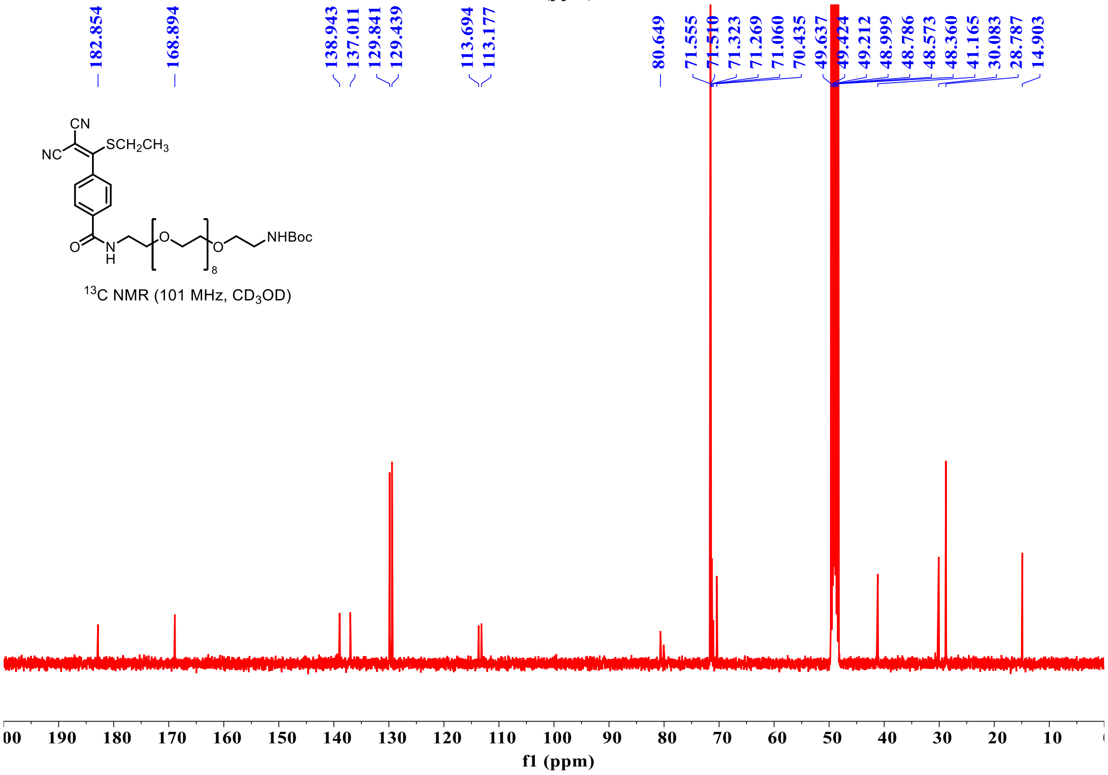
182.854
168.894
138.943
137.011
129.841
129.439
113.694
113.177
80.649
71.555
71.510
71.323
71.269
71.060
70.435
49.637
49.424
49.212
48.999
48.786
48.573
48.360
41.165
30.083
28.787
14.903
CN
SCH_2CH_3
NC
O
N
H
O
O
8
NHBoc
^{13}C NMR (101 MHz, CD_3OD)
f1 (ppm)

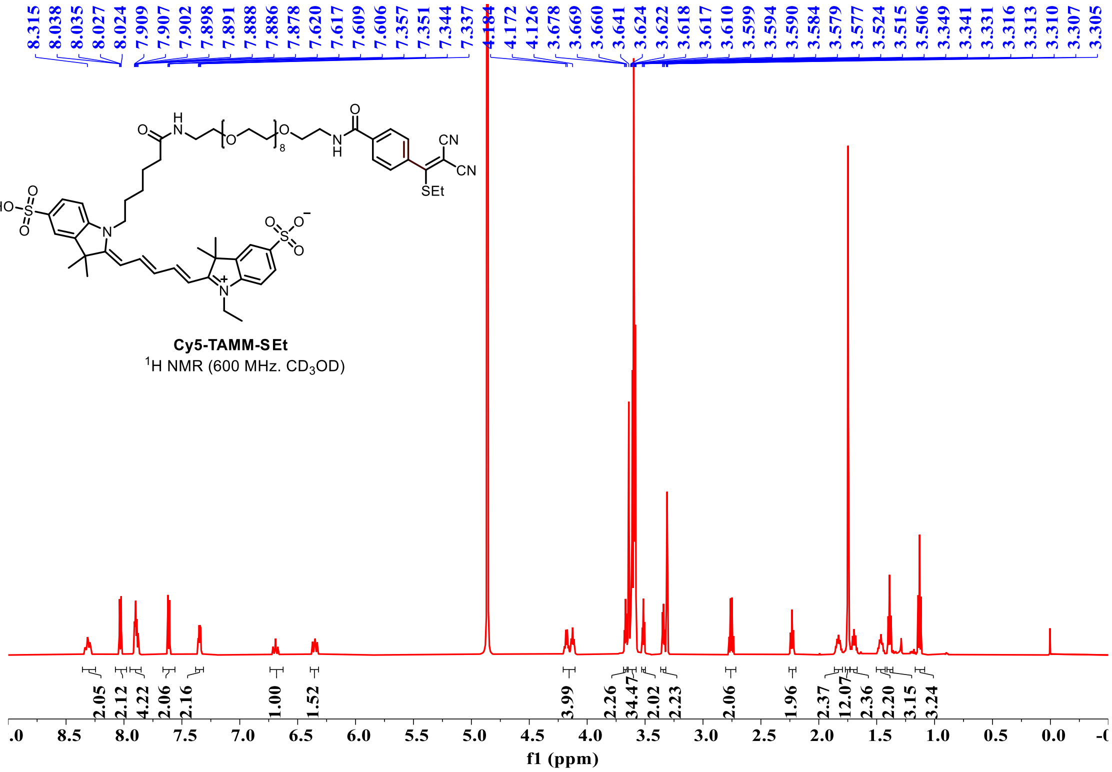
8.315 8.038 8.035 8.027 8.024 7.909 7.907 7.902 7.898 7.891 7.888 7.886 7.878 7.620 7.617 7.609 7.606 7.357 7.351 7.344 7.337 4.184 4.172 4.126 3.678 3.669 3.660 3.641 3.624 3.622 3.618 3.617 3.610 3.599 3.594 3.590 3.584 3.579 3.577 3.524 3.515 3.506 3.349 3.341 3.331 3.316 3.313 3.310 3.307 3.305
Cy5-TAMM-SEt
1H NMR (600 MHz. CD3OD)
2.05 2.12 4.22 2.06 2.16 1.00 1.52 3.99 2.26 34.47 2.02 2.23 2.06 1.96 2.37 12.07 2.36 2.20 3.15 3.24
f1 (ppm)

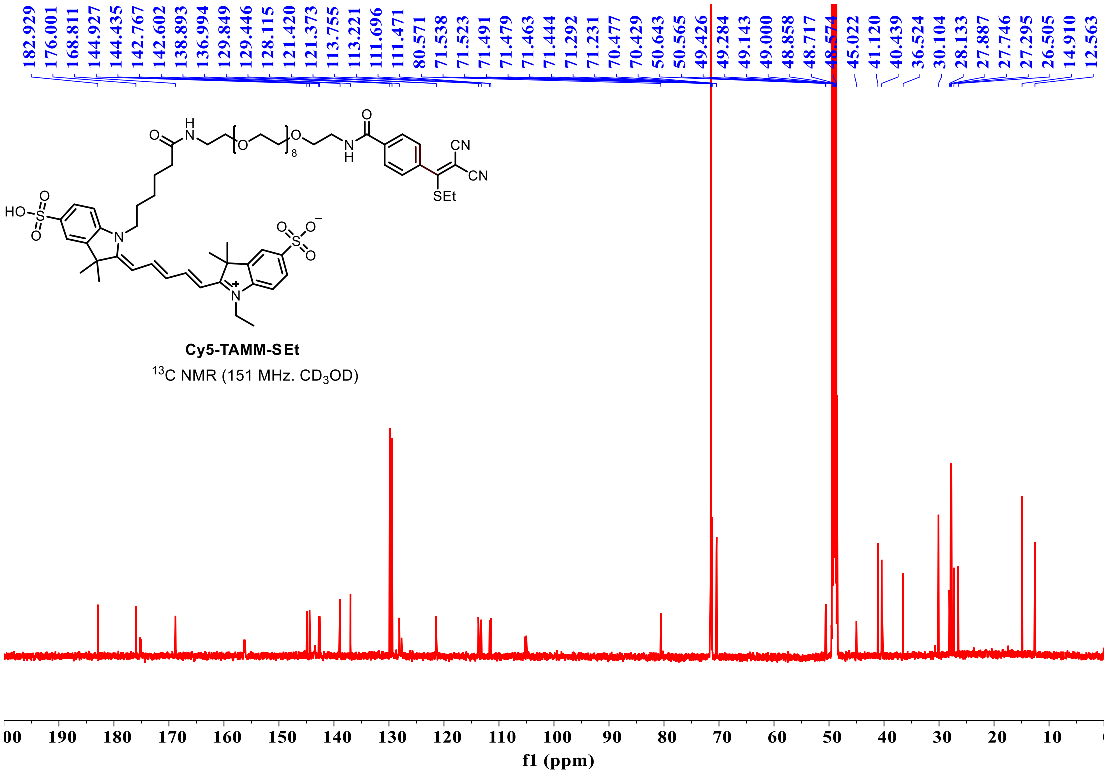
182.929 176.001 168.811 144.927 144.435 142.767 142.602 138.893 136.994 129.849 129.446 128.115 121.420 121.373 113.755 113.221 111.696 111.471 80.571 71.538 71.523 71.491 71.479 71.463 71.444 71.292 71.231 70.477 70.429 50.643 50.565 49.426 49.284 49.143 49.000 48.858 48.717 48.574 45.022 41.120 40.439 36.524 30.104 28.133 27.887 27.746 27.295 26.505 14.910 12.563
Cy5-TAMM-SEt
13C NMR (151 MHz. CD3OD)
f1 (ppm)

Synthesis of **Cy5-TAMM-SPhF**

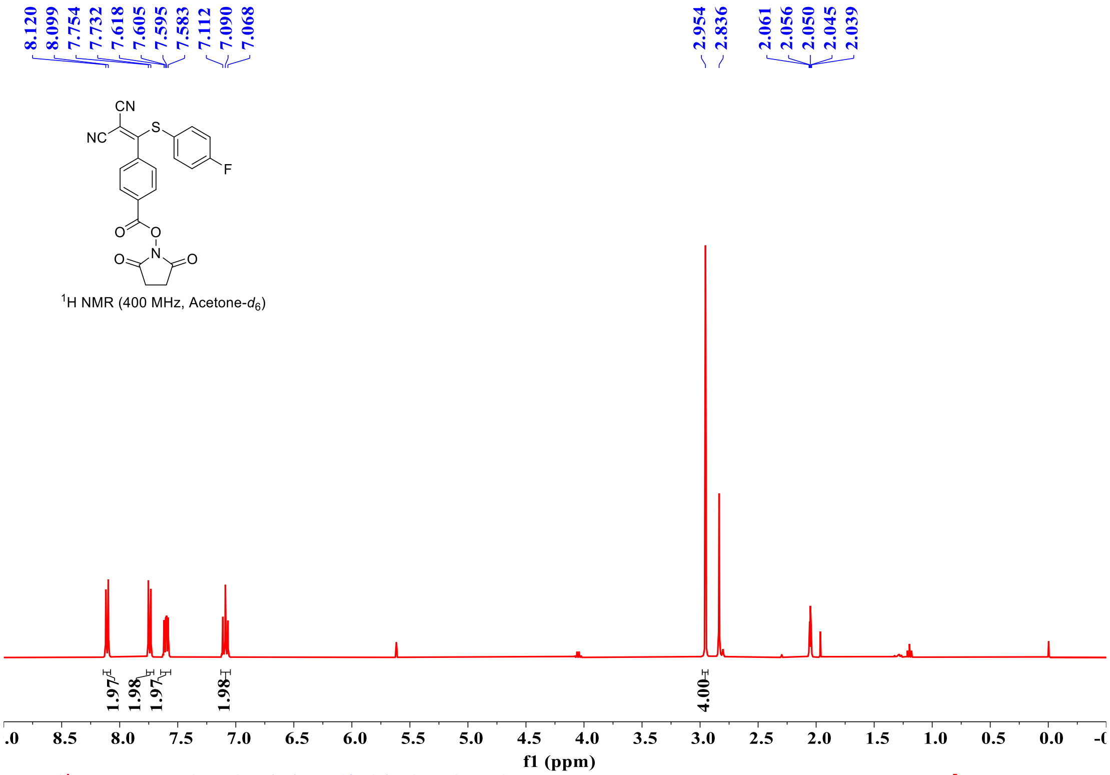

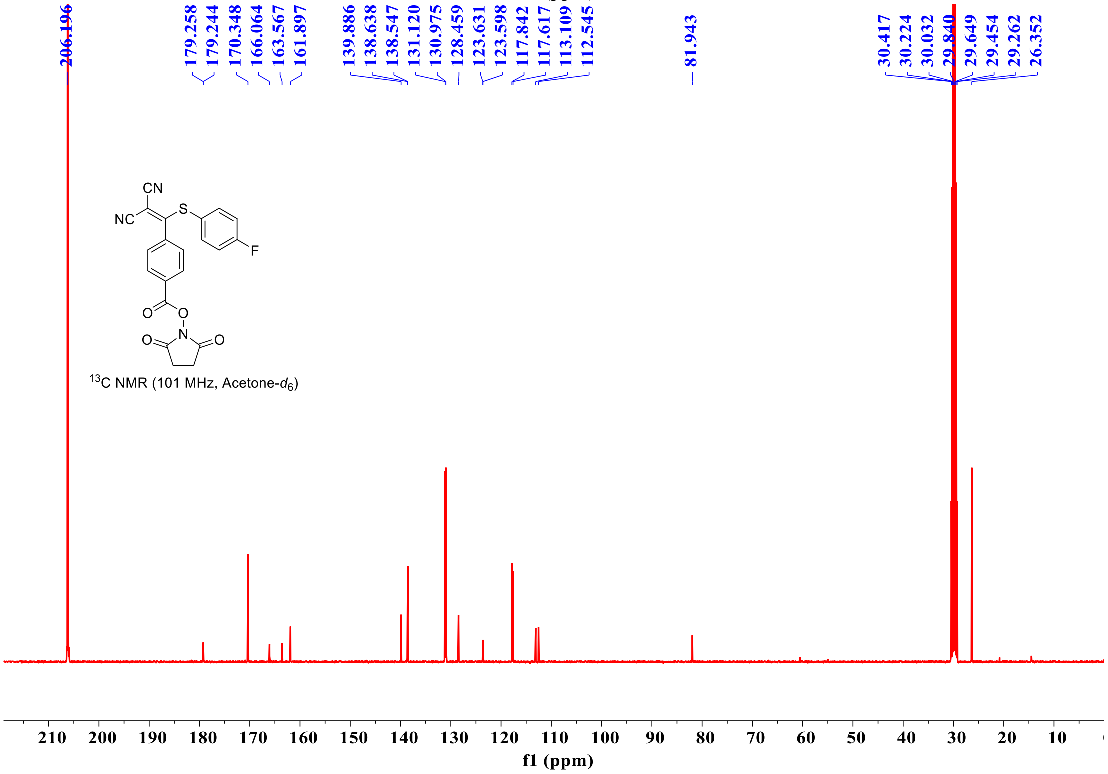

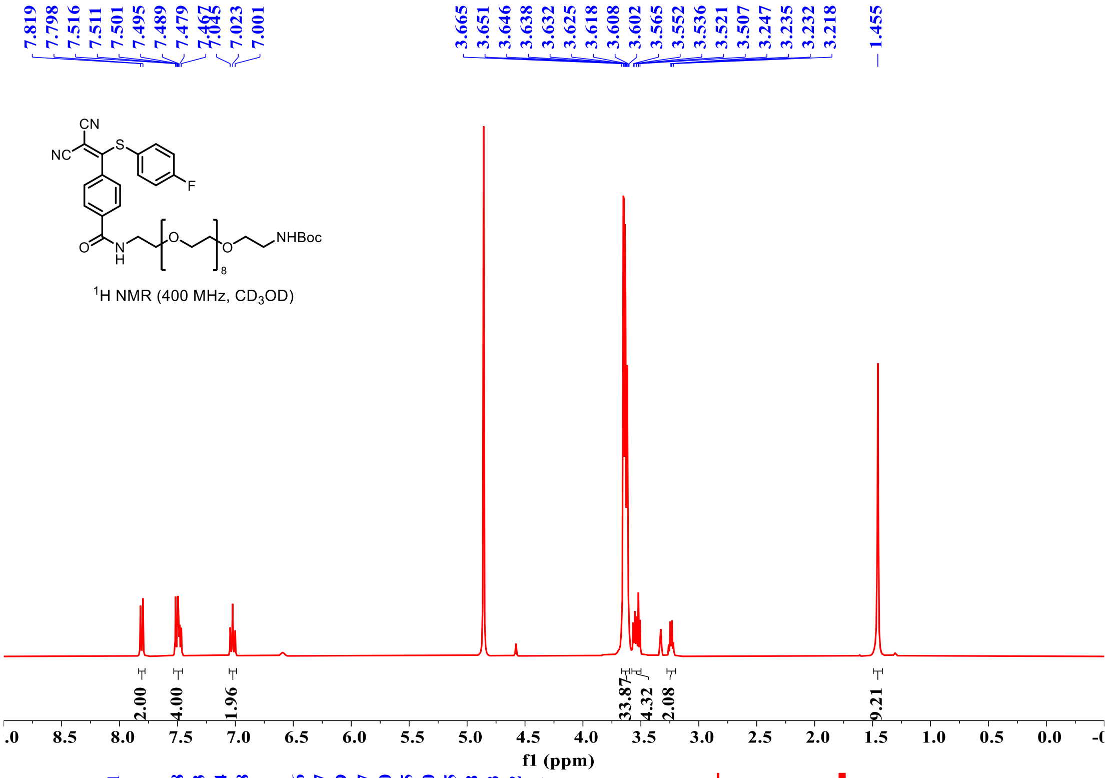
7.819 7.798 7.516 7.511 7.501 7.495 7.489 7.479 7.467 7.045 7.023 7.001
3.665 3.651 3.646 3.638 3.632 3.625 3.618 3.608 3.602 3.565 3.552 3.536 3.521 3.507 3.247 3.235 3.232 3.218
1.455
CN
NC
S
F
O
N
H
O
O
8
NHBoc
^{1}H NMR (400 MHz, CD_3OD)
2.00
4.00
1.96
33.87
4.32
2.08
9.21
f1 (ppm)

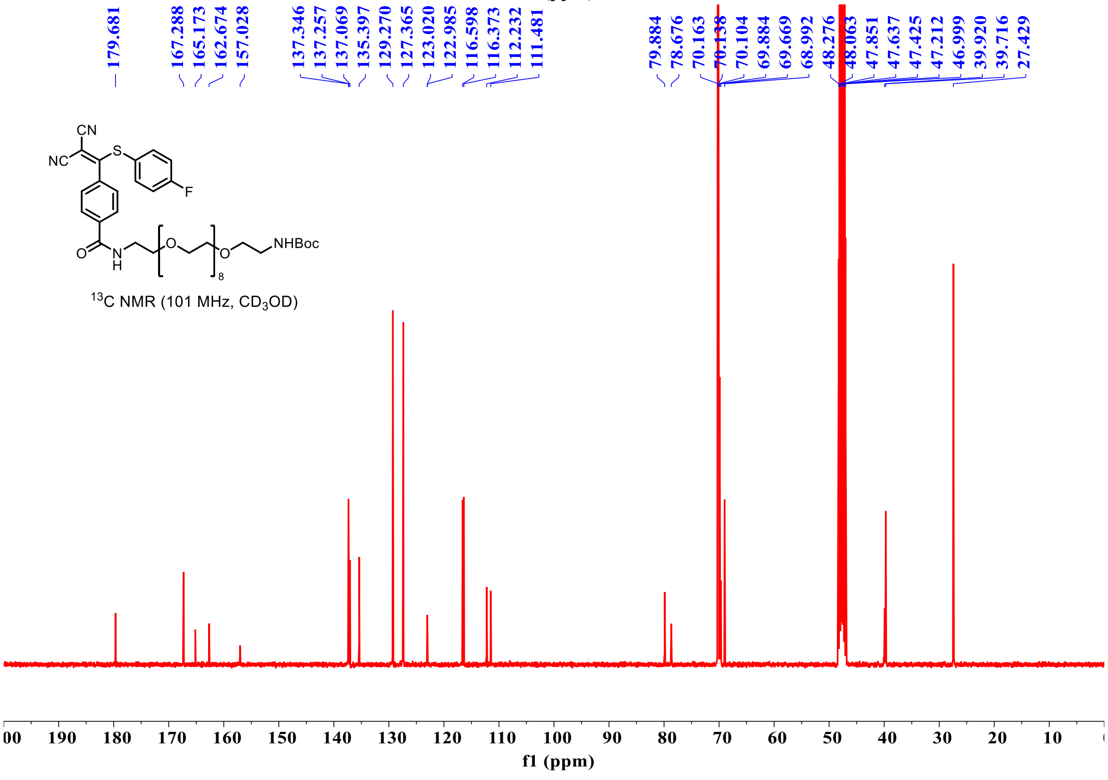
179.681
167.288 165.173 162.674 157.028
137.346 137.257 137.069 135.397 129.270 127.365 123.020 122.985 116.598 116.373 112.232 111.481
79.884 78.676 70.163 70.138 70.104 69.884 69.669 68.992 48.276 48.063 47.851 47.637 47.425 47.212 46.999 39.920 39.716 27.429
CN
NC
S
F
O
N
H
O
O
8
NHBoc
^{13}C NMR (101 MHz, CD_3OD)
f1 (ppm)

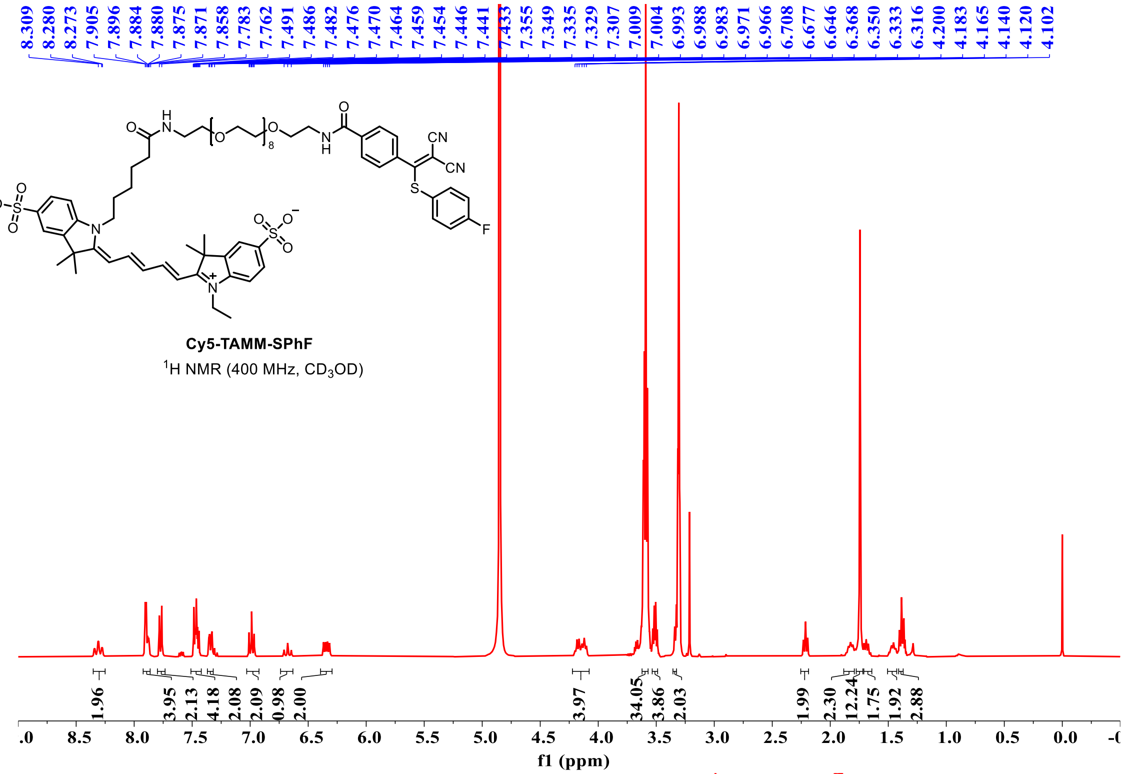
8.309 8.280 8.273 7.905 7.896 7.884 7.880 7.875 7.871 7.858 7.783 7.762 7.491 7.486 7.482 7.476 7.470 7.464 7.459 7.454 7.446 7.441 7.433 7.355 7.349 7.335 7.329 7.307 7.009 7.004 6.993 6.988 6.983 6.971 6.966 6.708 6.677 6.646 6.368 6.350 6.333 6.316 4.200 4.183 4.165 4.140 4.120 4.102
Cy5-TAMM-SPhF
^{1}H NMR (400 MHz, CD_3OD)
1.96 3.95 2.13 4.18 2.08 2.09 0.98 2.00 3.97 34.05 3.86 2.03 1.99 2.30 12.24 1.75 1.92 2.88
.0 8.5 8.0 7.5 7.0 6.5 6.0 5.5 5.0 4.5 4.0 3.5 3.0 2.5 2.0 1.5 1.0 0.5 0.0 -(
f1 (ppm)

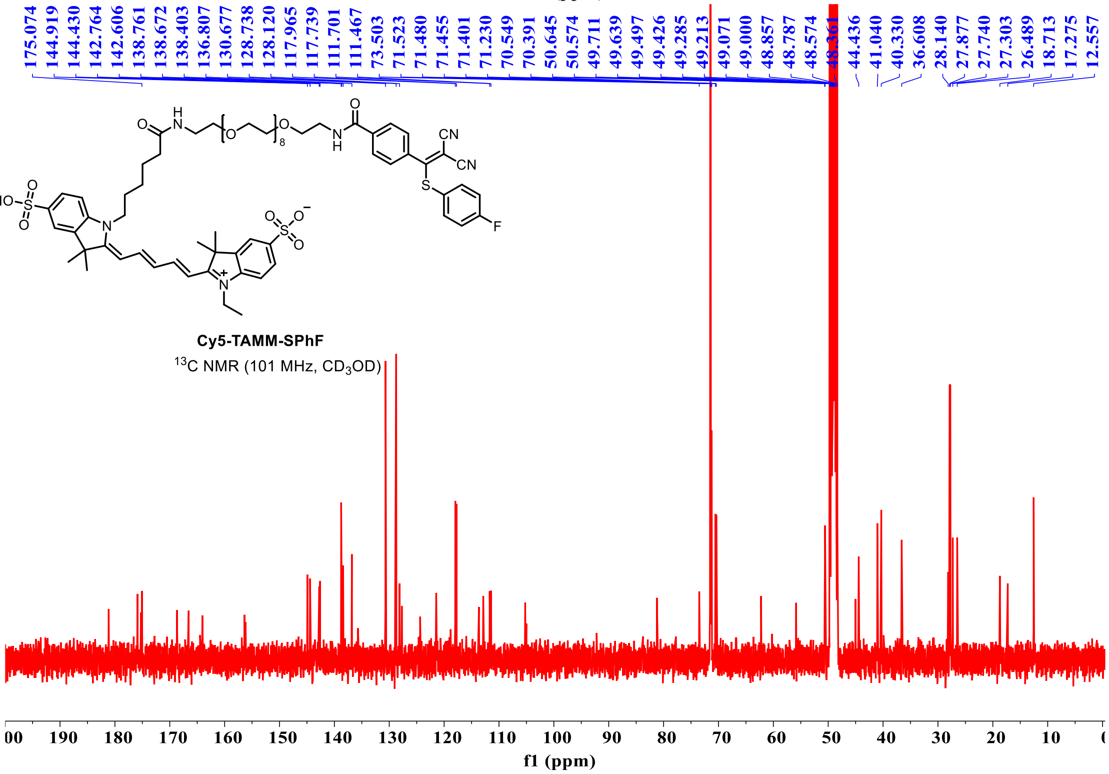
175.074 144.919 144.430 142.764 142.606 138.761 138.672 138.403 136.807 130.677 128.738 128.120 117.965 117.739 111.701 111.467 73.503 71.523 71.480 71.455 71.401 71.230 70.549 70.391 50.645 50.574 49.711 49.639 49.497 49.426 49.285 49.213 49.071 49.000 48.857 48.787 48.574 [illegible] 44.436 41.040 40.330 36.608 28.140 27.877 27.740 27.303 26.489 18.713 17.275 12.557
Cy5-TAMM-SPhF
^{13}C NMR (101 MHz, CD_3OD)
00 190 180 170 160 150 140 130 120 110 100 90 80 70 60 50 40 30 20 10 (
f1 (ppm)

<u>Synthesis of **Cy3-tetrazine**</u>

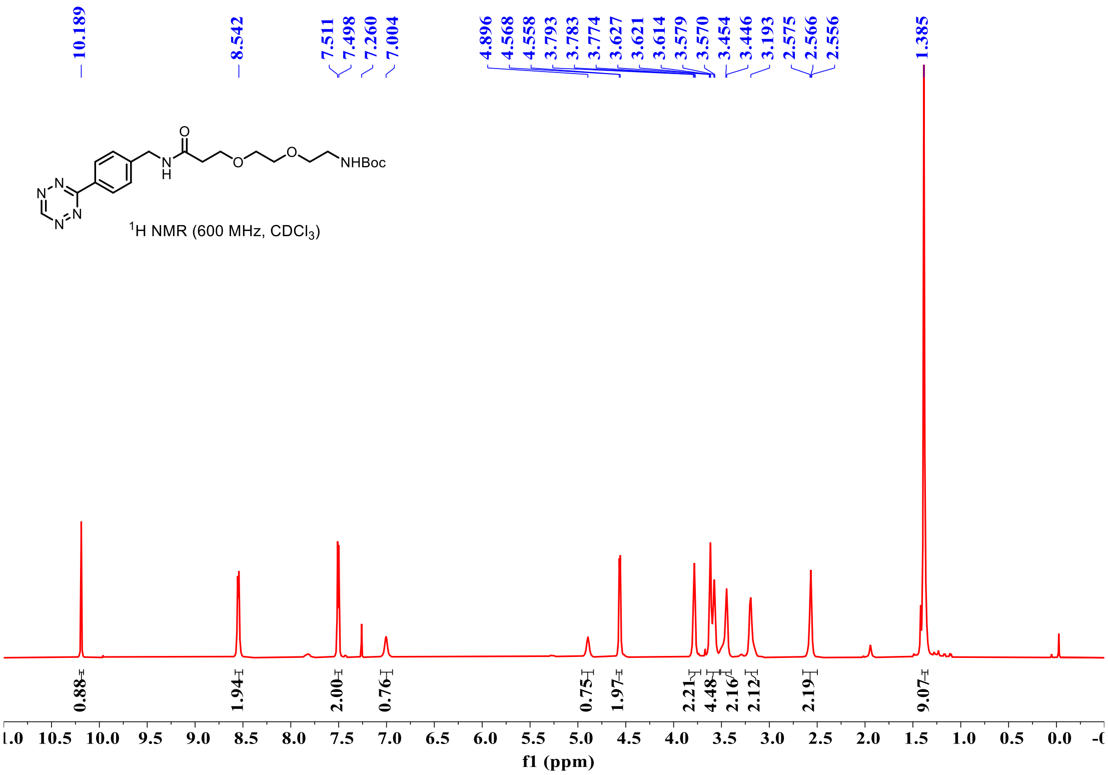

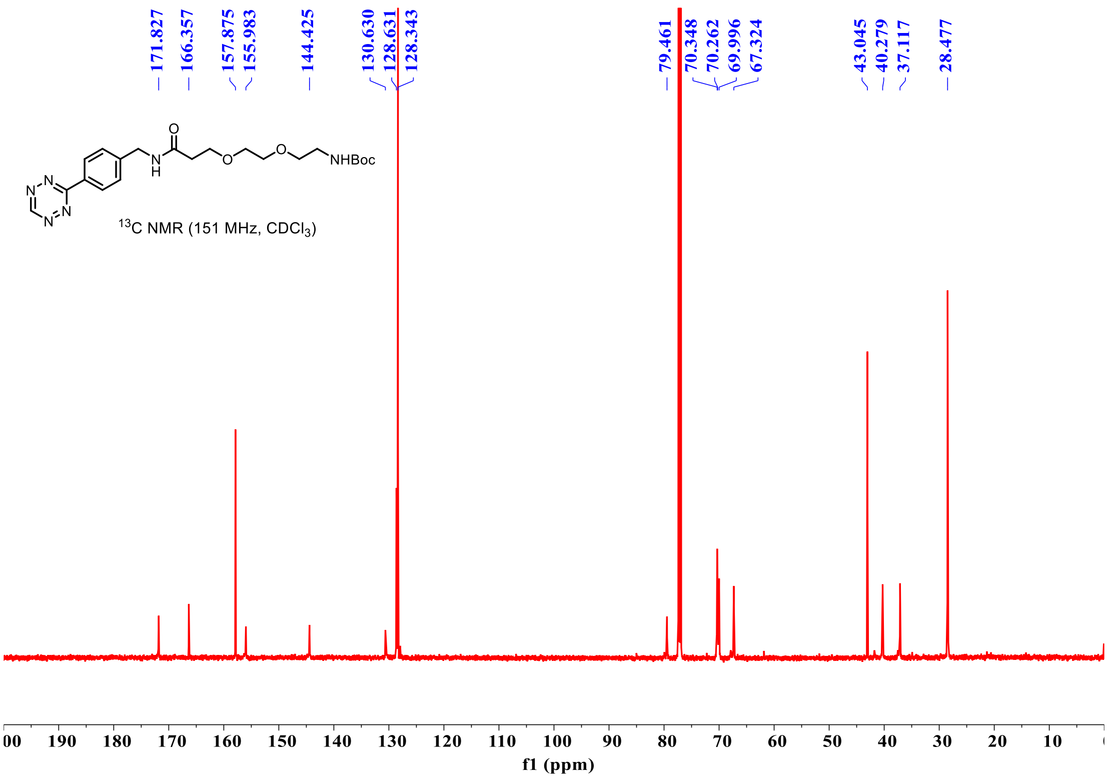

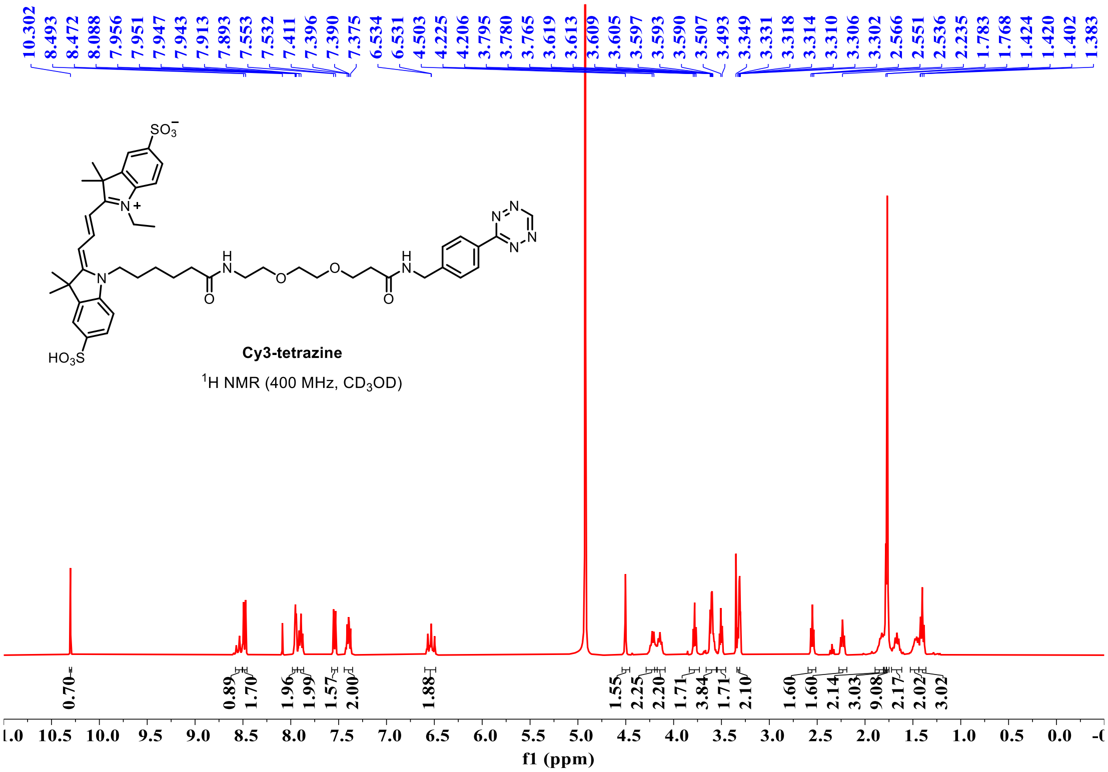
10.302 8.493 8.472 8.088 7.956 7.951 7.947 7.943 7.913 7.893 7.553 7.532 7.411 7.396 7.390 7.375 6.534 6.531 4.503 4.225 4.206 3.795 3.780 3.765 3.619 3.613 3.609 3.605 3.597 3.593 3.590 3.507 3.493 3.349 3.331 3.318 3.314 3.310 3.306 3.302 2.566 2.551 2.536 2.235 1.783 1.768 1.424 1.420 1.402 1.383
Cy3-tetrazine
1H NMR (400 MHz, CD3OD)
0.70 0.89 1.70 1.96 1.99 1.57 2.00 1.88 1.55 2.25 2.20 1.71 3.84 1.71 2.10 1.60 1.60 2.14 3.03 9.08 2.17 2.02 3.02
f1 (ppm)

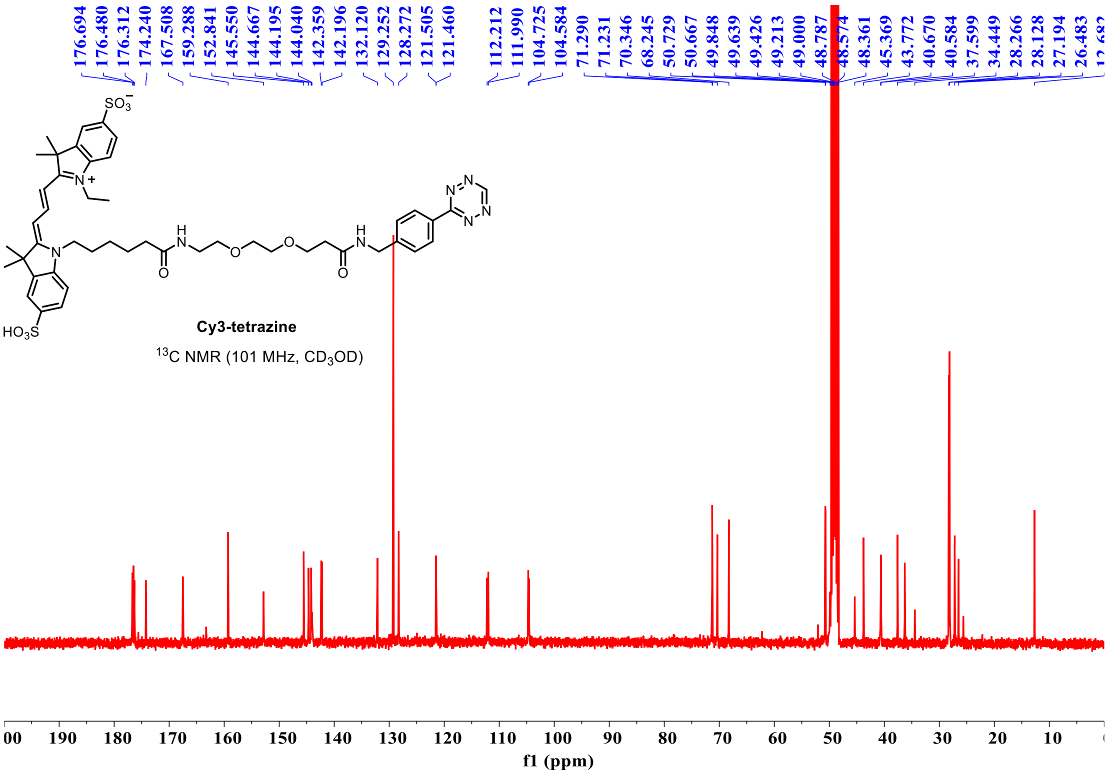
176.694 176.480 176.312 174.240 167.508 159.288 152.841 145.550 144.667 144.195 144.040 142.359 142.196 132.120 129.252 128.272 121.505 121.460 112.212 111.990 104.725 104.584 71.290 71.231 70.346 68.245 50.729 50.667 49.848 49.639 49.426 49.213 49.000 48.787 48.574 48.361 45.369 43.772 40.670 40.584 37.599 34.449 28.266 28.128 27.194 26.483
Cy3-tetrazine
13C NMR (101 MHz, CD3OD)
f1 (ppm)

Synthesis of **Cy7-tetrazine**

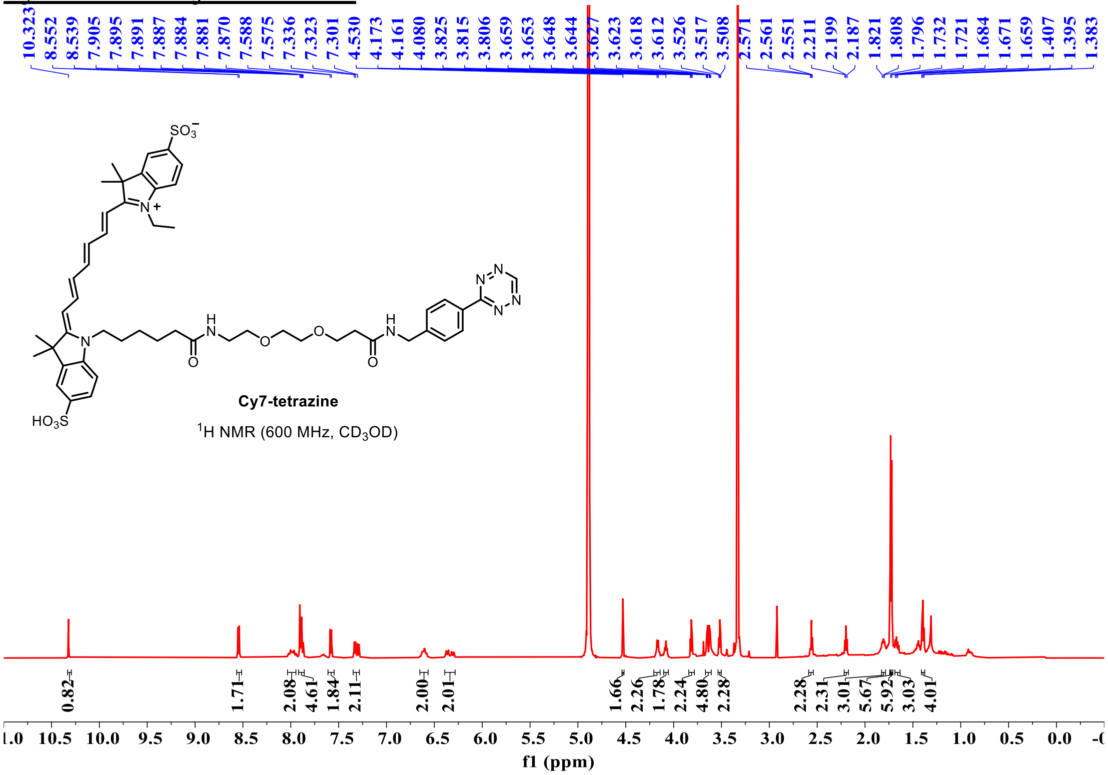

Synthesis of **FITC-N_3**

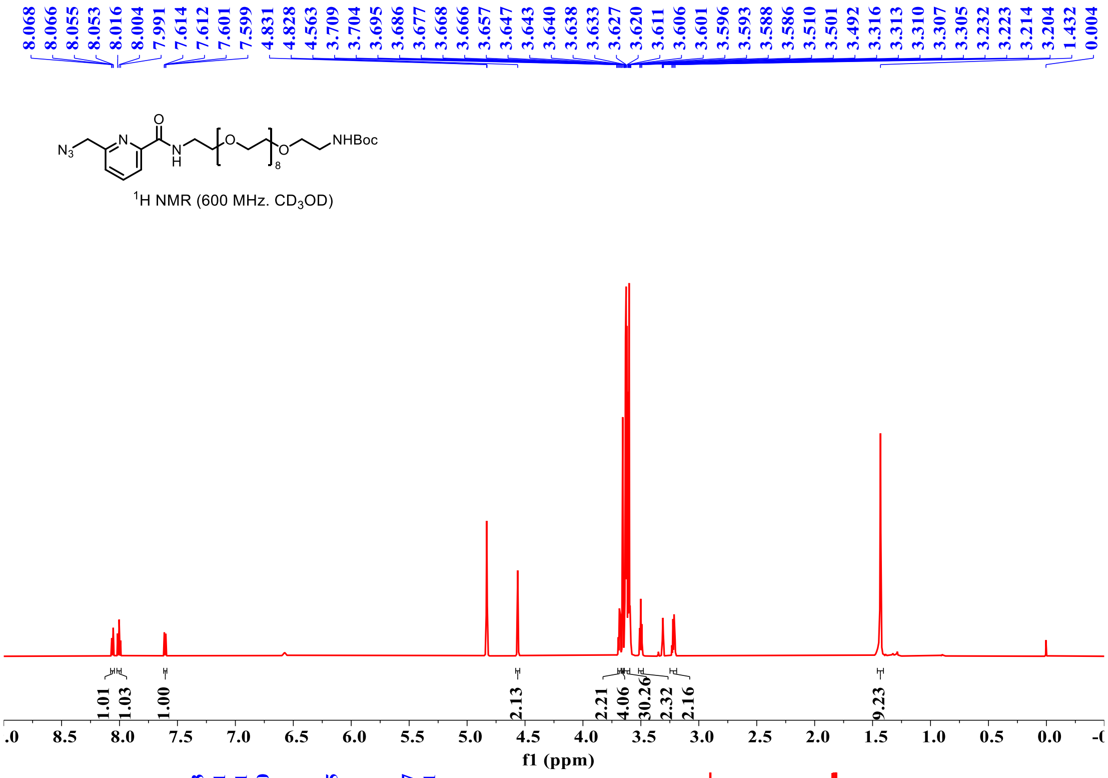

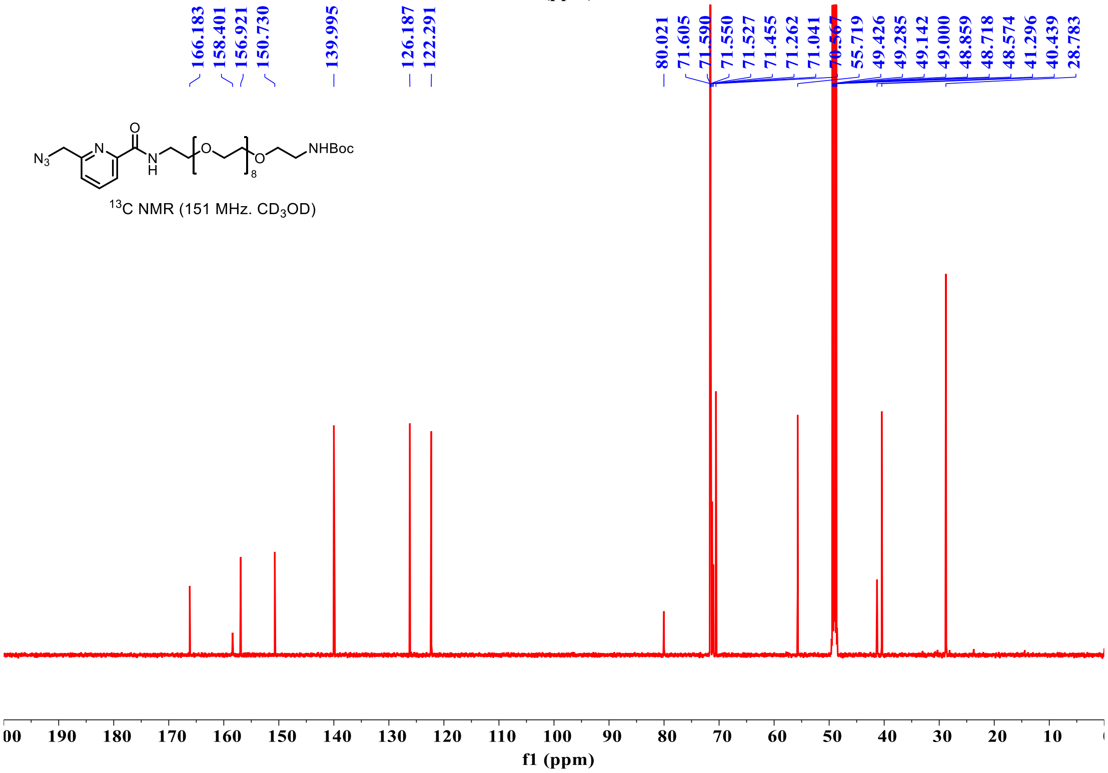

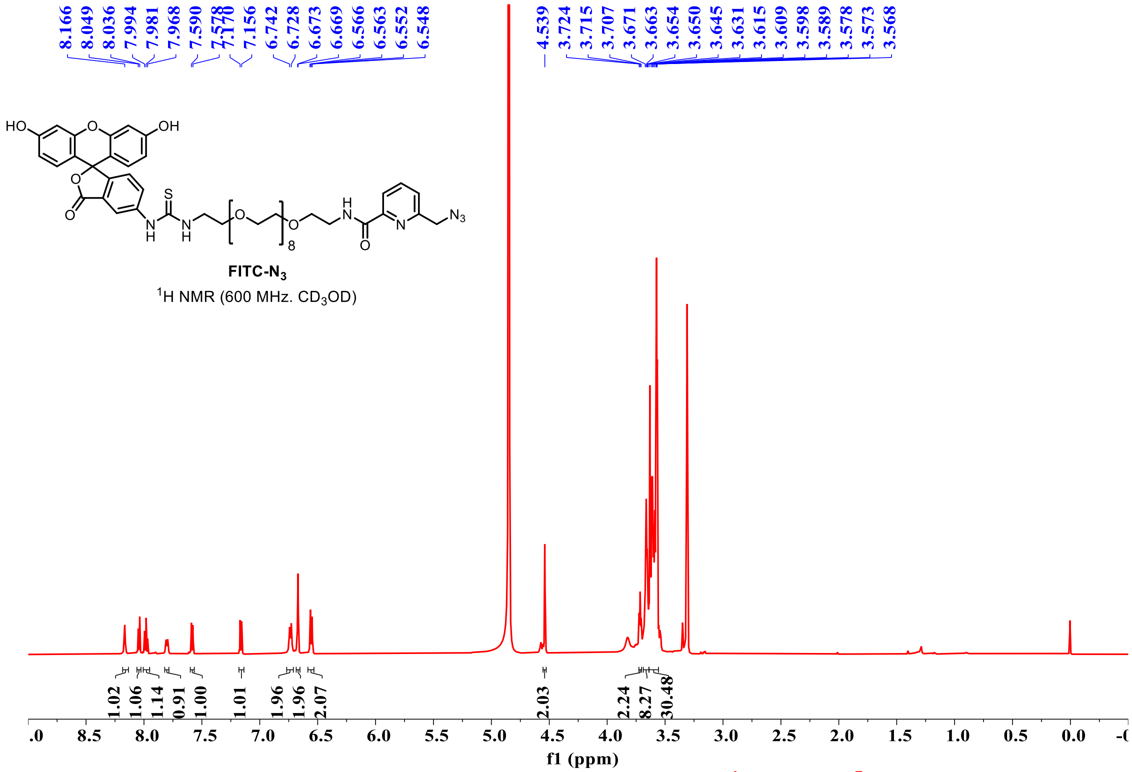
FITC-N3
1H NMR (600 MHz. CD3OD)
f1 (ppm)

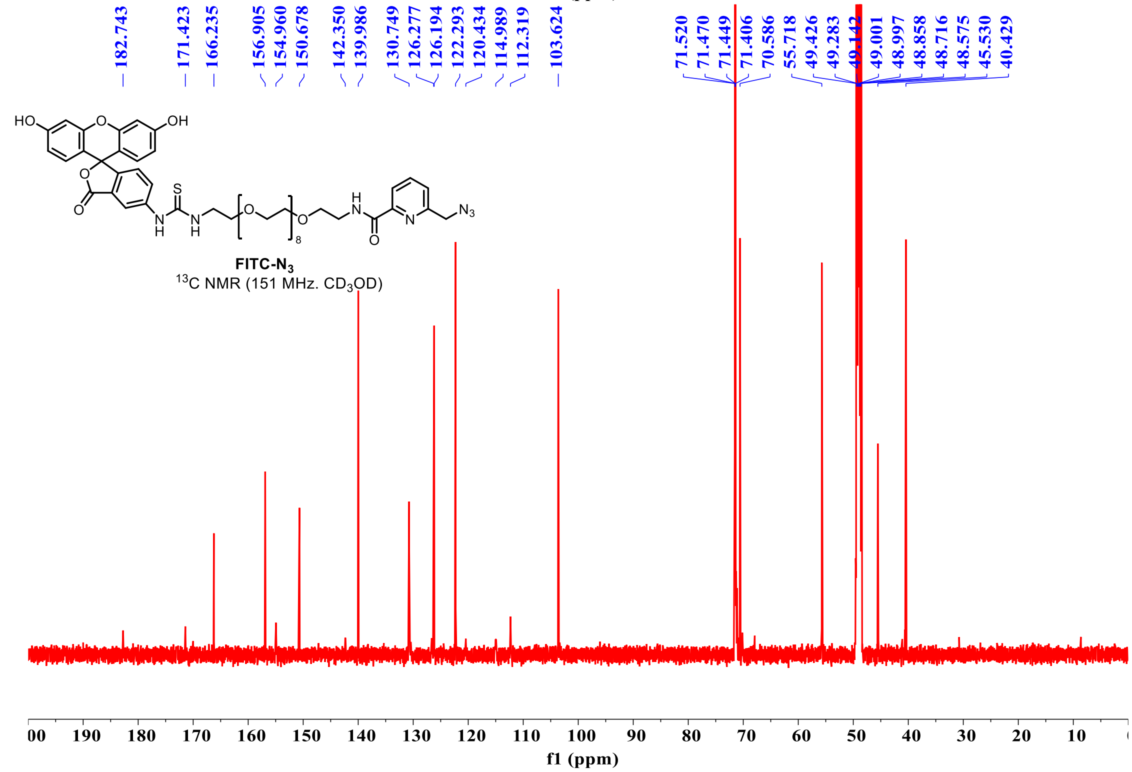
FITC-N3
13C NMR (151 MHz. CD3OD)
f1 (ppm)

Synthesis of **Pyranine-tetrazine**

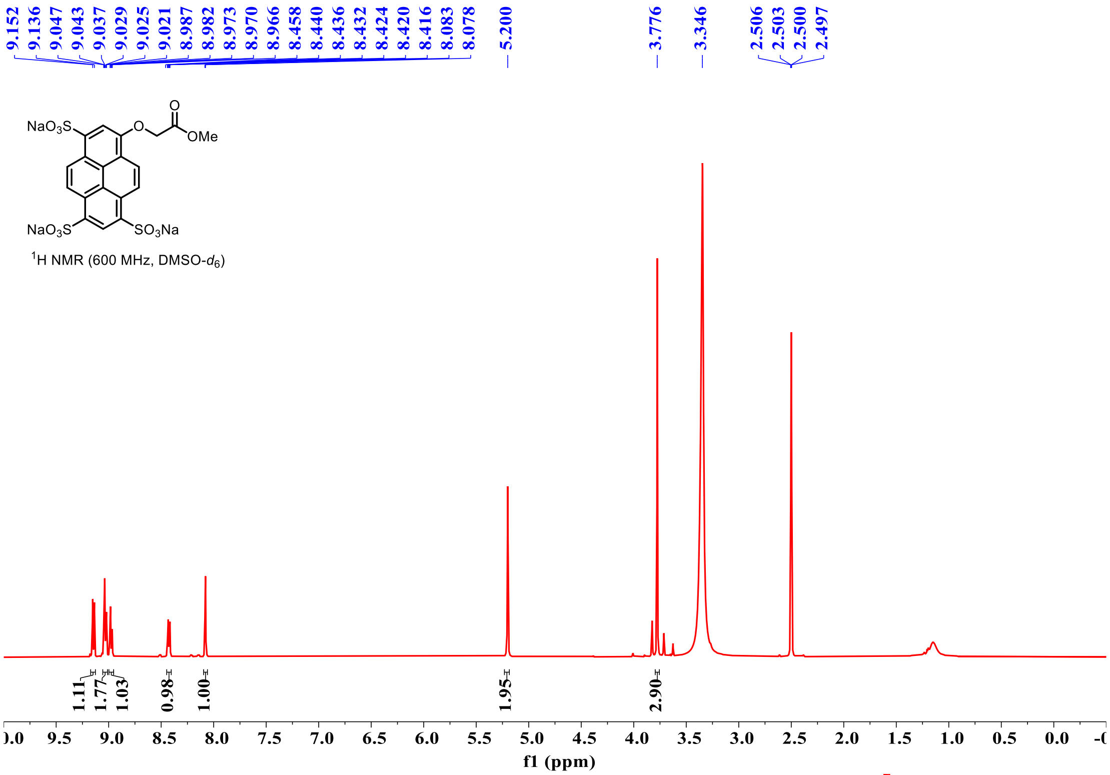

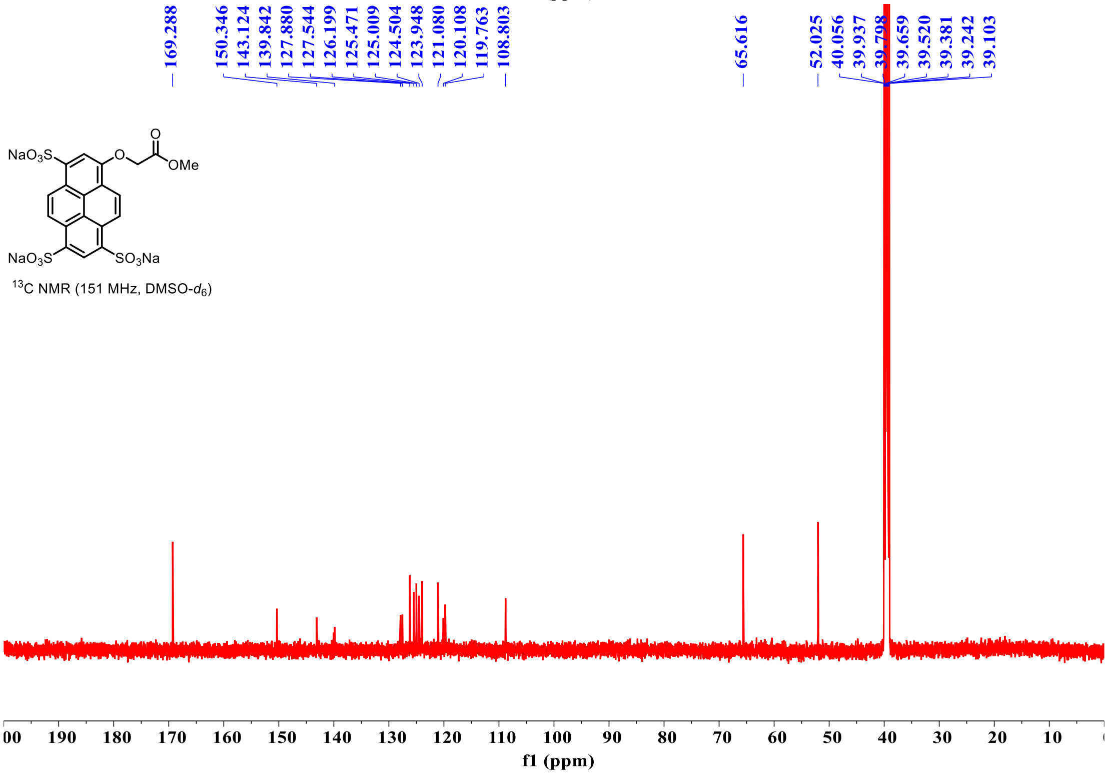

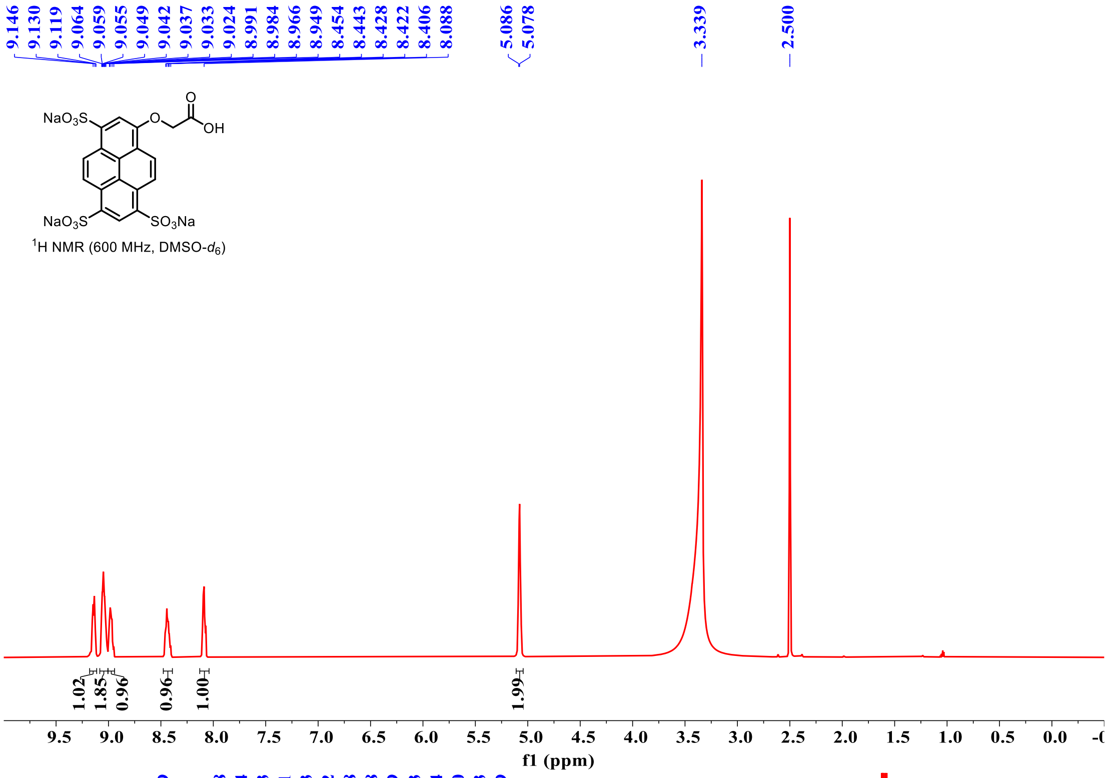
9.146
9.130
9.119
9.064
9.059
9.055
9.049
9.042
9.037
9.033
9.024
8.991
8.984
8.966
8.949
8.454
8.443
8.428
8.422
8.406
8.088
5.086
5.078
3.339
2.500
NaO_3S
O
OH
NaO_3S
SO_3Na
^1H NMR (600 MHz, DMSO-d_6)
1.02
1.85
0.96
0.96
1.00
1.99
f1 (ppm)

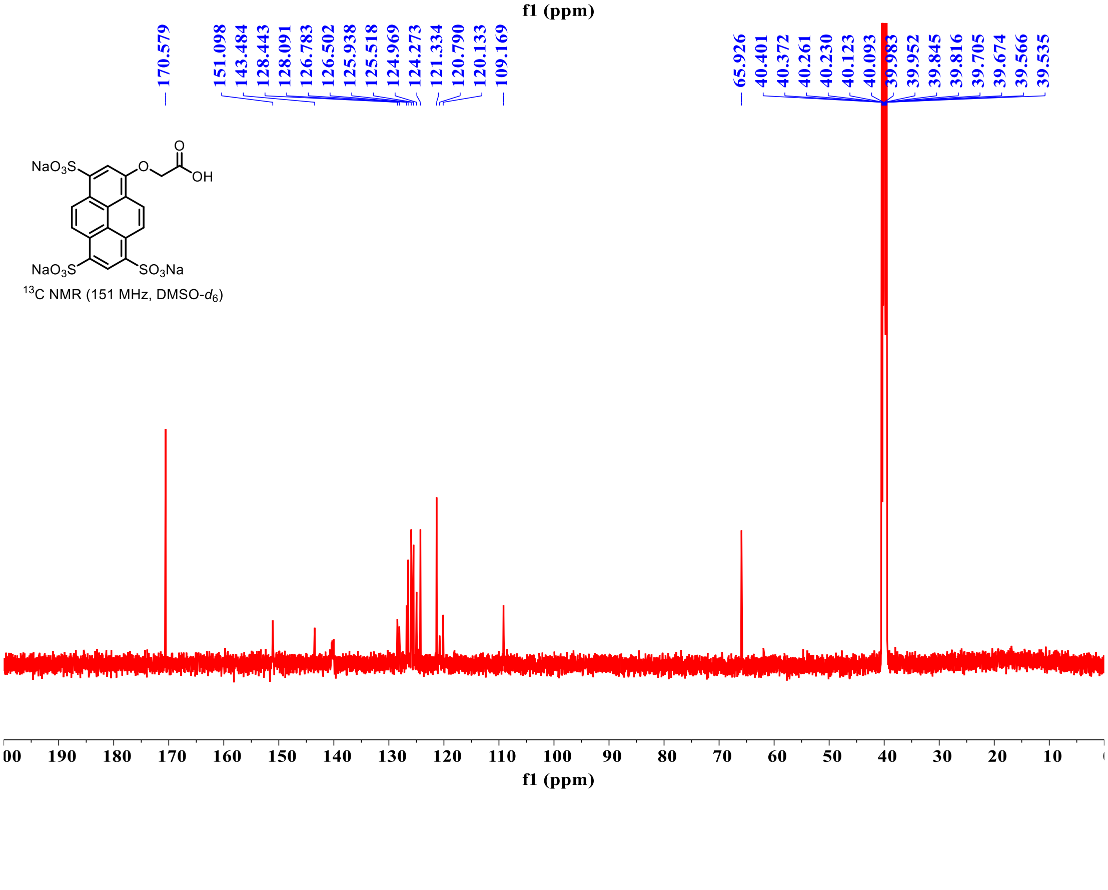
170.579
151.098
143.484
128.443
128.091
126.783
126.502
125.938
125.518
124.969
124.273
121.334
120.790
120.133
109.169
65.926
40.401
40.372
40.261
40.230
40.123
40.093
39.983
39.952
39.845
39.816
39.705
39.674
39.566
39.535
NaO_3S
O
OH
NaO_3S
SO_3Na
^{13}C NMR (151 MHz, DMSO-d_6)
f1 (ppm)

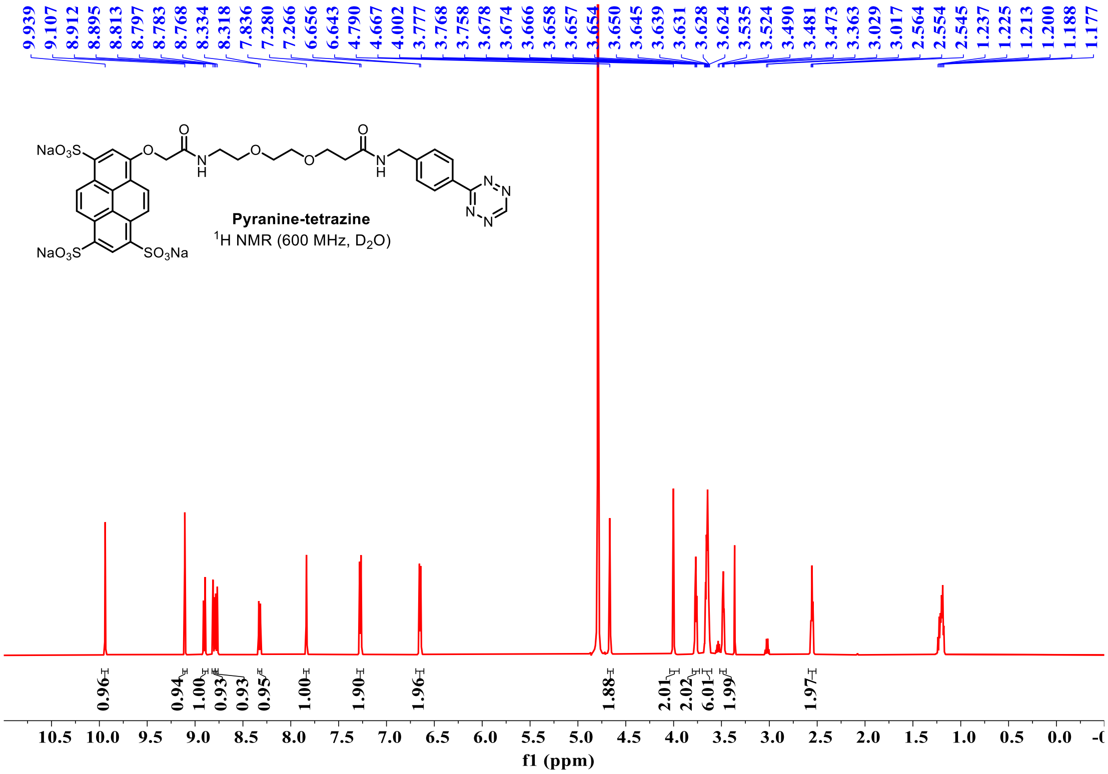
9.939 9.107 8.912 8.895 8.813 8.797 8.783 8.768 8.334 8.318 7.836 7.280 7.266 6.656 6.643 4.790 4.667 4.002 3.777 3.768 3.758 3.678 3.674 3.666 3.658 3.657 3.654 3.650 3.645 3.639 3.631 3.628 3.624 3.535 3.524 3.490 3.481 3.473 3.363 3.029 3.017 2.564 2.554 2.545 1.237 1.225 1.213 1.200 1.188 1.177
Pyranine-tetrazine
1H NMR (600 MHz, D2O)
0.96 0.94 1.00 0.93 0.93 0.95 1.00 1.90 1.96 1.88 2.01 2.02 6.01 1.99 1.97
f1 (ppm)

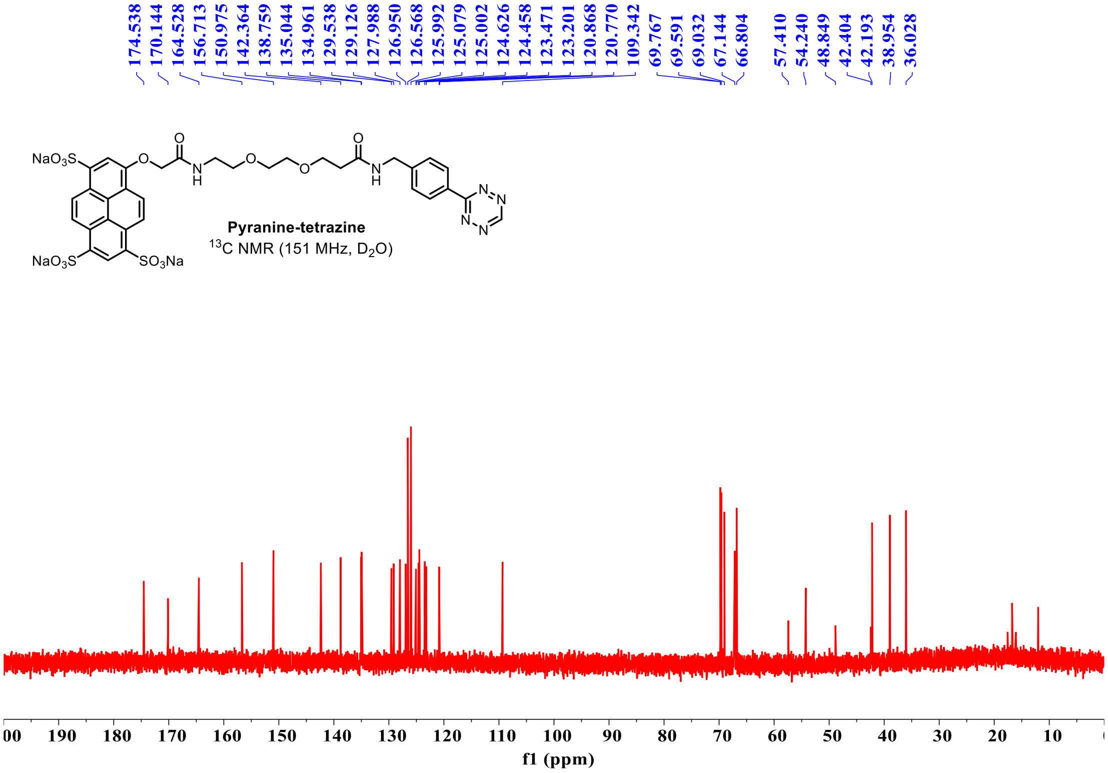
174.538 170.144 164.528 156.713 150.975 142.364 138.759 135.044 134.961 129.538 129.126 127.988 126.950 126.568 125.992 125.079 125.002 124.626 124.458 123.471 123.201 120.868 120.770 109.342 69.767 69.591 69.032 67.144 66.804 57.410 54.240 48.849 42.404 42.193 38.954 36.028
Pyranine-tetrazine
13C NMR (151 MHz, D2O)
f1 (ppm)

Synthesis of **BCN-NHS**

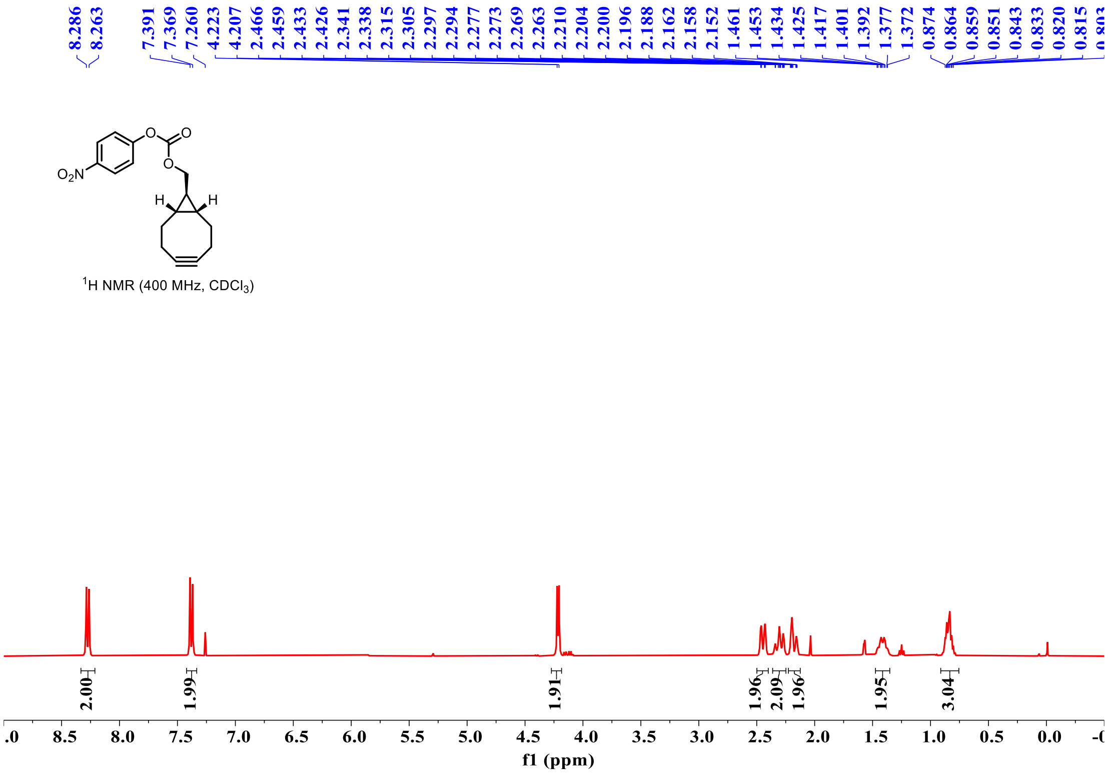

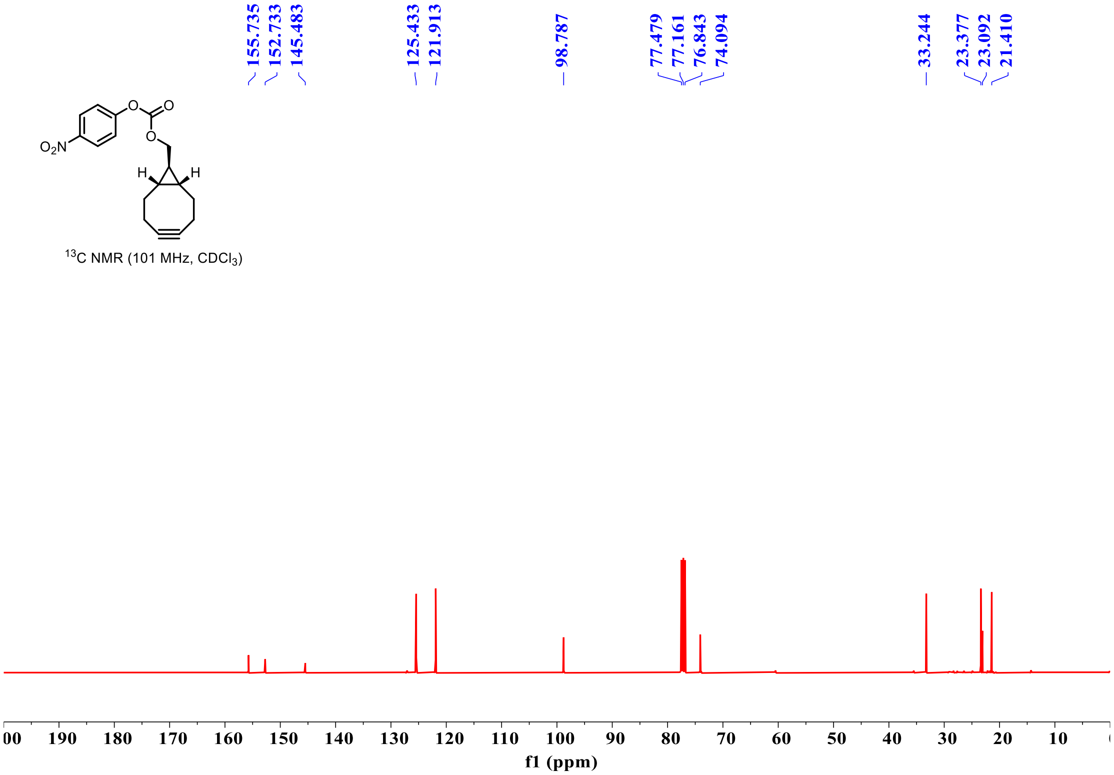

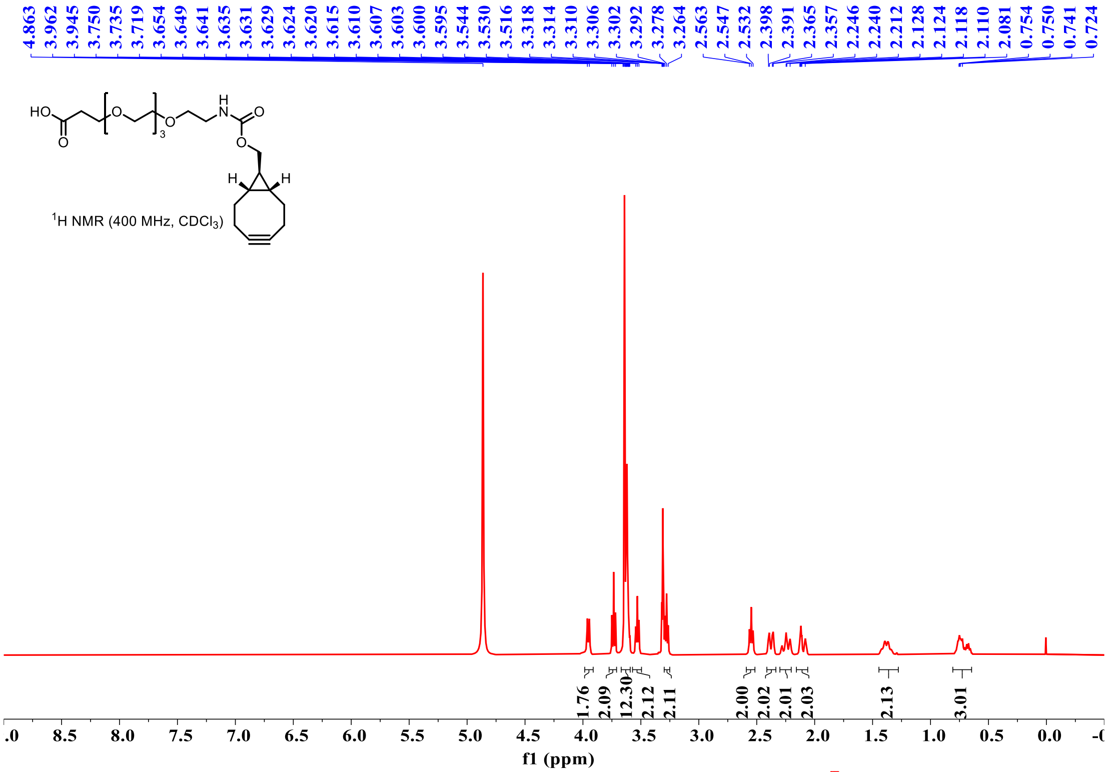
HO
O
O
3
O
H
N
O
O
H
H
1H NMR (400 MHz, CDCl3)
f1 (ppm)

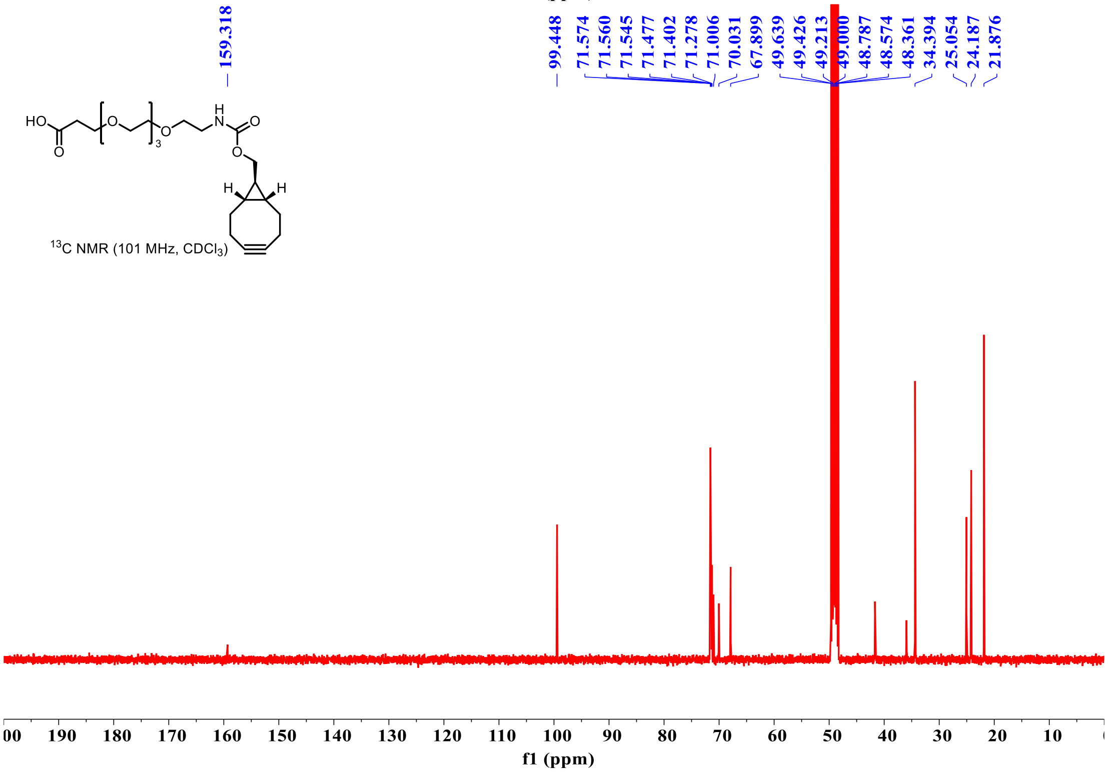
HO
O
O
3
O
H
N
O
O
H
H
13C NMR (101 MHz, CDCl3)
f1 (ppm)

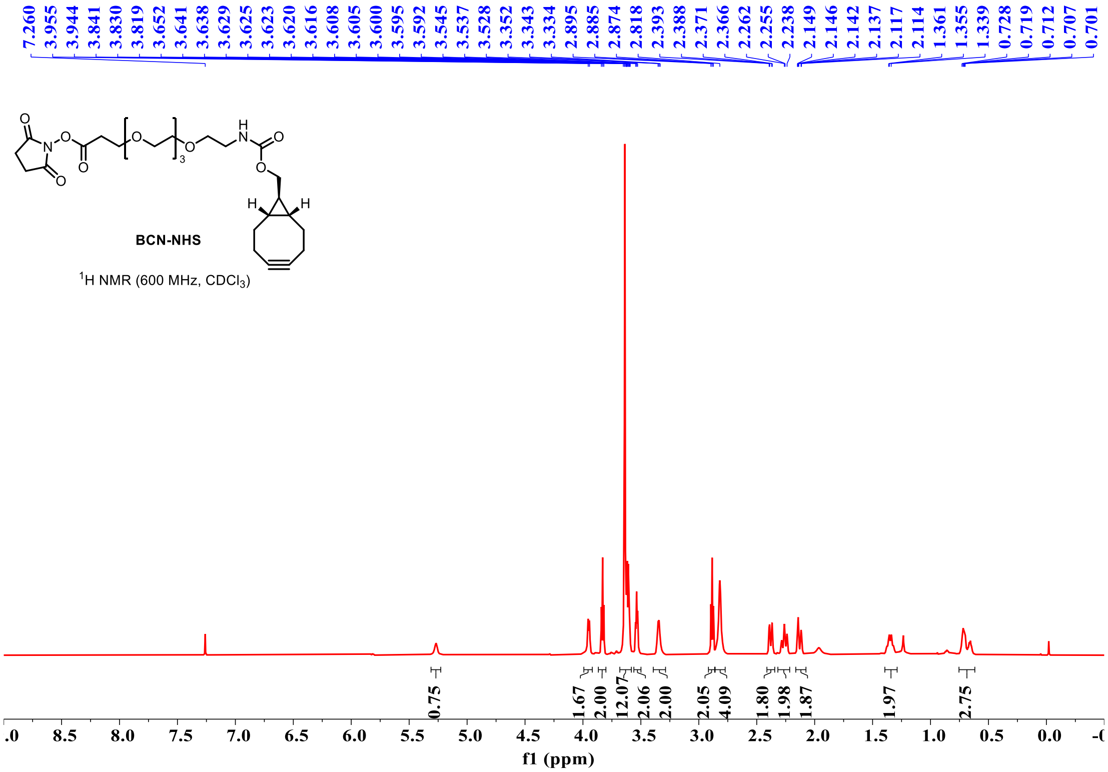
7.260 3.955 3.944 3.841 3.830 3.819 3.652 3.641 3.638 3.629 3.625 3.623 3.620 3.616 3.608 3.605 3.600 3.595 3.592 3.545 3.537 3.528 3.352 3.343 3.334 2.895 2.885 2.874 2.818 2.393 2.388 2.371 2.366 2.262 2.255 2.238 2.149 2.146 2.142 2.137 2.117 2.114 1.361 1.355 1.339 0.728 0.719 0.712 0.707 0.701
BCN-NHS
^{1}H NMR (600 MHz, $CDCl_3$)
0.75 1.67 2.00 12.07 2.06 2.00 2.05 4.09 1.80 1.98 1.87 1.97 2.75
f1 (ppm)

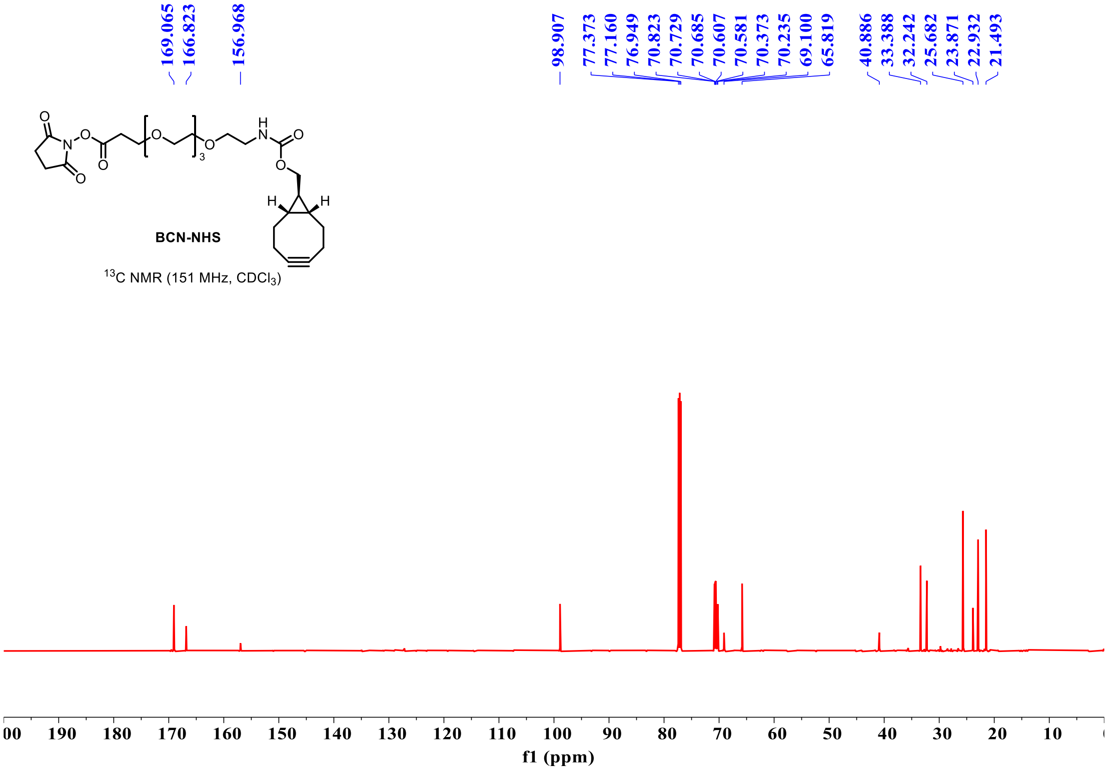
169.065 166.823 156.968 98.907 77.373 77.160 76.949 70.823 70.729 70.685 70.607 70.581 70.373 70.235 69.100 65.819 40.886 33.388 32.242 25.682 23.871 22.932 21.493
BCN-NHS
^{13}C NMR (151 MHz, $CDCl_3$)
f1 (ppm)

Synthesis of **Tetrazine-cysteine**

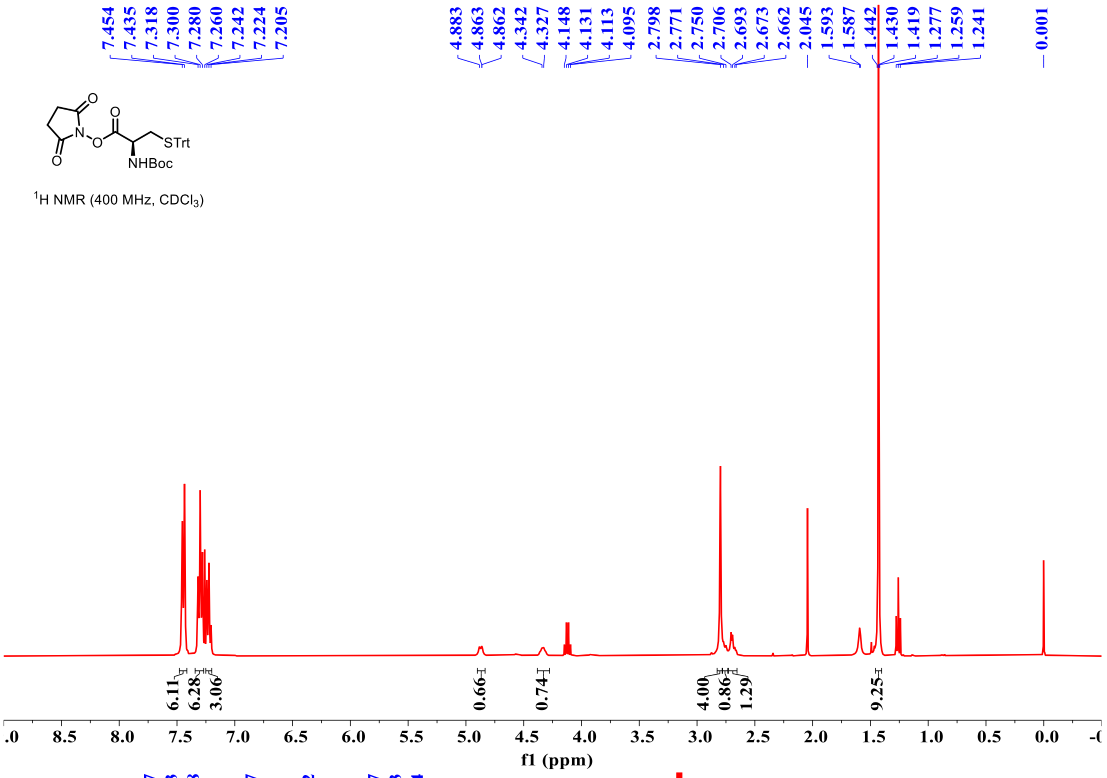

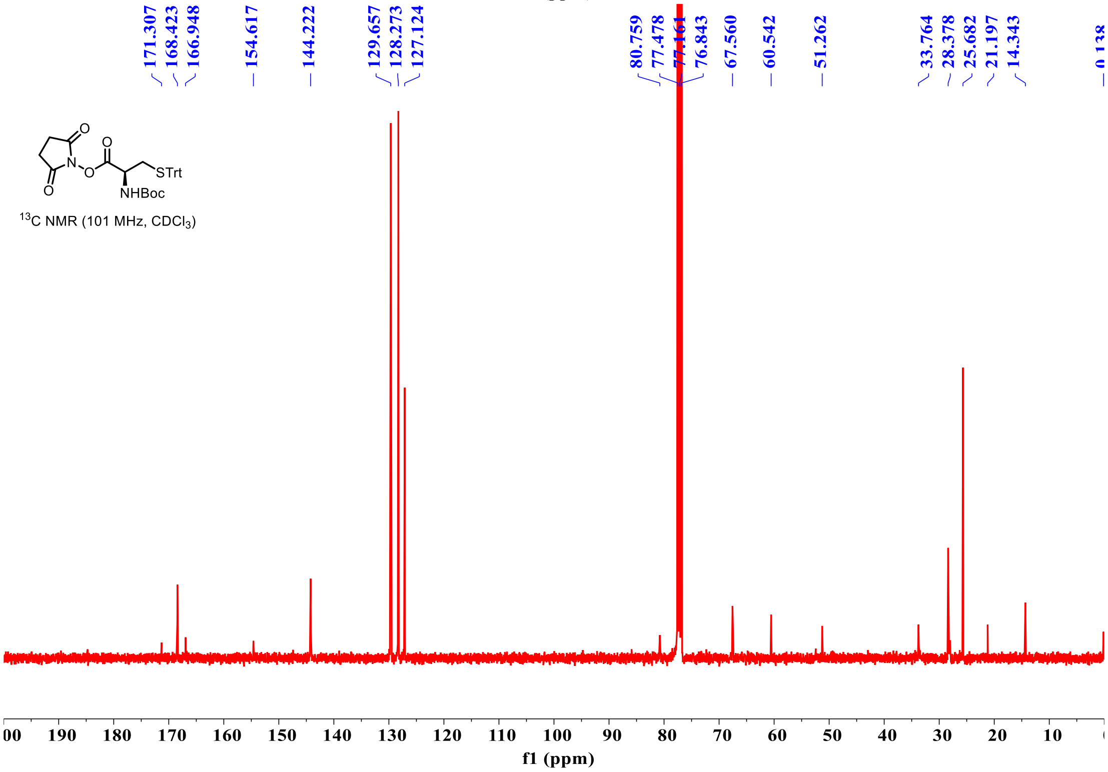

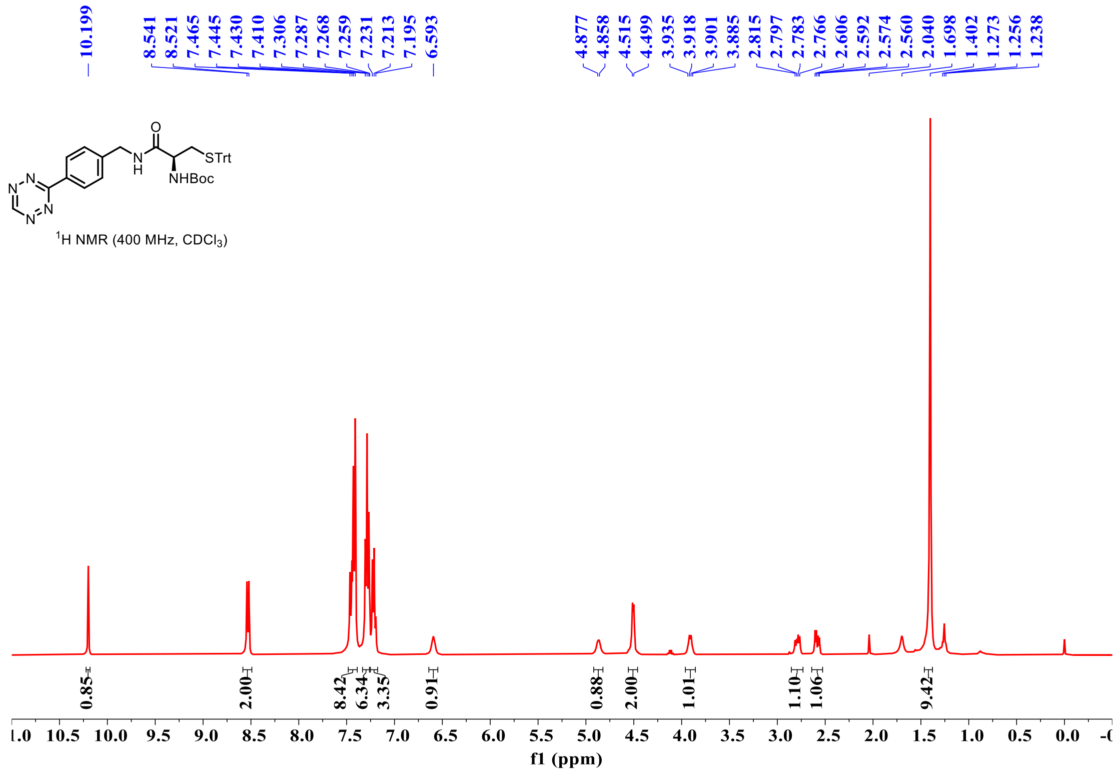
10.199
8.541 8.521 7.465 7.445 7.430 7.410 7.306 7.287 7.268 7.259 7.231 7.213 7.195 6.593
4.877 4.858 4.515 4.499 3.935 3.918 3.901 3.885 2.815 2.797 2.783 2.766 2.606 2.592 2.574 2.560 2.040 1.698 1.402 1.273 1.256 1.238
O
N
H
STrt
NHBoc
N
N
N
N
¹H NMR (400 MHz, CDCl₃)
0.85
2.00
8.42
6.34
3.35
0.91
0.88
2.00
1.01
1.10
1.06
9.42
f1 (ppm)

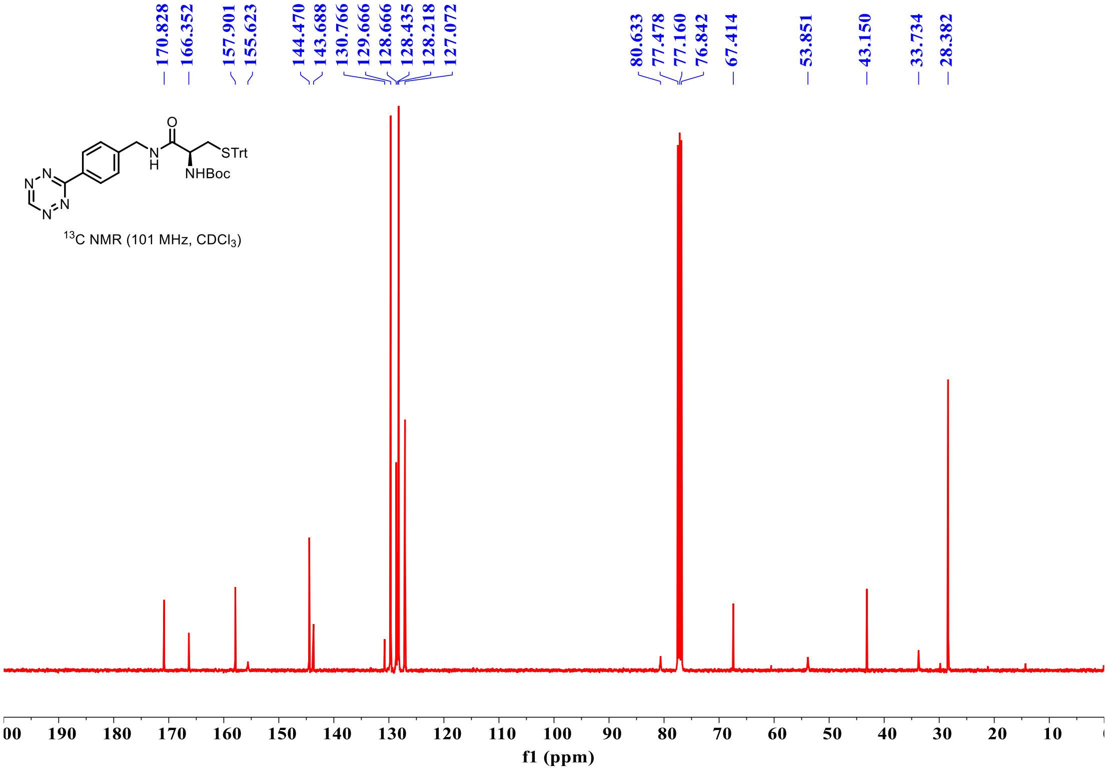
170.828 166.352 157.901 155.623 144.470 143.688 130.766 129.666 128.666 128.435 128.218 127.072
80.633 77.478 77.160 76.842 67.414 53.851 43.150 33.734 28.382
O
N
H
STrt
NHBoc
N
N
N
N
¹³C NMR (101 MHz, CDCl₃)
f1 (ppm)
